# Structure-Based Design of Novel Thiazolone[3,2-*a*]pyrimidine Derivatives as Potent RNase H Inhibitors for HIV Therapy

**DOI:** 10.3390/molecules29092120

**Published:** 2024-05-03

**Authors:** Xuan-De Zhu, Angela Corona, Stefania Maloccu, Enzo Tramontano, Shuai Wang, Christophe Pannecouque, Erik De Clercq, Ge Meng, Fen-Er Chen

**Affiliations:** 1 Engineering Center of Catalysis and Synthesis for Chiral Molecules, Department of Chemistry, Fudan University, Shanghai 200433, China; 21210220029@m.fudan.edu.cn (X.-D.Z.); shuaiwang@fudan.edu.cn (S.W.); 2 Department of Life and Environmental Sciences, Department of Applied Science Biosyst, University of Cagliari, 09042 Cagliari, Italy; angela.corona@unica.it (A.C.); stefania.maloccu@unica.it (S.M.); tramon@unica.it (E.T.); 3 Rega Institute for Medical Research, KU Leuven, Herestraat 49, B-3000 Leuven, Belgium; christophe.pannecouque@kuleuven.be (C.P.); erik.declercq@kuleuven.be (E.D.C.)

**Keywords:** thiazolone[3,2-*a*]pyrimidine, RNase H, allosteric inhibitors, 3D-QSAR

## Abstract

Ribonuclease H (RNase H) was identified as an important target for HIV therapy. Currently, no RNase H inhibitors have reached clinical status. Herein, a series of novel thiazolone[3,2-*a*]pyrimidine-containing RNase H inhibitors were developed, based on the hit compound **10i**, identified from screening our in-house compound library. Some of these derivatives exhibited low micromolar inhibitory activity. Among them, compound **12b** was identified as the most potent inhibitor of RNase H (IC_50_ = 2.98 μM). The experiment of magnesium ion coordination was performed to verify that this ligand could coordinate with magnesium ions, indicating its binding ability to the catalytic site of RNase H. Docking studies revealed the main interactions of this ligand with RNase H. A quantitative structure activity relationship (QSAR) was also conducted to disclose several predictive mathematic models. A molecular dynamics simulation was also conducted to determine the stability of the complex. Taken together, thiazolone[3,2-*a*]pyrimidine can be regarded as a potential scaffold for the further development of RNase H inhibitors.

## 1. Introduction

Acquired Immunodeficiency Syndrome (AIDS), a severe infectious disease, develops as a consequence of Human Immunodeficiency Virus (HIV) infection [1,2]. The estimated global prevalence of HIV infection in 2022 is 39.0 million, underscoring its enduring and profound impact on public health worldwide [3,4]. The current standard approach for managing HIV infection is antiretroviral therapy (ART) [5,6]. However, the prolonged administration of ART can easily lead to the emergence of drug-resistant viral strains, thereby limiting its long-term efficacy [7,8]. The development of innovative anti-HIV agents is crucial and requires more attention.

**Figure 1:**
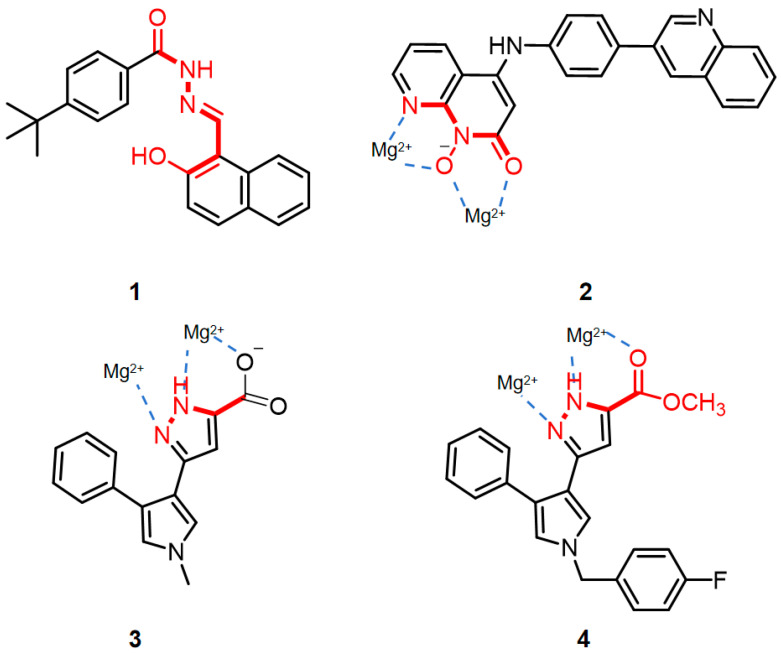
Previously disclosed representative HIV RNase H inhibitors, chelated with Mg^2+^. The dotted lines in the diagram showed the interaction of the molecule with the Mg^2+^.

In the past, targeting RNase H provided an opportunity for the development of new anti-HIV drugs [9]. The RNase H enzyme is strategically situated within the p66 subunit of reverse transcriptase (RT), enabling the precise hydrolysis of RNA in RNA/DNA heteroduplexes and tRNA precursors, to ensure seamless synthesis during the process of reverse transcription [10,11]. The active site of RNase H contains a conserved DEDD motif, comprising four carboxylate amino acid residues (D443, E478, D498, and D549), which interact with two Mg^2+^ ions [12]. Currently reported RNase H inhibitors generally function by binding to the active site competitively, through the chelation of trivalent ligands with divalent metals. Such inhibitors include hydroxyisoquinolinedione, *β*-thujaplicinol, and dihydroxycoumarin [13,14]. The potent and selective RNase H inhibition could be achieved by adopting structurally more elaborate chemotypes, such as diketoacid, pyrimidinolcarboxylic acid, hydroxynaphthyridine, and pyridopyrimidone, which featured a hydrophobic aromatic moiety that was seemingly important for antiviral activity [15]. Most of these small molecule inhibitors chelate with magnesium ions via oxygen atoms, at the active site of the RT RNase H. As an alternative, it is also possible for nitrogen atoms to form a chelation, such as naphthaldehyde hydrazone **1** [16], pyridopyrimidone **2**, and pyrrolyl pyrazoles **3**, **4** (Figure 1) [17]. Despite extensive efforts to produce RNase H inhibitors, no particular inhibitor has entered clinical trials [18,19,20]. The potential of HIV RT-associated RNase H as a therapeutic target has yet to be fully explored [21,22,23].

**Figure 2:**
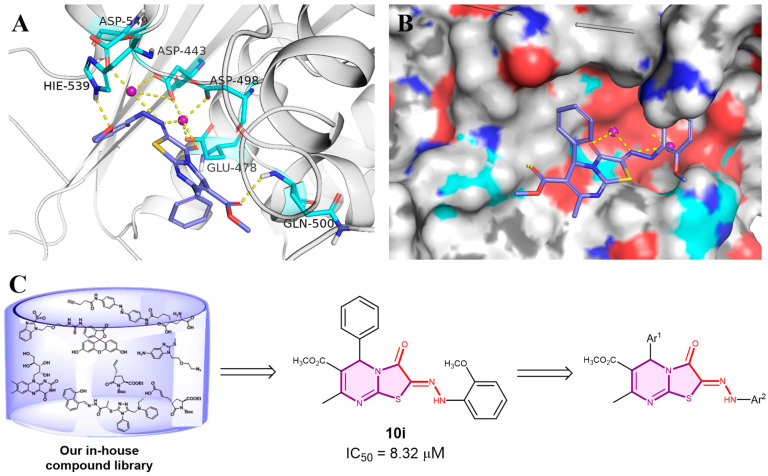
Docking poses of compounds **10i** (**A**,**B**) at the binding pocket of the HIV-1 RT (PDB code: 3QIP). (**C**) The discovery of novel thiazolone[3,2-*a*]pyrimidine derivatives.

In this work, a series of novel thiazolone[3,2-*a*]pyrimidine derivatives were designed and synthesized by using a structure-based design strategy (Figure 2). All of the target compounds were then subjected to in vitro evaluation to assess their ability to inhibit the specific enzymatic activity of recombinant RNase H. By comparing the activity results and analyzing the molecular docking and simulations, we gained some insights into the potential binding models of the compounds.

## 2. Results and Discussion

### 2.1. Chemistry

**Scheme 1:**
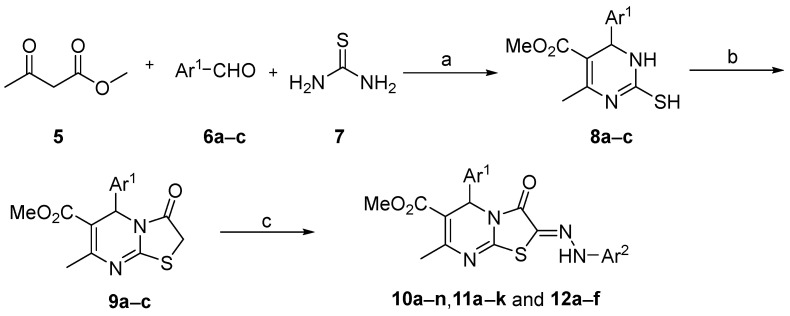
Synthesis of compounds **10a**–**n**, **11a**–**k**, and **12a**–**f**. Reagents and conditions: (**a**) SO_2_(NH_2_)_2_, EtOH, 80 °C, 2 h, yield: 70–85%; (**b**) methyl bromoacetate, pyridine, EtOH, reflux, 3 h, yield: 55–80%; (**c**) 1. ArNH_2_, NaNO_2_, HCl, H_2_O, 0 °C, 30 min; 2. pyridine, EtOH, 0 to 25 °C, 3 h, yield: 30–90%.

The synthetic route of the target compounds **10a**–**n**, **11a**–**k**, and **12a**–**f** is depicted in Scheme 1. Compounds **8a**–**c** were synthesized via the Biginelli reaction, using acetoacetic ethyl ester, thiourea, and different aromatic aldehydes as three components, catalyzed using sulfamic acid in ethanol solvent at 80 °C, yielding 70–85%. Subsequently, compounds **8a–c** were reacted with methyl bromoacetate in the presence of pyridine as the base, for 3 h under reflux in ethanol to obtain compounds **9a**–**c**, with yields ranging from 55% to 80%. Compounds **9a**–**c** were reacted with freshly prepared aromatic diazonium salts at 0 °C for 0.5 h in ethanol or dioxane. The reaction was then continued at room temperature for 2 h to obtain the final target compounds **10a**–**n**, **11a**–**k**, and **12a**–**f**, yielding 30–90%.

### 2.2. Anti-HIV-1 Activity of Compounds ***10a***–***n***, ***11a***–***k***, and ***12a***–***f***

We initiated this project by screening our-in-house compound library and compound **10i** was identified as one of the potentially active RNase H inhibitors (IC_50_ = 8.32 μM). Results from the docking study in Figure 2A,B indicated that **10i** was well positioned into the binding pocket of RNase H. The nitrogen atoms of the azo group of the compounds participate in the chelation of magnesium ions with the oxygen atoms of the thiazolone structure. The Ar^1^ and Ar^2^ aromatic segments of the molecule also approach the protein surface, through hydrophobic interactions. These findings provide important information for further structural optimization. All these newly designed thiazolone[3,2-*a*]pyrimidine derivatives were evaluated for their inhibitory activity against HIV RT-associated RNase H, using the diketo acid RDS1643 as positive control. The results are reported in Table 1. Among this series, compounds **12a**, **12b**, and **12f** were found to be the most active derivatives, with IC_50_ values ranging from 2 to 3 μM. The inhibitory activity of compounds **10d** (IC_50_ = 8.27 μM), **10g** (IC_50_ = 9.01 μM), **12b** (IC_50_ = 2.97 μM), and **12f** (IC_50_ = 3.275 μM) was stronger than others, when the polar substituents were present at the 2-position of the aryl group. The presence of polar substituted groups at the 2-position of the aromatic moiety Ar^2^ showed a significantly enhanced inhibitory activity, compared to Ar^2^ with a 3-substituted group or without any substituents on the aromatic ring. The presence of a methyl group at the 2-position of **12d** led to a minor decrease in activity (IC_50_ = 44.86 μM), suggesting that non-polar substituents on Ar^2^’s aromatic moiety may negatively impact its inhibitory activity. The decrease in activity observed in compounds containing a sulfonic acid group might be attributed to the excessively strong acidity of the sulfonic acid group, which could protonate the diazo group within the molecule. The strong solvent effect of compounds induced by large polar sulfonic acid groups might also contribute to the reduced inhibitory activity of these compounds. When Ar^2^ was changed from a phenyl group to a naphthyl group, the activity of compound **10f** gained a great improvement over that of compound **10k**. Generally, by comparing the compounds from series **10**, **11**, and **12**, we found compounds belonging to series **12** exhibited higher inhibitory activity, indicating that the Ar^1^ fragment with a single methoxy substitution at the 4-position was favorable for increasing their biological activities. And when a negatively charged polar group was present at the 2-position of Ar^2^, the inhibitory activity is enhanced.

### 2.3. QSAR Model

**Figure 3:**
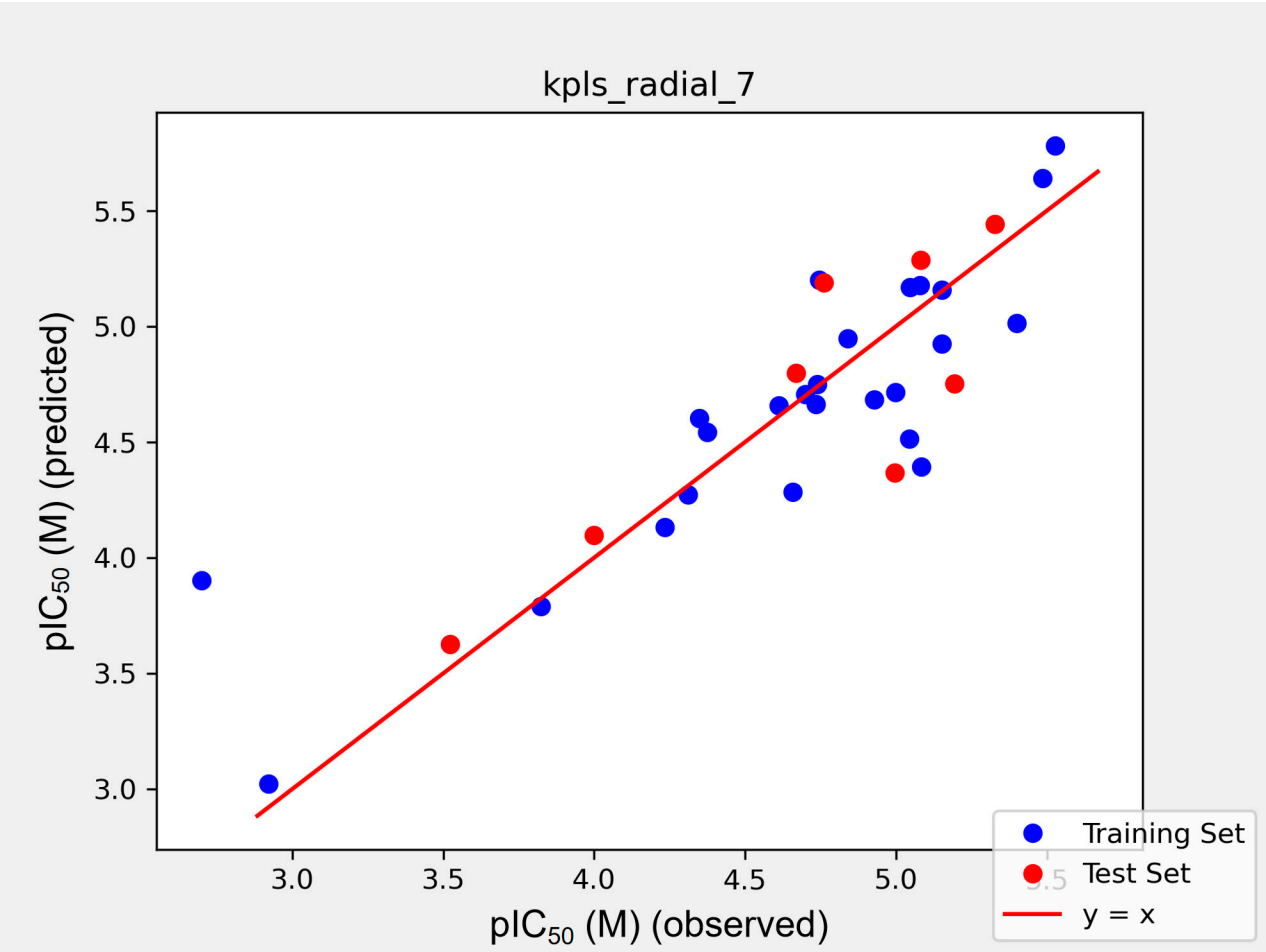
Scatter plot of the QSAR model. The blue dots in the figure are from the training set and the red dots are from the independent test set. pIC_50_ (observer) is the experimental value and pIC_50_ (predicted) is the model predicted value. The IC_50_ values used for training are from Table 1, converted to moles.

To further design HIV-1 RNase H inhibitors with improved activity, based on the molecular scaffold, we trained a QSAR model for this series of molecules to investigate the structure–activity relationship. We utilized the QSAR module in Maestro. We used pIC_50_ as the predicted value, with the unit of IC_50_ activity converted to moles. Among the obtained models, a QSAR model based on the Kernel Partial Least Squares algorithm demonstrated a good fit to both the training and test datasets. The training and prediction outcomes were depicted in Figure 3. For this mathematical model, the training set’s S.D. (standard deviation) = 0.3783, r^2^ = 0.7242, while the test Set’s rmse = 0.3287 and q^2^ = 0.6857. These results indicated that the model had a certain degree of reliability, which can guide further structural optimization.

### 2.4. Prediction of Physicochemical Properties

The values of the physicochemical parameters of compounds with good inhibitory activity were assessed using a free online forecasting tool (www.swissadme.ch/ (accessed on 31 December 2023)); they are summarized in Table 2. The compounds tested did not penetrate the blood–brain barrier, act as P-gp substrates, or inhibit CYP1A2 or CYP2D6. They were not identified as pain-inducing, indicating no interaction with multiple targets and avoiding false positive results. Notably, the representative compound **12b** complied with the Lipinski rule. Our findings confirm their safety and favorable physicochemical properties.

### 2.5. Molecular Docking

**Figure 4:**
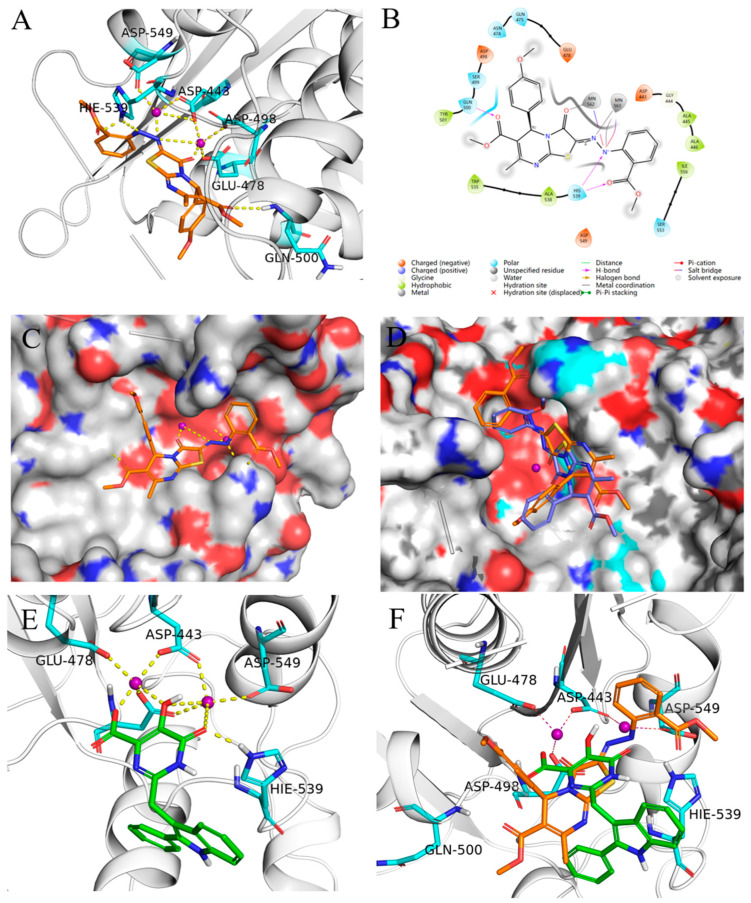
(**A**–**C**) Molecular docking of compound **12b** with RNase H (PDB code: 3QIP); (**D**) overlay of **12b** and **10i** within the binding pocket of RNase H; (**E**) the crystal structure of 5,6-dihydroxy-2-[(2-phenyl-1H-indol-3-yl)methyl]pyrimidine-4-carboxylic acid with RNase H (PDB code: 3QIP); (**F**) overlay of **12b** and 5,6-dihydroxy-2-[(2-phenyl-1H-indol-3-yl)methyl]pyrimidine-4-carboxylic acid within the binding pocket of RNase H; the Mg^2+^ ions are represented by the purple sphere. The interactions between residues, ligands, and Mg^2+^ ions are indicated by the yellow lines. The molecular scaffold of 5,6-dihydroxy-2-[(2-phenyl-1H-indol-3-yl)methyl]pyrimidine-4-carboxylic acid is green and the molecular scaffold of compound **12b** is orange. Important amino acid residues are represented in cyan.

We performed a molecular docking study of compounds **10i** and **12b** with the RNase H active site (PDB code: 3QIP) [25]. The docking results for compounds **10i** and **12b** are shown in Figure 4. The predicted binding modes of compounds **10i** and **12b** at the active site of RNase H suggested an interaction between the amide group and diazo group with Mg^2+^. On one side, Mg^2+^ ions were connected to the protein through the carboxyl groups of the protein residues Asp443, Asp549, Asp498, and GLu478. On the other side, one Mg^2+^ ion was involved in a five-membered ring chelation through the oxygen of the amide group and the nitrogen of the diazo group, while another Mg^2+^ ion interacted with one nitrogen atom in the azo group. The 2-position substituent on the Ar^2^ fragment could participate in hydrogen bonding interactions with residue HIE539. In addition, the oxygen substituent at the 2-position may interact with the Mg^2+^ ions. The significant increase in activity may be attributed to the presence of polar substituents at the 2-position of the aromatic moiety Ar^2^. The ester group on the thiazolopyrimidine core also exhibited a hydrogen bonding interaction with GLN500. The entire diazo group was situated in the hydrophilic region of the protein. The Ar^1^ and Ar^2^ fragments of compounds **10i** and **12b** were located in the hydrophobic region of the protein. The compound **12b** coordinated with one magnesium ion through the amide and azo groups, while the nitrogen atom of the azo group interacted with the other magnesium ion. Meanwhile, 5,6-dihydroxy-2-[(2-phenyl-1*H*-indol-3-yl)methyl]pyrimidine-4-carboxylic acid interacted with one magnesium ion through the carboxyl and hydroxyl groups, and its carbonyl and hydroxyl groups also interacted with the magnesium ion. Both compounds exhibited similar interactions with residue HIE539 via their carbonyl groups.

### 2.6. Dynamic Simulation

**Figure 5:**
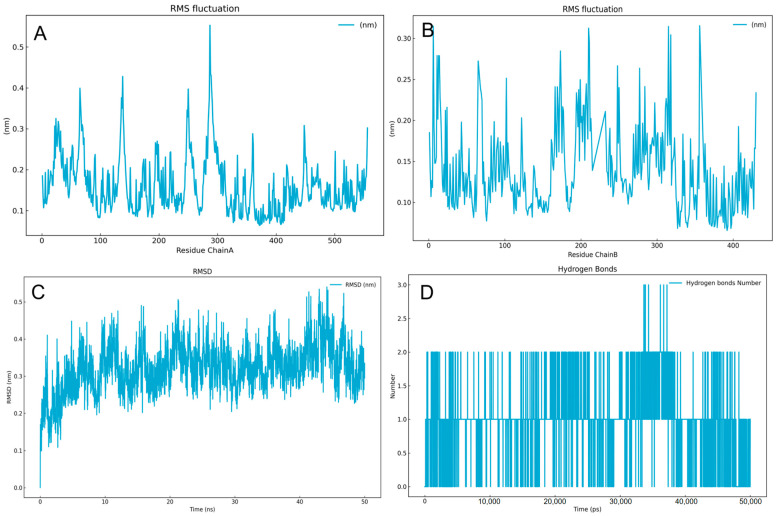
(**A**,**B**): Protein-RMSF during the process of the molecular dynamic simulation, ChainA: p66, ChainB: p51. (**C**): Ligand-RMSD. (**D**): The number of hydrogen bonds with a time range from 0 ps to 50,000 ps.

We aimed to observe the dynamic binding of the small molecule **12b** to the protein, through molecular dynamics simulations. The RMSF values of Chain were within the range of 0.1 to 0.4 nm (Figure 5A,B). The RMSD fluctuation plot (Figure 5C) indicated that the compound **12b** rapidly reached equilibrium during the dynamic binding process. The RMSD fluctuation range was within 0.25 to 0.45 nm, which was within a reasonable range. Comparison plots of three dynamic simulations are shown in the Supplementary Information (SI). Compound **12b** did not dissociate from the docking pocket during the simulation that affirmed compound **12b**’s reliability as an inhibitor for the RNase H. The hydrogen bonds (Figure 5D) showed that, apart from the coordination interactions with Mg^2+^, the dynamic changes in hydrogen bond formation between compound **12b** and the protein generally ranged from 1 to 2.

### 2.7. Magnesium Complexation

**Figure 6:**
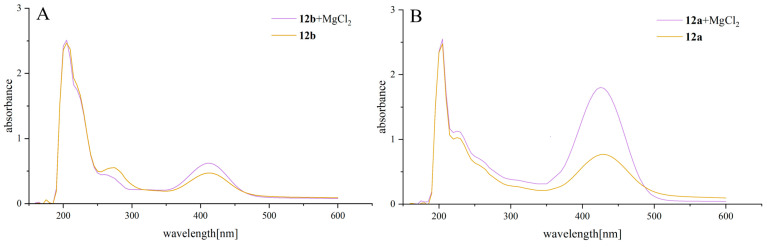
(**A**) UV spectra of **12b** in EtOH:dioxane:water = 1:1:1 = 5.21 × 10^−5^ M (orange trace) and **12b** = 5.21 × 10^−5^ M + MgCl_2_ = 10^−3^ M (purple trace). (**B**) UV spectra of **12a** in EtOH:dioxane:water = 1:1:1 = 1.24 × 10^−4^ M (orange trace) and **12a** = 1.24 × 10^−4^ M + MgCl_2_ = 10^−3^ M (purple trace).

To investigate the potential importance of the interaction between the active compounds and Mg^2+^, spectrophotometric complexation studies were carried out on the good active derivatives, **12a** and **12b**. In Figure 6, it can be observed that, upon the addition of magnesium ions, the absorbance of the solution increased around the wavelength range of 400–450 nm, indicating a certain interaction between compounds **12a** and **12b** with magnesium ions.

## 3. Conclusions

In this study, we have identified a novel class of thiazolone[3,2-*a*]pyrimidine-containing RNase H inhibitors, derived from the hit compound **10i**, by using a structure-based design strategy. Some of these compounds had IC_50_ values in the low micromolar range. The structure–activity relationship studies suggested that series **12** exhibited the best activity, with compound **12b** displaying the highest inhibitory activity against RNase H (IC_50_ = 2.98 μM). Furthermore, the presence of electron-rich substituents at the 2-position of Ar^2^ was favorable for increasing the ligand activity. The presence of aromatic fused-ring substituents appears to enhance RNase H inhibition activity, suggesting a direction for further design of inhibitor molecules. The QSAR model was also established to give a predictive guide, which inferred their potential MOA with RNase H. We further validated the reliability of this series of molecules in binding to the RNase H site through molecular docking, dynamic simulations, and Mg^2+^ coordination experiments. In summary, compound **12b** can be used as a lead compound for the further development of potent thiazolone[3,2-*a*]pyrimidine-based RNase H inhibitors.

## 4. Experimental

### 4.1. Chemistry

Commercially available reagents and solvents were procured for use. Thin-layer chromatography (TLC) (Yantai Xinnuo new material Technology Co., Ltd., Yantai, China) analysis was conducted using a 0.25 mm silica gel plate, followed by visualization under UV light with a wavelength of 254 nm. The ^1^H NMR and ^13^C NMR spectra were analyzed in DMSO-*d*_6_ or CDCl_3_, using a Bruker AV-400 spectrometer, with TMS serving as the internal standard for characterization. Due to the low solubility of certain compounds, we had to select relatively more favorable deuterated solvents for different compounds. The determination of the melting point was conducted using the Hanon MP-430 digital melting point instrument. The HRMS data were acquired using the Agilent 1290II-6545 spectrometer (Q-TOF). Analysis of sample purity was performed on a Shimadzu SPD-20A/20AV HPLC system using an Inertsil ODS-SP, 5 μm C18 column (150 mm × 4.6 mm).

#### 4.1.1. General Procedure for the Synthesis of Compounds **8a**–**c**

To a mixture of sulfourea (55 mmol, 1.1 eq), methyl acetoacetate (60 mmol, 1.2 eq), and the proper aromatic aldehyde (50 mmol, 1 eq) in EtOH (100 mL), 5 mmol (0.1 eq) sulfonamide was added as catalyst. The mixture was heated to 50 °C for 2 h, which was monitored using TLC. In total, 100 mL of water was added to terminate the reaction and then solid precipitates were observed. The precipitated solid was filtered, recrystallized twice using 30 mL ethanol each time, and dried to obtain the pure product.

#### 4.1.2. General Procedure for the Synthesis of Compounds **9a** and **9c**

The proper methyl 2-mercapto-4-methyl-6-aryl-1,6-dihydropyrimidine-5-carboxylate (32 mmol, 1 eq) and pyridine (35.2 mmol, 1.1 eq) were added to 100 mL EtOH. Then, 35.2 mmol (1.1 eq) methyl bromoacetate was added to the system, which was stirred under reflux for 3 h. Upon completion of the reaction, monitored using TLC, 100 mL water was added to terminate the reaction, affording solid precipitates, which were then filtrated and purified using gel column chromatography to give the pure products.

*Methyl 7-methyl-3-oxo-5-phenyl-2,3-dihydro-5H-thiazolo[3,2-a]pyrimidine-6-carboxylate* (**9a**). Yellow solid; ^1^H NMR (CDCl_3_, 400 MHz): δ [ppm] 7.23–6.98 (m, 5H), 5.84 (s, 1H), 3.63 (d, *J* = 17.5 Hz, 1H), 3.51 (d, *J* = 17.5 Hz, 1H), 3.42 (s, 3H), 2.26 (s, 3H).
*Methyl 5-(4-methoxyphenyl)-7-methyl-3-oxo-2,3-dihydro-5H-thiazolo[3,2-a]pyrimidine-6-carboxylate* (**9c**). Yellow solid; ^1^H NMR (CDCl_3_, 400 MHz): δ 7.29 (d, *J* = 8.6 Hz, 2H), 6.83 (d, *J* = 8.7 Hz, 2H), 6.02 (s, 1H), 3.84 (d, *J* = 17.4 Hz, 1H), 3.78 (s, 3H), 3.73 (d, *J* = 17.5 Hz, 1H), 3.64 (s, 3H), 2.48 (s, 3H).

#### 4.1.3. General Procedure for the Synthesis of Compound **9b**

Methyl 2-mercapto-4-methyl-6-(2,3,4-trimethoxyphenyl)-1,6-dihydropyrimidine-5-carboxylate (16mmol, 1 eq) and pyridine (17.6 mmol, 1.1 eq) were added to 50 mL dioxane. Then, 17.6 mmol (1.1 eq) methyl bromoacetate was added into the system. The resulting mixtures was stirred under reflux for 3 h and then TLC was used to monitor the reaction. A total of 50 mL water was added to terminate the reaction, then an extraction with ethyl acetate (3 × 50 mL) was performed. The organic layer was dried over anhydrous sodium sulfate and was concentrated under vacuum. The pure product was then obtained through purification using silica gel column chromatography.

*Methyl 7-methyl-3-oxo-5-(2,3,4-trimethoxyphenyl)-2,3-dihydro-5H-thiazolo[3,2-a]pyrimidine-6-carboxylate* (**9b**). Yellow solid; ^1^H NMR (CDCl_3_, 400 MHz): δ [ppm] 7.03 (d, *J* = 8.6 Hz, 1H), 6.58 (d, *J* = 8.7 Hz, 1H), 6.09 (s, 1H), 3.88 (s, 3H), 3.83 (s, 3H), 3.81 (s, 3H), 3.64 (s, 3H), 2.39 (s, 3H).

#### 4.1.4. General Procedure for the Synthesis of Compounds **10a**–**n** and **12a**–**f**

The proper methyl 7-methyl-3-oxo-5-aryl-2,3-dihydro-5*H*-thiazolo[3,2-*a*]pyrimidine-6-carboxylate (1 mmol, 1 eq) and pyridine (4 mmol, 4 eq) were dissolved in 5 mL EtOH and stirred at 0 °C. The appropriate aromatic amine (1.2 mmol, 1.1 eq) was dissolved in hydrochloric acid (3 mmol, 1 eq) aqueous solution and stirred at 0 °C. Then, a solution of sodium nitrite (1.44 mmol, 1.44 eq) was added to the hydrochloric acid–aromatic amine mixture. The above aqueous solution was stirred for 0.5 h at 0 °C to obtain the aromatic diazonium salt. The resulting aqueous solution was then added dropwise into the ethanol system and stirred for 1 h at 0 °C, followed by 3 h at room temperature, before being quenched with water (10 mL). The crude product obtained from precipitation of a solid was purified using silica gel column chromatography to yield the final product.

*Methyl 2-(2-(2-hydroxyphenyl)hydrazineylidene)-7-methyl-3-oxo-5-phenyl-2,3-dihydro-5H-thiazolo[3,2-a]pyrimidine-6-carboxylate* (**10a**). Yellow solid, yield: 75%, mp: 227–229 °C; ^1^H NMR (400 MHz, CDCl_3_) δ 7.43 (d, *J* = 7.0 Hz, 2H), 7.34 (t, *J* = 8.1 Hz, 4H), 6.90 (dd, *J* = 17.3, 7.9 Hz, 3H), 6.25 (s, 1H), 3.69 (s, 3H), 2.56 (s, 3H). ^13^C NMR (101 MHz, CDCl_3_ + CD_3_OD) δ 169.99, 165.12, 158.27, 155.99, 148.44, 143.42, 132.78, 132.67, 131.74, 131.64, 127.66, 124.38, 119.38, 119.03, 117.17, 113.45, 59.07, 55.51, 26.23. HRMS (ESI) *m*/*z*: calcd for C_21_H_18_N_4_O_4_S, [M − H]^−^: 421.0969; found: 421.0972.
*(2-(6-(Methoxycarbonyl)-7-methyl-3-oxo-5-phenyl-5H-thiazolo[3,2-a]pyrimidin-2(3H)-ylidene)hydrazineyl)-3-methylbenzoic acid* (**10b**). Yellow solid, yield: 88%, mp: 261–263 °C; ^1^H NMR (400 MHz, DMSO-*d*_6_) δ 11.60 (s, 1H), 7.77 (d, *J* = 6.4 Hz, 1H), 7.45 (d, *J* = 7.4 Hz, 1H), 7.40–7.27 (m, 5H), 7.10 (t, *J* = 7.7 Hz, 1H), 6.02 (s, 1H), 3.60 (s, 3H), 2.40 (s, 3H), 2.39 (s, 3H). ^13^C NMR (101 MHz, DMSO-*d*_6_) δ 170.42, 166.07, 160.50, 155.59, 152.61, 145.89, 141.48, 135.24, 129.77, 129.22, 128.87, 127.63, 126.41, 123.47, 121.49, 118.56, 108.42, 54.54, 51.86, 23.08, 22.26. HRMS (ESI) *m*/*z*: calcd for C_23_H_20_N_4_O_5_S, [M + H]^+^: 465.1255; found: 465.1262.
*Methyl 7-methyl-3-oxo-5-phenyl-2-(2-(3,4,5-trimethoxyphenyl)hydrazineylidene)-2,3-dihydro-5H-thiazolo[3,2-a]pyrimidine-6-carboxylate* (**10c**). Yellow solid, yield: 85%, mp: 211–213 °C; ^1^H NMR (400 MHz, DMSO-*d*_6_) δ 10.90 (s, 1H), 7.38–7.26 (m, 5H), 6.52 (s, 2H), 6.01 (s, 1H), 3.74 (s, 6H), 3.59 (d, *J* = 2.7 Hz, 6H), 2.38 (s, 3H). ^13^C NMR (101 MHz, CDCl_3_ + CD_3_OD) δ 169.98, 165.53, 159.24, 157.73, 155.80, 143.54, 143.16, 137.62, 132.77, 132.65, 131.74, 131.68, 123.04, 113.28, 96.06, 64.82, 59.83, 59.05, 55.52, 26.23. HRMS (ESI) *m*/*z*: calcd for C_24_H_24_N_4_O_6_S, [M + H]^+^: 497.1551; found: 497.1567.
*Methyl 2-((2-(methoxycarbonyl)phenyl)diazenyl)-7-methyl-3-oxo-5-phenyl-2,3-dihydro-5H-thiazolo[3,2-a]pyrimidine-6-carboxylate* (**10d**). Yellow solid, yield: 65%, mp: 184–186 °C; ^1^H NMR (400 MHz, CDCl_3_) δ 11.35 (s, 1H), 7.95 (d, *J* = 6.5 Hz, 1H), 7.76 (d, *J* = 8.6 Hz, 1H), 7.50 (t, *J* = 7.1 Hz, 1H), 7.44–7.38 (m, 2H), 7.33–7.27 (m, 3H), 7.00 (t, *J* = 7.9 Hz, 1H), 6.24 (s, 1H), 3.94 (s, 3H), 3.66 (s, 3H), 2.53 (s, 3H). ^13^C NMR (101 MHz, CDCl_3_) δ 168.77, 165.79, 160.71, 152.63, 152.34, 145.05, 139.43, 134.95, 130.80, 128.86, 128.79, 127.99, 125.98, 121.59, 115.00, 112.12, 109.92, 55.27, 52.51, 51.65, 22.75. HRMS (ESI) *m*/*z*: calcd for C_23_H_20_N_4_O_5_S, [M + H]^+^: 465.1254; found: 465.1274.
*5-Methoxy-2-(2-(6-(methoxycarbonyl)-7-methyl-3-oxo-5-phenyl-5H-thiazolo[3,2-a]pyrimidin-2(3H)-ylidene)hydrazineyl)benzenesulfonic acid* (**10e**). Yellow solid, yield: 86%, mp: 247–249 °C; ^1^H NMR (400 MHz, DMSO-*d*_6_) δ 10.89 (s, 1H), 7.38–7.25 (m, 6H), 6.91 (d, *J* = 8.8 Hz, 1H), 6.86 (d, *J* = 2.5 Hz, 1H), 6.01 (s, 1H), 3.70 (s, 3H), 3.60 (s, 3H), 2.38 (s, 3H). ^13^C NMR (101 MHz, DMSO-*d*_6_) δ 165.88, 160.45, 154.77, 153.42, 151.61, 140.83, 133.90, 132.26, 129.29, 129.04, 127.65, 122.12, 117.29, 115.59, 112.36, 109.68, 55.90, 55.05, 52.01, 22.87. HRMS (ESI) *m*/*z*: calcd for C_22_H_20_N_4_O_7_S_2_, [M + H]^+^: 517.0854; found: 517.0848.
*(6-(Methoxycarbonyl)-7-methyl-3-oxo-5-phenyl-5H-thiazolo[3,2-a]pyrimidin-2(3H)-ylidene)hydrazineyl)naphthalene-1-sulfonic acid* (**10f**). Yellow solid, yield: 80%, mp: 289–293 °C; ^1^H NMR (400 MHz, DMSO-*d*_6_) δ 12.84 (s, 1H), 8.83 (d, *J* = 9.1 Hz, 1H), 7.91 (d, *J* = 9.2 Hz, 1H), 7.80 (d, *J* = 7.6 Hz, 1H), 7.71 (d, *J* = 9.1 Hz, 1H), 7.51–7.45 (m, 1H), 7.41–7.28 (m, 6H), 6.07 (s, 1H), 3.62 (s, 3H), 2.40 (s, 3H). ^13^C NMR (101 MHz, DMSO-*d*_6_) δ 165.87, 160.55, 153.48, 151.54, 146.57, 142.85, 140.77, 136.52, 131.87, 130.90, 130.18, 129.30, 129.06, 128.44, 127.71, 127.60, 127.11, 124.75, 124.56, 123.61, 114.57, 109.77, 55.11, 52.03, 22.87. HRMS (ESI) *m*/*z*: calcd for C_25_H_20_N_4_O_6_S_2_, [M − H]^−^: 535.0744; found: 535.0749.
*Methyl 2-(2-(2-acetylphenyl)hydrazineylidene)-7-methyl-3-oxo-5-phenyl-2,3-dihydro-5H-thiazolo[3,2-a]pyrimidine-6-carboxylate* (**10g**). Yellow solid, yield: 78%, mp: 199–202 °C; ^1^H NMR (400 MHz, CDCl_3_) δ 12.07 (s, 1H), 7.73 (t, *J* = 7.4 Hz, 2H), 7.43 (t, *J* = 7.1 Hz, 1H), 7.34 (d, *J* = 6.5 Hz, 2H), 7.26–7.17 (m, 3H), 6.94 (t, *J* = 7.6 Hz, 1H), 6.15 (s, 1H), 3.59 (s, 3H), 2.58 (s, 3H), 2.46 (s, 3H). ^13^C NMR (101 MHz, CDCl_3_) δ 202.76, 165.80, 160.71, 152.67, 152.32, 145.11, 139.38, 135.46, 131.71, 128.89, 128.81, 128.01, 126.48, 121.37, 118.97, 115.25, 109.95, 55.28, 51.70, 27.99, 22.74. HRMS (ESI) *m*/*z*: calcd for C_23_H_20_N_4_O_4_S, [M − H]^−^: 447.1125; found: 447.1126.
*Methyl 2-(2-(3,4-dimethoxyphenyl)hydrazineylidene)-7-methyl-3-oxo-5-phenyl-2,3-dihydro-5H-thiazolo[3,2-a]pyrimidine-6-carboxylate* (**10h**). Yellow solid, yield: 73%, mp: 204–206 °C; ^1^H NMR (400 MHz, CDCl_3_) δ 8.44 (s, 1H), 7.32 (d, *J* = 6.6 Hz, 2H), 7.26–7.17 (m, 3H), 6.79 (d, *J* = 2.5 Hz, 1H), 6.65 (d, *J* = 8.6 Hz, 1H), 6.55 (dd, *J* = 8.6, 2.5 Hz, 1H), 6.13 (s, 1H), 3.74 (s, 3H), 3.70 (s, 3H), 3.58 (s, 3H), 2.44 (s, 3H). ^13^C NMR (101 MHz, CDCl_3_) δ 166.04, 159.82, 150.05, 149.95, 145.88, 145.78, 139.95, 136.04, 128.87, 128.84, 127.99, 127.82, 121.04, 111.74, 111.65, 109.44, 108.11, 106.59, 105.80, 99.27, 98.11, 55.99, 55.37, 54.87, 51.79, 22.91. HRMS (ESI) *m*/*z*: calcd for C_23_H_22_N_4_O_5_S, [M + H]^+^: 467.1430; found: 467.1438.
*Methyl 2-(2-(2-methoxyphenyl)hydrazineylidene)-7-methyl-3-oxo-5-phenyl-2,3-dihydro-5H-thiazolo[3,2-a]pyrimidine-6-carboxylate* (**10i**). Yellow solid, yield: 55%, mp: 189–192 °C; ^1^H NMR (400 MHz, DMSO-*d*_6_) δ 10.35 (s, 1H), 7.39–7.24 (m, 6H), 7.03 (q, *J* = 7.9 Hz, 2H), 6.93 (t, *J* = 6.9 Hz, 1H), 6.02 (s, 1H), 3.86 (s, 3H), 3.60 (s, 3H), 2.38 (s, 3H). ^13^C NMR (101 MHz, CDCl_3_) δ 165.73, 160.61, 153.08, 152.02, 146.28, 139.44, 130.96, 128.88, 128.85, 128.79, 127.99, 127.79, 123.35, 121.62, 114.51, 110.40, 109.74, 55.76, 55.23, 51.66, 22.53. HRMS (ESI) *m*/*z*: calcd for C_22_H_20_N_4_O_4_S, [M + H]^+^: 437.1321; found: 437.1332.
*Methyl 7-methyl-3-oxo-5-phenyl-2-(2-phenylhydrazineylidene)-2,3-dihydro-5H-thiazolo[3,2-a]pyrimidine-6-carboxylate* (**10j**). Yellow solid, yield: 49%, mp: 124–127 °C; ^1^H NMR (400 MHz, DMSO-*d*_6_) δ 10.99 (s, 1H), 7.38–7.29 (m, 7H), 7.23 (d, *J* = 7.4 Hz, 2H), 6.99 (t, *J* = 7.3 Hz, 1H), 6.04 (s, 1H), 3.61 (s, 3H), 2.39 (s, 3H). ^13^C NMR (101 MHz, CDCl_3_) δ 165.80, 160.84, 152.95, 152.17, 141.94, 139.43, 129.44, 128.89, 128.86, 128.83, 127.97, 123.56, 122.08, 114.62, 109.82, 55.27, 51.70, 22.67. HRMS (ESI) *m*/*z*: calcd for C_21_H_18_N_4_O_3_S, [M − H]^−^: 405.1020; found: 405.1022.
*(2-(6-(Methoxycarbonyl)-7-methyl-3-oxo-5-phenyl-5H-thiazolo[3,2-a]pyrimidin-2(3H)-ylidene)hydrazineyl)benzenesulfonic acid* (**10k**). Yellow solid, yield: 84%, mp: 262–266 °C; ^1^H NMR (400 MHz, DMSO-*d*_6_) δ 11.24 (s, 1H), 7.99–7.82 (m, 2H), 7.57 (d, *J* = 7.3 Hz, 1H), 7.37–7.29 (m, 6H), 6.05 (s, 1H), 3.62 (s, 3H), 2.39 (s, 3H). ^13^C NMR (101 MHz, DMSO-*d*_6_) δ 165.85, 160.46, 153.24, 151.42, 140.71, 138.56, 132.72, 131.07, 129.30, 129.07, 128.00, 127.69, 123.86, 122.28, 113.90, 109.90, 55.14, 52.04, 22.85. HRMS (ESI) *m*/*z*: calcd for C_21_H_18_N_4_O_6_S_2_, [M − H]^−^: 485.0588; found: 485.0593.
*Methyl 7-methyl-2-(2-(3-nitrophenyl)hydrazineylidene)-3-oxo-5-phenyl-2,3-dihydro-5H-thiazolo[3,2-a]pyrimidine-6-carboxylate* (**10l**). Yellow solid, yield: 71%, mp: 250–252 °C; ^1^H NMR (400 MHz, CDCl_3_) δ 8.00 (s, 1H), 7.88 (d, *J* = 8.0 Hz, 1H), 7.67 (s, 1H), 7.57 (d, *J* = 9.5 Hz, 1H), 7.47 (t, *J* = 8.2 Hz, 1H), 7.41 (d, *J* = 6.4 Hz, 2H), 7.31 (q, *J* = 8.5, 7.5 Hz, 3H), 6.24 (s, 1H), 3.67 (s, 3H), 2.54 (s, 3H). ^13^C NMR (101 MHz, CDCl_3_ + CD_3_OD) δ 165.78, 161.05, 154.73, 148.99, 144.07, 139.16, 130.11, 128.94, 128.77, 127.84, 120.16, 117.17, 109.71, 109.31, 55.16, 51.69, 22.22. HRMS (ESI) *m*/*z*: calcd for C_21_H_17_N_5_O_5_S, [M − H]^−^: 450.0870; found: 450.0879.
*Methyl 2-(2-(3-acetylphenyl)hydrazineylidene)-7-methyl-3-oxo-5-phenyl-2,3-dihydro-5H-thiazolo[3,2-a]pyrimidine-6-carboxylate* (**10m**). Yellow solid, yield: 45%, mp: 213–216 °C; ^1^H NMR (400 MHz, DMSO-*d*_6_) δ 11.12 (s, 1H), 7.76 (s, 1H), 7.59 (d, *J* = 3.1 Hz, 1H), 7.51–7.41 (m, 2H), 7.40–7.25 (m, 5H), 6.01 (s, 1H), 3.61 (s, 3H), 2.56 (s, 3H), 2.38 (s, 3H). ^13^C NMR (101 MHz, CDCl_3_) δ 203.14, 169.95, 165.43, 158.98, 155.78, 147.26, 143.38, 141.80, 133.57, 132.81, 132.77, 132.68, 131.85, 131.77, 126.77, 124.72, 123.21, 118.11, 113.44, 59.05, 55.56, 30.51, 26.25. HRMS (ESI) *m*/*z*: calcd for C_23_H_20_N_4_O_4_S, [M + H]^+^: 449.1295; found: 449.1286.
*Methyl 2-(2-(3-(methoxycarbonyl)phenyl)hydrazineylidene)-7-methyl-3-oxo-5-phenyl-2,3-dihydro-5H-thiazolo[3,2-a]pyrimidine-6-carboxylate* (**10n**). Yellow solid, yield: 42%, mp: 214–216 °C; ^1^H NMR (400 MHz, DMSO-*d*_6_) δ 11.14 (s, 1H), 7.92–7.17 (m, 9H), 6.03 (s, 1H), 3.86 (s, 3H), 3.61 (s, 3H), 2.39 (s, 3H). ^13^C NMR (101 MHz, CDCl_3_ + CD_3_OD) δ 167.07, 165.87, 161.21, 154.60, 151.85, 142.92, 139.45, 130.97, 129.46, 128.86, 128.76, 127.88, 123.97, 121.17, 118.97, 118.92, 115.50, 115.45, 55.11, 52.30, 51.68, 22.45. HRMS (ESI) *m*/*z*: calcd for C_23_H_20_N_4_O_5_S, [M + H]^+^: 465.1254; found: 465.1252.
*Methyl 2-(2-(2-acetylphenyl)hydrazineylidene)-5-(4-methoxyphenyl)-7-methyl-3-oxo-2,3-dihydro-5H-thiazolo[3,2-a]pyrimidine-6-carboxylate* (**12a**). Yellow solid, yield: 64%, mp: 199–201 °C; ^1^H NMR (400 MHz, CDCl_3_) δ 12.07 (s, 1H), 7.74 (t, *J* = 8.5 Hz, 2H), 7.43 (t, *J* = 8.6 Hz, 1H), 7.26 (d, *J* = 8.8 Hz, 2H), 7.02–6.89 (m, 1H), 6.75 (d, *J* = 8.7 Hz, 2H), 6.11 (s, 1H), 3.68 (s, 3H), 3.59 (s, 3H), 2.59 (s, 3H), 2.46 (s, 3H). ^13^C NMR (101 MHz, CDCl_3_) δ 202.71, 165.88, 160.81, 159.88, 152.48, 152.03, 145.15, 135.44, 131.70, 131.66, 129.41, 126.67, 121.33, 118.98, 115.28, 114.08, 110.10, 55.27, 54.74, 51.69, 27.98, 22.69. HRMS (ESI) *m*/*z*: calcd for C_24_H_22_N_4_O_5_S, [M − H]^−^: 477.1231; found: 477.1236.
*Methyl 2-(2-(2-(methoxycarbonyl)phenyl)hydrazineylidene)-5-(4-methoxyphenyl)-7-methyl-3-oxo-2,3-dihydro-5H-thiazolo[3,2-a]pyrimidine-6-carboxylate* (**12b**). Yellow solid, yield: 75%, mp: 219–221 °C; ^1^H NMR (400 MHz, CDCl_3_) δ 11.32 (s, 1H), 7.94 (d, *J* = 6.4 Hz, 1H), 7.76 (d, *J* = 7.3 Hz, 1H), 7.49 (t, *J* = 7.4 Hz, 1H), 7.34 (d, *J* = 8.8 Hz, 2H), 6.99 (t, *J* = 8.1 Hz, 1H), 6.82 (d, *J* = 8.7 Hz, 2H), 6.19 (s, 1H), 3.94 (s, 3H), 3.76 (s, 3H), 3.66 (s, 3H), 2.52 (s, 3H). ^13^C NMR (101 MHz, CDCl_3_) δ 168.79, 165.90, 160.87, 159.86, 152.47, 152.10, 145.07, 134.97, 131.68, 130.81, 129.41, 126.12, 121.58, 115.02, 114.08, 112.12, 110.05, 55.28, 54.72, 52.53, 51.69, 22.72. HRMS (ESI) *m*/*z*: calcd for C_24_H_22_N_4_O_6_S, [M + H]^+^: 495.1348; found: 495.1339.
*Methyl 2-(2-(3-(methoxycarbonyl)phenyl)hydrazineylidene)-5-(4-methoxyphenyl)-7-methyl-3-oxo-2,3-dihydro-5H-thiazolo[3,2-a]pyrimidine-6-carboxylate* (**12c**). Yellow solid, yield: 80%, mp: 209–211 °C; ^1^H NMR (400 MHz, CDCl_3_) δ 7.78 (s, 1H), 7.59 (d, *J* = 7.7 Hz, 1H), 7.48 (d, *J* = 7.7 Hz, 1H), 7.30 (dt, *J* = 10.7, 3.3 Hz, 2H), 7.24 (s, 1H), 6.77 (d, *J* = 8.7 Hz, 2H), 6.09 (s, 1H), 3.84 (s, 3H), 3.70 (s, 3H), 3.61 (s, 3H), 2.44 (s, 3H). ^13^C NMR (101 MHz, CDCl_3_ + CD_3_OD) δ 167.17, 165.99, 161.34, 159.78, 154.75, 151.54, 143.05, 131.74, 130.94, 129.42, 129.24, 123.90, 120.95, 118.95, 115.46, 114.00, 109.59, 55.16, 54.50, 52.21, 51.62, 22.29. HRMS (ESI) *m*/*z*: calcd for C_24_H_22_N_4_O_6_S, [M + H]^+^: 495.1348; found: 495.1341.
*(2-(6-(Methoxycarbonyl)-5-(4-methoxyphenyl)-7-methyl-3-oxo-5H-thiazolo[3,2-a]pyrimidin-2(3H)-ylidene)hydrazineyl)-3-methylbenzoic acid* (**12d**). Yellow solid, yield: 77%, mp: 265–267 °C; ^1^H NMR (400 MHz, DMSO-*d*_6_) δ 7.82 (d, *J* = 7.7 Hz, 1H), 7.22 (d, *J* = 8.7 Hz, 2H), 7.17 (d, *J* = 7.4 Hz, 1H), 6.90 (d, *J* = 8.8 Hz, 2H), 6.85 (d, *J* = 7.5 Hz, 1H), 5.98 (s, 1H), 3.72 (s, 3H), 3.60 (s, 3H), 2.43 (s, 3H), 2.38 (s, 3H). ^13^C NMR (101 MHz, DMSO-*d*_6_) δ 166.07, 160.56, 159.66, 154.97, 152.20, 145.25, 135.40, 133.40, 129.80, 129.10, 126.60, 123.18, 121.74, 119.44, 114.49, 108.80, 55.55, 54.04, 51.90, 23.00, 22.11. HRMS (ESI) *m*/*z*: calcd for C_24_H_22_N_4_O_6_S, [M + H]^+^: 495.1348; found: 495.1344.
*Methyl 5-(4-methoxyphenyl)-7-methyl-3-oxo-2-(2-(3,4,5-trimethoxyphenyl)hydrazineylidene)-2,3-dihydro-5H-thiazolo[3,2-a]pyrimidine-6-carboxylate* (**12e**). Yellow solid, yield: 65%, mp: 204–206 °C; ^1^H NMR (400 MHz, DMSO-*d*_6_) δ 10.88 (s, 1H), 7.22 (d, *J* = 8.7 Hz, 2H), 6.90 (d, *J* = 8.8 Hz, 2H), 6.54 (s, 2H), 5.97 (s, 1H), 3.76 (s, 6H), 3.72 (s, 3H), 3.61 (s, 3H), 3.60 (s, 3H), 2.39 (s, 3H). ^13^C NMR (101 MHz, DMSO-*d*_6_) δ 165.93, 160.85, 159.75, 154.21, 154.01, 151.69, 139.69, 133.50, 132.94, 129.21, 120.34, 114.50, 109.39, 92.19, 60.61, 56.13, 55.57, 55.36, 54.36, 51.95, 22.86. HRMS (ESI) *m*/*z*: calcd for C_25_H_26_N_4_O_7_S, [M + H]^+^: 527.1619; found: 527.1632.
*(6-(Methoxycarbonyl)-5-(4-methoxyphenyl)-7-methyl-3-oxo-5H-thiazolo[3,2-a]pyrimidin-2(3H)-ylidene)hydrazineyl)naphthalene-1-sulfonic acid* (**12f**). Yellow solid, yield: 90%, mp: 271–273 °C; ^1^H NMR (400 MHz, DMSO-*d*_6_) δ 12.81 (s, 1H), 8.84 (d, *J* = 7.7 Hz, 1H), 7.92 (d, *J* = 9.1 Hz, 1H), 7.80 (d, *J* = 6.7 Hz, 1H), 7.73 (d, *J* = 9.1 Hz, 1H), 7.48 (t, *J* = 7.0 Hz, 1H), 7.37 (t, *J* = 6.8 Hz, 1H), 7.24 (d, *J* = 8.7 Hz, 2H), 6.91 (d, *J* = 8.8 Hz, 2H), 6.02 (s, 1H), 3.72 (s, 3H), 3.62 (s, 3H), 2.41 (s, 3H). ^13^C NMR (101 MHz, DMSO-*d*_6_) δ 165.93, 160.60, 159.78, 153.19, 151.35, 136.56, 132.82, 131.90, 130.90, 130.17, 129.16, 128.45, 127.57, 127.13, 124.56, 123.76, 114.58, 109.92, 55.58, 54.53, 52.03, 22.83. HRMS (ESI) *m*/*z*: calcd for C_26_H_22_N_4_O_7_S_2_, [M + H]^+^: 567.1016; found: 567.1003.

#### 4.1.5. General Procedure for the Synthesis of Compounds **11a–k**

The methyl 7-methyl-3-oxo-5-(2,3,4-trimethoxyphenyl)-2,3-dihydro-5*H*-thiazolo[3,2-*a*]pyrimidine-6-carboxylate (1 mmol, 1 eq) and pyridine (4 mmol, 4 eq) were dissolved in 5 mL of dioxane at 0 °C. An appropriate aromatic amine (1.2 mmol, 1.2 eq) was dissolved in an aqueous solution of hydrochloric acid (3 mmol, 3 eq) and stirred at 0 °C. Then, a solution of sodium nitrite (1.44 mmol, 1.44 eq) was added to the hydrochloric acid–aromatic amine mixture to obtain the aromatic diazonium salt by stirring the above aqueous solution for 0.5 h at 0 °C. The resulting solution was added to the dioxane system and stirred at a temperature of -18 °C for 1 h, before raising it to room temperature and stirring for 3 h. The reaction was quenched by adding 10 mL of water, which resulted in precipitation of a solid product that was subsequently purified using silica gel column chromatography.

*Methyl 2-(2-(2-methoxyphenyl)hydrazineylidene)-7-methyl-3-oxo-5-(2,3,4-trimethoxyphenyl)-2,3-dihydro-5H-thiazolo[3,2-a]pyrimidine-6-carboxylate* (**11a**). Yellow solid, yield: 61%, mp: 178–180 °C; ^1^H NMR (400 MHz, CDCl_3_) δ 7.89 (s, 1H), 7.49 (dd, *J* = 7.7, 1.9 Hz, 1H), 7.08 (d, *J* = 8.7 Hz, 1H), 7.00–6.89 (m, 2H), 6.85 (dd, *J* = 7.8, 1.6 Hz, 1H), 6.58 (d, *J* = 8.7 Hz, 1H), 6.22 (s, 1H), 3.90 (s, 6H), 3.81 (s, 3H), 3.78 (s, 3H), 3.67 (s, 3H), 2.43 (s, 3H). ^13^C NMR (101 MHz, CDCl_3_) δ 166.24, 160.68, 154.31, 152.64, 146.17, 142.02, 131.14, 125.57, 124.93, 123.04, 121.58, 114.36, 110.32, 109.46, 106.41, 60.68, 60.56, 55.91, 55.75, 53.54, 51.45, 22.79. HRMS (ESI) *m*/*z*: calcd for C_25_H_26_N_4_O_7_S, [M + H]^+^: 527.1619; found: 527.1634.
*Methyl 7-methyl-3-oxo-2-(2-phenylhydrazineylidene)-5-(2,3,4-trimethoxyphenyl)-2,3-dihydro-5H-thiazolo [3,2-a]pyrimidine-6-carboxylate* (**11b**). Yellow solid, yield: 46%, mp: 189–192 °C; ^1^H NMR (400 MHz, CDCl_3_) δ 7.49 (s, 1H), 7.33 (t, *J* = 7.8 Hz, 2H), 7.22 (d, *J* = 7.6 Hz, 2H), 7.11 (d, *J* = 8.6 Hz, 1H), 7.05 (t, *J* = 7.3 Hz, 1H), 6.61 (d, *J* = 8.7 Hz, 1H), 6.25 (s, 1H), 3.92 (s, 3H), 3.85 (s, 3H), 3.82 (s, 3H), 3.70 (s, 3H), 2.46 (s, 3H). ^13^C NMR (101 MHz, CDCl_3_ + CD_3_OD) δ 166.53, 161.55, 155.53, 154.35, 152.60, 150.25, 142.88, 141.98, 129.27, 125.73, 125.00, 123.04, 119.80, 114.63, 109.05, 106.56, 60.61, 60.55, 55.90, 53.47, 51.48, 22.45. HRMS (ESI) *m*/*z*: calcd for C_24_H_24_N_4_O_6_S, [M + H]^+^: 497.1509; found: 497.1519.
*(2-(6-(Methoxycarbonyl)-7-methyl-3-oxo-5-(2,3,4-trimethoxyphenyl)-5H-thiazolo[3,2-a]pyrimidin-2(3H)-ylidene)hydrazineyl)-3-methylbenzoic acid* (**11c**). Yellow solid, yield: 30%, mp: 209–213 °C; ^1^H NMR (400 MHz, DMSO-*d*_6_) δ 7.81 (s, 1H), 7.15 (d, *J* = 7.3 Hz, 1H), 6.96 (d, *J* = 8.6 Hz, 1H), 6.84 (t, *J* = 7.4 Hz, 1H), 6.75 (d, *J* = 8.7 Hz, 1H), 6.06 (s, 1H), 3.78 (s, 6H), 3.71 (s, 3H), 3.58 (s, 3H), 2.41 (s, 3H), 2.30 (s, 3H). ^13^C NMR (101 MHz, DMSO-*d*_6_) δ 166.36, 160.48, 154.96, 154.12, 152.26, 150.82, 145.44, 141.92, 135.32, 129.72, 126.58, 126.26, 125.23, 123.13, 121.53, 119.69, 108.38, 107.56, 61.00, 60.62, 56.24, 52.70, 51.62, 23.04, 22.06. HRMS (ESI) *m*/*z*: calcd for C_26_H_26_N_4_O_8_S, [M + H]^+^: 554.1570; found: 555.1573.
*Methyl 7-methyl-2-(2-(3-nitrophenyl)hydrazineylidene)-3-oxo-5-(2,3,4-trimethoxyphenyl)-2,3-dihydro-5H-thiazolo[3,2-a]pyrimidine-6-carboxylate* (**11d**). Yellow solid, yield: 48%, mp: 236–238 °C; ^1^H NMR (400 MHz, CDCl_3_) δ 8.20 (s, 1H), 7.97 (t, *J* = 2.2 Hz, 1H), 7.82 (d, *J* = 6.8 Hz, 1H), 7.50 (d, *J* = 9.4 Hz, 1H), 7.41 (t, *J* = 8.1 Hz, 1H), 7.08 (d, *J* = 8.6 Hz, 1H), 6.58 (d, *J* = 8.7 Hz, 1H), 6.21 (s, 1H), 3.88 (s, 3H), 3.82 (s, 3H), 3.79 (s, 3H), 3.68 (s, 3H), 2.43 (s, 3H). ^13^C NMR (101 MHz, CDCl_3_) δ 166.05, 160.48, 154.50, 152.60, 149.70, 149.01, 143.49, 142.01, 130.29, 125.72, 124.27, 120.09, 117.56, 109.84, 109.35, 106.46, 60.72, 60.60, 55.91, 54.00, 51.62, 22.59. HRMS (ESI) *m*/*z*: calcd for C_24_H_23_N_5_O_8_S, [M + H]^+^: 541.1357; found: 542.1361.
*2-(2-(6-(Methoxycarbonyl)-7-methyl-3-oxo-5-(2,3,4-trimethoxyphenyl)-5H-thiazolo[3,2-a]pyrimidin-2(3H)-ylidene)hydrazineyl)benzenesulfonic acid* (**11e**). Yellow solid, yield: 74%, mp: 238–240 °C; ^1^H NMR (400 MHz, DMSO-*d*_6_) δ 11.15 (s, 1H), 7.59 (d, *J* = 7.7 Hz, 1H), 7.35 (d, *J* = 7.7 Hz, 2H), 6.99 (d, *J* = 8.6 Hz, 2H), 6.76 (d, *J* = 8.7 Hz, 1H), 6.07 (s, 1H), 3.78 (s, 6H), 3.70 (s, 3H), 3.60 (s, 3H), 2.31 (s, 3H). ^13^C NMR (101 MHz, DMSO-*d*_6_) δ 166.17, 160.32, 154.31, 152.88, 152.37, 149.94, 141.89, 138.67, 132.55, 131.04, 127.98, 125.50, 125.35, 124.20, 122.06, 113.76, 109.42, 107.44, 61.02, 60.64, 56.24, 53.38, 51.82, 22.86. HRMS (ESI) *m*/*z*: calcd for C_24_H_24_N_4_O_9_S_2_, [M + H]^+^: 577.1067; found: 577.1062.
*5-Methoxy-2-(2-(6-(methoxycarbonyl)-7-methyl-3-oxo-5-(2,3,4-trimethoxyphenyl)-5H-thiazolo[3,2-a]pyrimidin-2(3H)-ylidene)hydrazineyl)benzenesulfonic acid* (**11f**). Yellow solid, yield: 76%, mp: 244–246 °C; ^1^H NMR (400 MHz, DMSO-*d*_6_) δ 10.93 (s, 1H), 7.31 (d, *J* = 8.9 Hz, 1H), 7.13 (s, 1H), 6.98 (d, *J* = 8.6 Hz, 2H), 6.75 (d, *J* = 8.6 Hz, 1H), 6.06 (s, 1H), 3.77 (s, 6H), 3.73 (s, 3H), 3.70 (s, 3H), 3.59 (s, 3H), 2.31 (s, 3H). ^13^C NMR (101 MHz, DMSO-*d*_6_) δ 166.19, 160.34, 154.58, 154.28, 153.06, 152.36, 150.10, 141.87, 133.57, 132.38, 125.47, 122.52, 117.31, 115.45, 112.32, 109.23, 107.43, 61.02, 60.63, 56.22, 55.88, 53.30, 51.79, 22.87. HRMS (ESI) *m*/*z*: calcd for C_25_H_26_N_4_O_10_S_2_, [M − H]^−^: 605.1010; found: 605.1006.
*(2-(6-(Methoxycarbonyl)-7-methyl-3-oxo-5-(2,3,4-trimethoxyphenyl)-5H-thiazolo[3,2-a]pyrimidin-2(3H)-ylidene)hydrazineyl)naphthalene-1-sulfonic acid* (**11g**). Yellow solid, yield: 76%, mp: 241–244 °C; ^1^H NMR (400 MHz, DMSO-*d*_6_) δ 12.74 (s, 1H), 8.85 (d, *J* = 8.9 Hz, 1H), 7.90 (d, *J* = 9.0 Hz, 1H), 7.79 (d, *J* = 8.1 Hz, 1H), 7.71 (d, *J* = 9.0 Hz, 1H), 7.48 (t, *J* = 7.8 Hz, 1H), 7.36 (t, *J* = 7.5 Hz, 1H), 7.00 (d, *J* = 8.7 Hz, 1H), 6.77 (d, *J* = 8.6 Hz, 1H), 6.09 (s, 1H), 3.78 (d, *J* = 4.4 Hz, 6H), 3.70 (s, 3H), 3.60 (s, 3H), 2.32 (s, 3H). ^13^C NMR (101 MHz, DMSO-*d*_6_) δ 166.20, 160.41, 154.32, 153.09, 152.36, 150.03, 141.95, 136.69, 131.82, 130.95, 130.10, 128.42, 127.57, 127.08, 125.49, 125.43, 124.55, 124.46, 123.96, 114.59, 109.41, 107.54, 61.04, 60.64, 56.28, 53.29, 51.77, 22.86. HRMS (ESI) *m*/*z*: calcd for C_28_H_26_N_4_O_9_S_2_, [M − H]^−^: 625.1061; found: 625.1056.
*Methyl 2-(2-(2-(methoxycarbonyl)phenyl)hydrazineylidene)-7-methyl-3-oxo-5-(2,3,4-trimethoxyphenyl)-2,3-dihydro-5H-thiazolo[3,2-a]pyrimidine-6-carboxylate* (**11h**). Yellow solid, yield: 55%, mp: 200–202 °C; ^1^H NMR (400 MHz, CDCl_3_) δ 11.29 (s, 1H), 7.95 (d, *J* = 6.4 Hz, 1H), 7.74 (d, *J* = 8.4 Hz, 1H), 7.48 (t, *J* = 7.1 Hz, 1H), 7.08 (d, *J* = 8.6 Hz, 1H), 7.01–6.94 (m, 1H), 6.58 (d, *J* = 8.6 Hz, 1H), 6.23 (s, 1H), 3.95 (s, 3H), 3.90 (s, 3H), 3.82 (s, 3H), 3.79 (s, 3H), 3.67 (s, 3H), 2.43 (s, 3H). ^13^C NMR (101 MHz, CDCl_3_) δ 168.42, 165.89, 160.36, 153.97, 152.29, 144.83, 141.67, 134.57, 130.44, 126.06, 125.20, 124.48, 121.03, 114.60, 111.66, 109.27, 106.03, 60.31, 60.21, 55.56, 53.22, 52.14, 51.10, 22.49. HRMS (ESI) *m*/*z*: calcd for C_26_H_26_N_4_O_8_S, [M + H]^+^: 554.1570; found: 555.1574.
*Methyl 2-(2-(2-acetylphenyl)hydrazineylidene)-7-methyl-3-oxo-5-(2,3,4-trimethoxyphenyl)-2,3-dihydro-5H-thiazolo[3,2-a]pyrimidine-6-carboxylate* (**11i**). Yellow solid, yield: 52%, mp: 205–208 °C; ^1^H NMR (400 MHz, CDCl_3_) δ 12.12 (s, 1H), 7.81 (dd, *J* = 15.9, 8.3 Hz, 2H), 7.49 (t, *J* = 7.2 Hz, 1H), 7.08 (d, *J* = 8.6 Hz, 1H), 7.01 (t, *J* = 7.0 Hz, 1H), 6.58 (d, *J* = 8.7 Hz, 1H), 6.23 (s, 1H), 3.90 (s, 3H), 3.82 (s, 3H), 3.78 (s, 3H), 3.67 (s, 3H), 2.67 (s, 3H), 2.44 (s, 3H). ^13^C NMR (101 MHz, CDCl_3_) δ 202.65, 166.25, 160.69, 154.31, 152.63, 152.54, 150.55, 145.27, 142.02, 135.40, 131.69, 127.02, 125.55, 124.83, 121.12, 118.88, 115.20, 109.68, 106.39, 60.68, 60.56, 55.91, 53.55, 51.46, 27.98, 22.88. HRMS (ESI) *m*/*z*: calcd for C_26_H_26_N_4_O_7_S, [M + H]^+^: 539.1609; found: 539.1605.
*Methyl 2-(2-(3-(methoxycarbonyl)phenyl)hydrazineylidene)-7-methyl-3-oxo-5-(2, 3, 4-trimethoxyphenyl)-2, 3-dihydro-5H-thiazolo[3,2-a]pyrimidine-6-carboxylate* (**11j**). Yellow solid, yield: 67%, mp: 213–215 °C; ^1^H NMR (400 MHz, CDCl_3_) δ 7.94 (s, 1H), 7.72 (d, *J* = 7.6 Hz, 1H), 7.60 (d, *J* = 8.3 Hz, 1H), 7.44 (d, *J* = 8.4 Hz, 1H), 7.16 (d, *J* = 8.7 Hz, 1H), 6.69 (d, *J* = 8.7 Hz, 1H), 6.27 (s, 1H), 3.97 (d, *J* = 5.4 Hz, 6H), 3.91 (s, 6H), 3.76 (s, 3H), 2.50 (s, 3H). ^13^C NMR (101 MHz, CDCl_3_ + CD_3_OD) δ 167.36, 166.49, 161.39, 155.14, 154.43, 152.66, 150.18, 143.33, 142.05, 131.10, 129.54, 125.80, 124.89, 123.91, 121.30, 119.05, 115.54, 109.27, 60.67, 60.62, 55.97, 53.65, 52.34, 51.56, 22.55. HRMS (ESI) *m*/*z*: calcd for C_26_H_26_N_4_O_8_S, [M + H]^+^: 661.2733; found: 555.1577.
*Methyl 2-(2-(3-acetylphenyl)hydrazineylidene)-7-methyl-3-oxo-5-(2,3,4-trimethoxyphenyl)-2,3-dihydro-5H-thiazolo[3,2-a]pyrimidine-6-carboxylate* (**11k**). Yellow solid, yield: 40%, mp: 216–219 °C; ^1^H NMR (400 MHz, DMSO-*d*_6_) δ 11.00 (s, 1H), 7.75 (s, 1H), 7.59 (dt, *J* = 6.4, 2.1 Hz, 1H), 7.51–7.40 (m, 2H), 6.97 (d, *J* = 8.7 Hz, 1H), 6.75 (d, *J* = 8.8 Hz, 1H), 6.04 (s, 1H), 3.78 (s, 6H), 3.70 (s, 3H), 3.59 (s, 3H), 2.56 (s, 3H), 2.31 (s, 3H). ^13^C NMR (101 MHz, DMSO-*d*_6_) δ 198.09, 166.19, 160.67, 154.25, 152.31, 141.86, 138.23, 130.31, 125.45, 122.89, 119.04, 109.04, 107.46, 61.05, 60.62, 56.24, 53.08, 51.78, 27.25, 22.90. HRMS (ESI) *m*/*z*: calcd for C_26_H_26_N_4_O_7_S, [M + H]^+^: 539.1609; found: 539.1604.

### 4.2. Other Protocols

Additional experimental methods are provided in the Supporting Information.

## Figures and Tables

**Table 1:** RNase H inhibition of the synthesized compounds **10a**–**n**, **11a**–**k**, and **12a**–**f**.

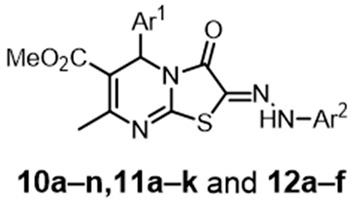			
Comp.	Ar^1^	Ar^2^	IC_50_ (μM) ^a^
**10a**	Ph	2-OH-Ph	18.23 ± 0.85
**10b**	Ph	2-Me-5-CO_2_H-Ph	44.86 ± 3.53
**10c**	Ph	3,4,5-triOMe-Ph	24.38 ± 1.25
**10d**	Ph	2-CO_2_CH_3_-Ph	8.27 ± 0.39
**10e**	Ph	2-SO_3_H-4-OMe-Ph	58.37 ± 1.71
**10f**	Ph	2-SO_3_H-Naph	7.04 ± 0.57
**10g**	Ph	2-COCH_3_-Ph	9.01 ± 2.17
**10h**	Ph	3,4-bis-OMe-Ph	14.42 ± 1.23
**10i**	Ph	2-OMe-Ph	8.32 ± 0.06
**10j**	Ph	Ph	21.45 ± 0.10
**10k**	Ph	2-SO_3_H-Ph	>100 (85%) ^b^
**10l**	Ph	3-NO_2_-Ph	6.41 ± 0.24
**10m**	Ph	3-COCH_3_-Ph	18.39 ± 0.79
**10n**	Ph	3-CO_2_CH_3_-Ph	7.03 ± 0.17
**11a**	2,3,4-3OMe-Ph	2-OMe-Ph	11.81 ± 0.91
**11b**	2,3,4-3OMe-Ph	Ph	48.95 ± 4.73
**11c**	2,3,4-3OMe-Ph	2-Me-5-CO_2_H-Ph	8.23 ± 2.44
**11d**	2,3,4-3OMe-Ph	3-NO_2_	10.10 ± 1.64
**11e**	2,3,4-3OMe-Ph	2-SO_3_H-Ph	>100 (97%)
**11f**	2,3,4-3OMe-Ph	2-SO_3_H-4-OMe-Ph	>100 (61%)
**11g**	2,3,4-3OMe-Ph	2-SO_3_H-Naph	19.95 ± 3.84
**11h**	2,3,4-3OMe-Ph	2-CO_2_CH_3_	10.05 ± 0.95
**11i**	2,3,4-3OMe-Ph	2-COCH_3_	>100 (100%)
**11j**	2,3,4-3OMe-Ph	3-CO_2_CH_3_-Ph	42.15 ± 9.78
**11k**	2,3,4-3OMe-Ph	3-COCH_3_-Ph	21.96 ± 0.36
**12a**	4-OMe-Ph	2-COCH_3_-Ph	3.98 ± 0.30
**12b**	4-OMe-Ph	2-CO_2_CH_3_-Ph	2.97 ± 0.88
**12c**	4-OMe-Ph	3-CO_2_CH_3_-Ph	4.71 ± 0.86
**12d**	4-OMe-Ph	2-Me-5-CO_2_H	17.97 ± 1.87
**12e**	4-OMe-Ph	3,4,5-tri-OMe-Ph	8.99 ± 0.27
**12f**	4-OMe-Ph	2-SO_3_H-Naph	3.28 ± 0.92
RDS1643 [24]			8.6 ± 1.30

^a^ Compound concentration required to reduce HIV-1 RT-associated RNase H activity by 50%. ^b^ Percentage of the control activity measured at the indicated compound concentration.

**Table 2:** Prediction of pharmacokinetics of representative compounds with good inhibitory activity.

Compd.	^a^ GI Absorption	^b^ BBB Permeant	^c^ P-gp Substrate	CYP1A2 Inhibitor	CYP2D6 Inhibitor	^d^ Lipinski	^e^ PAINS
**10d**	High	No	No	No	No	Yes; 0 violation	0 alert
**10f**	Low	No	No	No	No	Yes; 1 violation	0 alert
**12a**	High	No	No	No	No	Yes; 0 violation	0 alert
**12b**	Low	No	No	No	No	Yes; 0 violation	0 alert
**12e**	Low	No	No	No	No	No; 2 violations	0 alert
**12f**	Low	No	No	No	No	No; 2 violations	0 alert

^a^ GI absorption—according to the white of the BOILED-Egg. ^b^ BBB permeant—according to the yolk of the BOILED-Egg. ^c^ P-gp substrate, CYP1A2 inhibitor, and CYP2D6 inhibitor—from Swiss ADME. ^d^ Lipinski (Pfizer) filter—MW ≤ 500; MLOGP ≤ 4.15; N or O ≤ 10; NH or OH ≤ 5. ^e^ PAINS—Pan Assay Interference Structures.

## Data Availability

The data that support the findings of this study are available from the corresponding author, F.-E.C., upon reasonable request.

## Supplementary Materials

The following supporting information can be downloaded at: https://www.mdpi.com/article/10.3390/molecules29092120/s1, Figure S1: Ligand-RMSD comparison of three-time molecular dynamics simulation; Figure S2: Inhibitory profile of compounds on HIV-1 RNase H activity; References citation [26,27,28].
