# Supplementary material for: Structure-Based Design of Novel Thiazolone[3,2-a]pyrimidine Derivatives as Potent RNase H Inhibitors for HIV Therapy
Source: Molecules. 2024 May 3;29(9):2120. doi: 10.3390/molecules29092120 (PMC11085872; doi:10.3390/molecules29092120)
Supplement: Supplementary file 1 [file molecules-29-02120-s001.zip › molecules-2961823-supplementary.pdf]

Supporting Information

Structure-Based Design of Novel Thiazolone[3,2-*a*]pyrimidine Derivatives as Potent RNase H Inhibitors for HIV Therapy

Xuan-De Zhu [1], Angela Corona [2], Stefania Maloccu [2], Enzo Tramontano [2], Shuai Wang [1], Christophe Pannecouque [3], Erik De Clercq [3], Ge Meng [1,*] and Fen-Er Chen [1,*]

[1] Engineering Center of Catalysis and Synthesis for Chiral Molecules, Department of Chemistry, Fudan University, Shanghai 200433, China; 21210220029@m.fudan.edu.cn (X.-D.Z.); shuaiwang@fudan.edu.cn (S.W.)

[2] Department of Life and Environmental Sciences, Department of Applied Science Biosyst, University of Cagliari, 09042, Cagliari, Italy; angela.corona@unica.it (A.C.); stefania.maloccu@unica.it (S.M.); tramon@unica.it (E.T.)

[3] Rega Institute for Medical Research, KU Leuven, Herestraat 49, B-3000 Leuven, Belgium; christophe.pannecouque@kuleuven.be (C.P.); erik.declercq@kuleuven.be (E.D.C.)

* Correspondence: mgfudan@fudan.edu.cn (G.M.); rfchen@fudan.edu.cn (F.-E.C.)

Table of Contents

1. Molecular Modeling....................3
2. Molecular Dynamic Simulations....................3
3. Biology Experimental section....................4
3.1 Expression and purification of recombinant HIV-1 RTs. 4
3.2 RNase H Polymerase-Independent Cleavage Assay 5
4. Magnesium Complexation 9
5. ^{1}H-NMR, ^{13}C-NMR spectra and HRMS of compounds....................10

1. Molecular Modeling

The Schrödinger Maestro(version 13.7) was used to perform the ensemble docking calculations. The HIV-1 reverse transcriptase X-ray structure used for the experiment had the PDB code: 3QIP. The protein's 3D structure was prepared using the Protein Preparation Workflow. The protein preparation followed a standard protocol: (1) addition of missing hydrogen atoms to the X-ray structure, (2) removal of all crystallographic water, (3) assignment of ionizable state to each titrable groups by PropKa, and (4) energy minimization using OPLS4 force field to optimize all hydrogen-bonding networks. The docking study was performed using Glide-docking module. The small molecule, taken from the PDB structures, was rocked into its corresponding protein structure at the Standard Precision (SP) without the need for any constraints. The stability of the simulation was assessed by monitoring the RMSD with respect to the minimized starting structure. The molecular docking result was generated using PyMol (http://pymol.sourceforge.net/).

2. Molecular Dynamic Simulations

We obtained the RMSF (Root Mean Square Fluctuation) (Figure 5A, Figure 5B), RMSD (Root Mean Square Deviation) (Figure 5C), dynamic hydrogen bond count (Figure 5D) using the GROMACS software. The RMSF plots (Figure 5A-5B) for p66 and p51 further illustrated the structural fluctuation range of amino acid residues in both peptide chains upon the binding of compound **12b** to the RT enzyme. The dynamics simulation software GROMACS (version 2023.3) was used to investigating the dynamic equilibrium stability of protein-ligand docking. We generated PDB files for the protein, ligand and magnesium ion using the molecular docking structure information obtained from Maestro. In the system environment of Ubuntu 22.04, we selected AMBER14 as the molecular force field. We selected a 0.15M sodium chloride (NaCl) aqueous solution as the environmental system and balanced the charges. We equilibrated the system at a temperature of 310 K and maintained standard atmospheric pressure as the equilibrium pressure. Then we ran the dynamics simulation. After that, we analyzed the simulation trajectory and obtained the results of RMSD, RMSF and numbers of H-bond. The gmx_MMPBSA(version 1.5.0.3) was used to calculate the binding free energy change. In the term of General namelist variables, we set start frame at "1", the final frame at "5000", the interval as 20 the temperature at 310k.

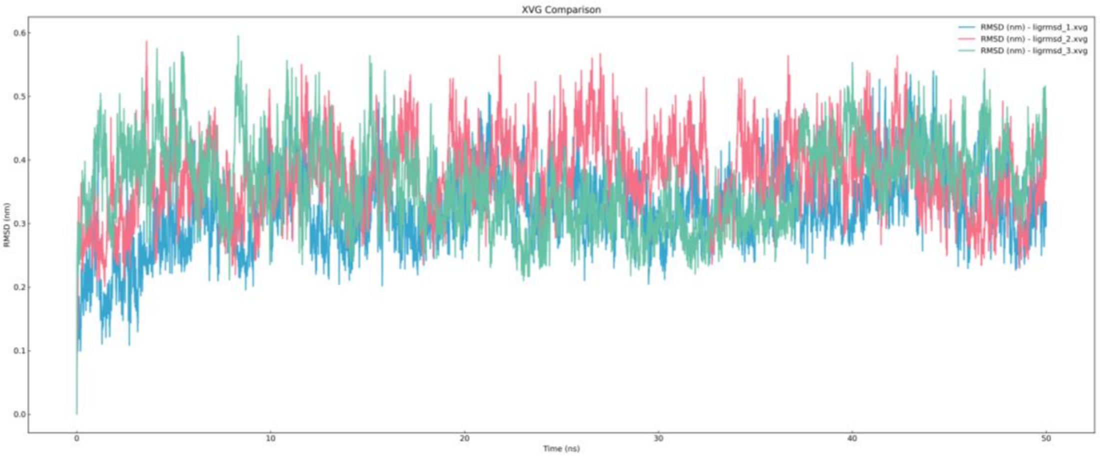

Figure S1. Ligand-RMSD comparison of three-time molecular dynamics simulation.

3. Biology Experimental section

3.1 Expression and purification of recombinant HIV-1 RTs.

His-tagged p66/p51 HIV-1 RT group M subtype B coded in a p6HRT-prot plasmid was expressed in the Escherichia coli strain M15.[1] Heterodimeric RTs were expressed essentially and purified as described.[2] E. coli cells harboring the expression vector were cultured until reaching an optical density at 600 nm of 0.7. Induction of protein expression was achieved by treating the cells with 1.7 mM isopropyl β-D-1-thiogalactopyranoside (IPTG) for a duration of 4 hours. The subsequent purification of the protein was performed using a BioLogic LP system (BioRad) employing a combination of immobilized metal affinity chromatography and ion exchange chromatography.

To initiate the purification process, the cell pellets were resuspended in lysis buffer at a ratio of 1:2 (cell pellet volume: lysis buffer volume). The lysis buffer used consisted of 50 mM sodium phosphate buffer with a pH of 7.8, supplemented with 0.5 mg/ml lysozyme. The resuspended cells were incubated on ice for 20 minutes. Following this, NaCl was added to achieve a final concentration of 0.3 M. The cells were then subjected to sonication, with four cycles of 60 seconds each, alternating between one second on and one second off. Subsequently, the cell lysate was centrifuged at 30, 000×g for 1 hour.

The supernatant obtained from the centrifugation step was loaded onto a Ni2+-NTA-Sepharose column that had been pre-equilibrated with a loading buffer consisting of 50 mM sodium phosphate buffer (pH 7.8), 0.3 M NaCl, 10% glycerol, and 10 mM imidazole. The column was washed with a buffer containing 50 mM sodium phosphate buffer (pH 6.0), 0.3 M NaCl, 10% glycerol, and 80 mM imidazole. The target protein, RT, was eluted using an imidazole gradient ranging from 0.08 M to 0.5 M in the wash buffer. Fractions containing the enzyme were collected and combined, and then diluted 1:1 with 50 mM sodium phosphate buffer (pH 7.0) containing 10% glycerol. The diluted protein sample was loaded onto a Hi-trap heparin HP column that had been pre-equilibrated with 10 column volumes of a loading buffer consisting of 50 mM sodium phosphate buffer (pH 7.0), 10% glycerol, and 150 mM NaCl. The column was washed with the loading buffer, and the RT enzyme was eluted using Elute Buffer 2, which

comprised 50 mM sodium phosphate buffer (pH 7.0), 10% glycerol, and 1 M NaCl. The eluted fractions containing the purified protein were collected, and the protein was subsequently dialyzed and stored in a buffer composed of 50 mM Tris-HCl (pH 7.0), 25 mM NaCl, 1 mM EDTA, and 50% glycerol. The catalytic activities of the purified protein samples were determined, and aliquots were stored at -80 °C for further use.

3.2 RNase H Polymerase-Independent Cleavage Assay

The HIV-1 RT-associated RNase H activity was measured as described.[3] In summary, the reaction was performed in a black 96-well plate with a total volume of 100 μL. The reaction mix consisted of 50 mM Tris HCl (pH 7.8), 6 mM $MgCl_2$, 1 mM dithiothreitol (DTT), 80 mM KCl, and 250 nM hybrid RNA/DNA substrate (50-GTTTTCTTTTCCCCCCTGAC-30-Fluorescein, 50-CAAAAGAAAAGGGGGGACUG-30-Dabcyl). Serial dilutions of compounds were added to the reaction mix. The reaction was initiated by adding 20 ng of HIV-1 wt RT, 20 ng of R448A RT, 20 ng of K451A RT, 40 ng of K540 RT, 60 ng of Q475A RT, 500 ng of N474A RT, 500 ng of Y501A RT, and 500 ng of W535A RT. The reaction mixture was then incubated at 37 °C for 1 hour. The products of the reaction were quantified using a Perkin-Elmer Victor 3 multilabel counter plate reader, with excitation and emission wavelengths set at 490/528 nm. The experiments were performed in duplicate and replicated at least two times to ensure reliability. The data obtained were analyzed according to the described methodology.[4] Dose response curves were fitted to a nonlinear regression of (log10) dose vs normalized response-variable slope. Assay quality was assessed using the Z′-factor calculation with $Z' > 0.5$ as the threshold for acceptance. Mean ± standard deviation of IC_{50} values were determined, and p values were calculated between the IC_{50} value against the wt and IC_{50} value against the mutants by paired, two-tailed *t* tests using GraphPad Prism 6.01 software (GraphPad Software, Inc.; San Diego, CA). Figures were made with GraphPad Prism 6 version 6.01.

A

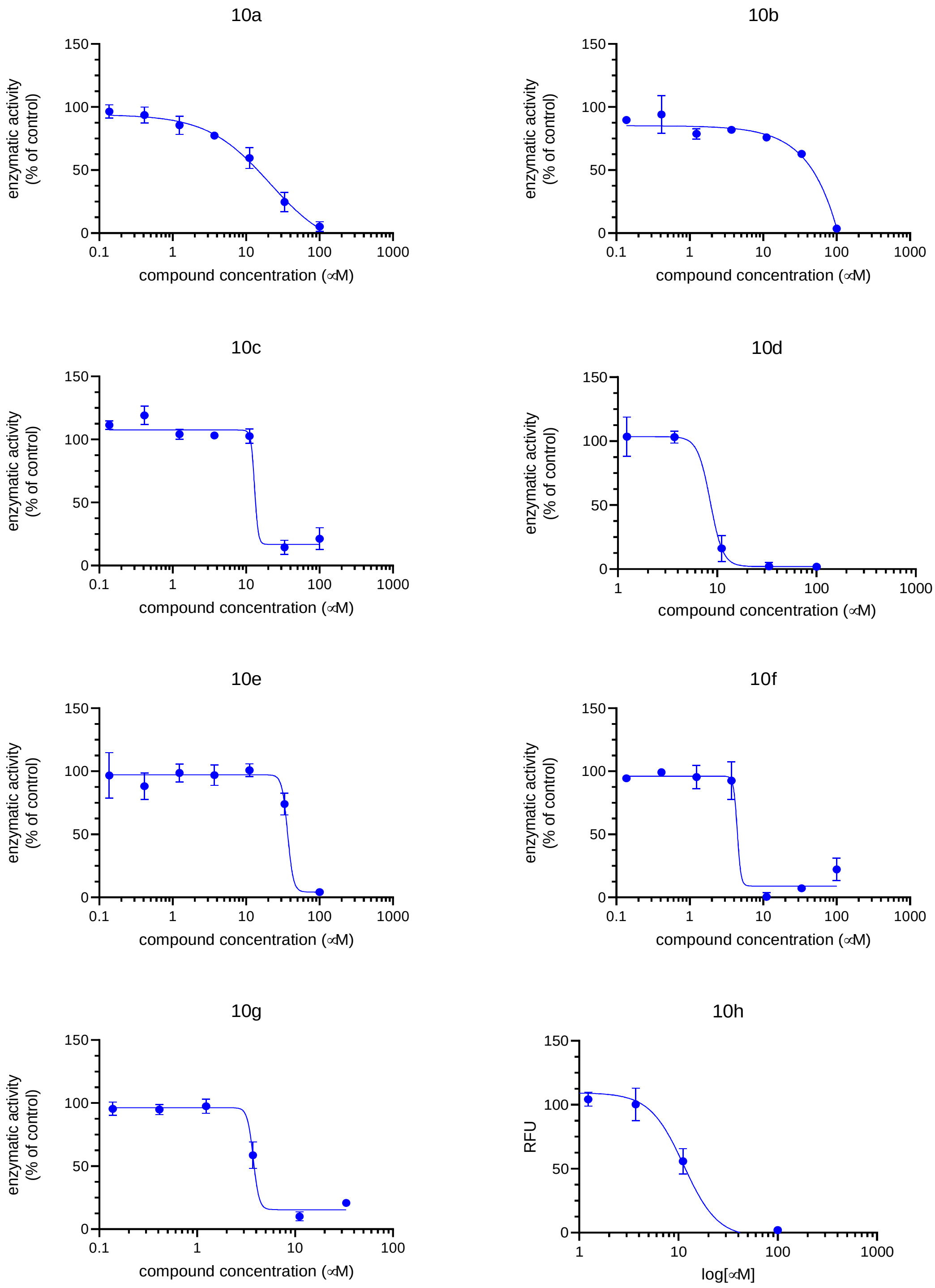

10a
10b
10c
10d
10e
10f
10g
10h
enzymatic activity
(% of control)
compound concentration (∝M)
RFU
log[∝M]
150
100
50
0
0.1
1
10
100
1000

B

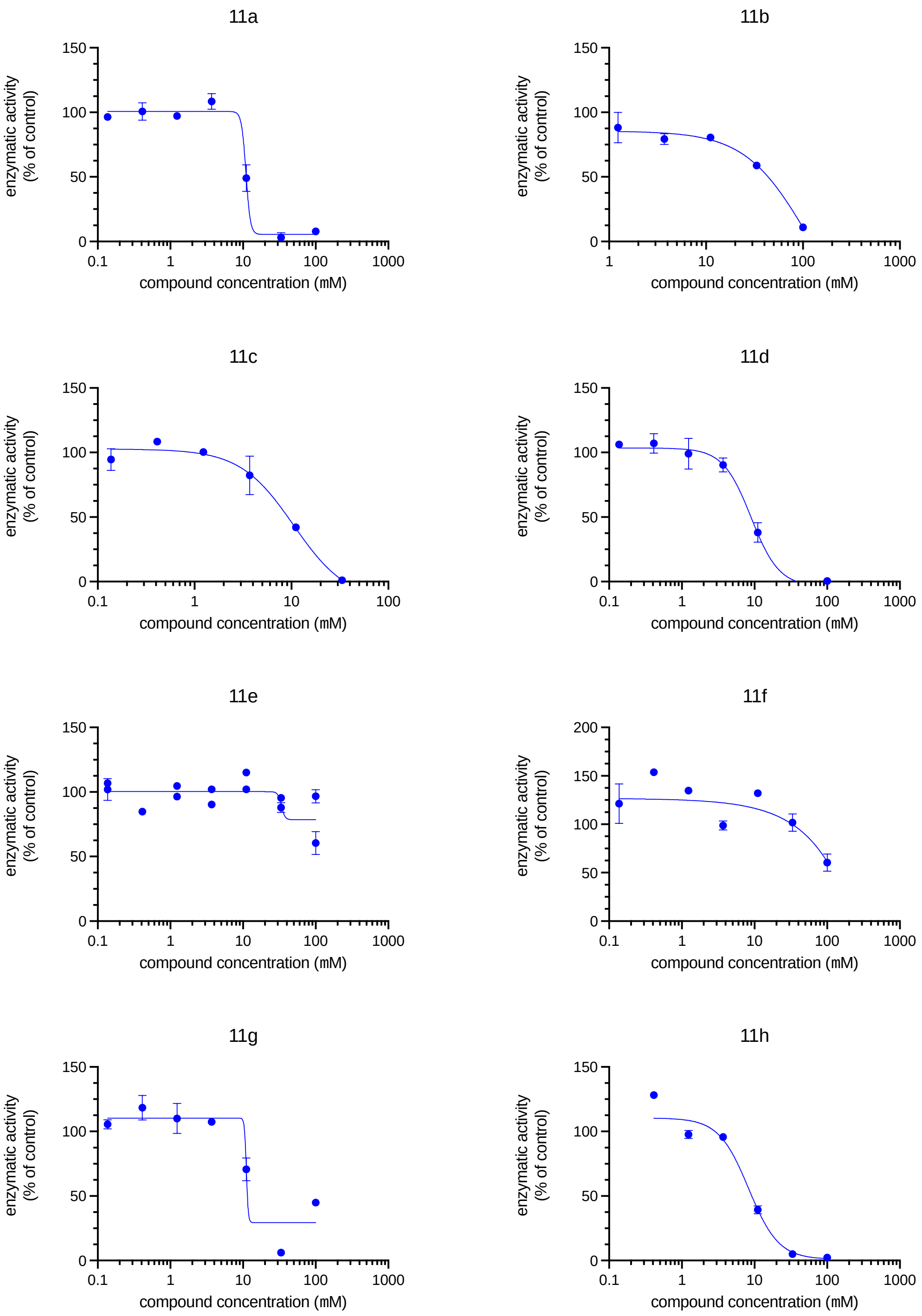
11a
11b
11c
11d
11e
11f
11g
11h
enzymatic activity
(% of control)
compound concentration (mM)
0
50
100
150
200
0.1
1
10
100
1000

C

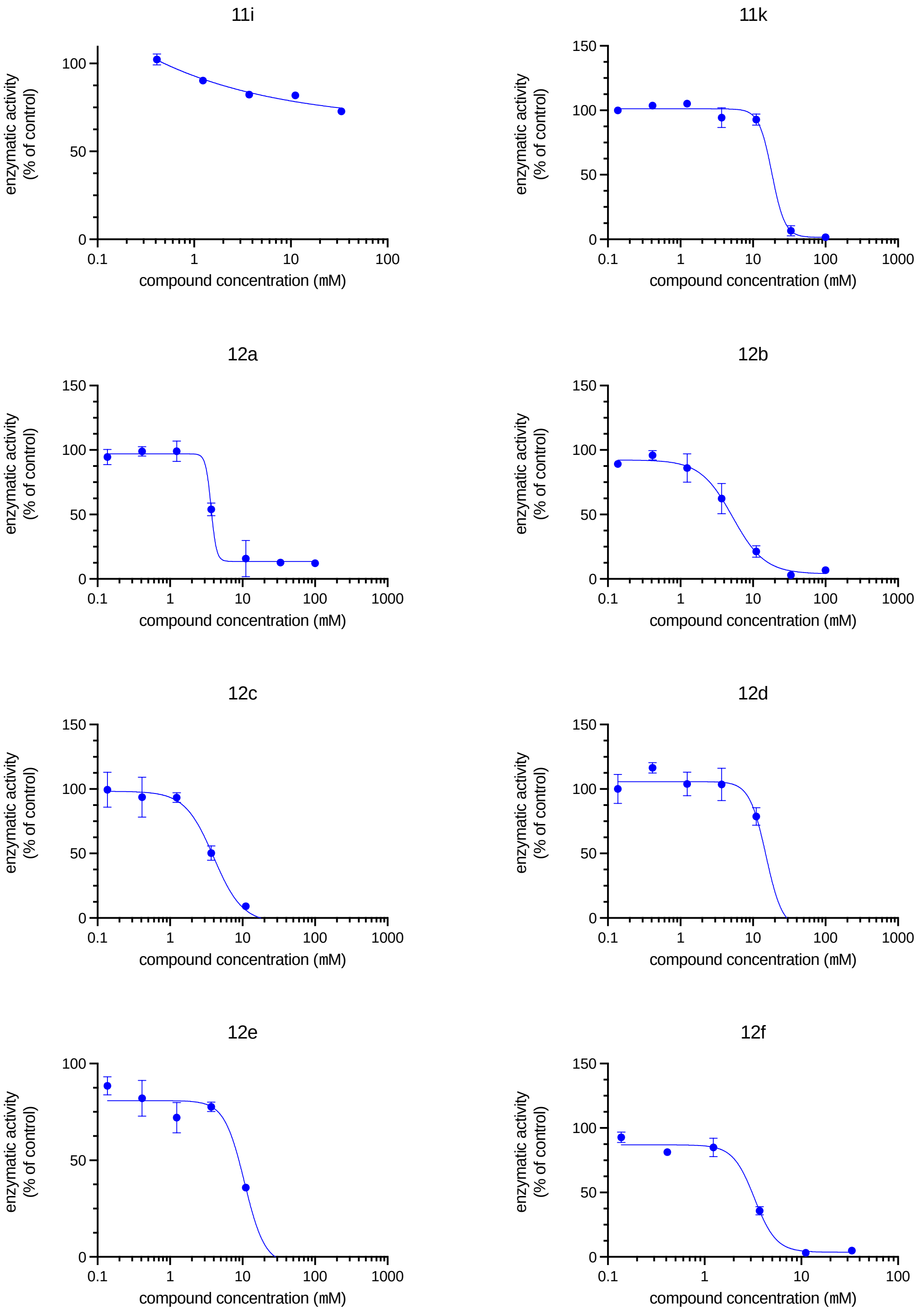
11i
11k
12a
12b
12c
12d
12e
12f
enzymatic activity
(% of control)
compound concentration (mM)
0
50
100
150
0.1
1
10
100
1000

D

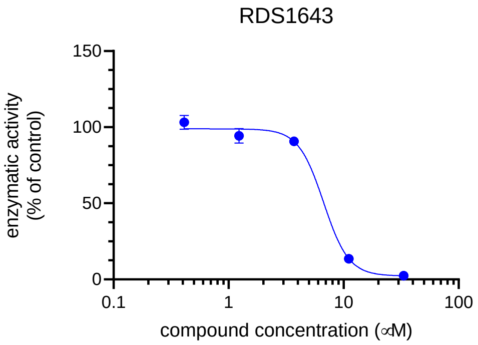

Figure S2. Inhibitory profile of compounds on HIV-1 RNase H activity. Panels A, B and C show the inhibitory profile of tested compounds, results represent the mean percentage ± sd of enzymatic activity calculated with respect to the control in presence of indicated concentration of compound. Experiments were performed in triplicate with at least three replicates per concentration. D panel shows the inhibitory profile of RDS1643 used as positive control

4. Magnesium Complexation

Complexation assays with Mg^{2+} were carried out on compounds **12b**. The effects of the Mg^{2+} were evaluated by a spectrophotometric method, using the UV Spectrophotometer Lambda 1050+ and a Hellma quartz cuvette with a 1 cm optical path. $MgCl_2{\cdot}6H_2O$ was purchased from Aladdin Company. In the experimental study of the binding of compound **12b** with Mg^{2+}, we prepared solvent with a volume ratio of EtOH:dioxane:water = 1:1:1. We prepared 1000ml of this solvent and then weighed 25.75 mg of compound **12b** and dissolved it in the solvent. We take 500 mL of the solution and add magnesium chloride to achieve Mg^{2+} concentration of 10^{-3} M. Each cuvette contained 3ml solution, and the UV–vis spectrum was recorded between 150 and 600 nm.

5. ^{1}H-NMR, ^{13}C-NMR spectra and HRMS of compounds

Methyl 7-methyl-3-oxo-5-phenyl-2,3-dihydro-5H-thiazolo[3,2-a]pyrimidine-6-carboxylate(**9a**).

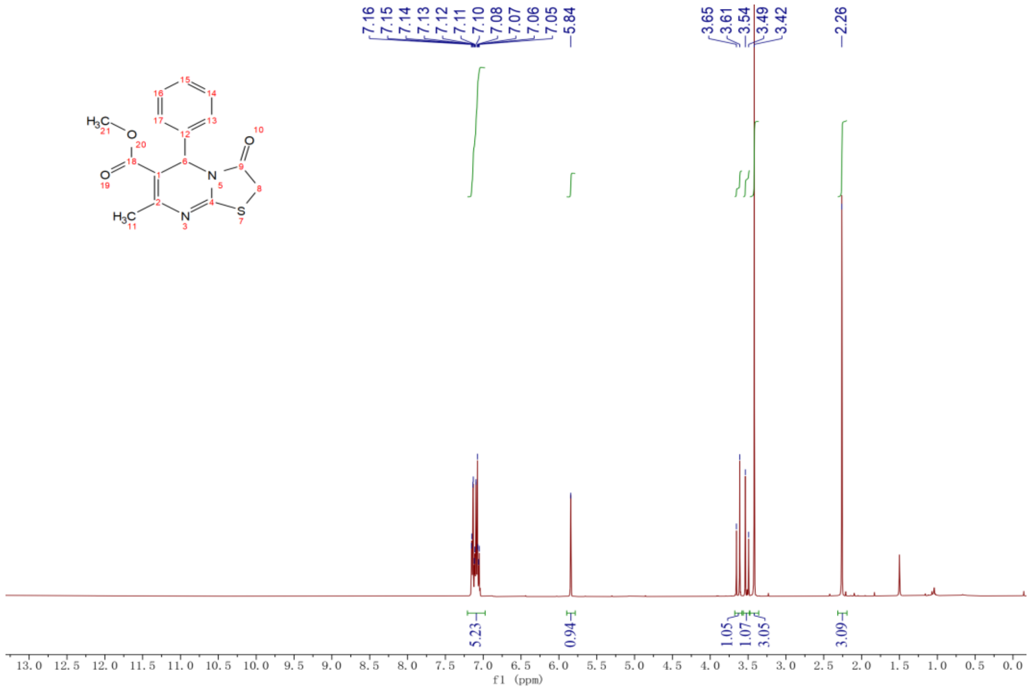

Methyl 5-(4-methoxyphenyl)-7-methyl-3-oxo-2,3-dihydro-5H-thiazolo[3,2-a]pyrimidine-6-carboxylate(**9c**).

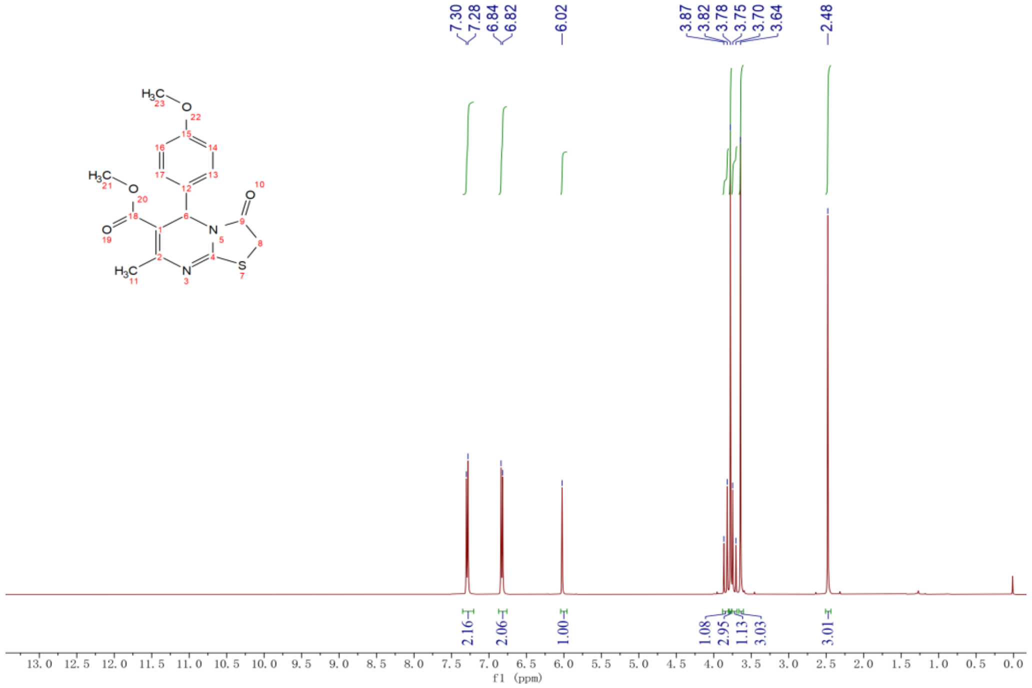

Methyl 7-methyl-3-oxo-5-(2,3,4-trimethoxyphenyl)-2,3-dihydro-5H-thiazolo[3,2-a]pyrimidine-6-carboxylate(**9b**).

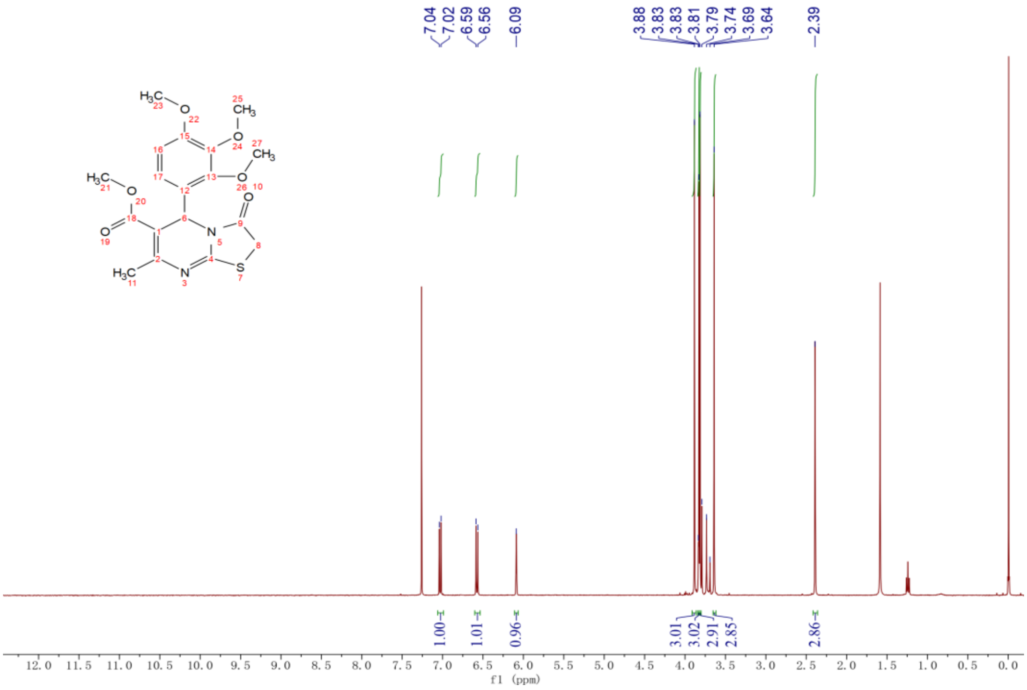

Methyl 2-(2-(2-hydroxyphenyl)hydrazineylidene)-7-methyl-3-oxo-5-phenyl-2, 3-dihydro-5H-thiazolo[3, 2-a]pyrimidine-6-carboxylate (**10a**).

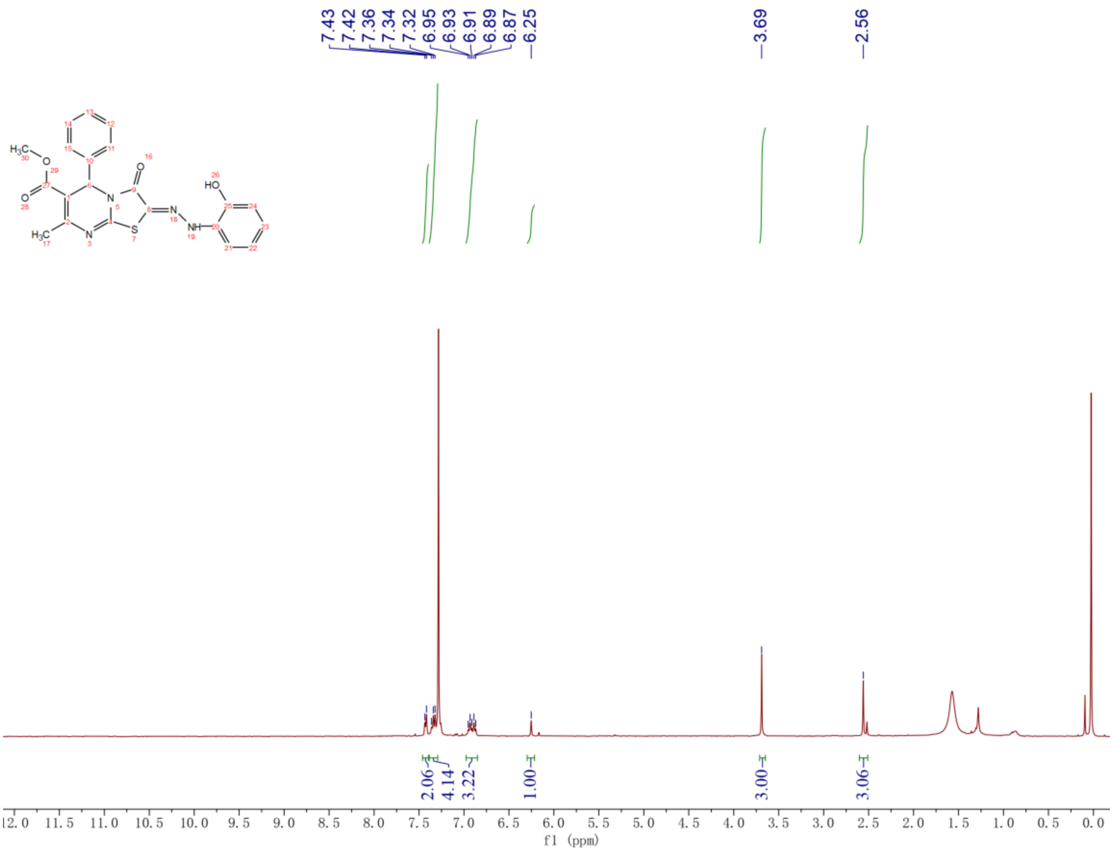

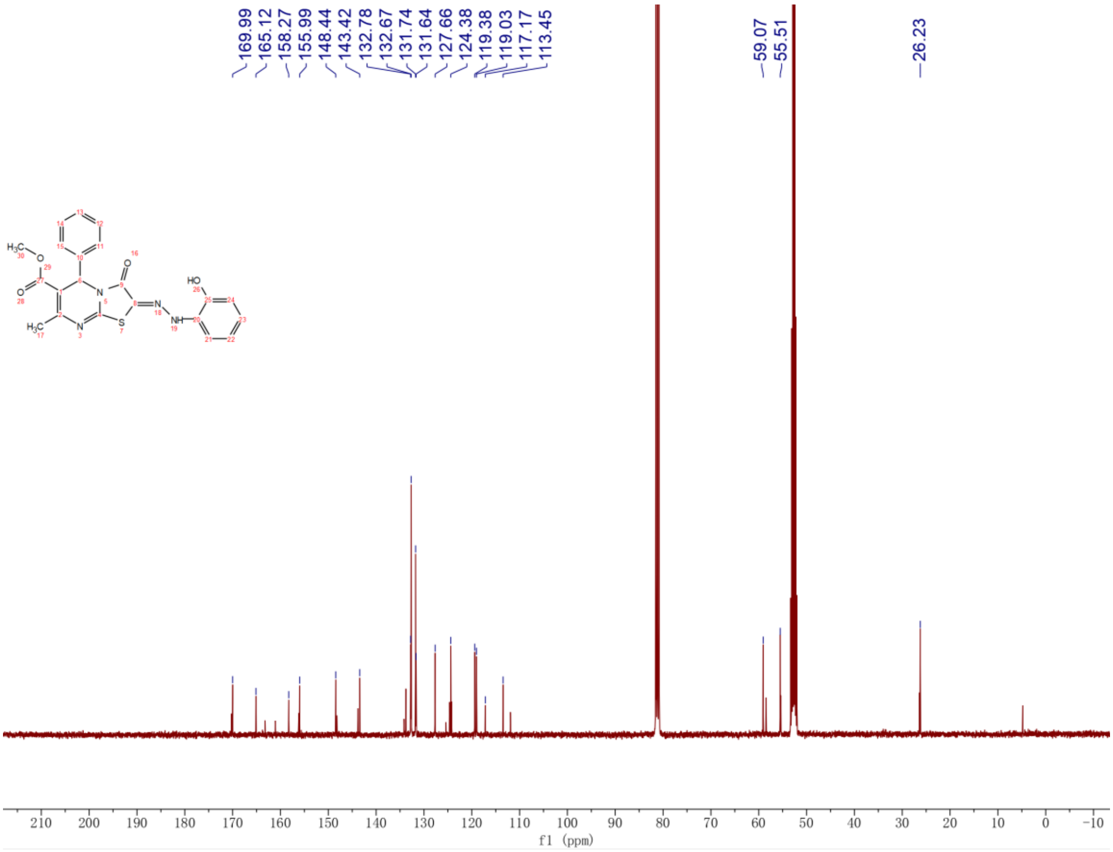

(2-(6-(Methoxycarbonyl)-7-methyl-3-oxo-5-phenyl-5H-thiazolo[3, 2-a]pyrimidin-2(3H)-ylidene)hydrazineyl)-3-methylbenzoic acid **(10b)**.

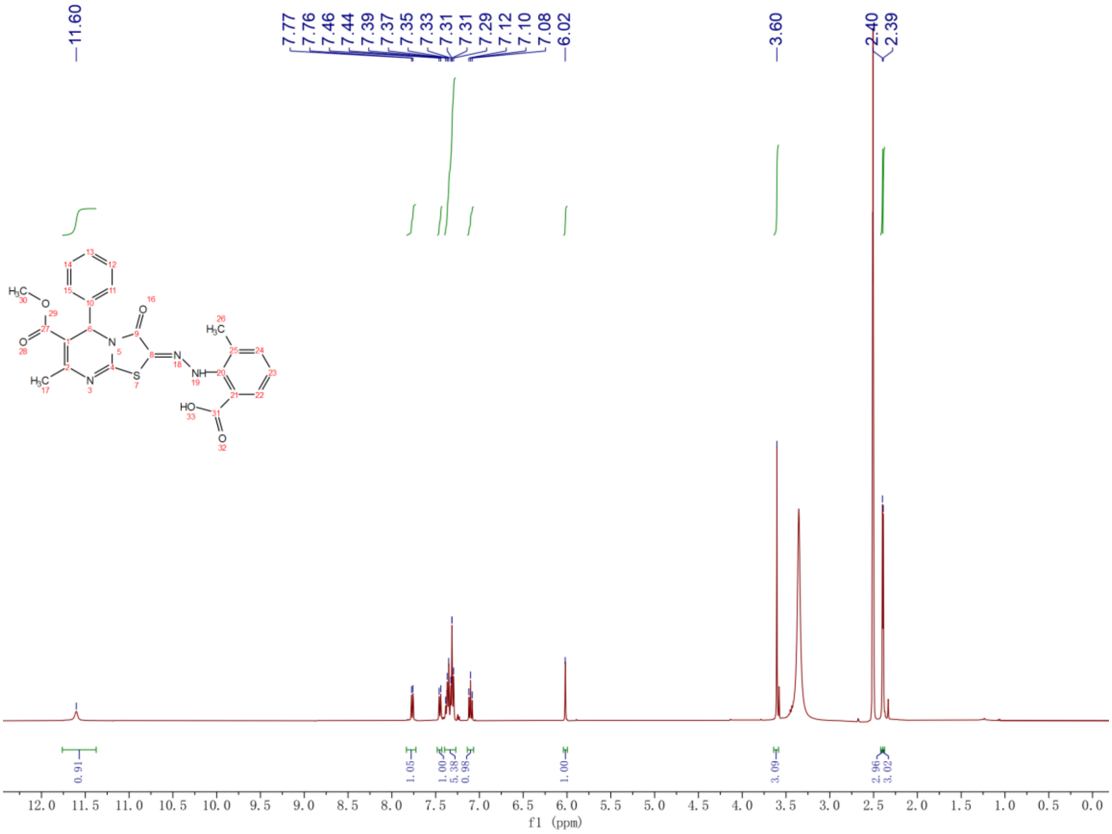

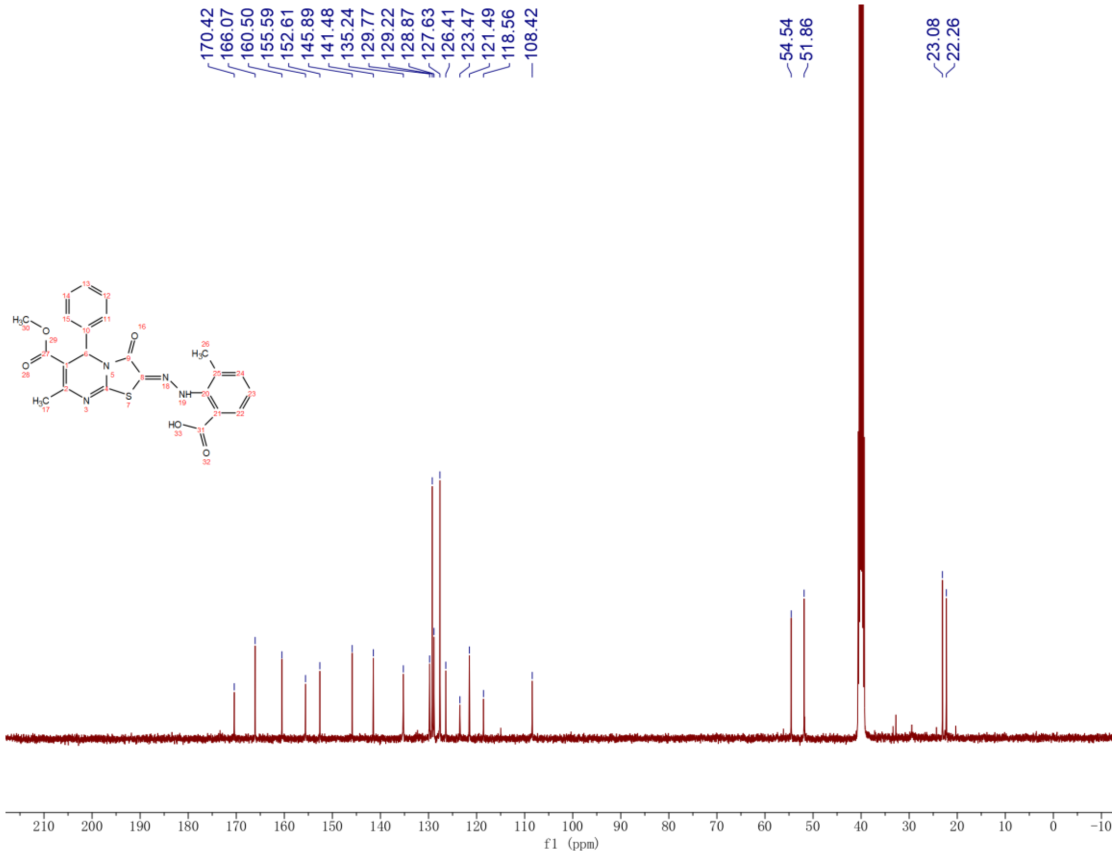

Methyl 7-methyl-3-oxo-5-phenyl-2-(2-(3, 4, 5-trimethoxyphenyl)hydrazineylidene)-2, 3-dihydro-5H-thiazolo[3, 2-a]pyrimidine-6-carboxylate **(10c)**.

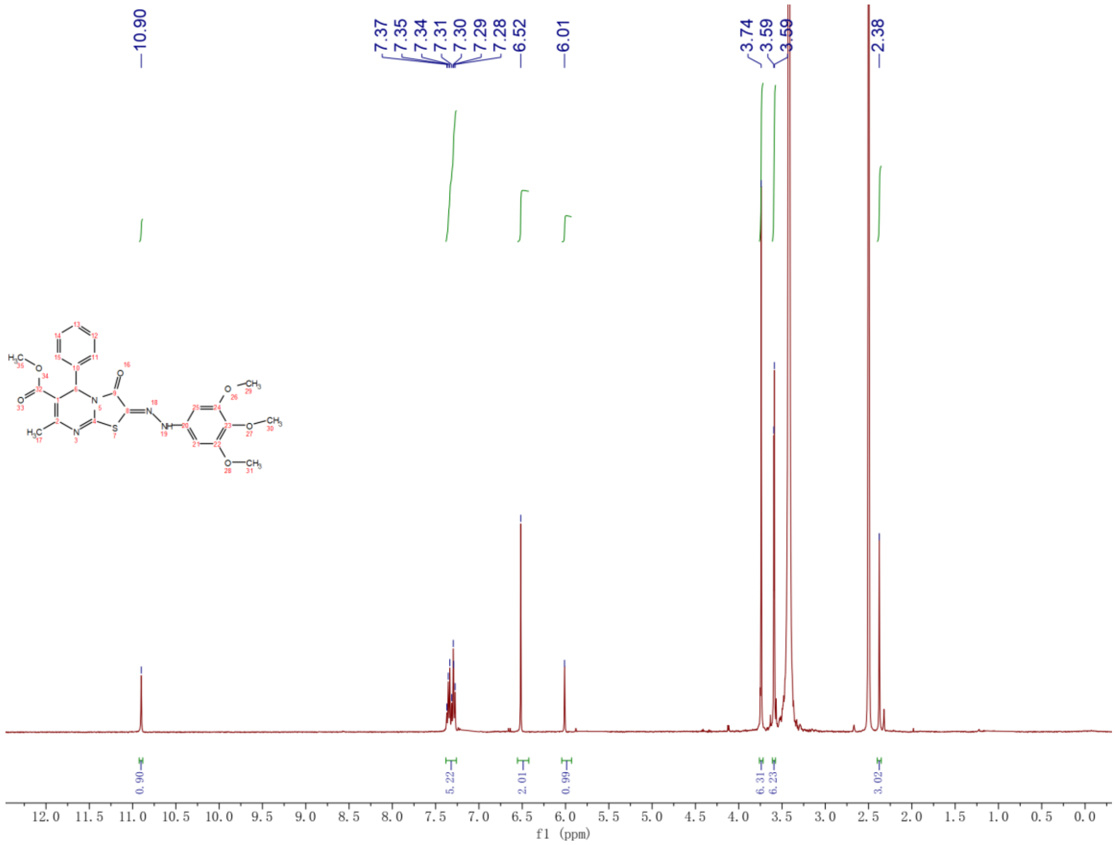

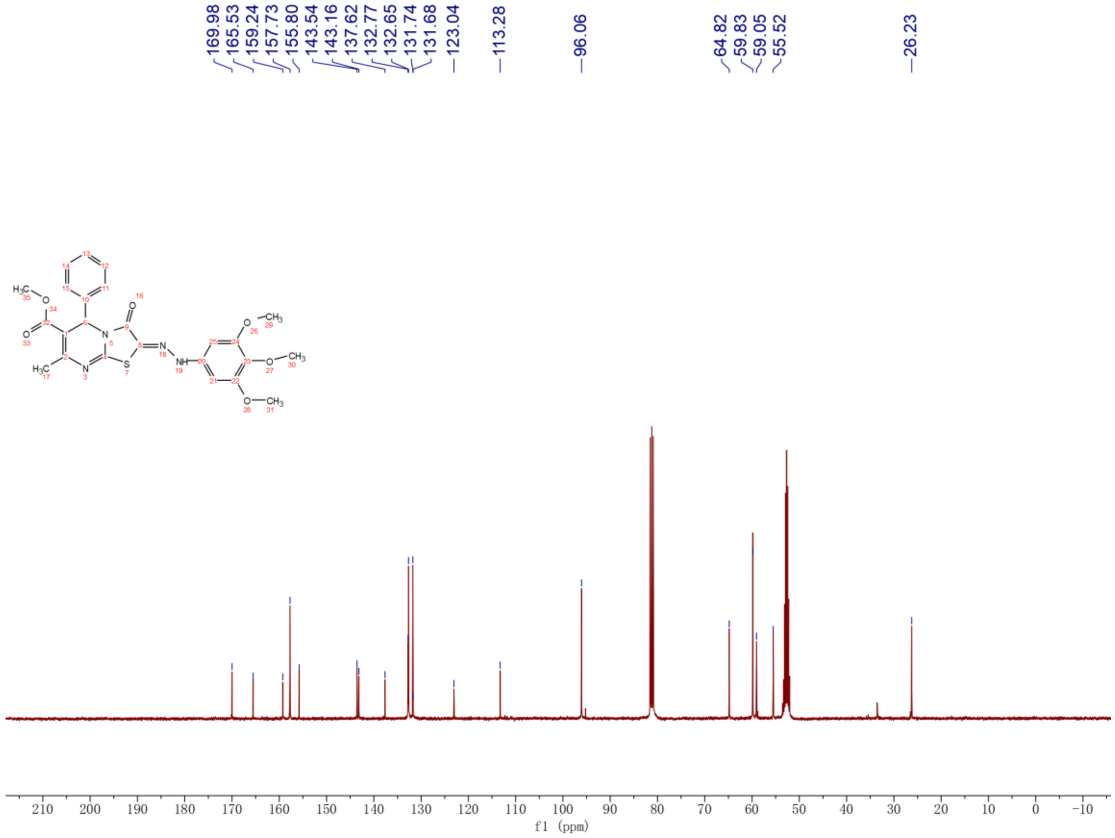

Methyl 2-((2-(methoxycarbonyl)phenyl)diazenyl)-7-methyl-3-oxo-5-phenyl-2, 3-dihydro-5H-thiazolo[3, 2-a]pyrimidine-6-carboxylate **(10d)**.

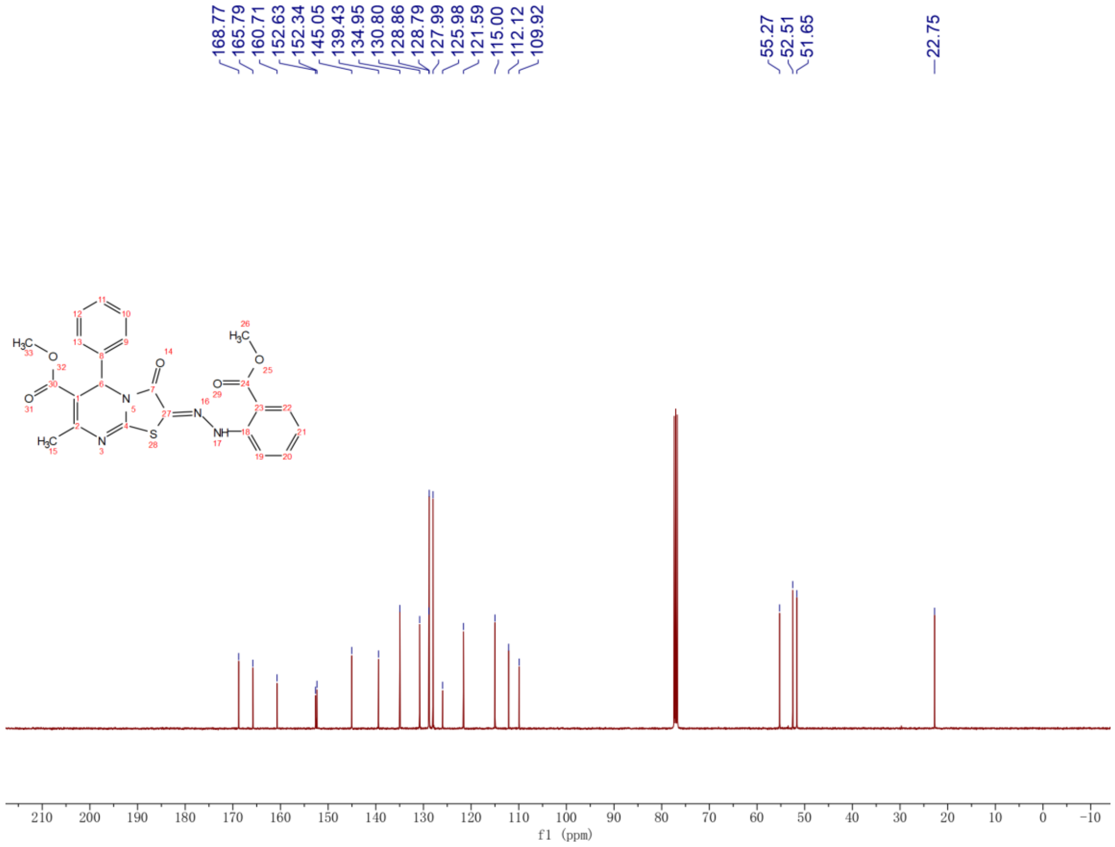

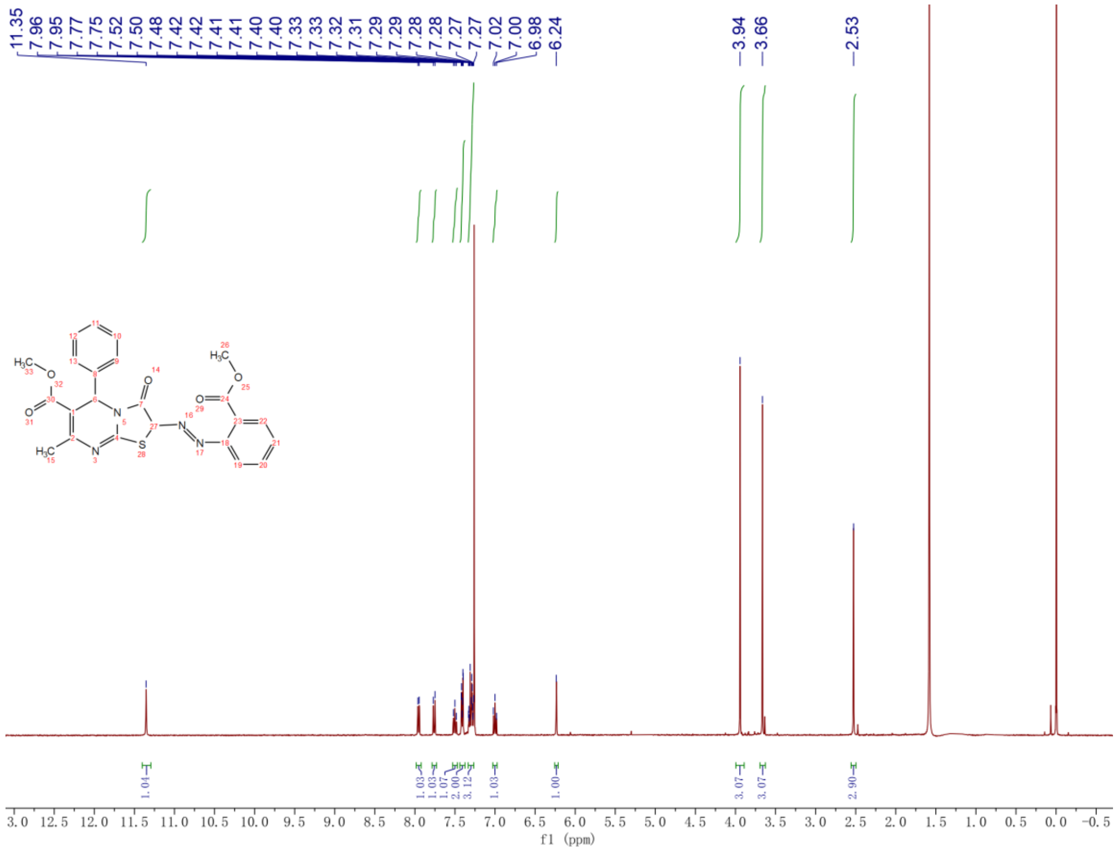

5-Methoxy-2-(2-(6-(methoxycarbonyl)-7-methyl-3-oxo-5-phenyl-5H-thiazolo[3, 2-a]pyrimidin-2(3H)-ylidene)hydrazineyl)benzenesulfonic acid **(10e)**.

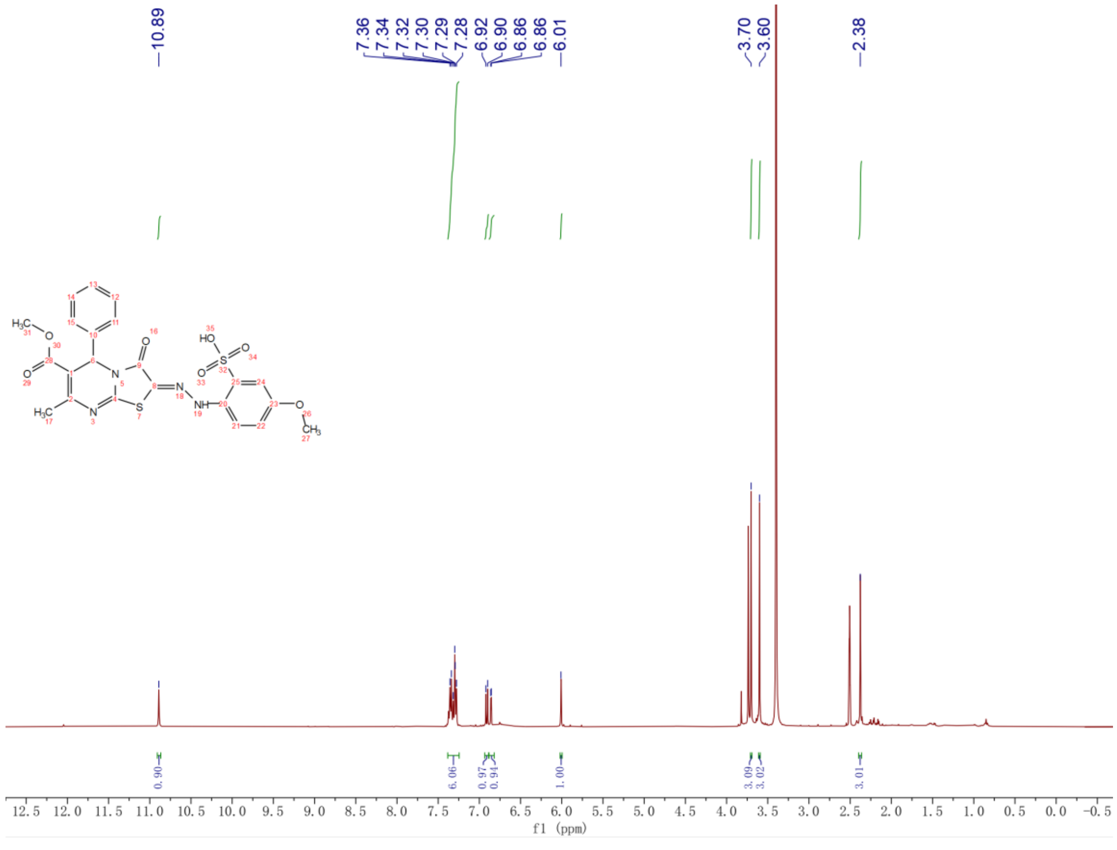

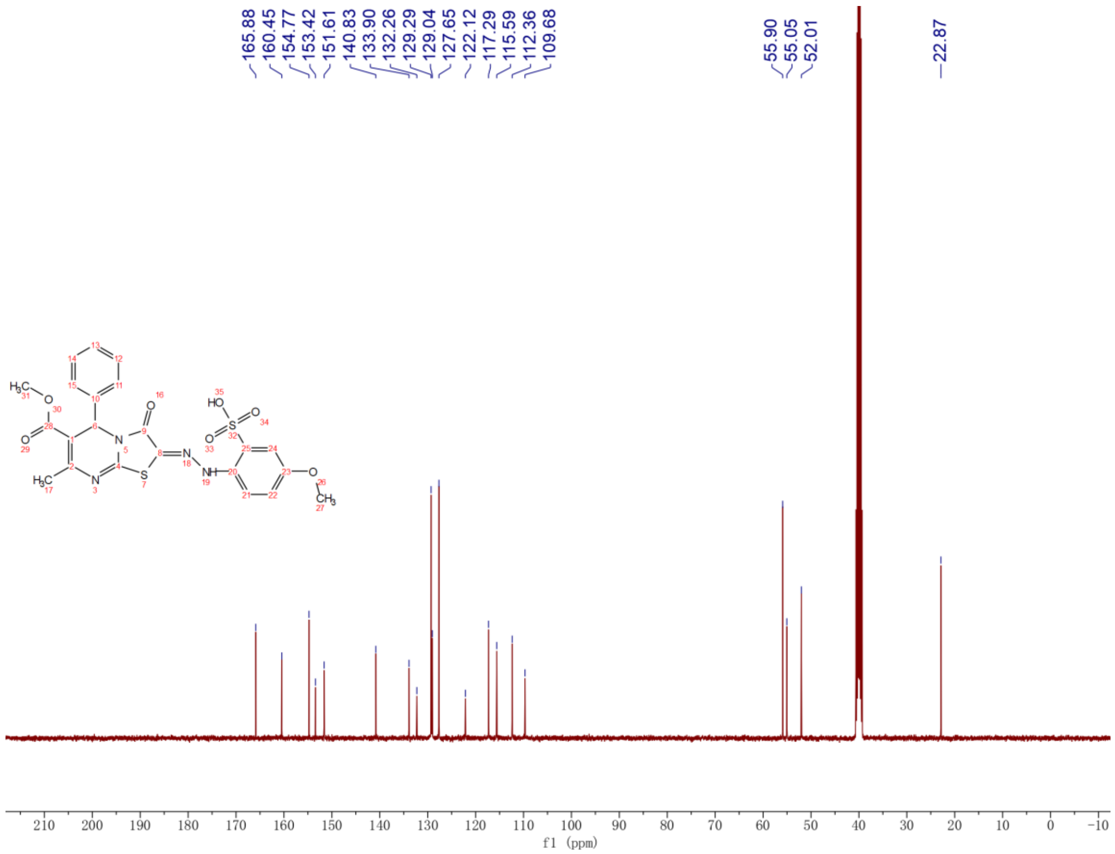

(6-(Methoxycarbonyl)-7-methyl-3-oxo-5-phenyl-5H-thiazolo[3, 2-a]pyrimidin-2(3H)-ylidene)hydrazineyl)naphthalene-1-sulfonic acid **(10f)**.

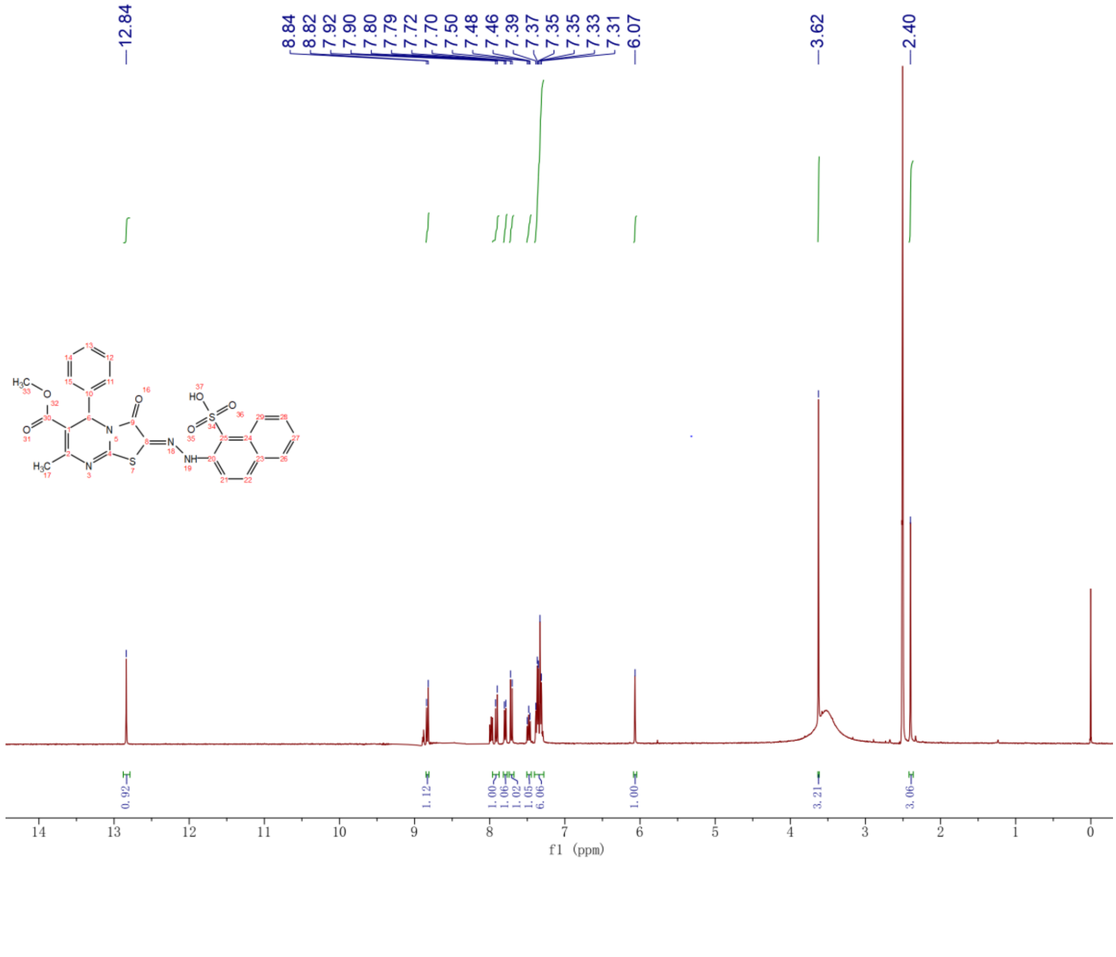

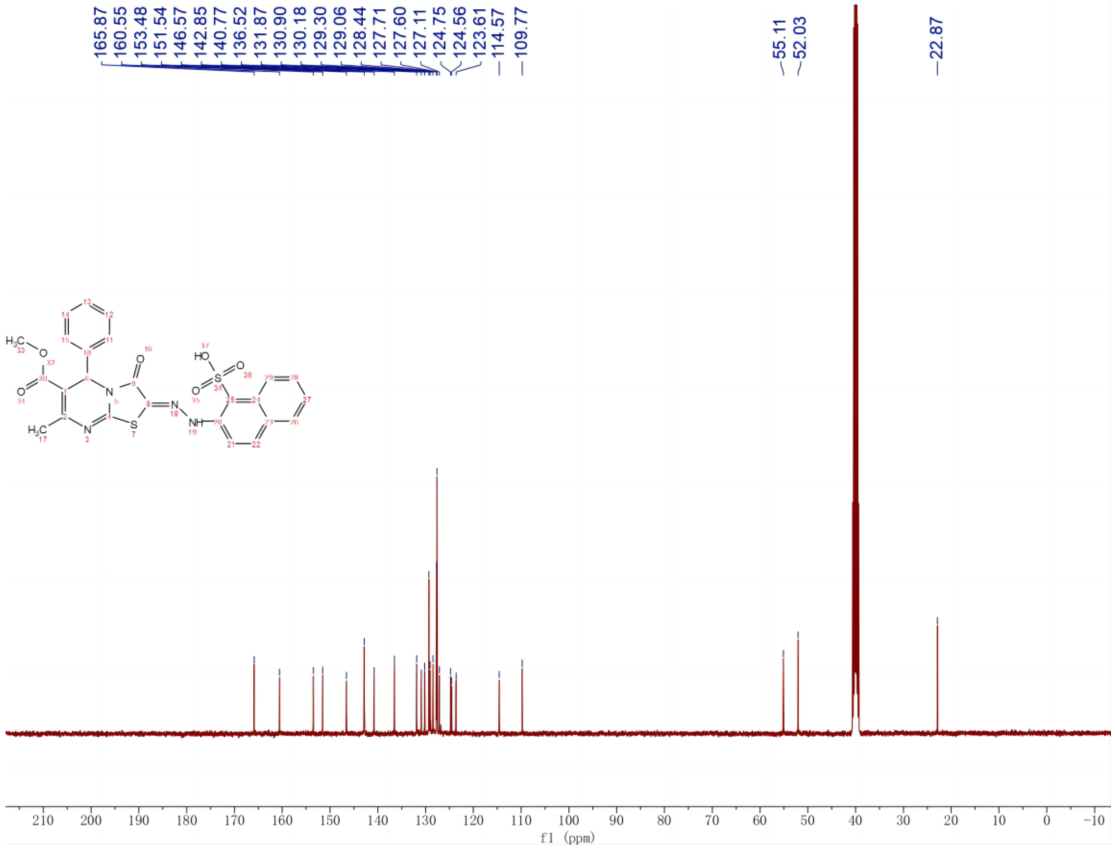

Methyl 2-(2-(2-acetylphenyl)hydrazineylidene)-7-methyl-3-oxo-5-phenyl-2, 3-dihydro-5H-thiazolo[3, 2-a]pyrimidine-6-carboxylate **(10g)**.

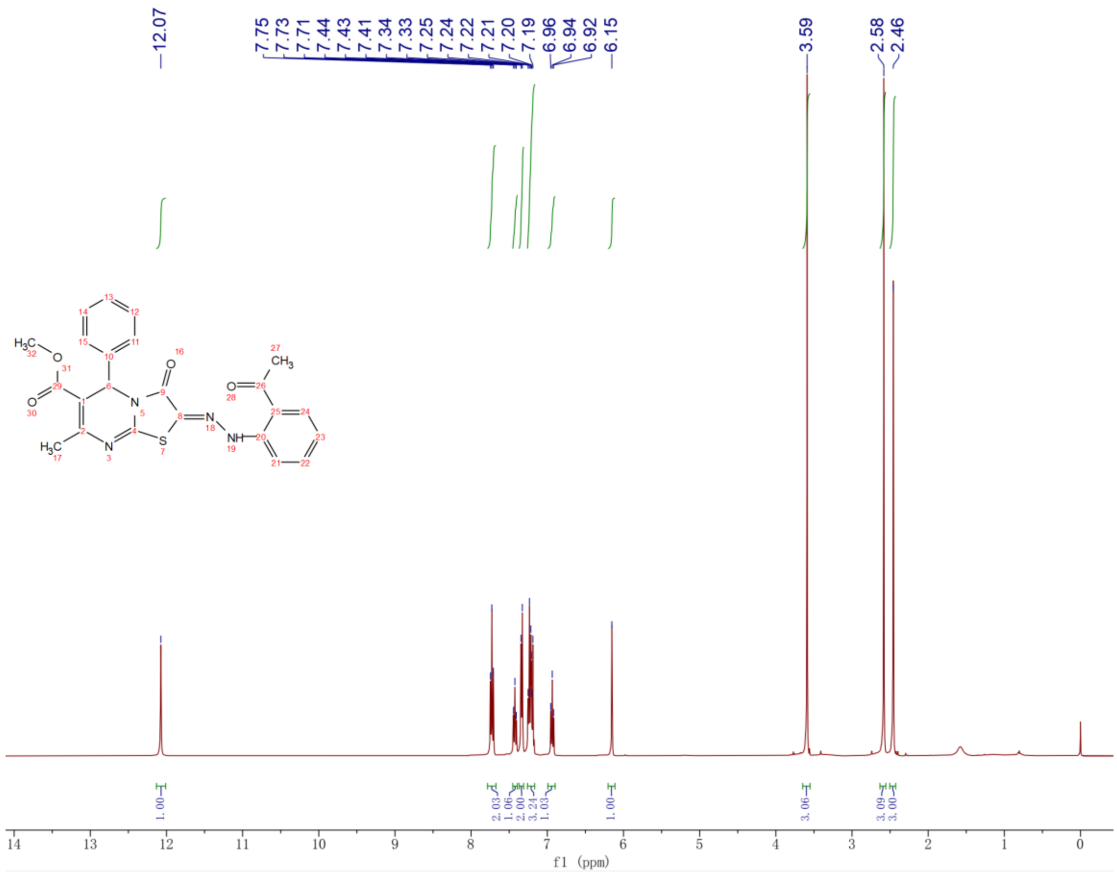

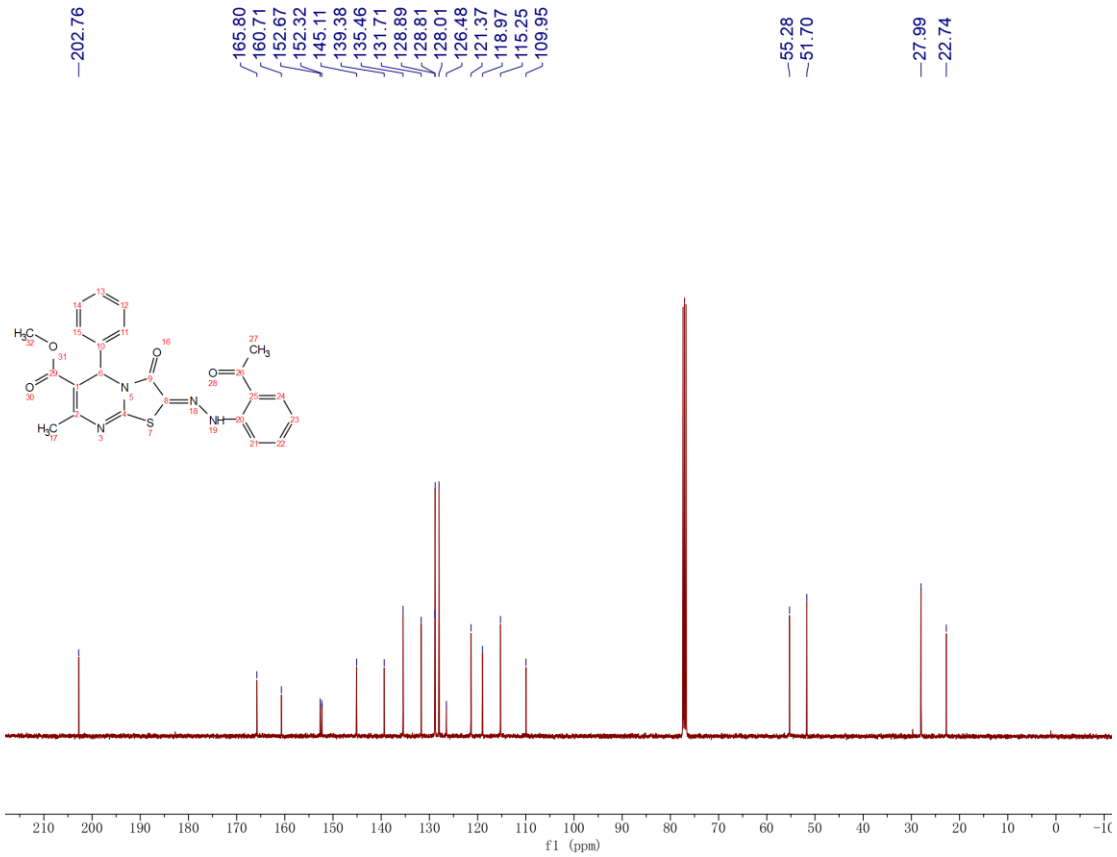

Methyl 2-(2-(3, 4-dimethoxyphenyl)hydrazineylidene)-7-methyl-3-oxo-5-phenyl-2, 3-

dihydro-5H-thiazolo[3, 2-a]pyrimidine-6-carboxylate **(10h)**.

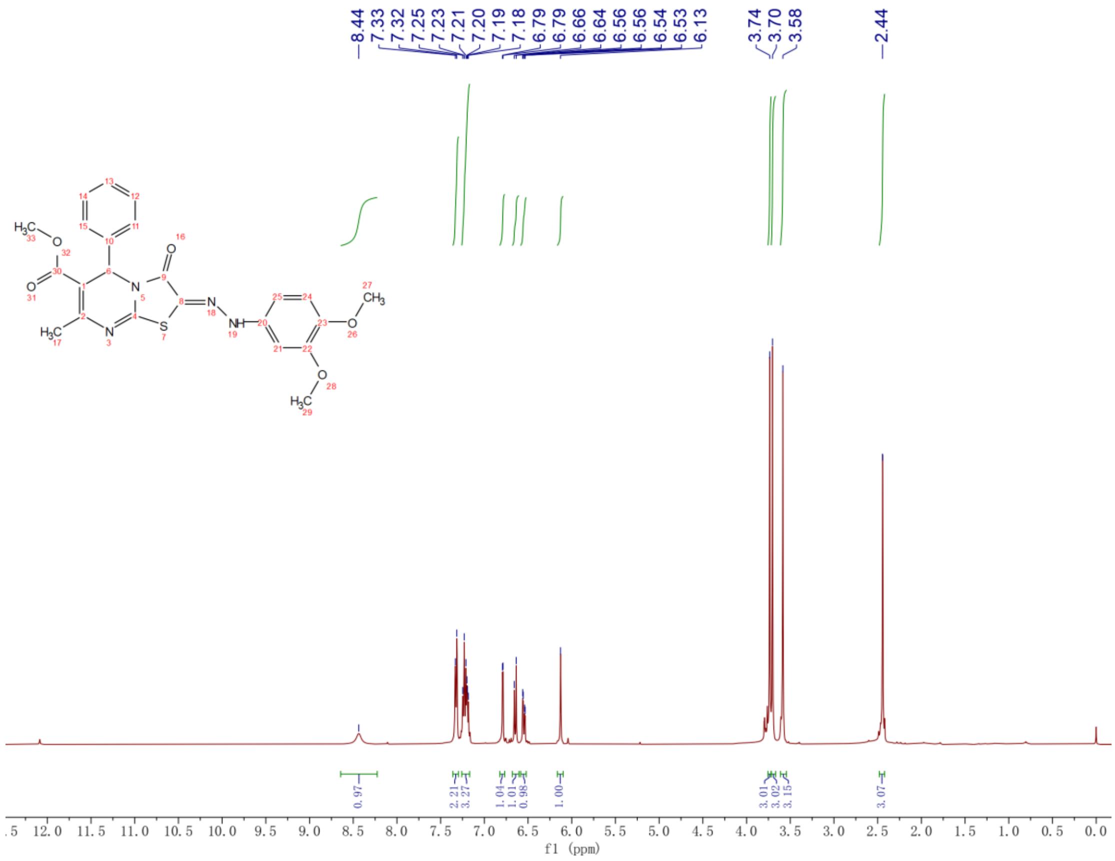

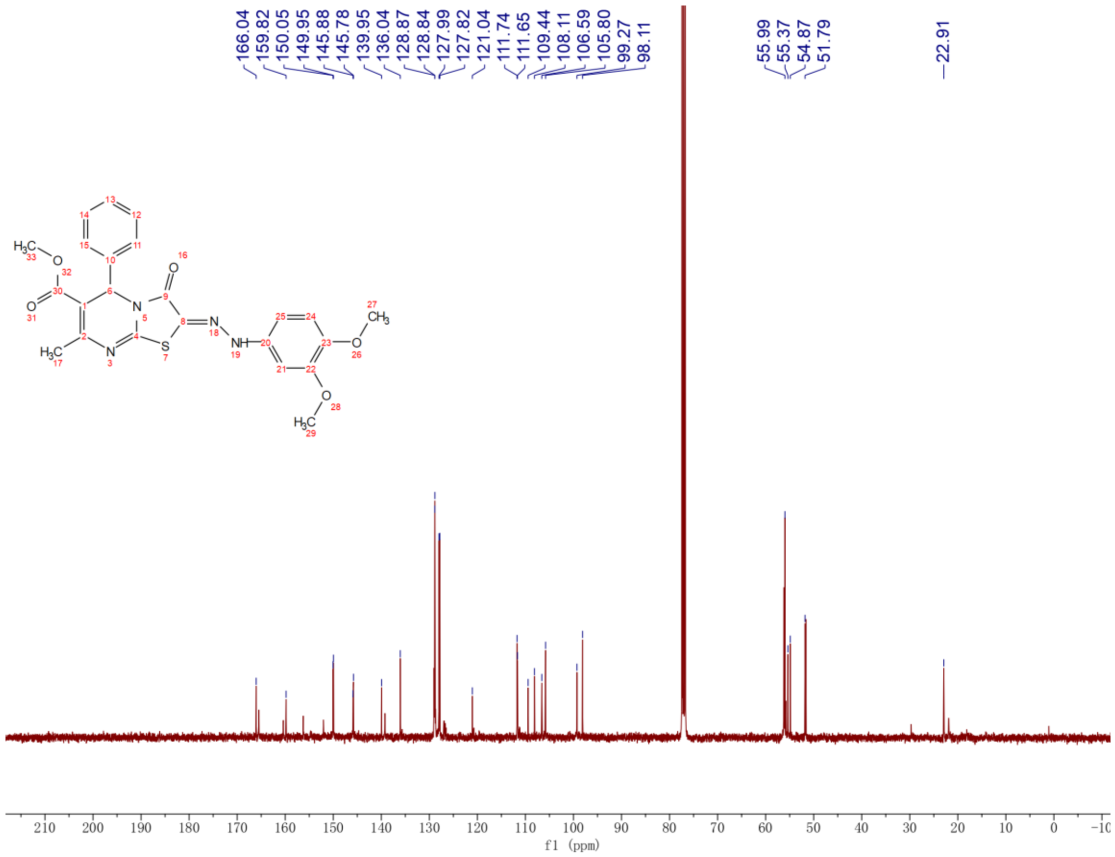

Methyl 2-(2-(2-methoxyphenyl)hydrazineylidene)-7-methyl-3-oxo-5-phenyl-2, 3-dihydro-5H-thiazolo[3, 2-a]pyrimidine-6-carboxylate **(10i)**.

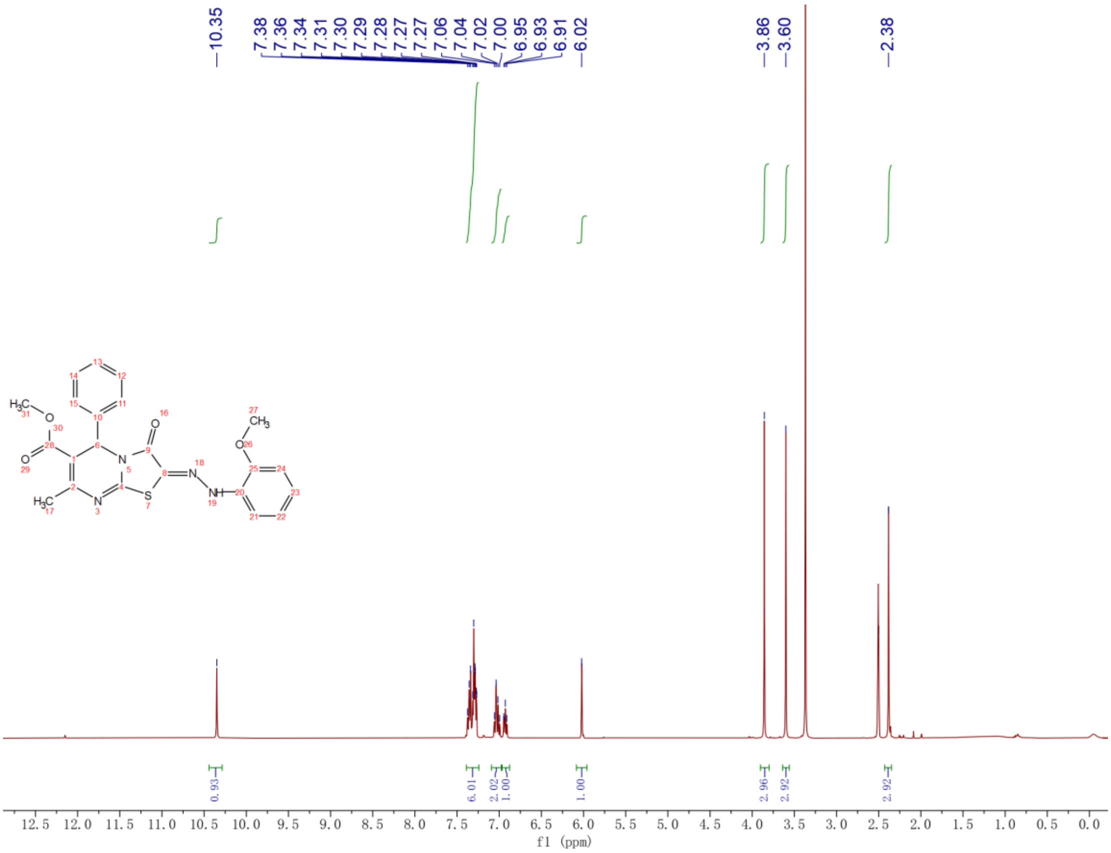

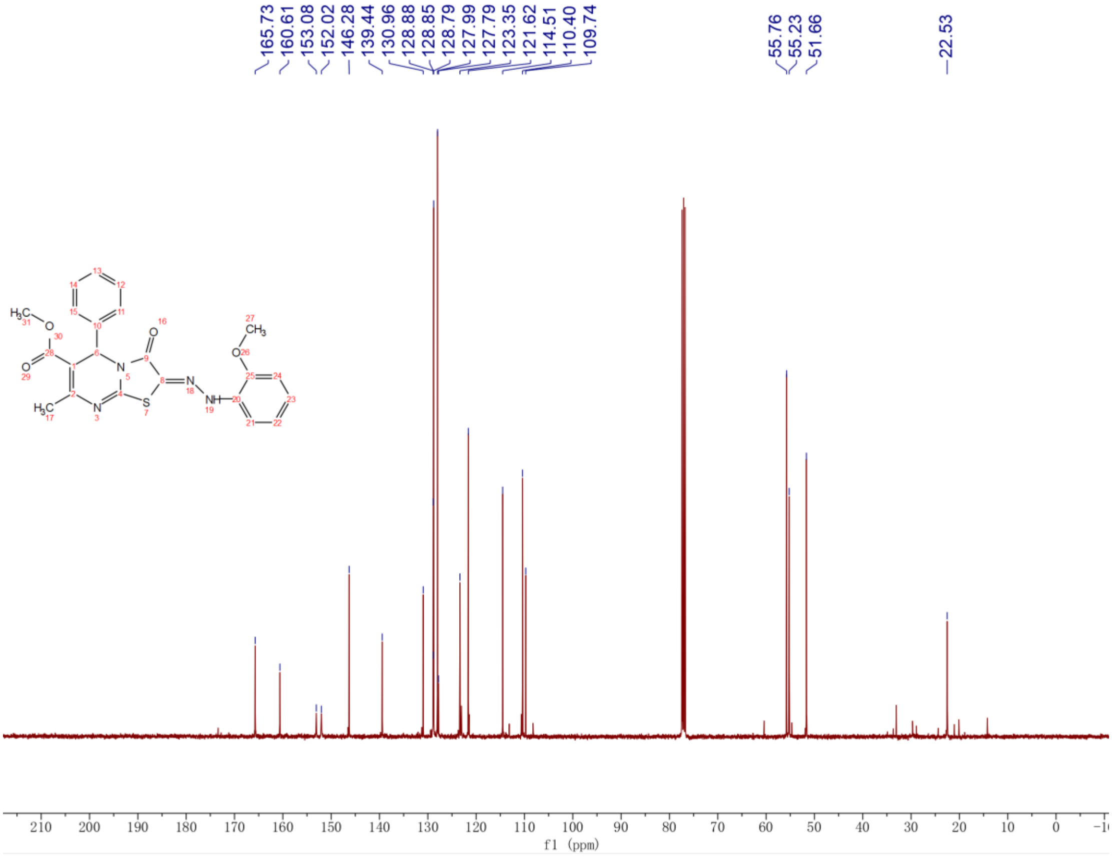

Methyl 7-methyl-3-oxo-5-phenyl-2-(2-phenylhydrazineylidene)-2, 3-dihydro-5H-thiazolo[3, 2-a]pyrimidine-6-carboxylate **(10j)**.

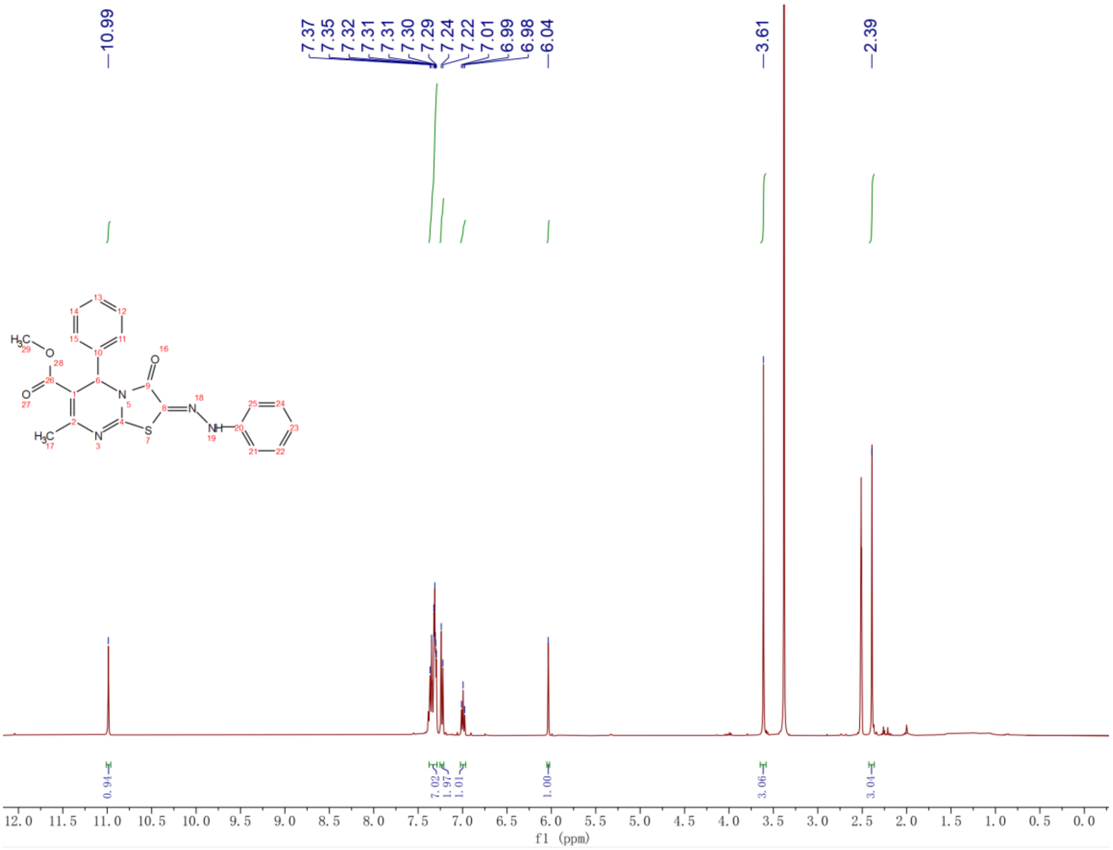

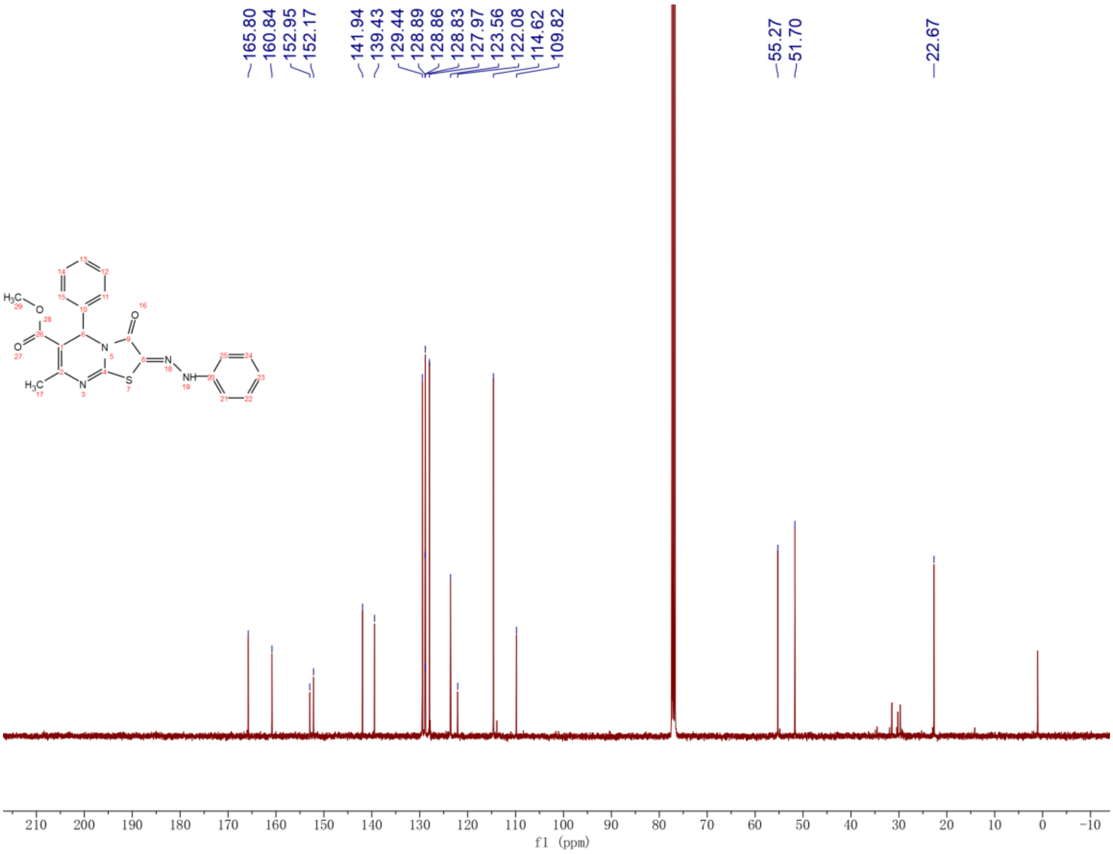

(2-(6-(Methoxycarbonyl)-7-methyl-3-oxo-5-phenyl-5H-thiazolo[3, 2-a]pyrimidin-2(3H)-ylidene)hydrazineyl)benzenesulfonic acid **(10k)**.

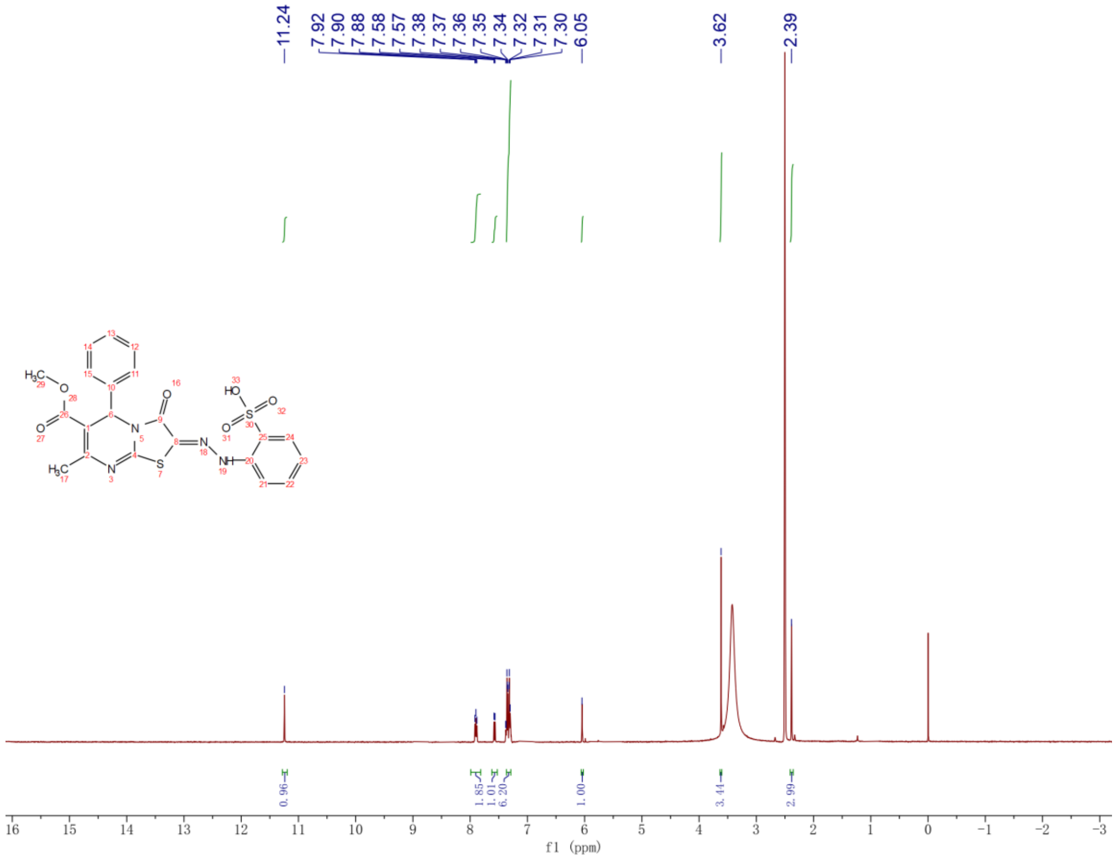

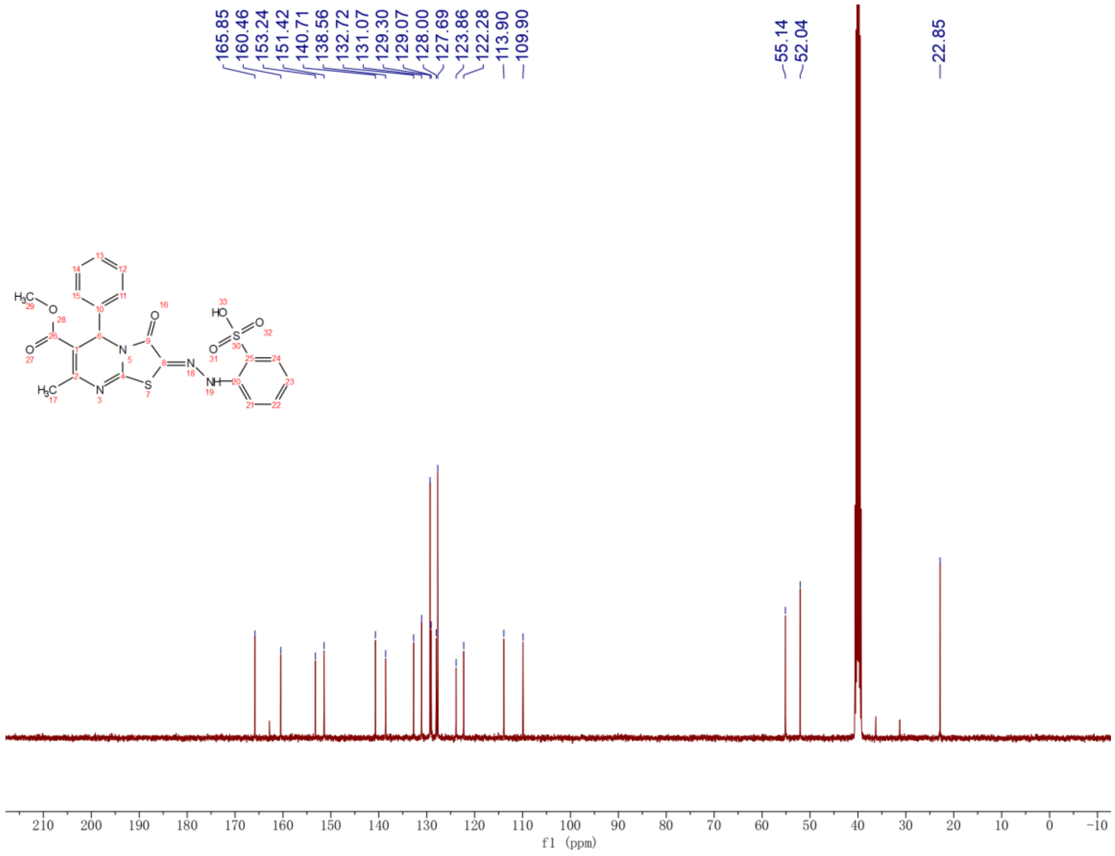

Methyl 7-methyl-2-(2-(3-nitrophenyl)hydrazineylidene)-3-oxo-5-phenyl-2, 3-dihydro-5H-thiazolo[3, 2-a]pyrimidine-6-carboxylate **(10l)**.

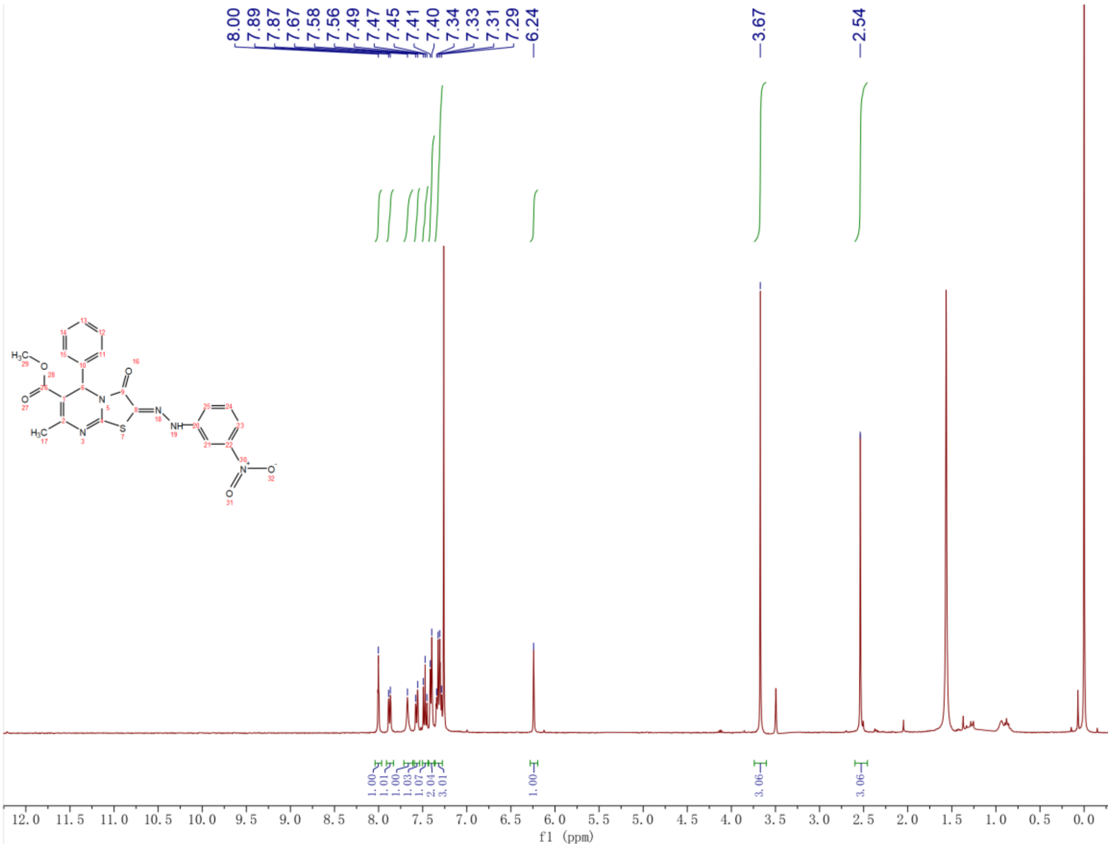

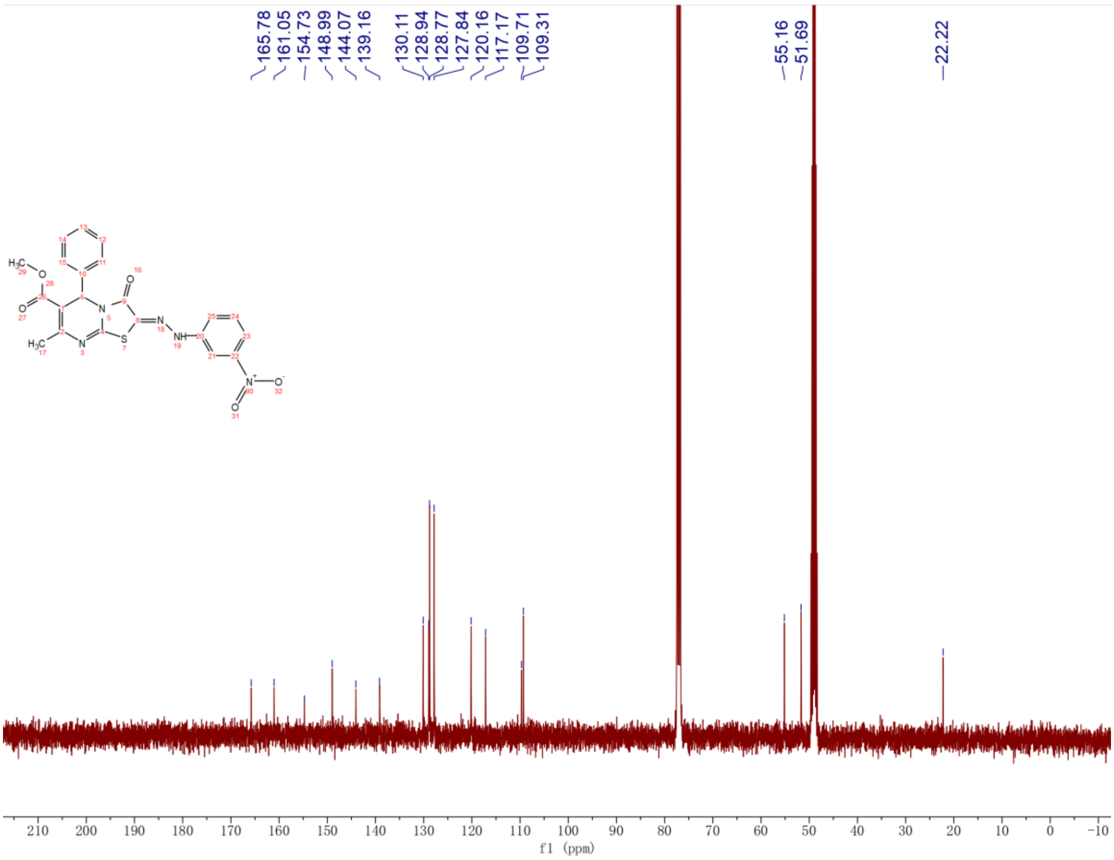

Methyl 2-(2-(3-acetylphenyl)hydrazineylidene)-7-methyl-3-oxo-5-phenyl-2, 3-dihydro-5H-thiazolo[3, 2-a]pyrimidine-6-carboxylate **(10m)**.

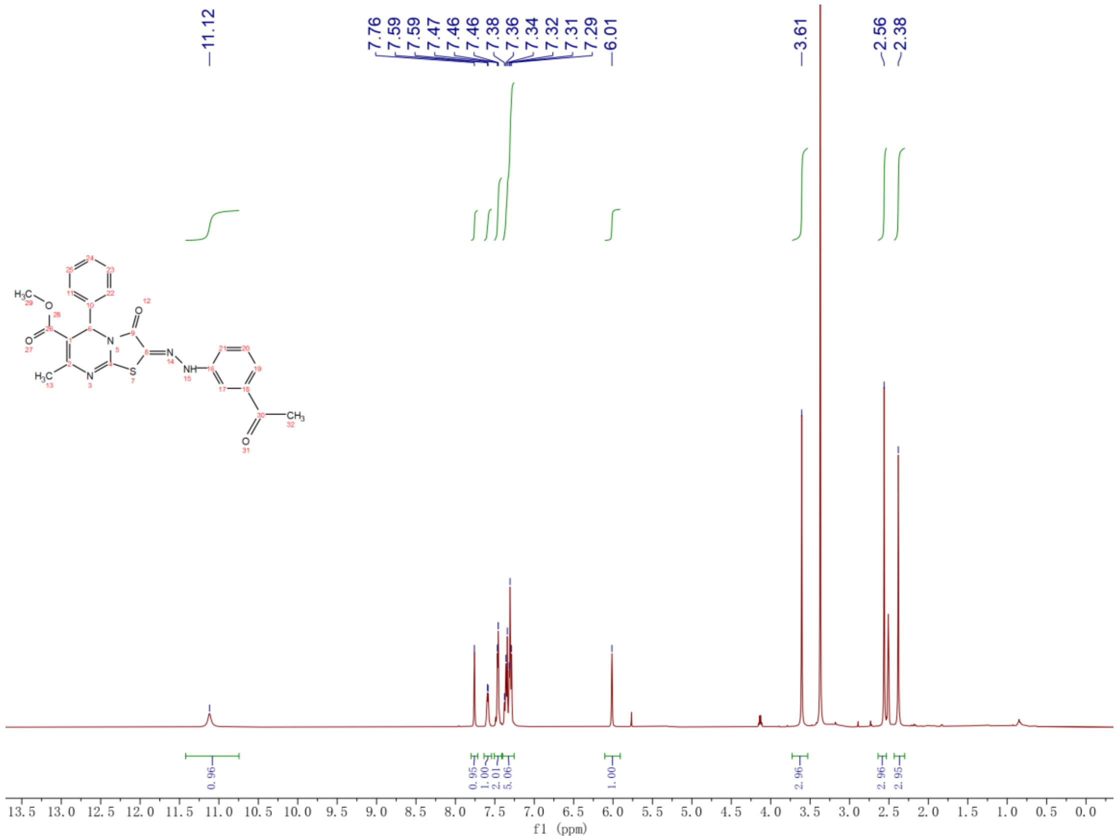

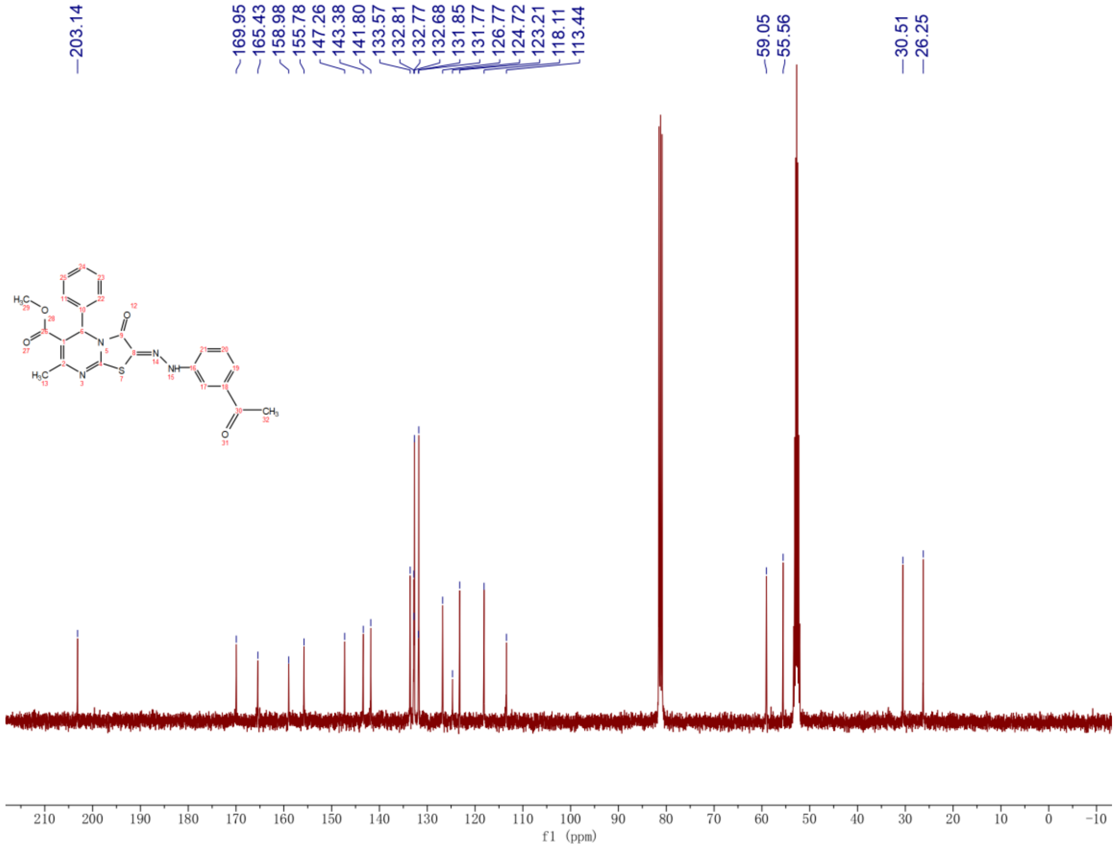

Methyl 2-(2-(3-(methoxycarbonyl)phenyl)hydrazineylidene)-7-methyl-3-oxo-5-phenyl-2, 3-dihydro-5H-thiazolo[3, 2-a]pyrimidine-6-carboxylate **(10n)**.

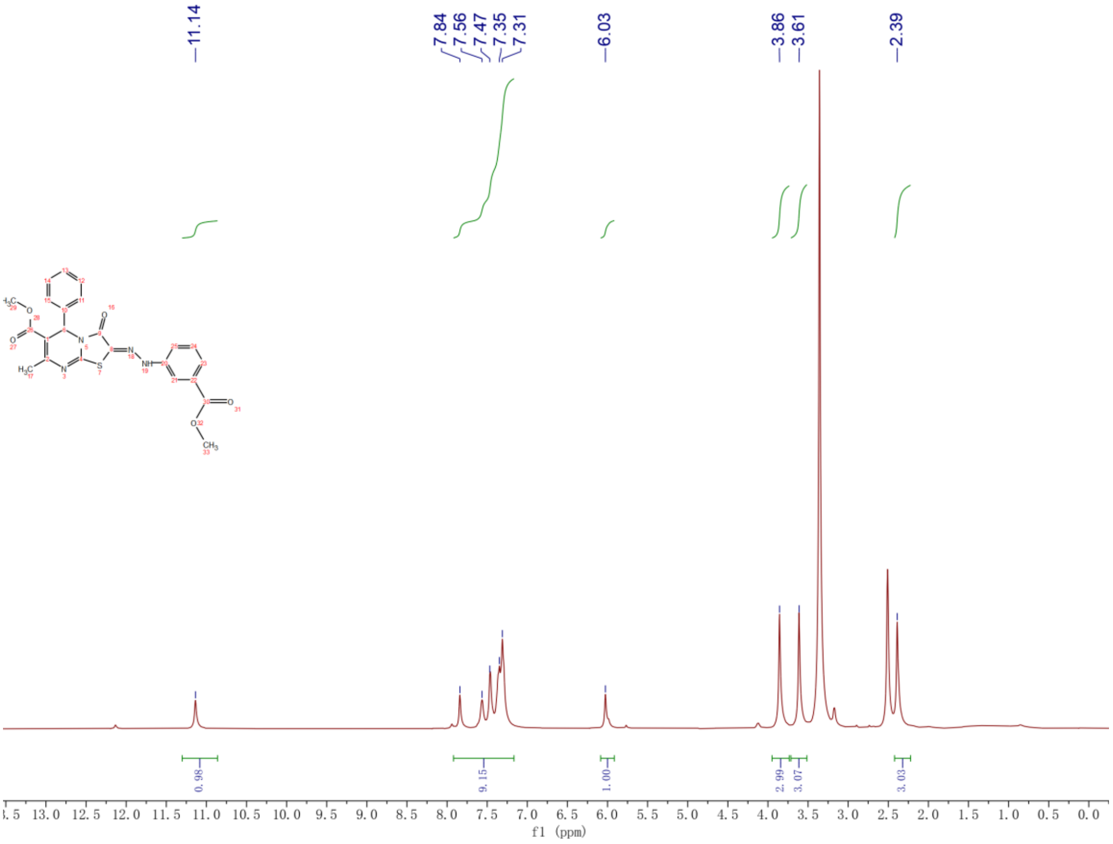

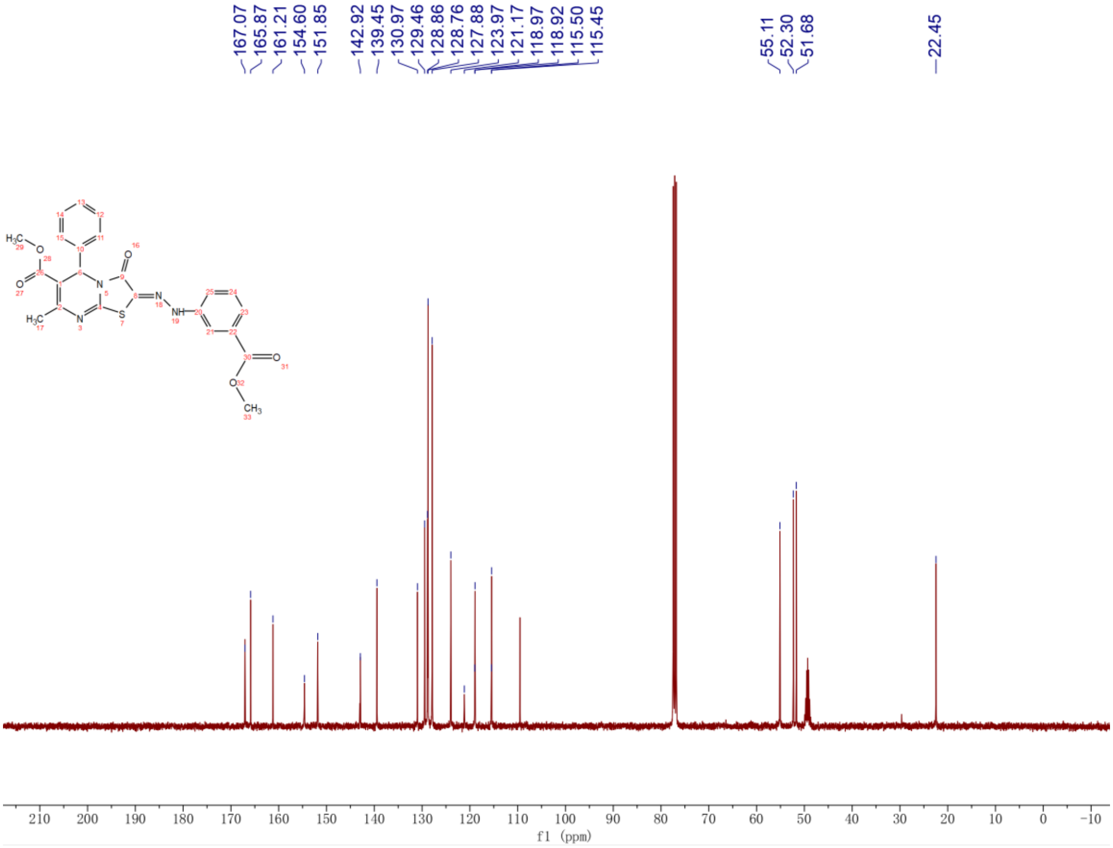

Methyl 2-(2-(2-acetylphenyl)hydrazineylidene)-5-(4-methoxyphenyl)-7-methyl-3-oxo-2, 3-dihydro-5H-thiazolo[3, 2-a]pyrimidine-6-carboxylate **(12a)**.

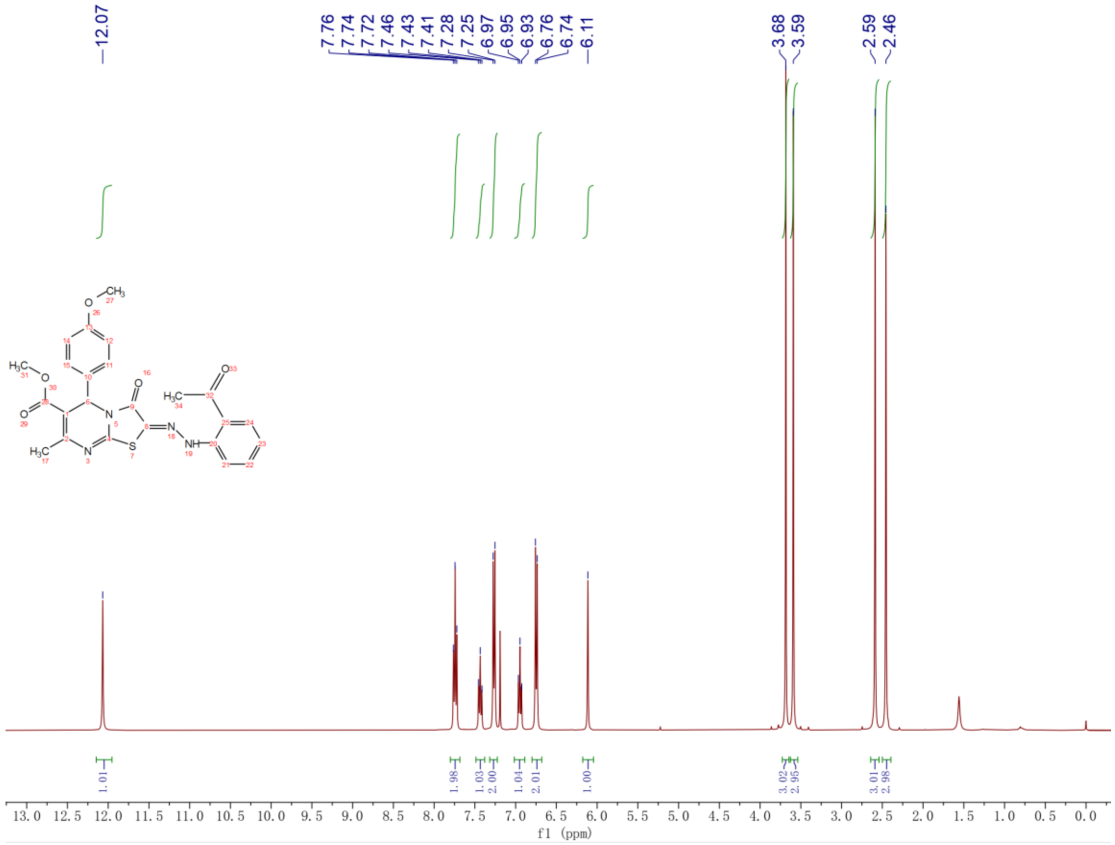

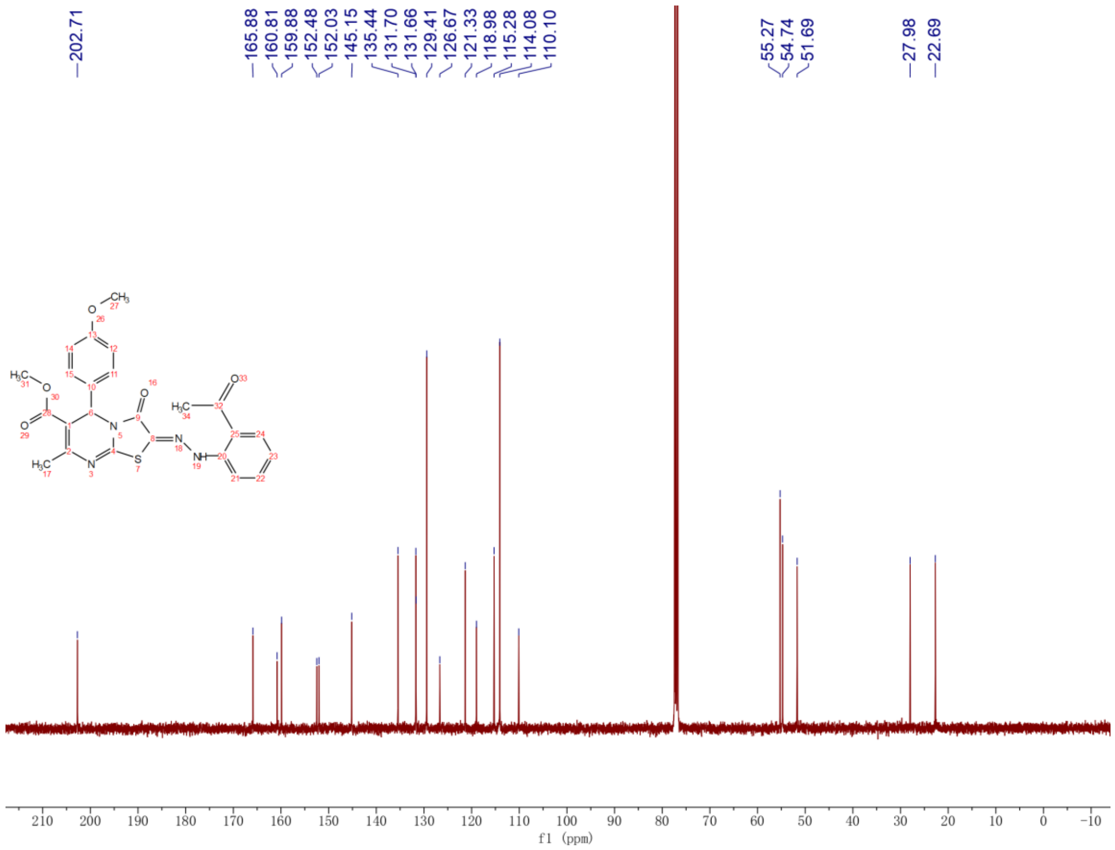

Methyl 2-(2-(2-(methoxycarbonyl)phenyl)hydrazineylidene)-5-(4-methoxyphenyl)-7-methyl-3-oxo-2, 3-dihydro-5H-thiazolo[3, 2-a]pyrimidine-6-carboxylate **(12b)**.

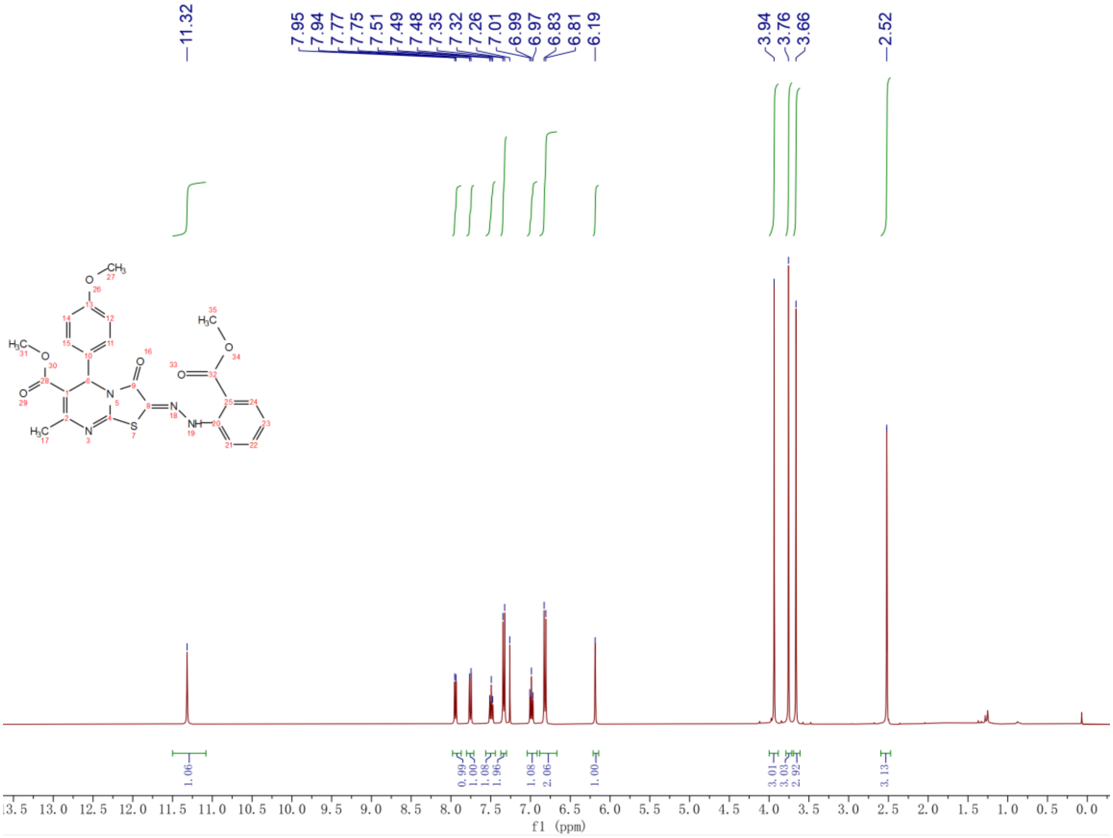

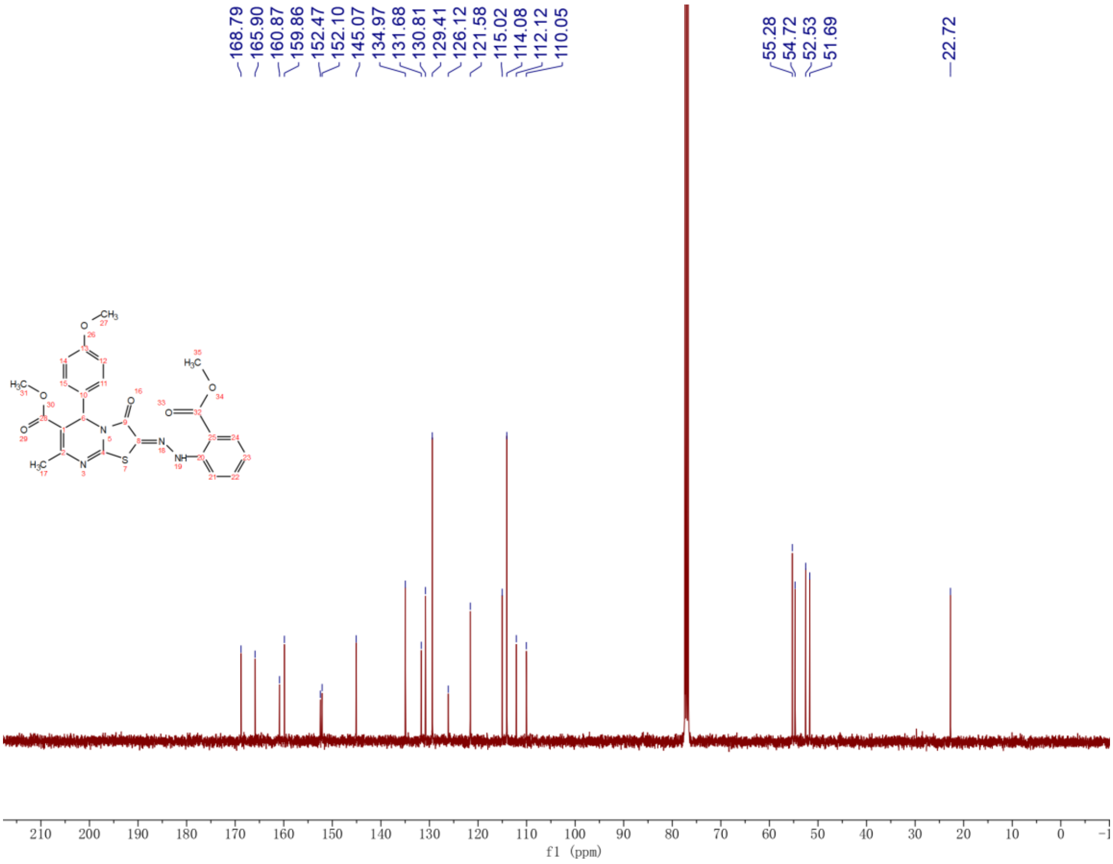

Methyl 2-(2-(3-(methoxycarbonyl)phenyl)hydrazineylidene)-5-(4-methoxyphenyl)-7-methyl-3-oxo-2, 3-dihydro-5H-thiazolo[3, 2-a]pyrimidine-6-carboxylate **(12c)**.

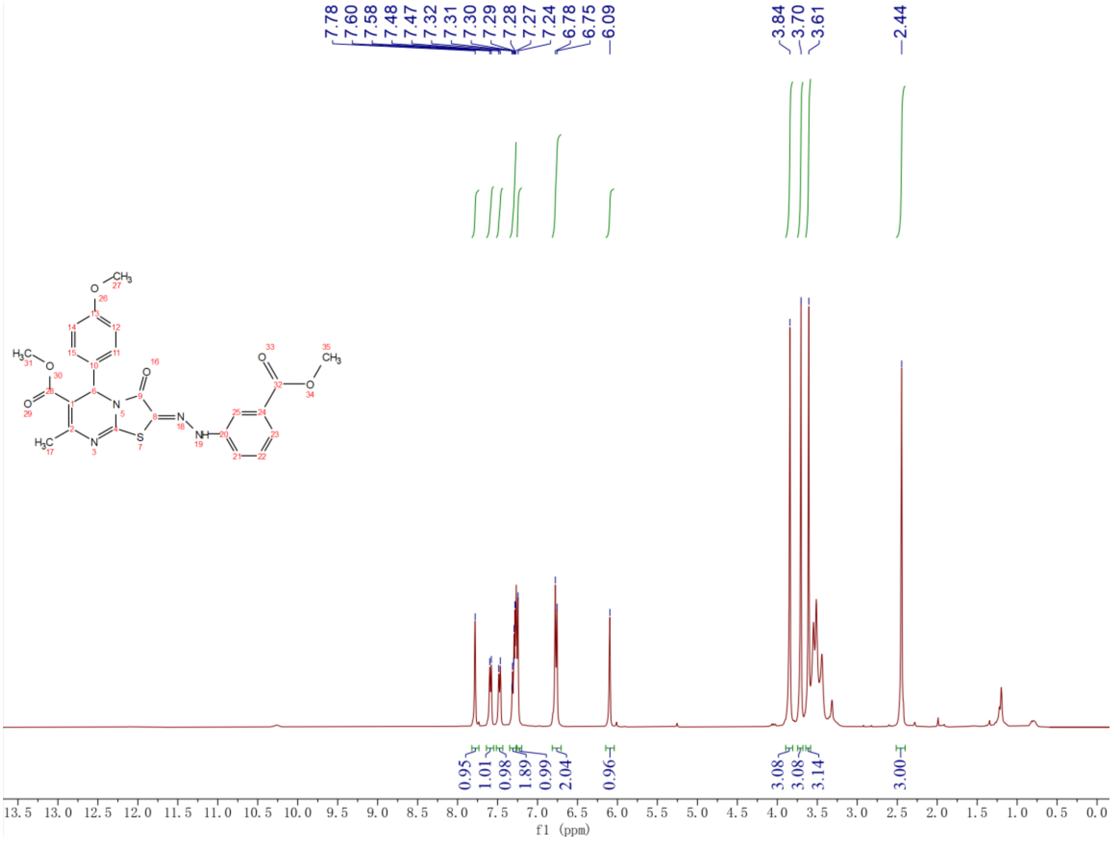

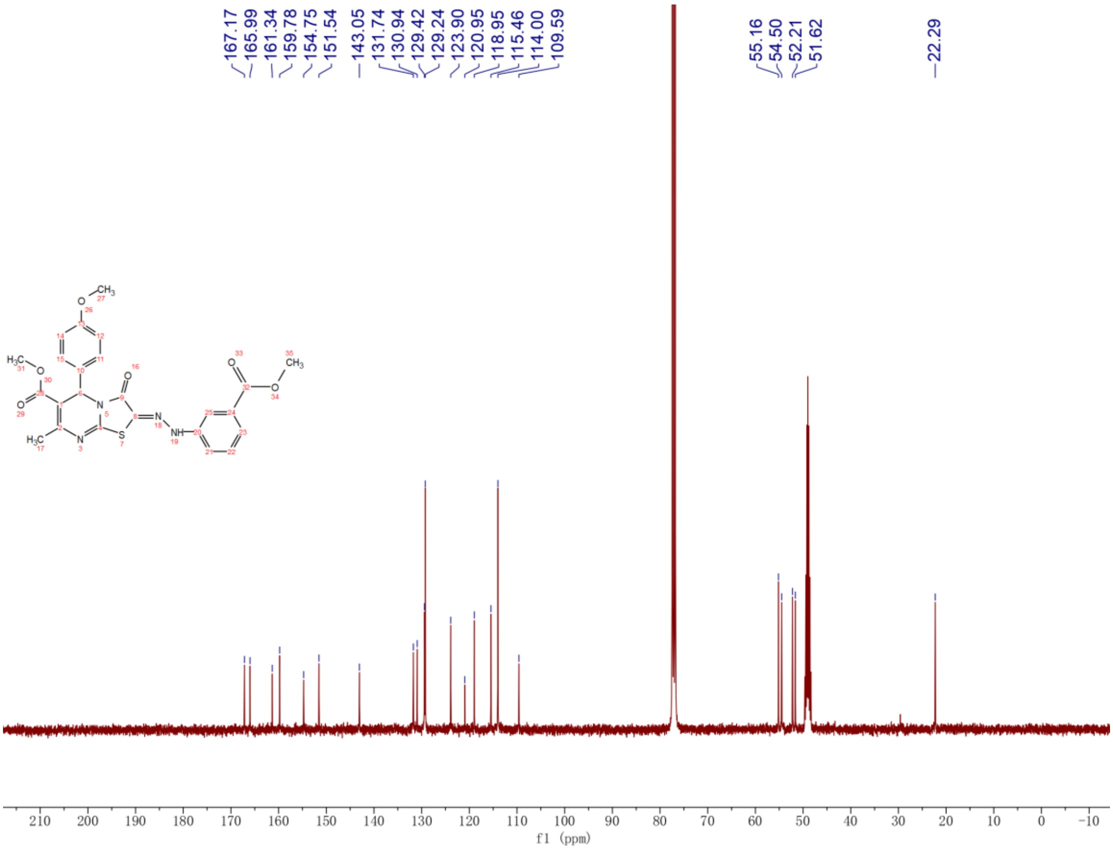

(2-(6-(Methoxycarbonyl)-5-(4-methoxyphenyl)-7-methyl-3-oxo-5H-thiazolo[3, 2-a]pyrimidin-2(3H)-ylidene)hydrazineyl)-3-methylbenzoic acid **(12d)**.

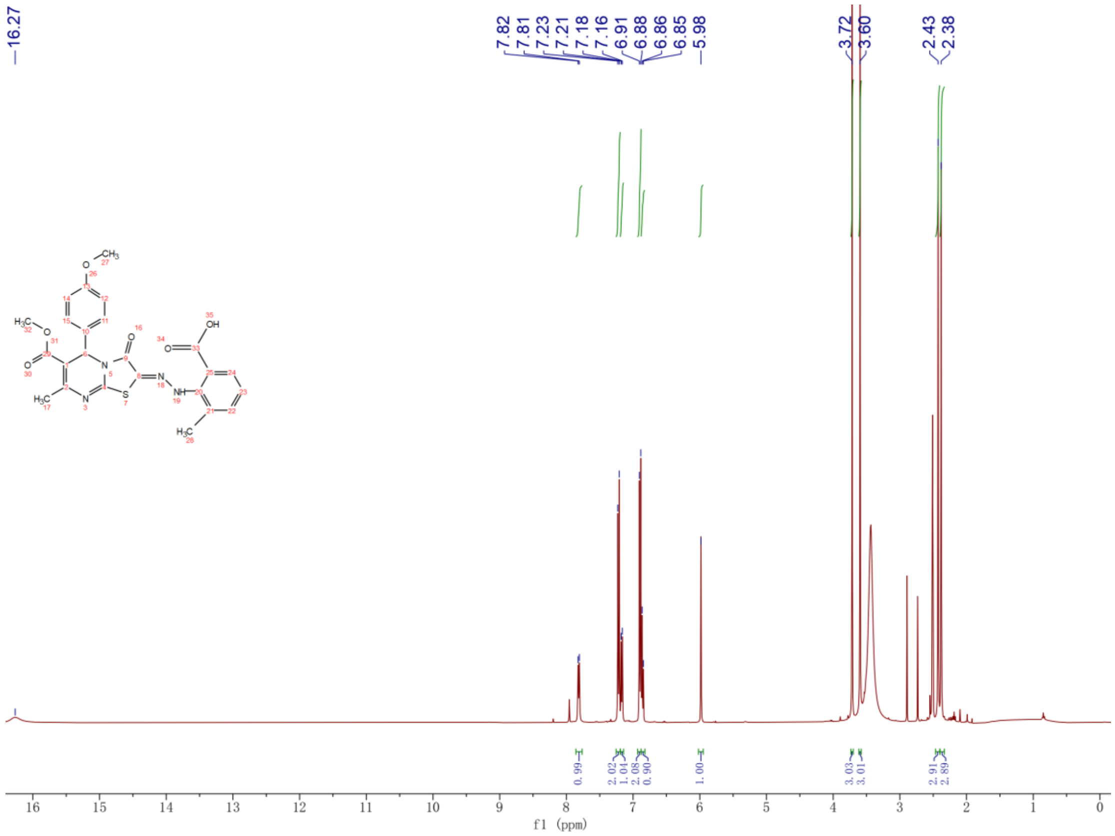

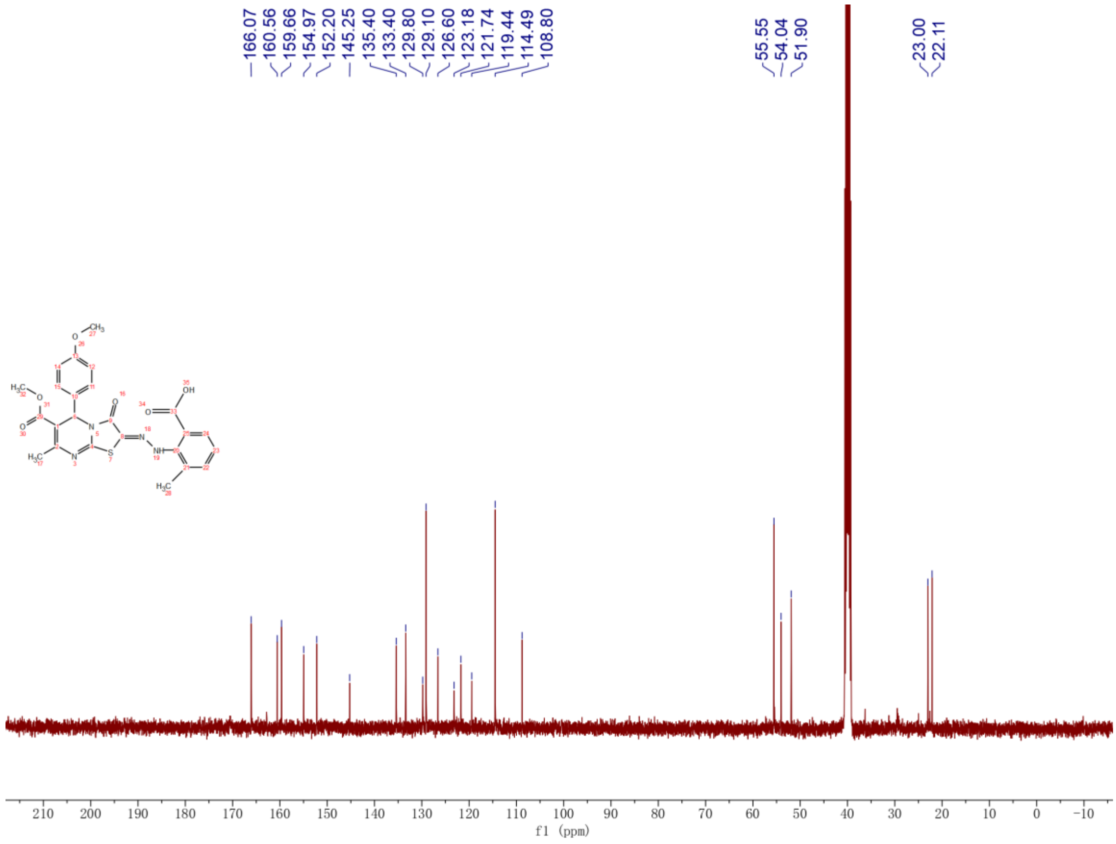

Methyl 5-(4-methoxyphenyl)-7-methyl-3-oxo-2-(2-(3, 4, 5-trimethoxyphenyl)hydrazineylidene)-2, 3-dihydro-5H-thiazolo[3, 2-a]pyrimidine-6-carboxylate **(12e)**.

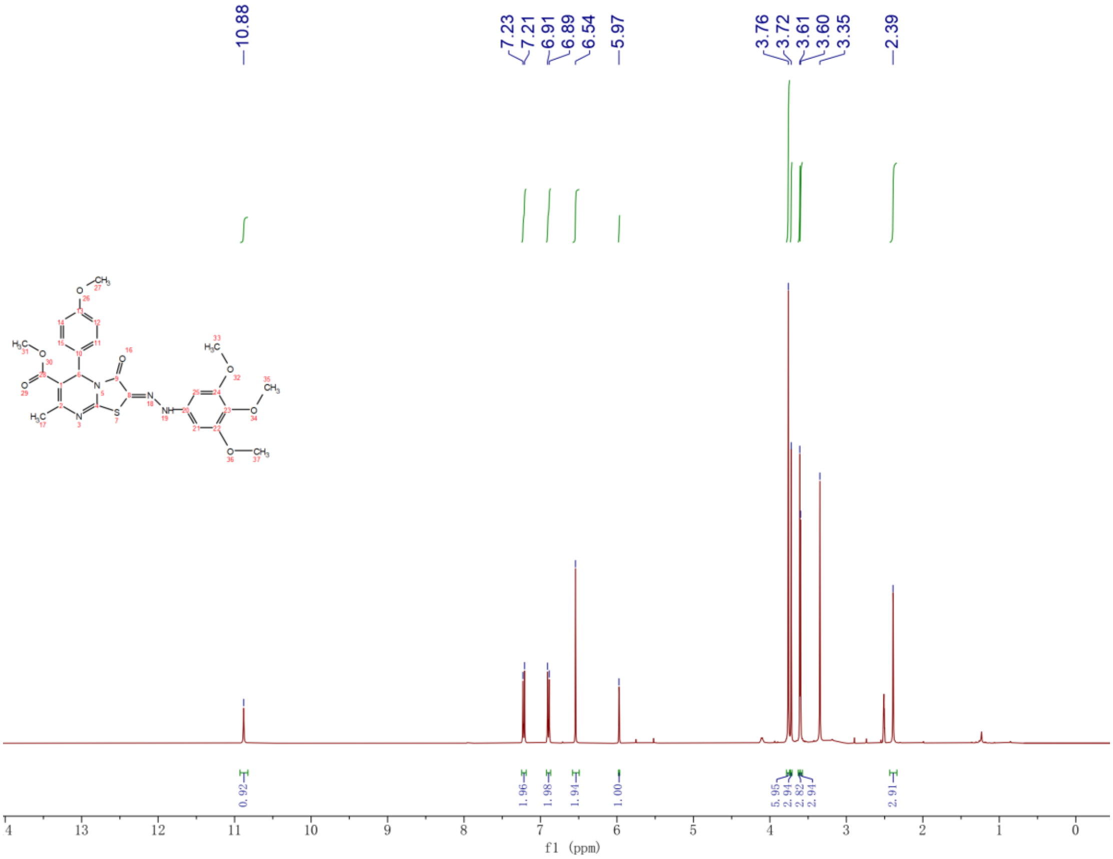

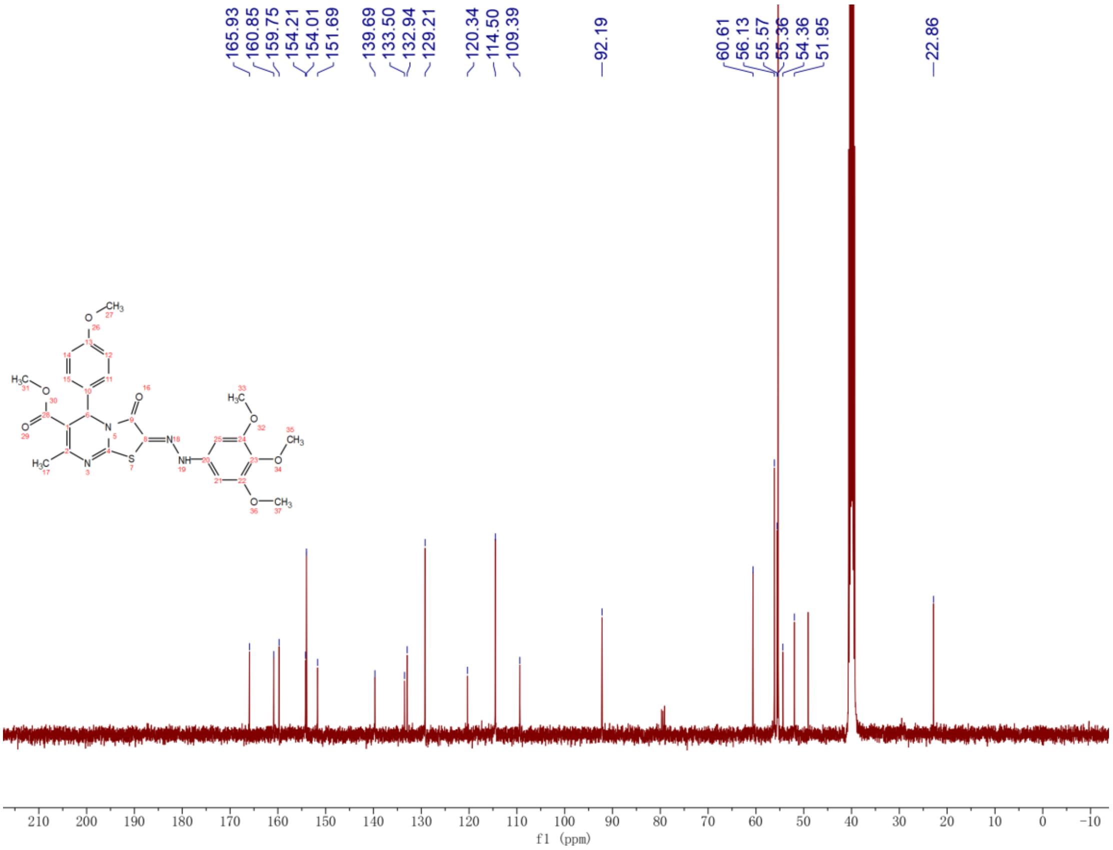

(6-(Methoxycarbonyl)-5-(4-methoxyphenyl)-7-methyl-3-oxo-5H-thiazolo[3, 2-a]pyrimidin-2(3H)-ylidene)hydrazineyl)naphthalene-1-sulfonic acid **(12f)**.

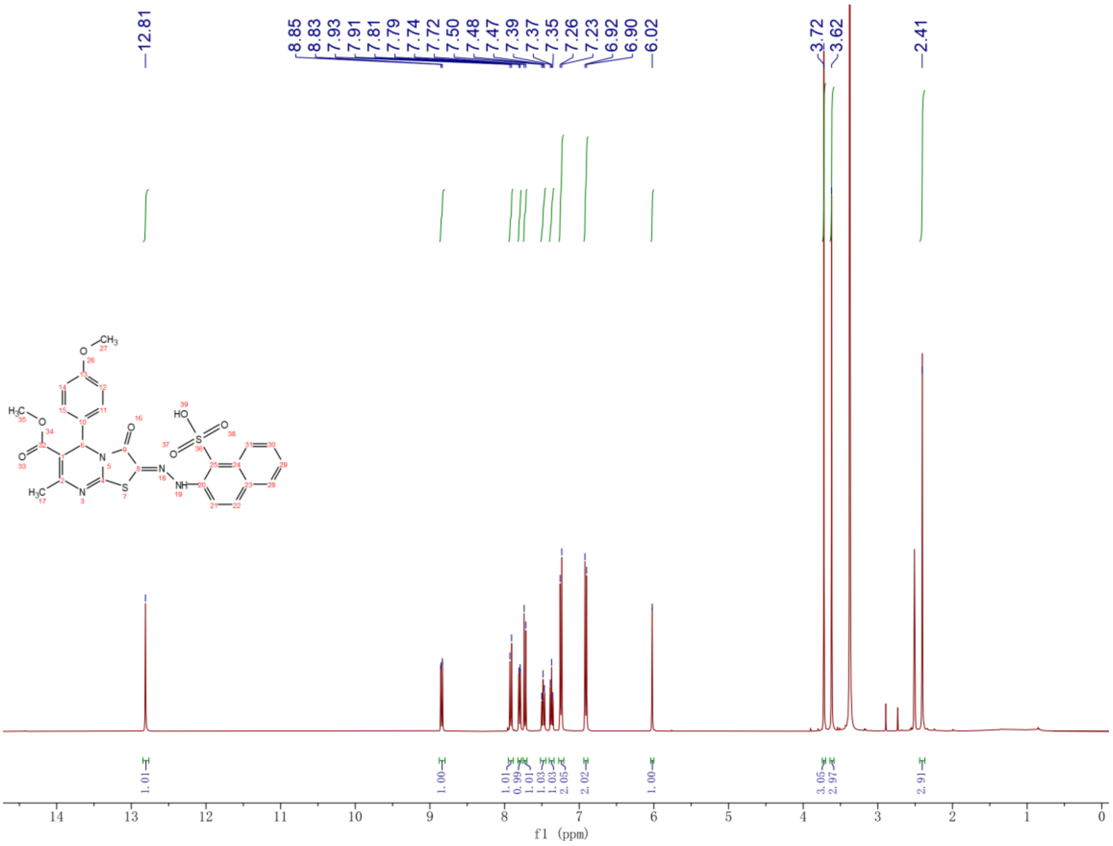

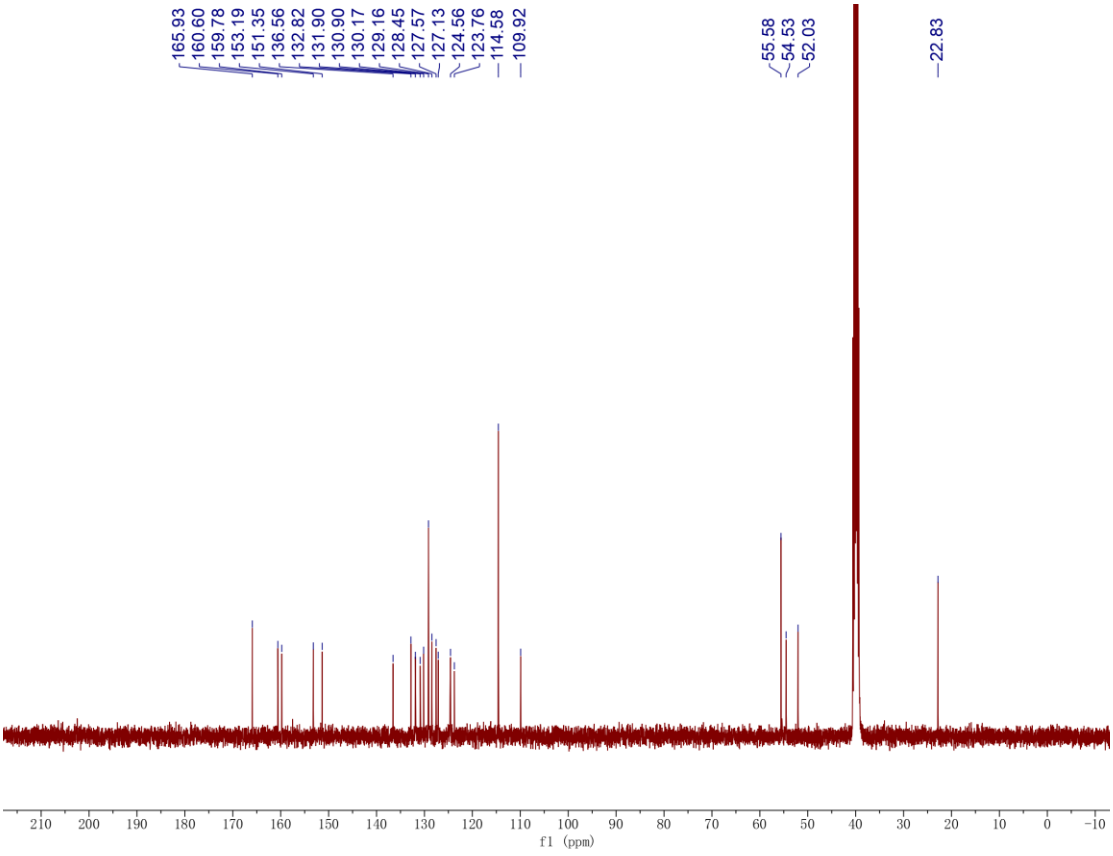

Methyl 2-(2-(2-methoxyphenyl)hydrazineylidene)-7-methyl-3-oxo-5-(2, 3, 4-trimethoxyphenyl)-2, 3-dihydro-5H-thiazolo[3, 2-a]pyrimidine-6-carboxylate **(11a)**.

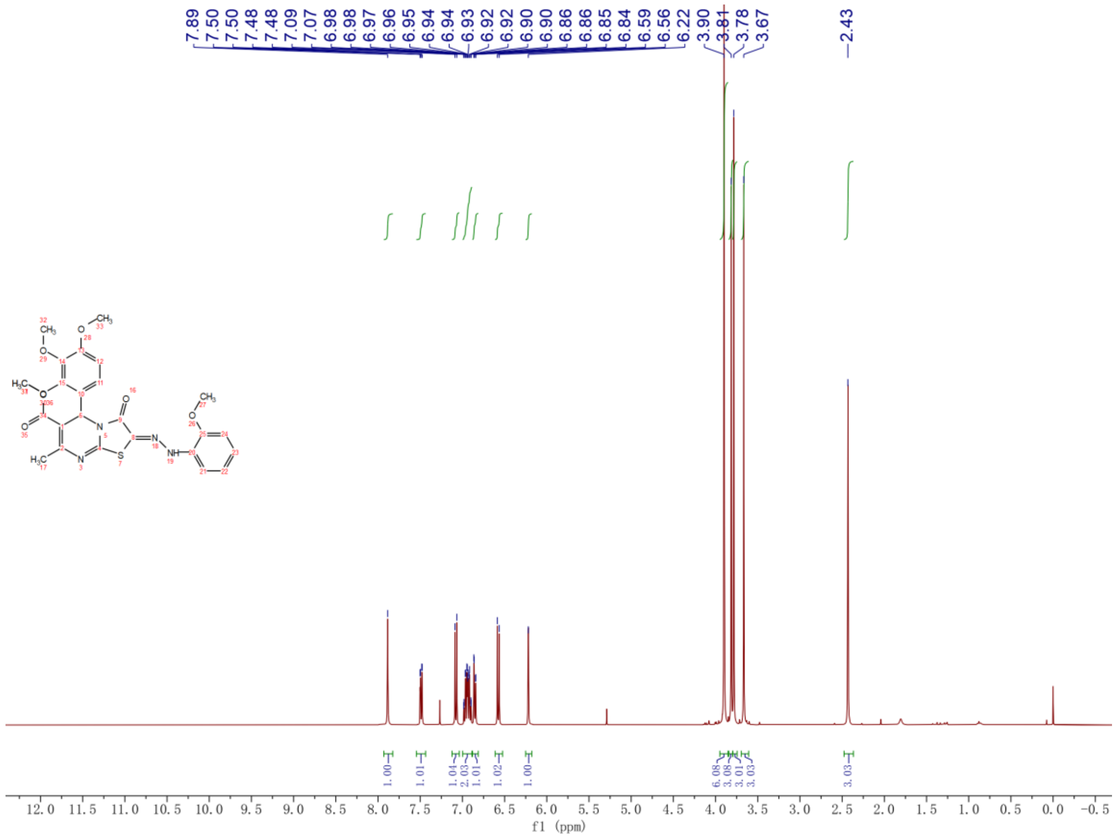

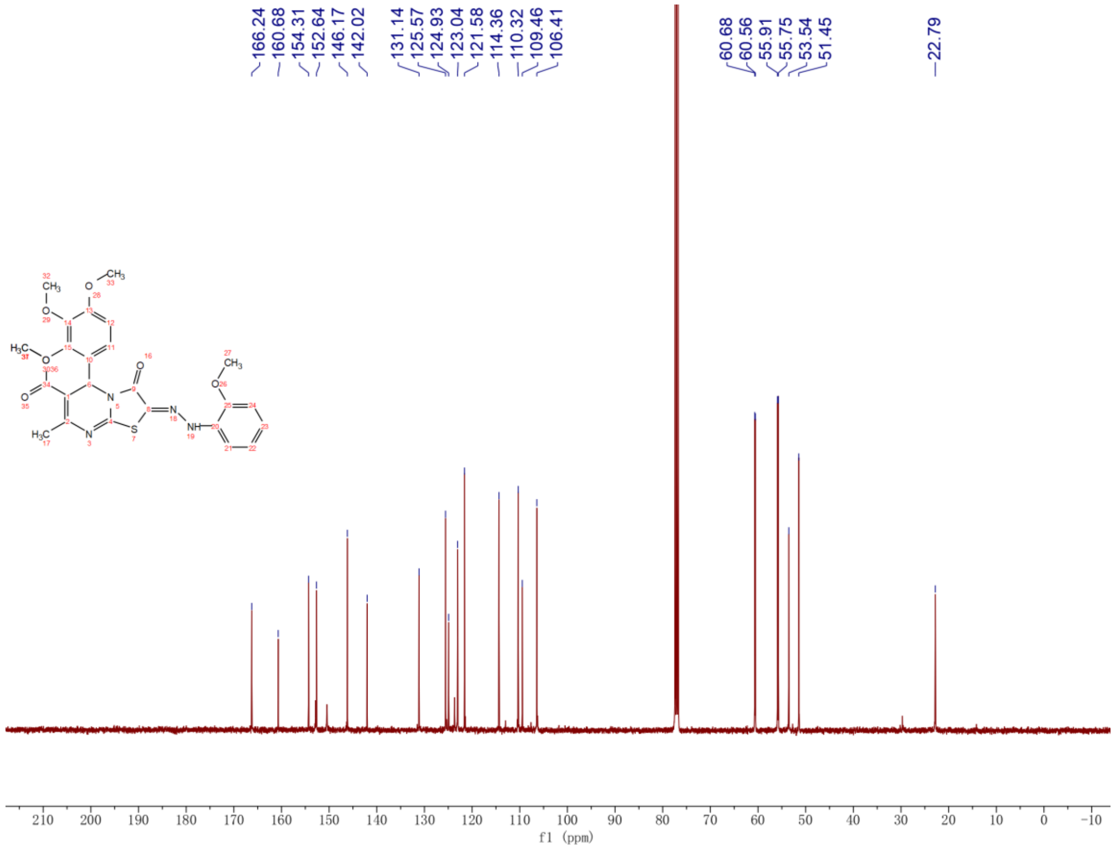

Methyl 7-methyl-3-oxo-2-(2-phenylhydrazineylidene)-5-(2, 3, 4-trimethoxyphenyl)-2, 3-dihydro-5H-thiazolo[3, 2-a]pyrimidine-6-carboxylate **(11b)**.

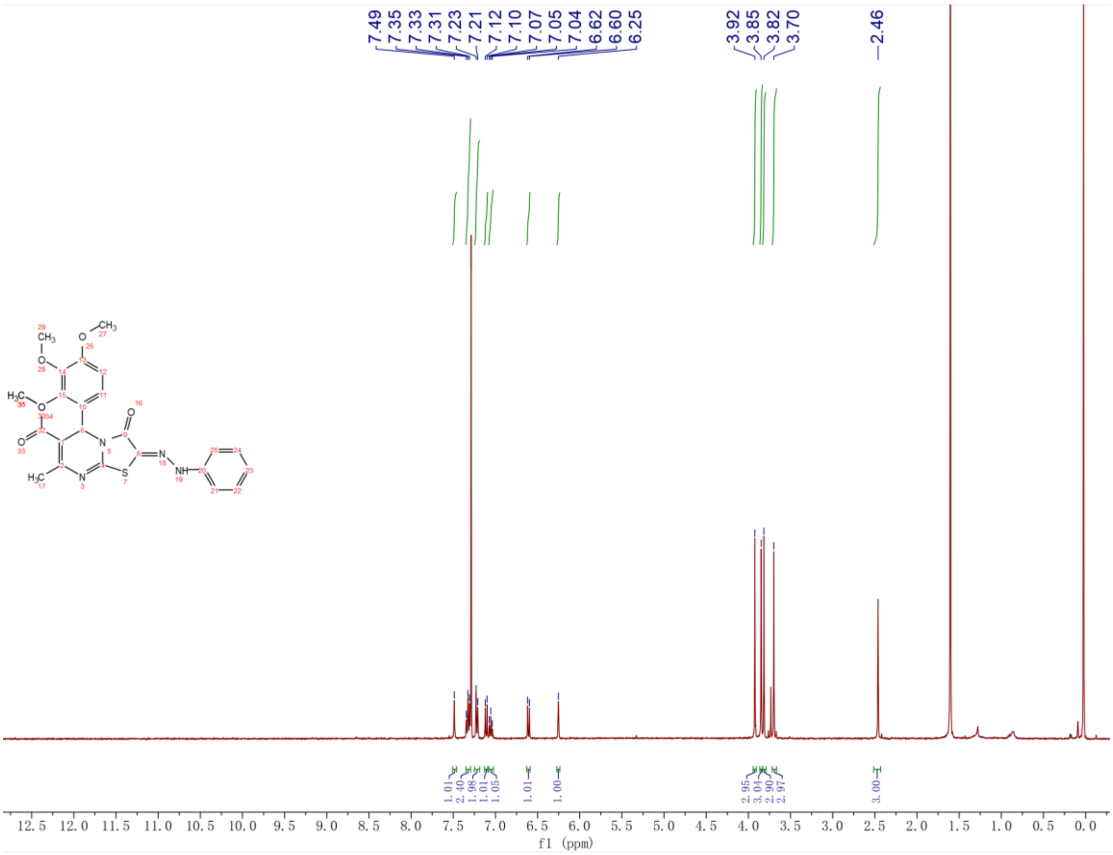

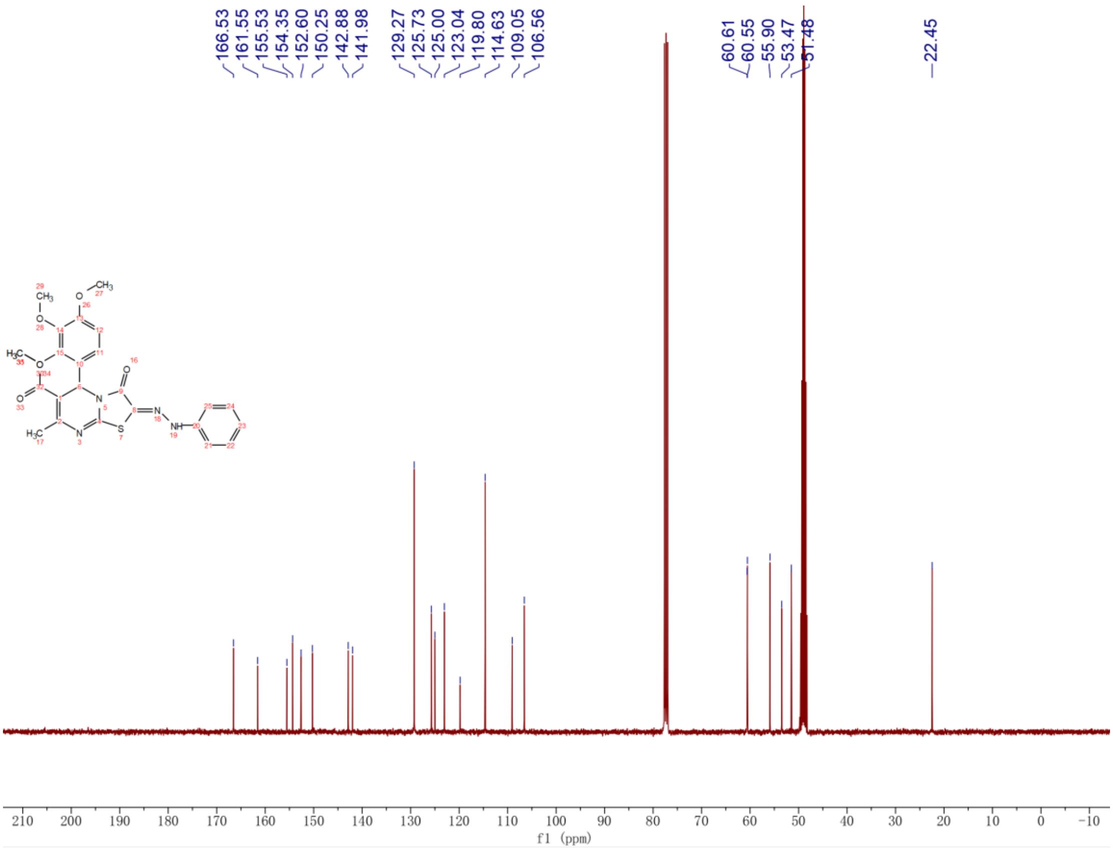

(2-(6-(Methoxycarbonyl)-7-methyl-3-oxo-5-(2, 3, 4-trimethoxyphenyl)-5H-thiazolo[3, 2-a]pyrimidin-2(3H)-ylidene)hydrazineyl)-3-methylbenzoic acid **(11c)**.

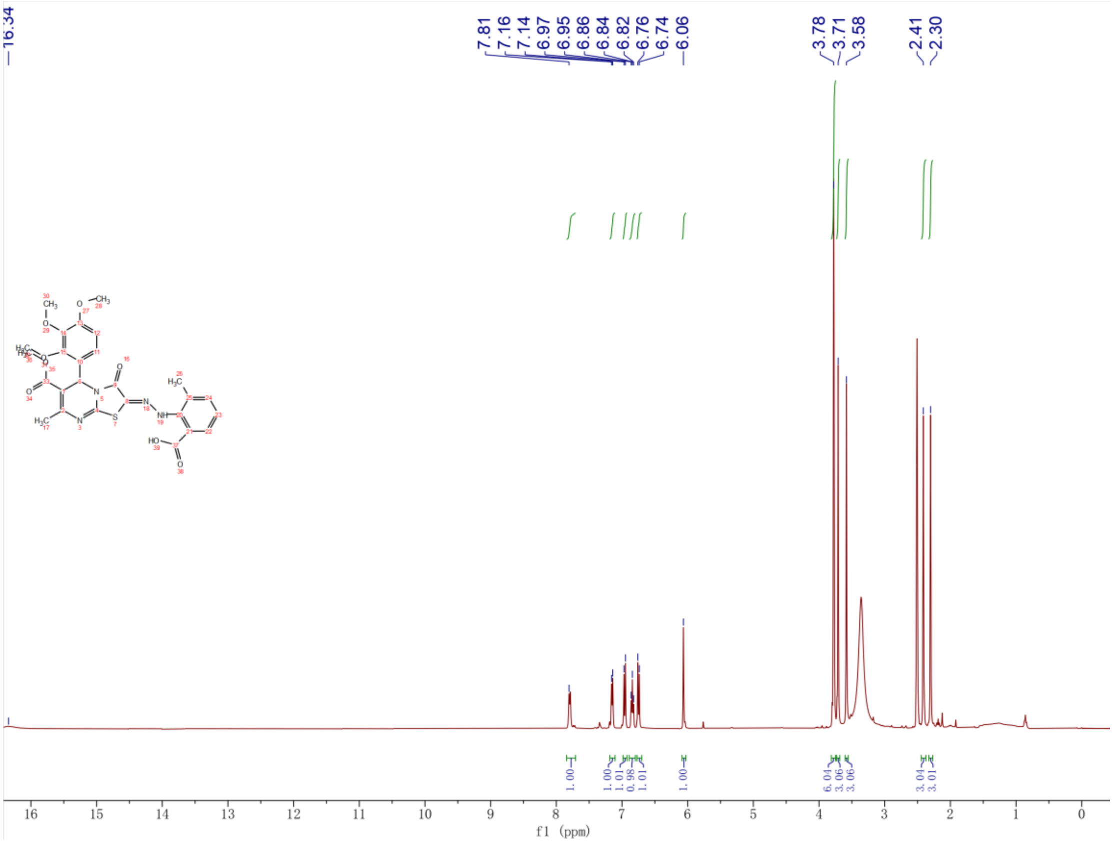

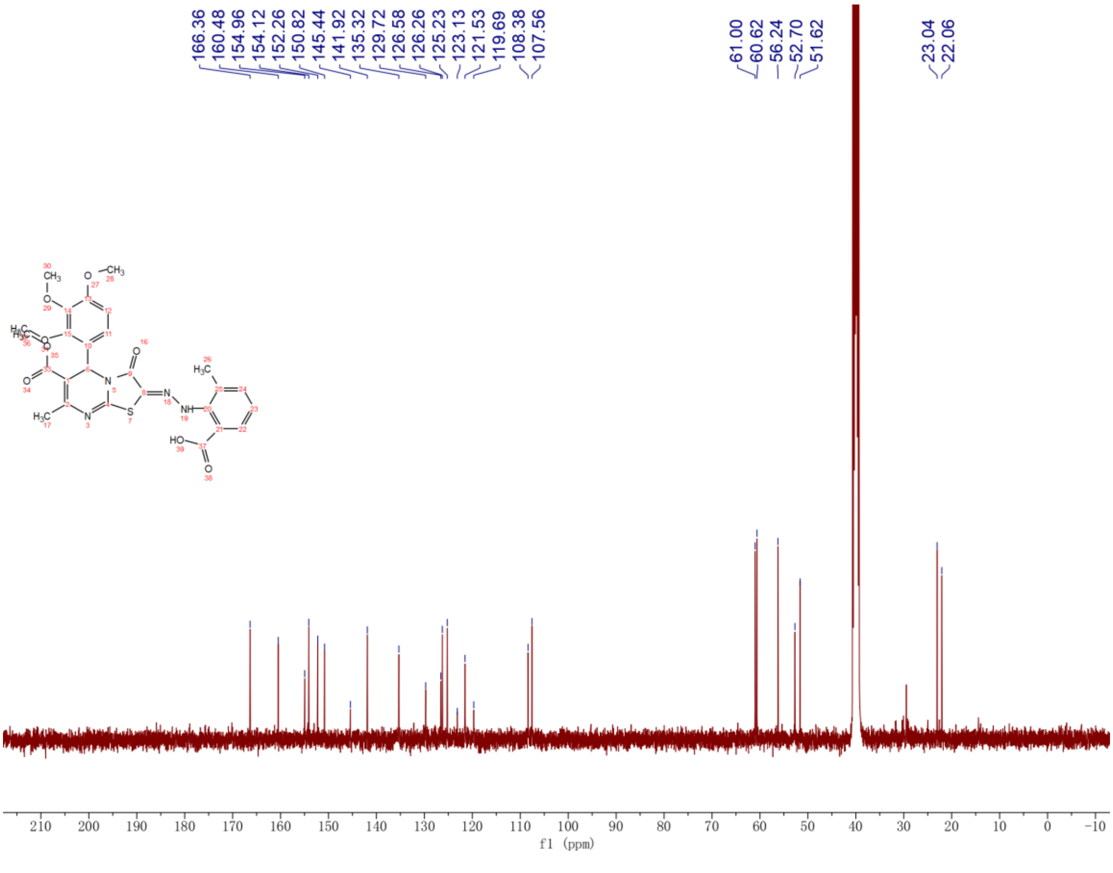

Methyl 7-methyl-2-(2-(3-nitrophenyl)hydrazineylidene)-3-oxo-5-(2, 3, 4-trimethoxyphenyl)-2, 3-dihydro-5H-thiazolo[3, 2-a]pyrimidine-6-carboxylate **(11d)**.

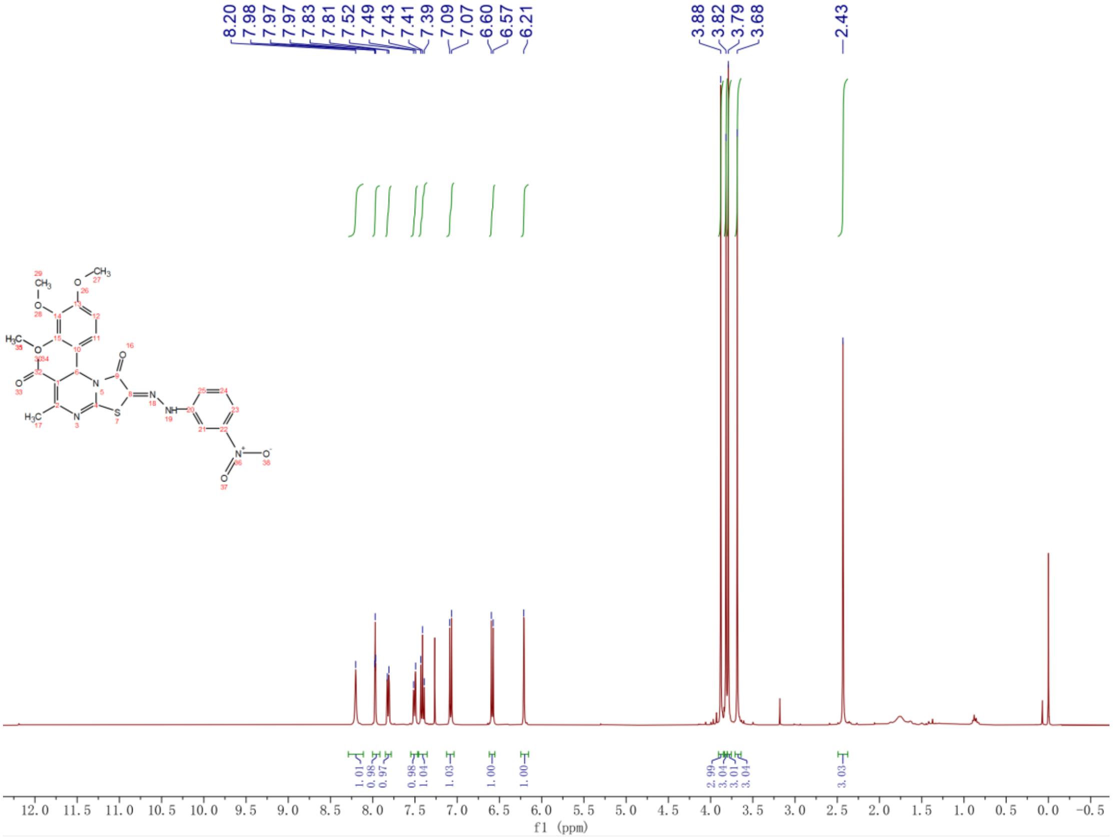

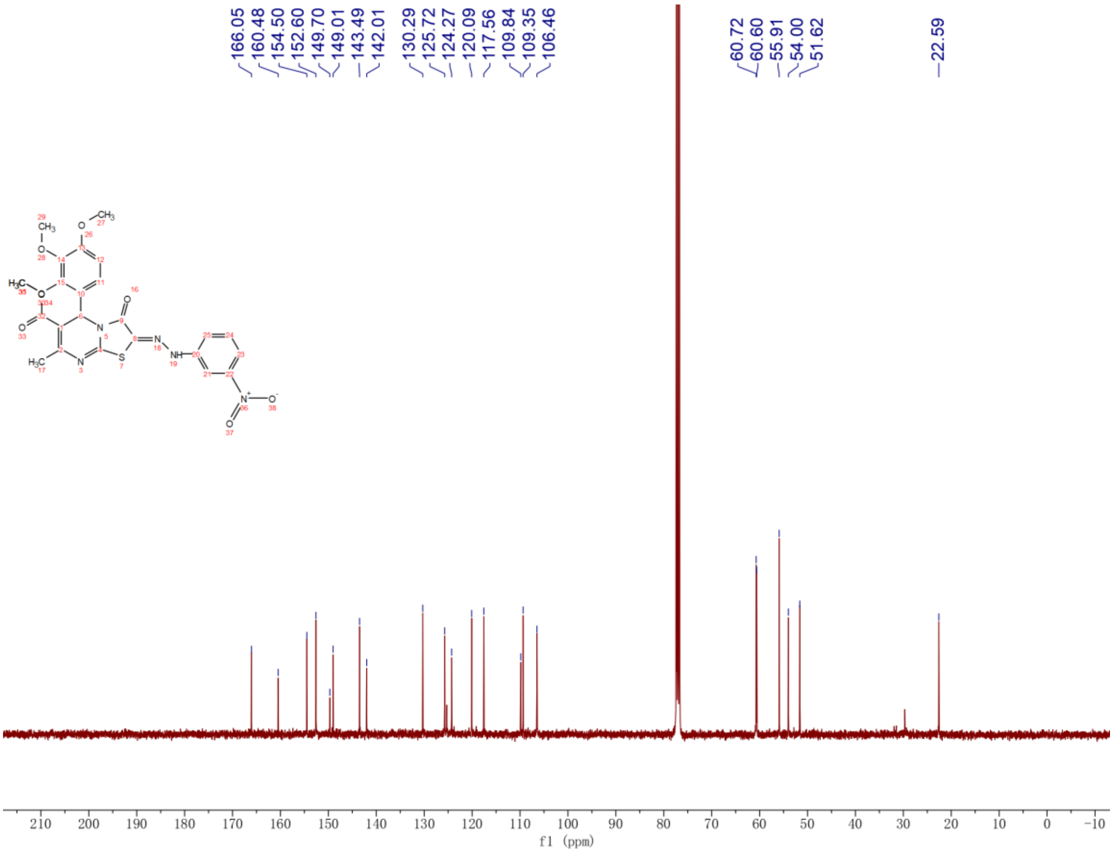

2-(2-(6-(Methoxycarbonyl)-7-methyl-3-oxo-5-(2, 3, 4-trimethoxyphenyl)-5H-thiazolo[3, 2-a]pyrimidin-2(3H)-ylidene)hydrazineyl)benzenesulfonic acid **(11e)**.

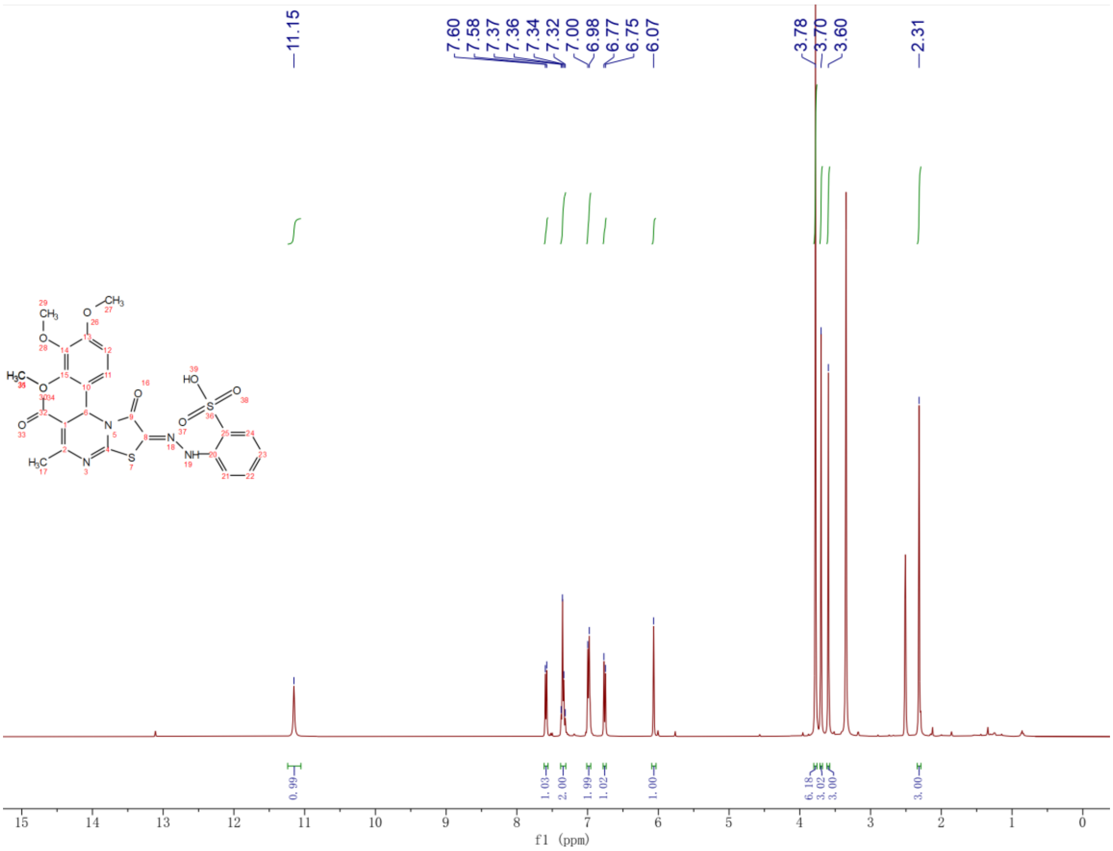

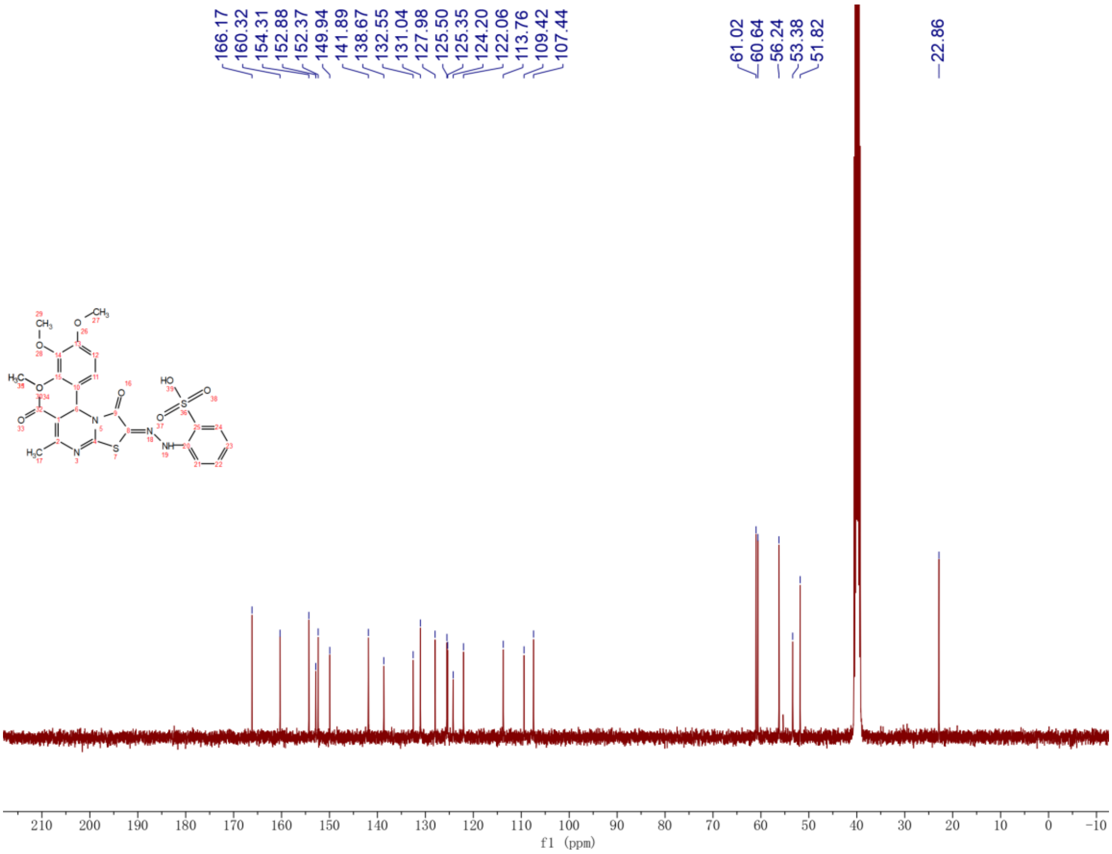

5-Methoxy-2-(2-(6-(methoxycarbonyl)-7-methyl-3-oxo-5-(2,3,4-trimethoxyphenyl)-5H-thiazolo[3,2-a]pyrimidin-2(3H)-ylidene)hydrazineyl)benzenesulfonic acid **(11f)**.

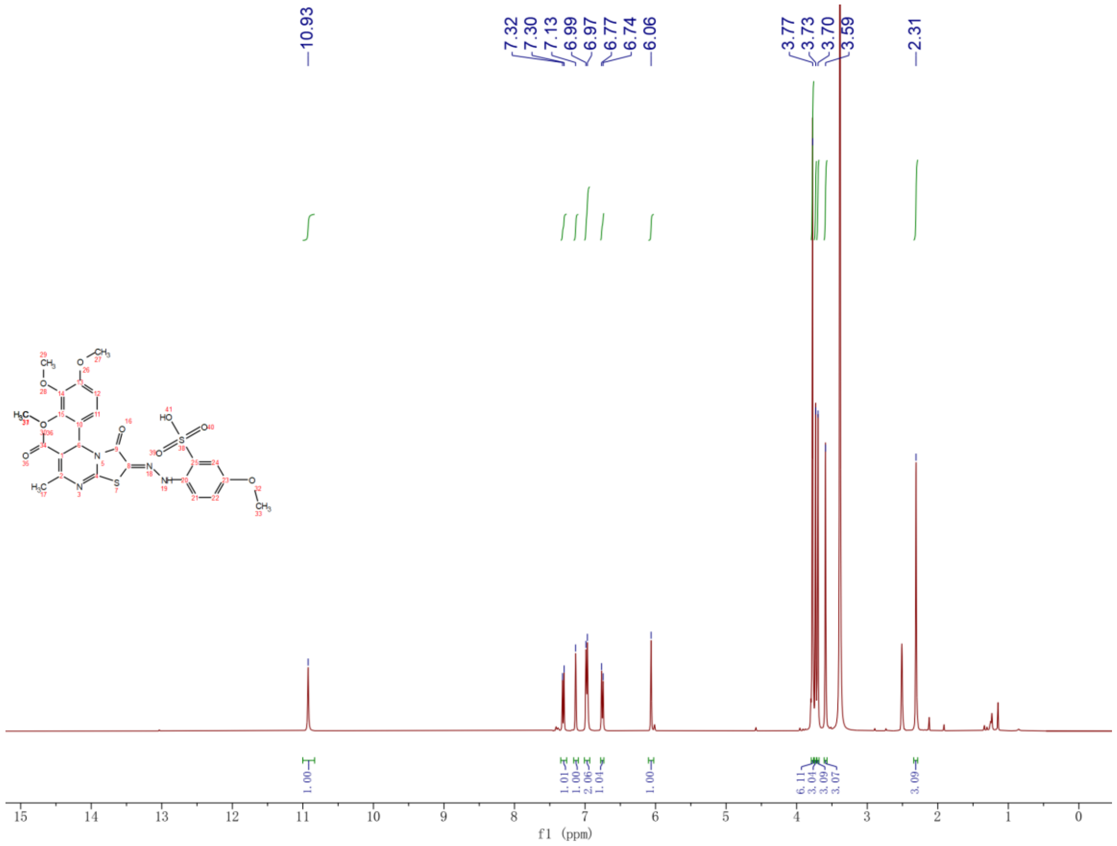

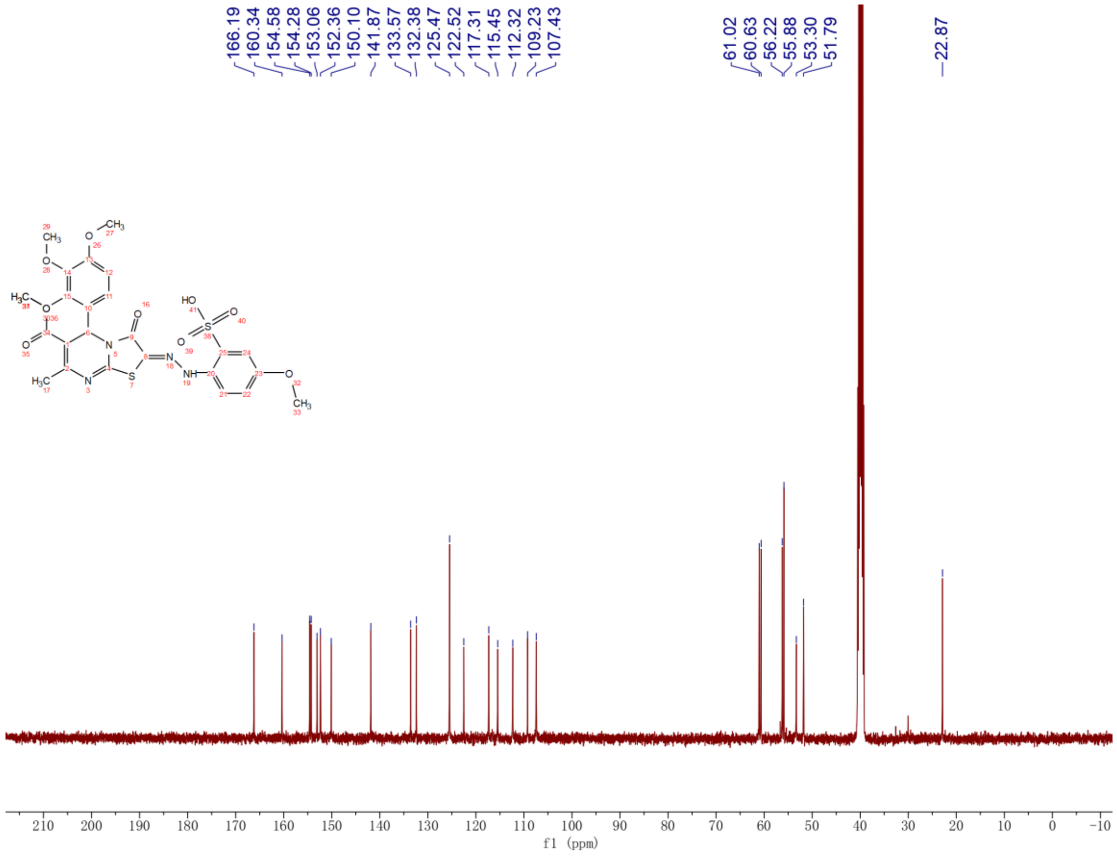

(2-(6-(Methoxycarbonyl)-7-methyl-3-oxo-5-(2, 3, 4-trimethoxyphenyl)-5H-thiazolo[3, 2-a]pyrimidin-2(3H)-ylidene)hydrazineyl)naphthalene-1-sulfonic acid **(11g)**.

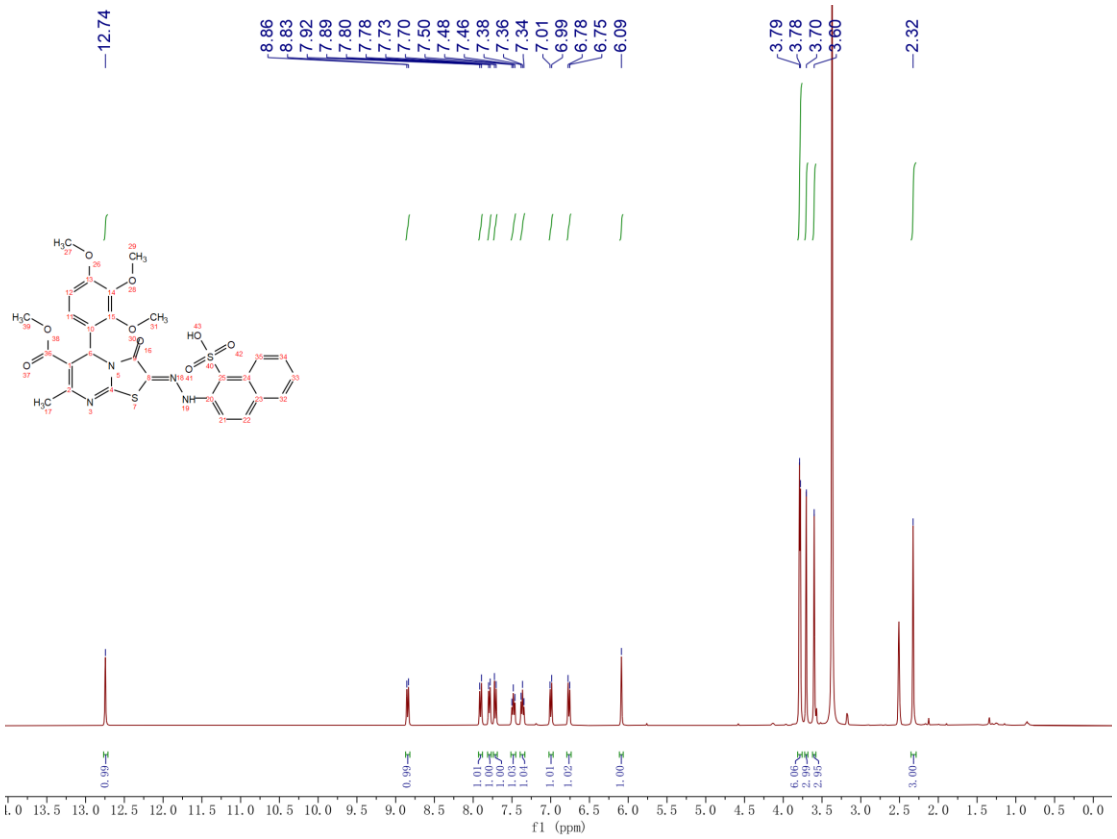

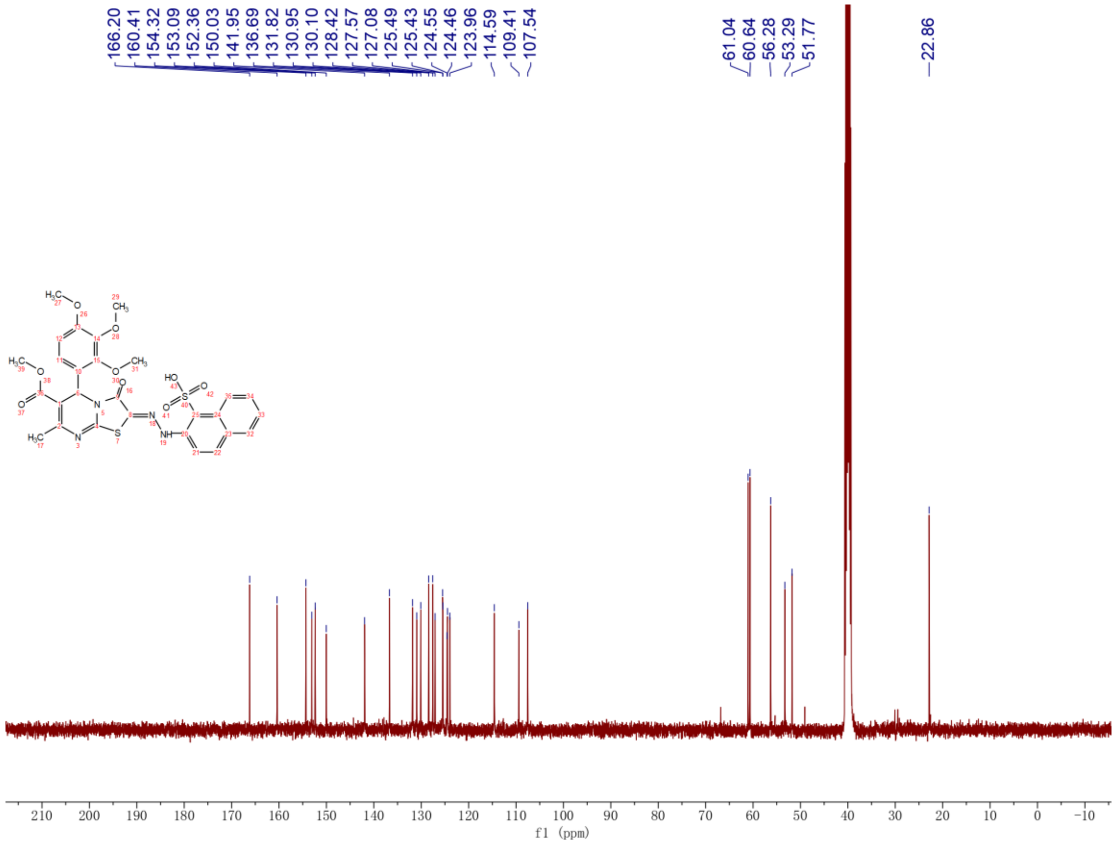

Methyl 2-(2-(2-(methoxycarbonyl)phenyl)hydrazineylidene)-7-methyl-3-oxo-5-(2, 3, 4-trimethoxyphenyl)-2, 3-dihydro-5H-thiazolo[3, 2-a]pyrimidine-6-carboxylate **(11h)**.

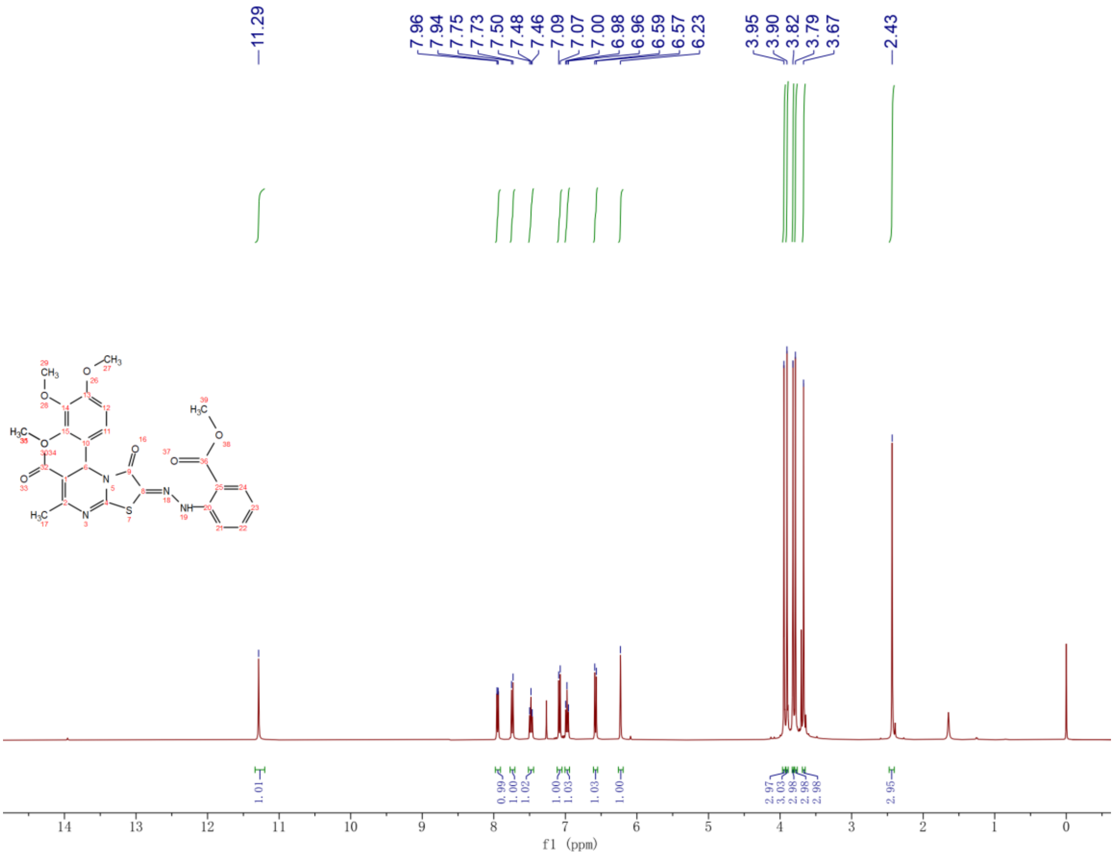

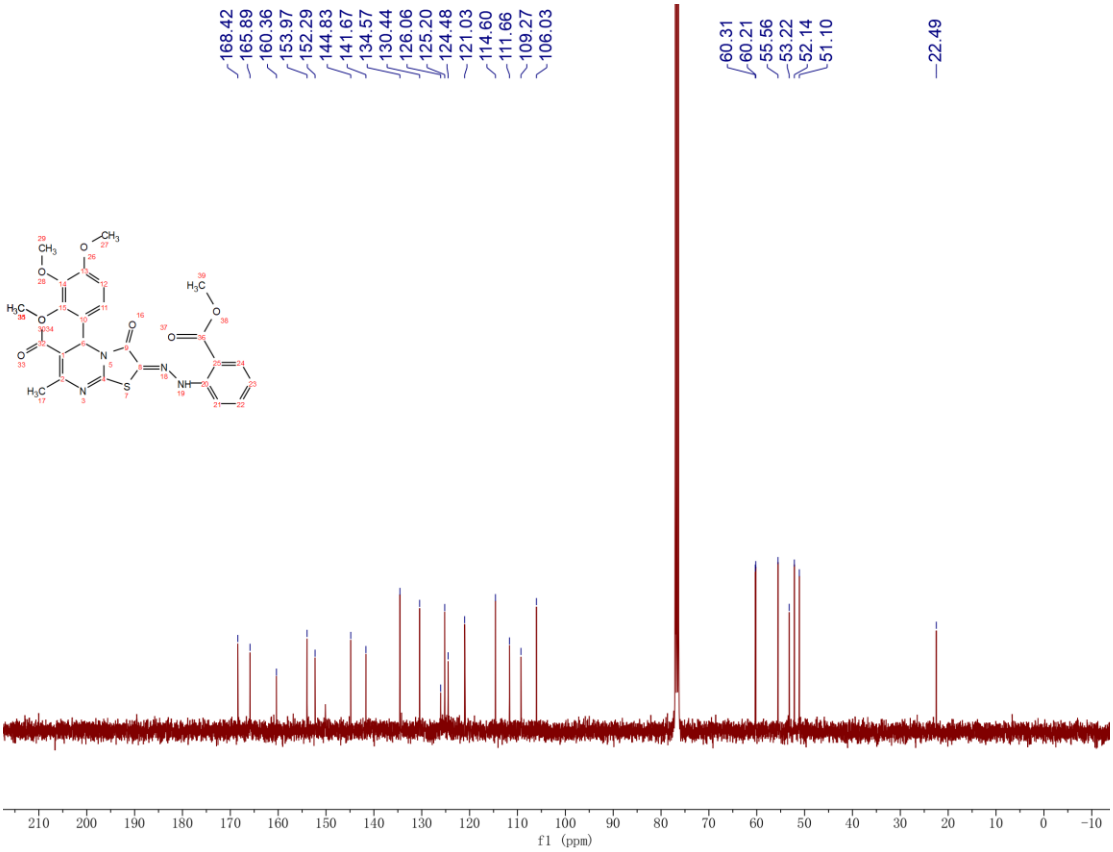

Methyl 2-(2-(2-acetylphenyl)hydrazineylidene)-7-methyl-3-oxo-5-(2, 3, 4-trimethoxyphenyl)-2, 3-dihydro-5H-thiazolo[3, 2-a]pyrimidine-6-carboxylate **(11i)**.

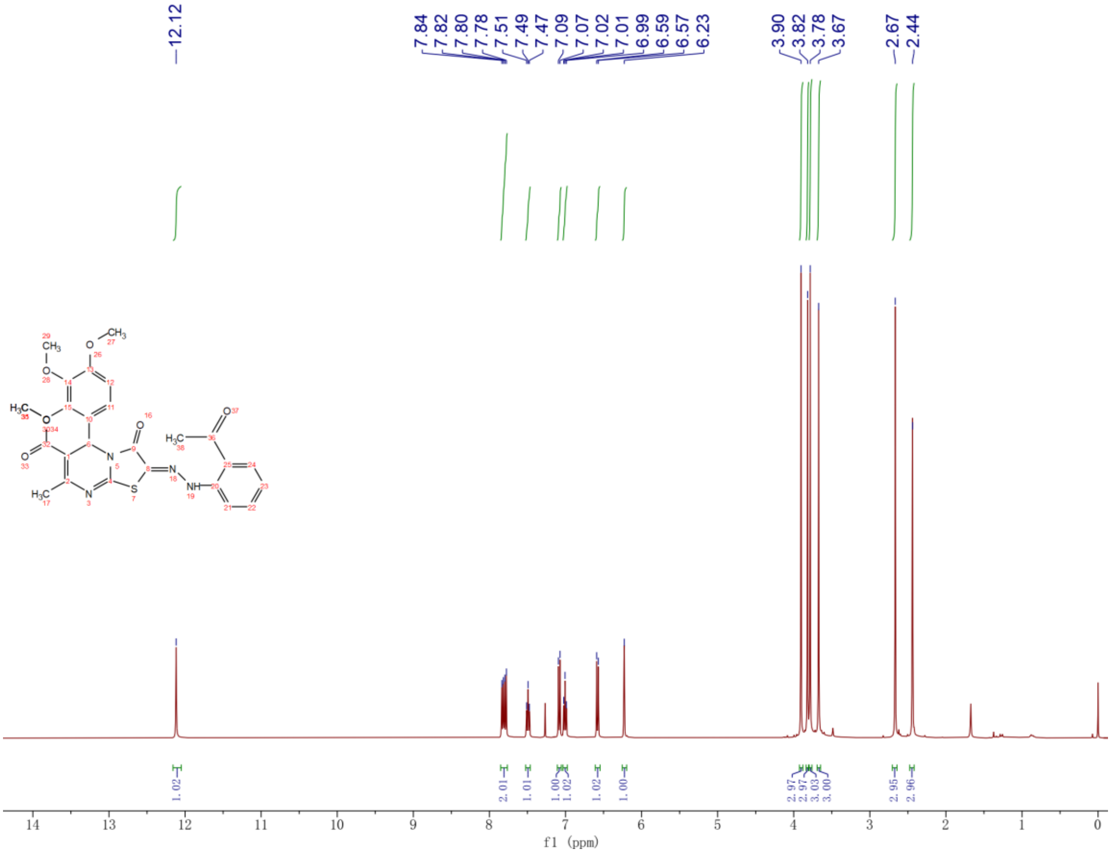

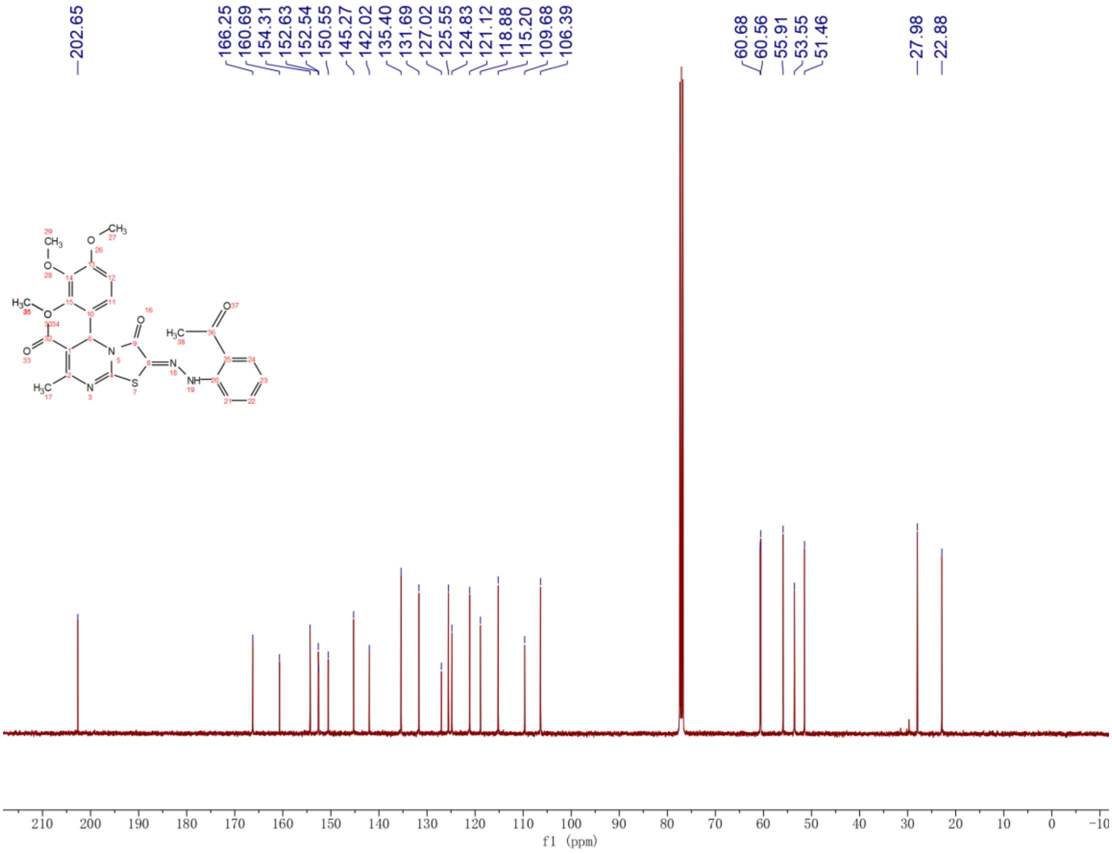

Methyl 2-(2-(3-(methoxycarbonyl)phenyl)hydrazineylidene)-7-methyl-3-oxo-5-(2, 3, 4-trimethoxyphenyl)-2, 3-dihydro-5H-thiazolo[3, 2-a]pyrimidine-6-carboxylate **(11j)**.

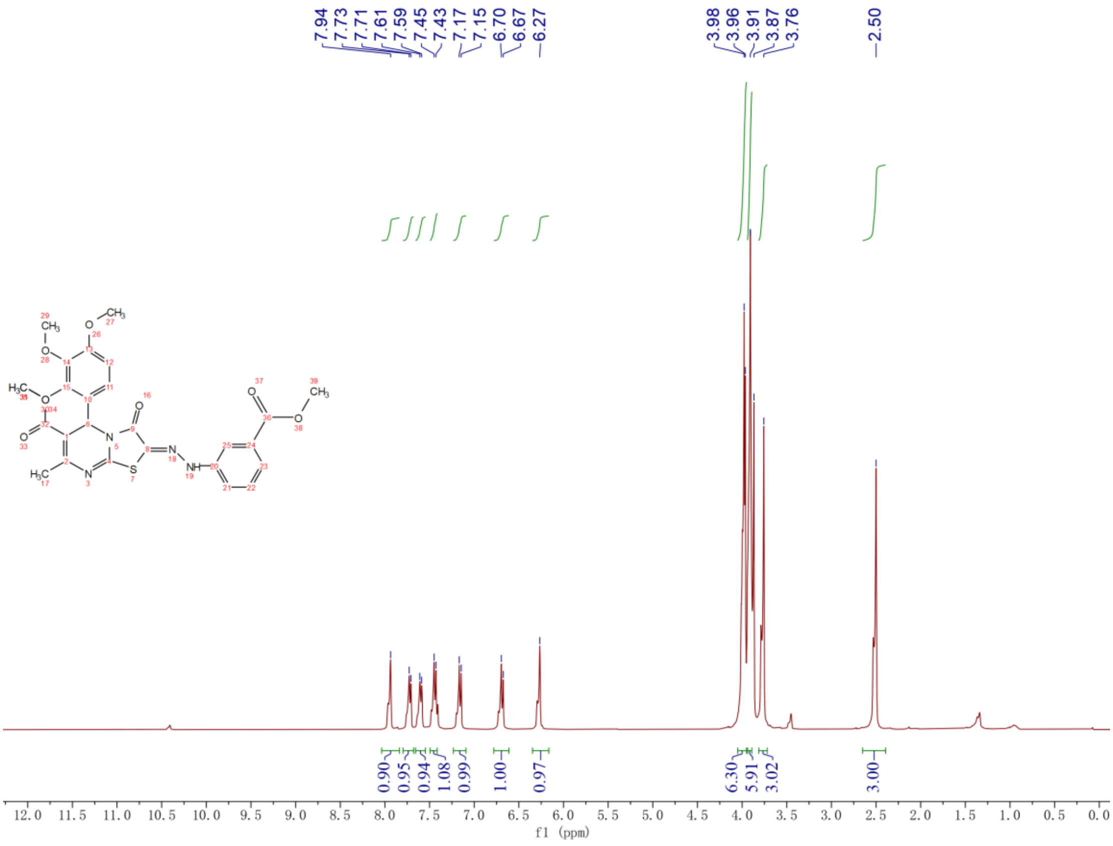

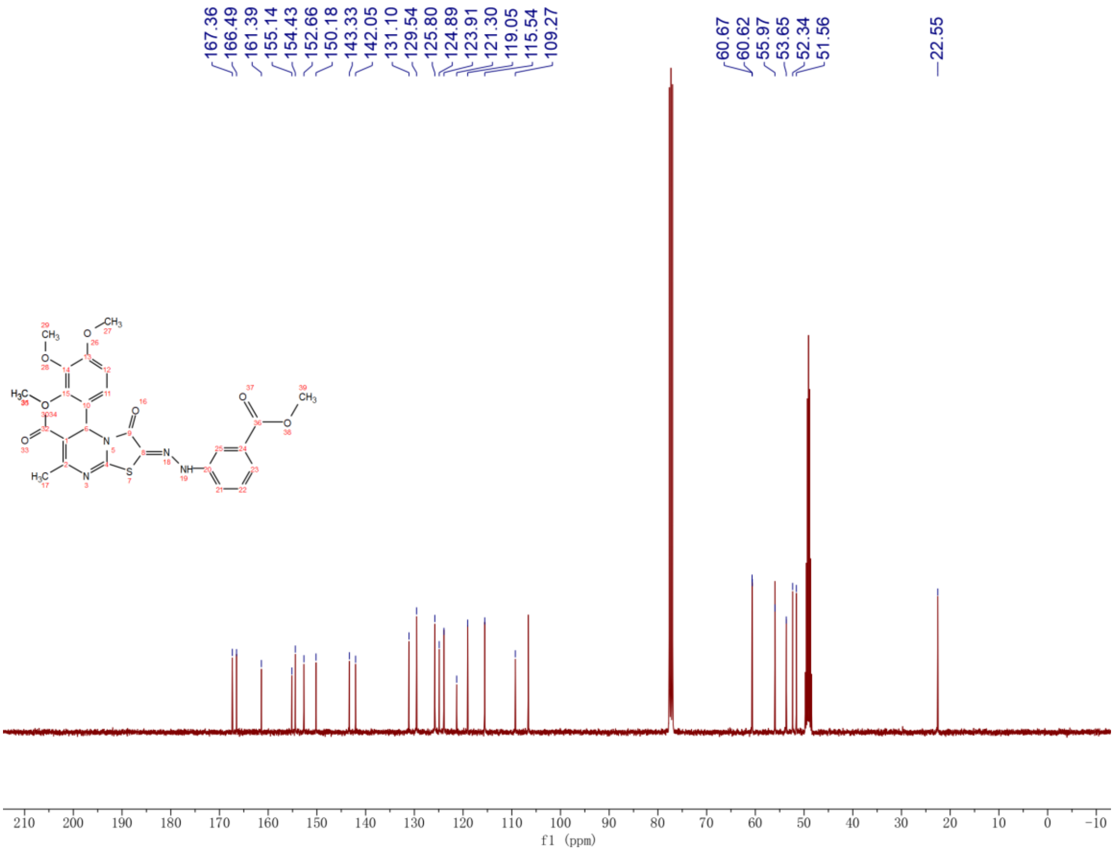

Methyl 2-(2-(3-acetylphenyl)hydrazineylidene)-7-methyl-3-oxo-5-(2, 3, 4-trimethoxyphenyl)-2, 3-dihydro-5H-thiazolo[3, 2-a]pyrimidine-6-carboxylate **(11k)**.

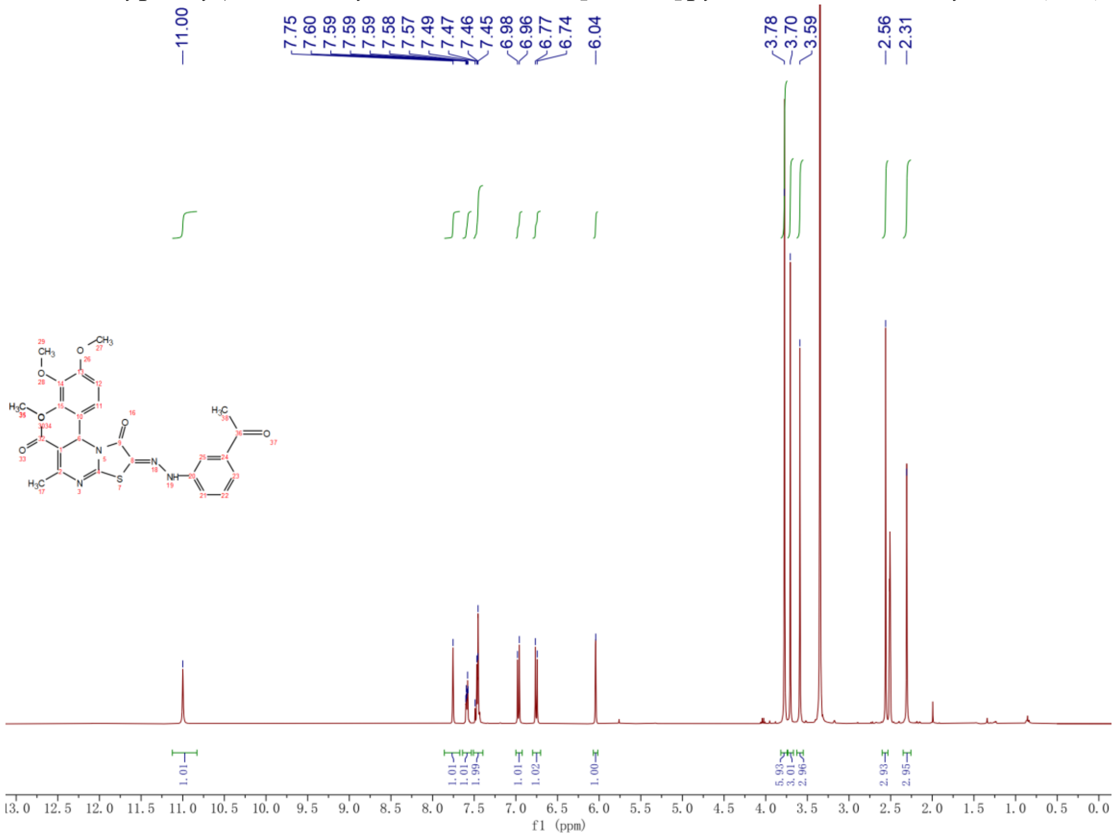

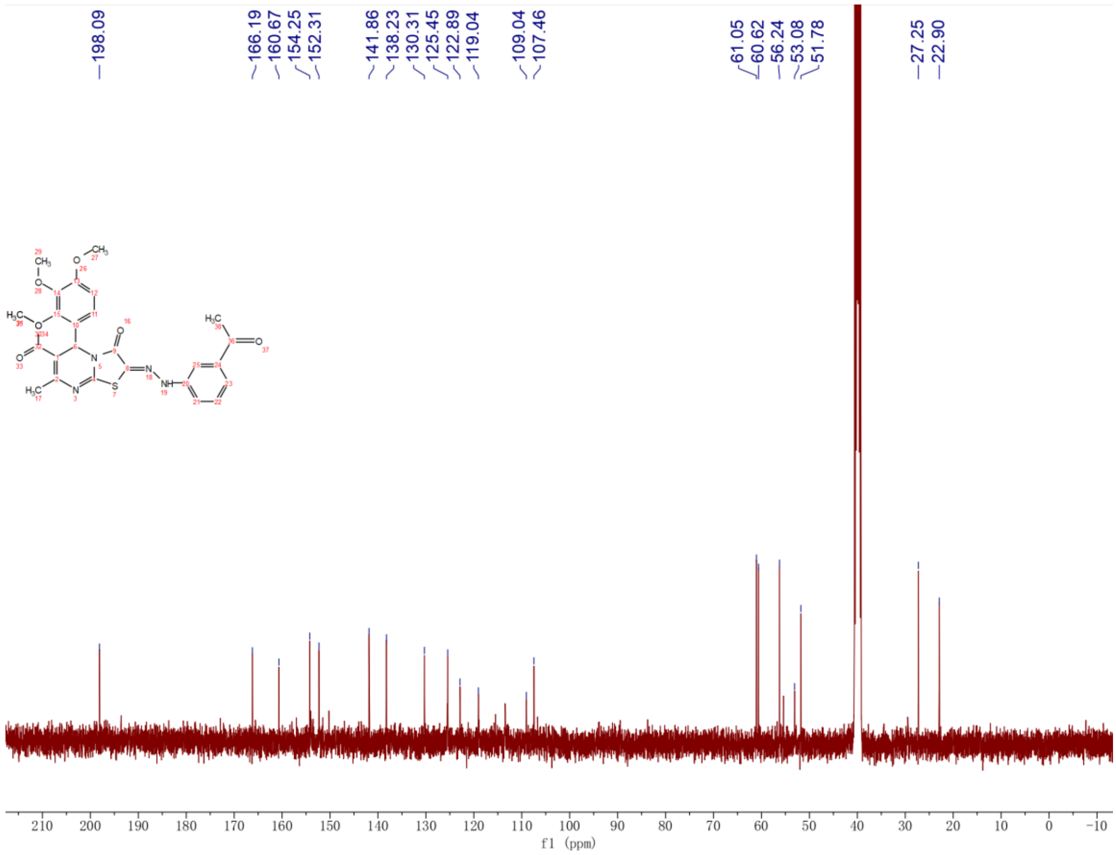

HRMS

Compound 10b [M + H]+

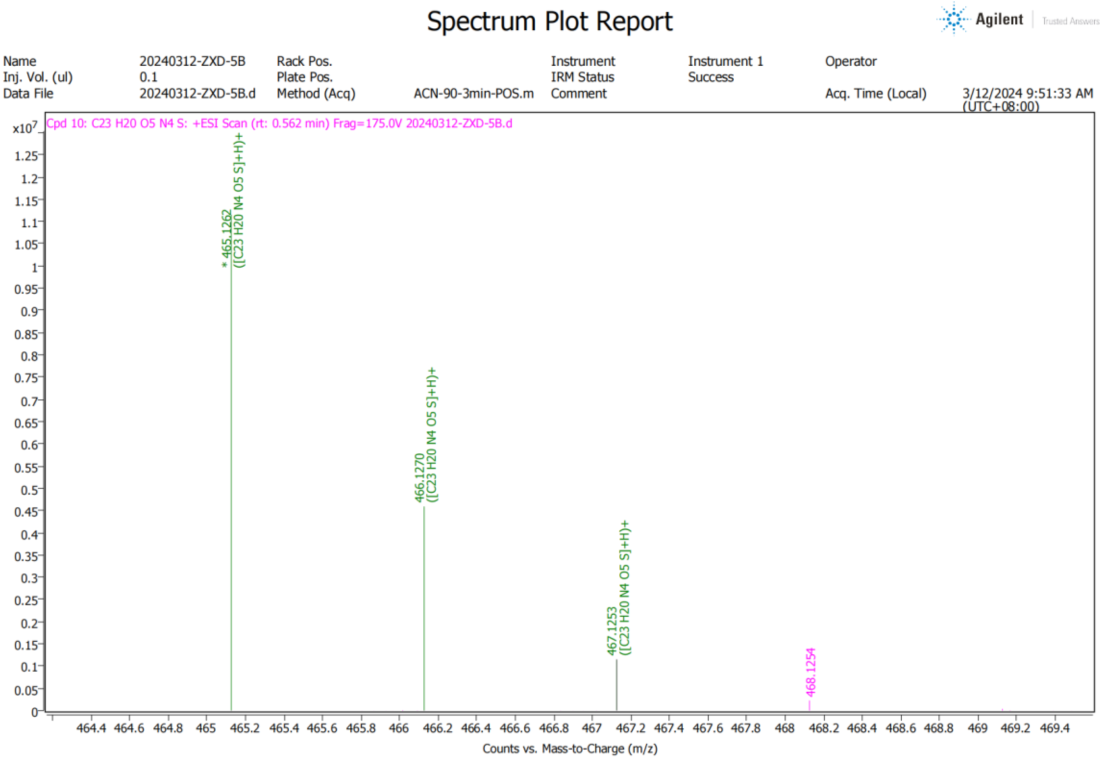

Compound 10c [M + H]+

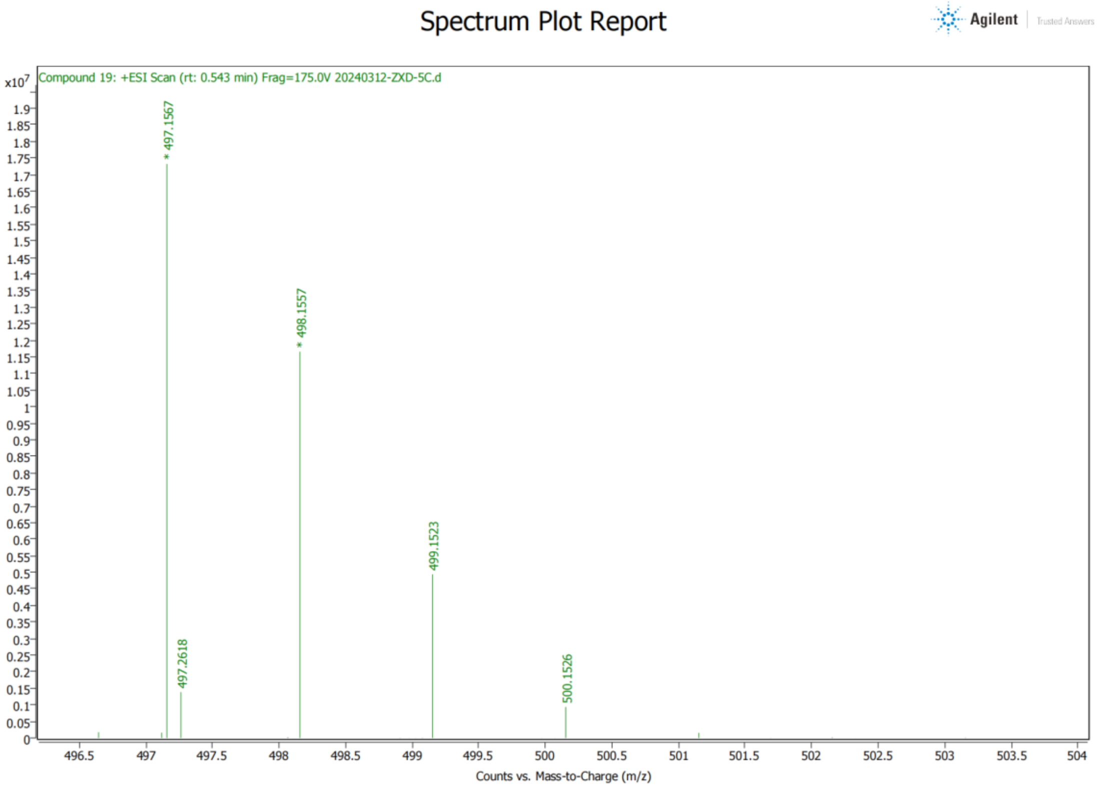

Compound 10d [M + H]$^+$

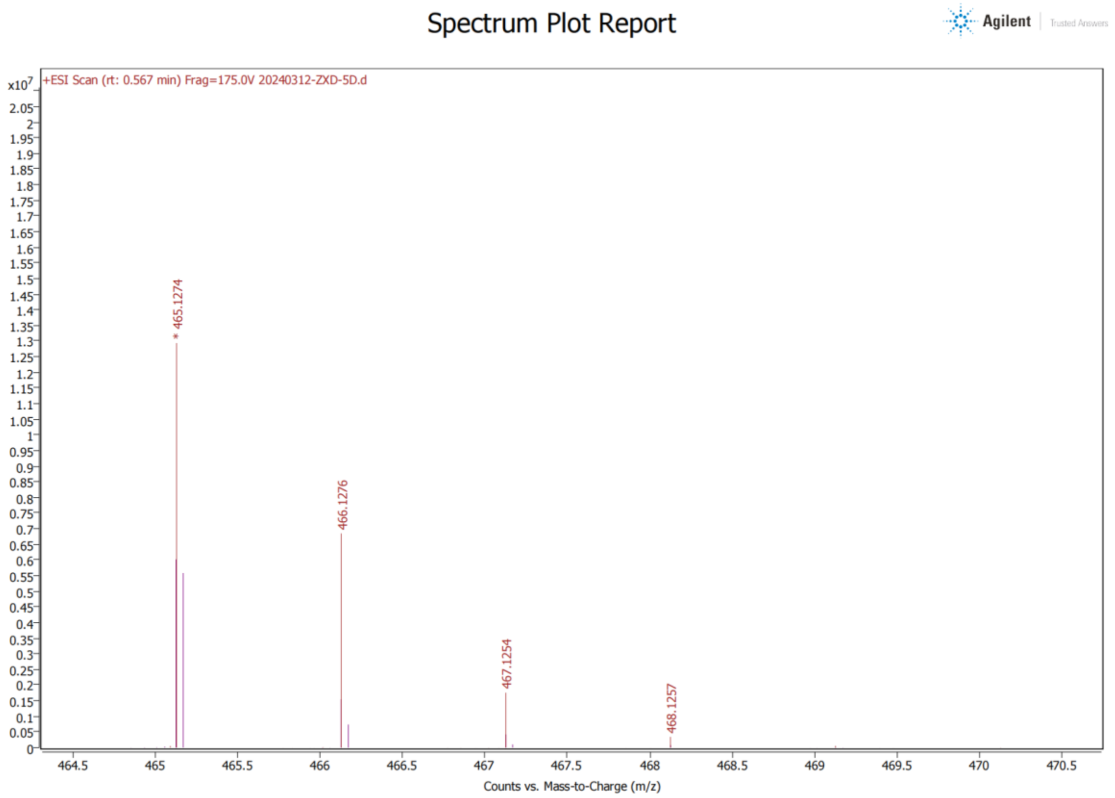

Compound 10e [M + H]$^+$

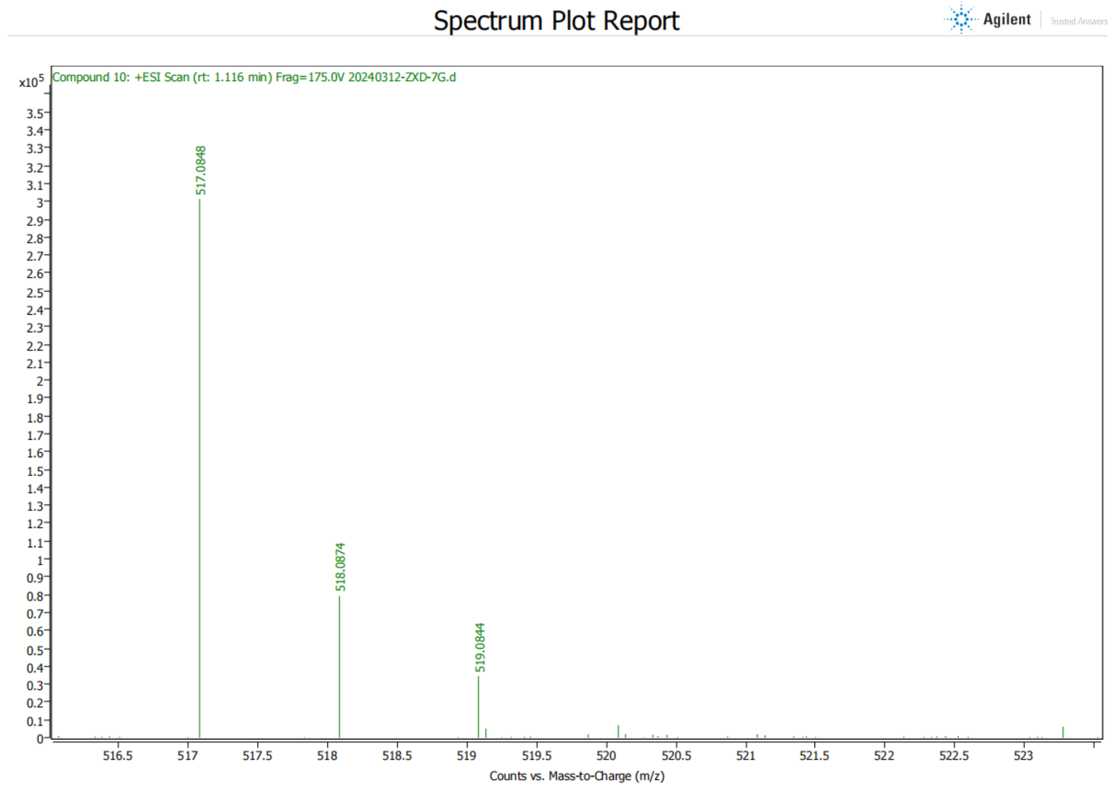

Compound 10h [M + H]$^+$

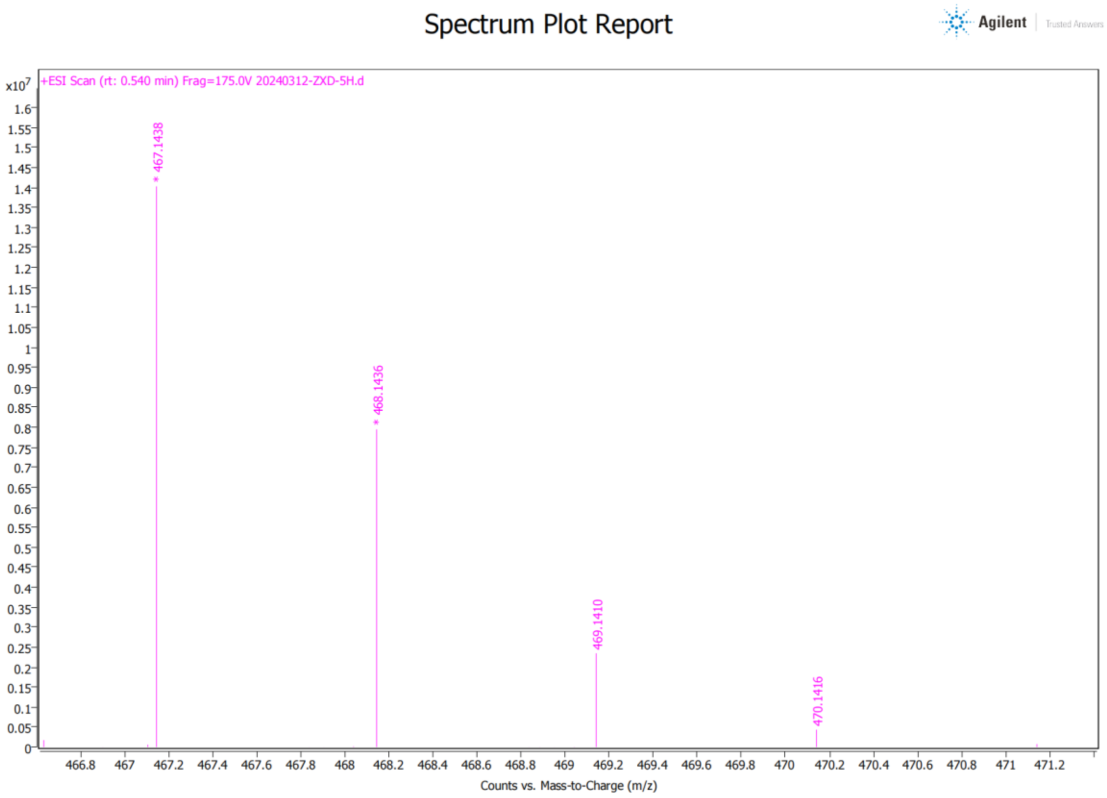

Compound 10i [M + H]$^+$

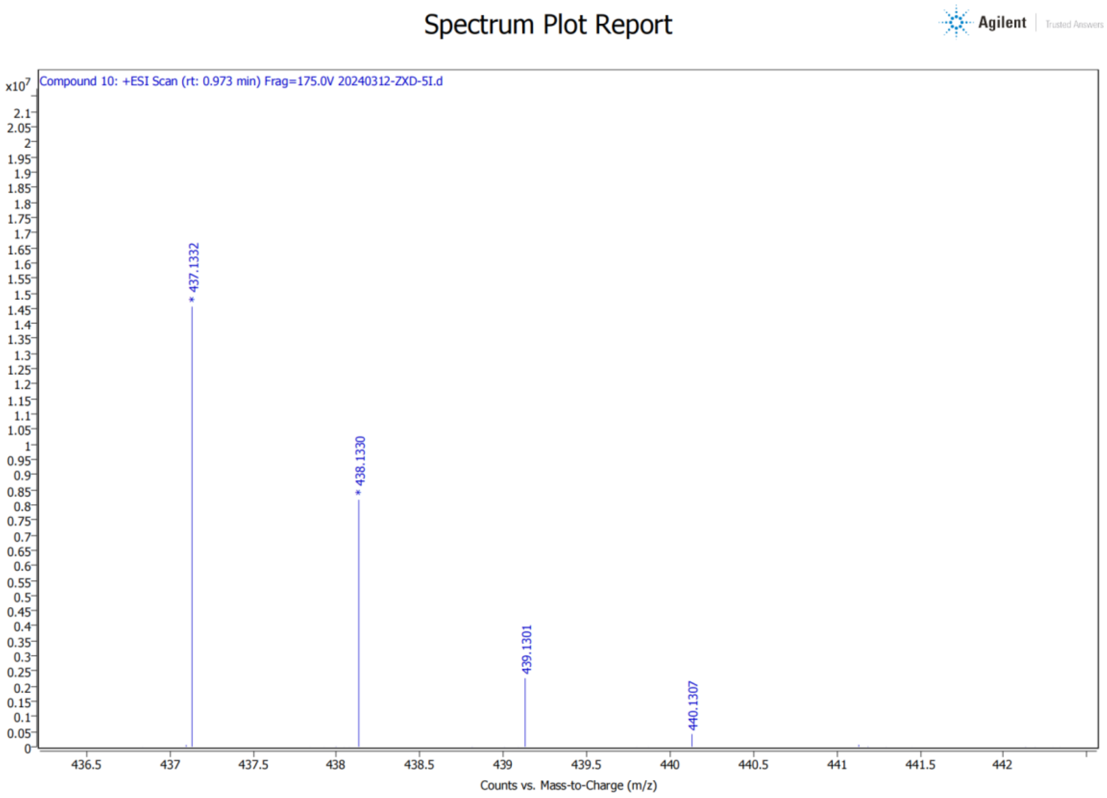

Compound 10m [M + H]$^+$

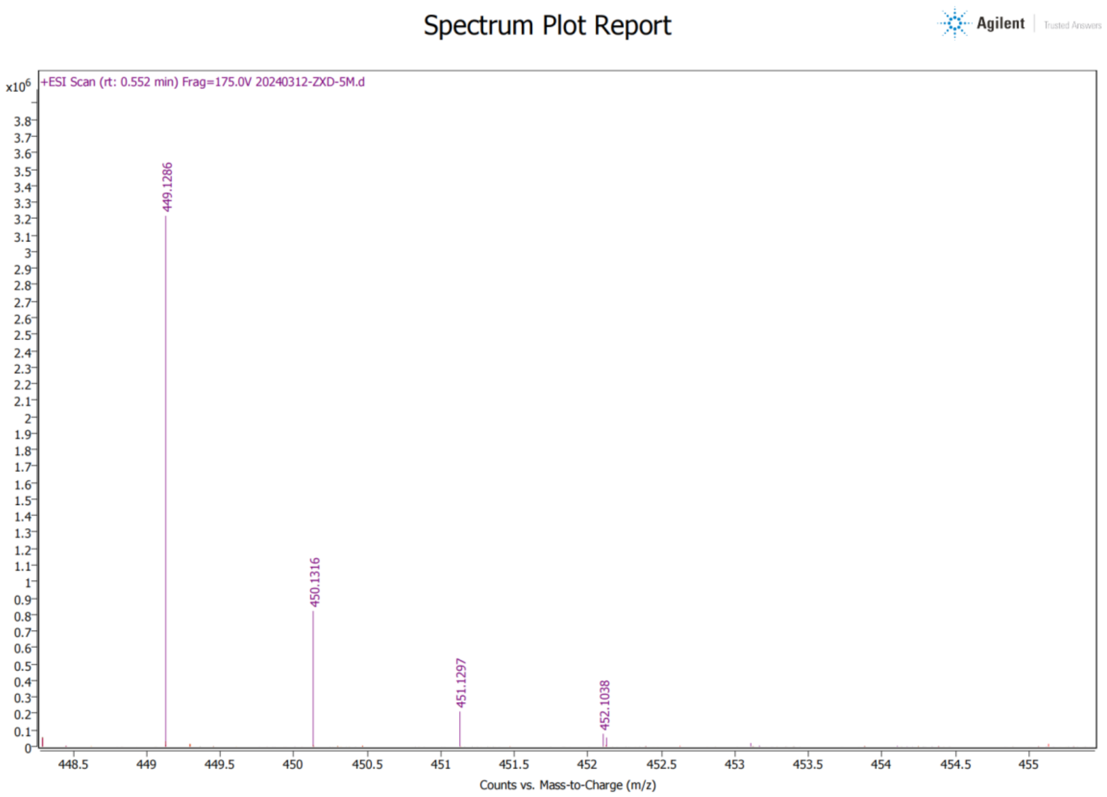

Compound 10n [M + H]$^+$

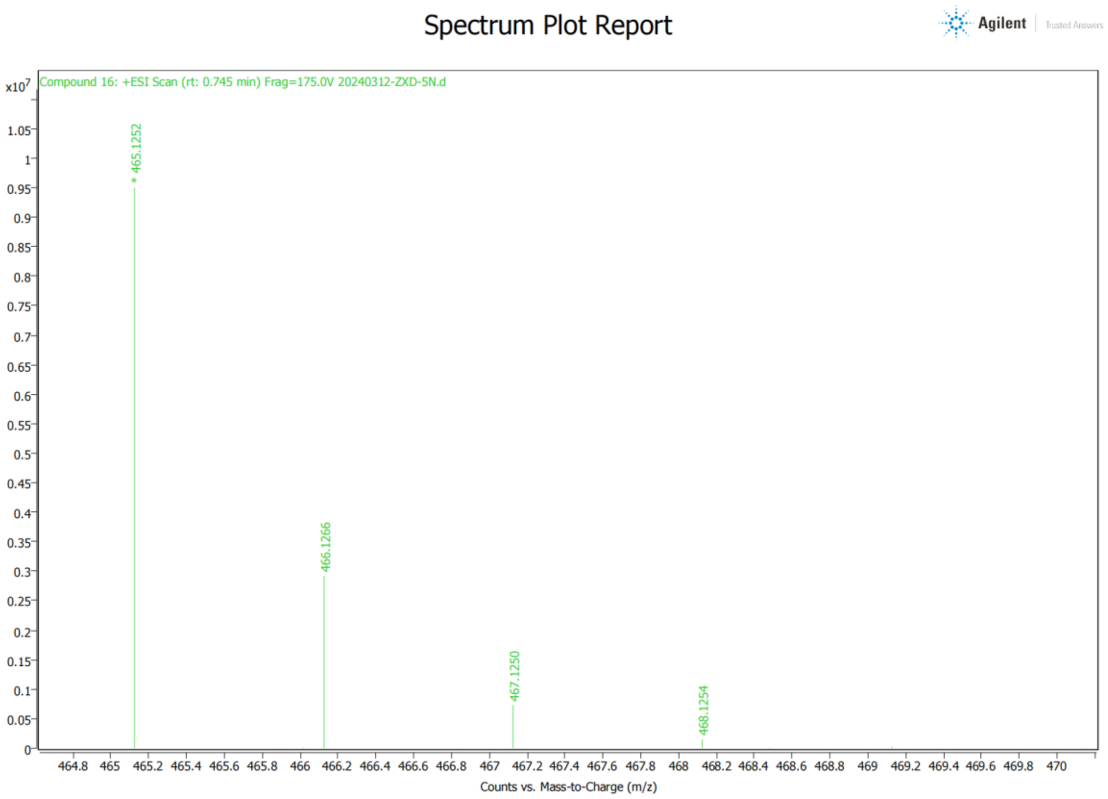

Compound 11a [M + H]$^+$

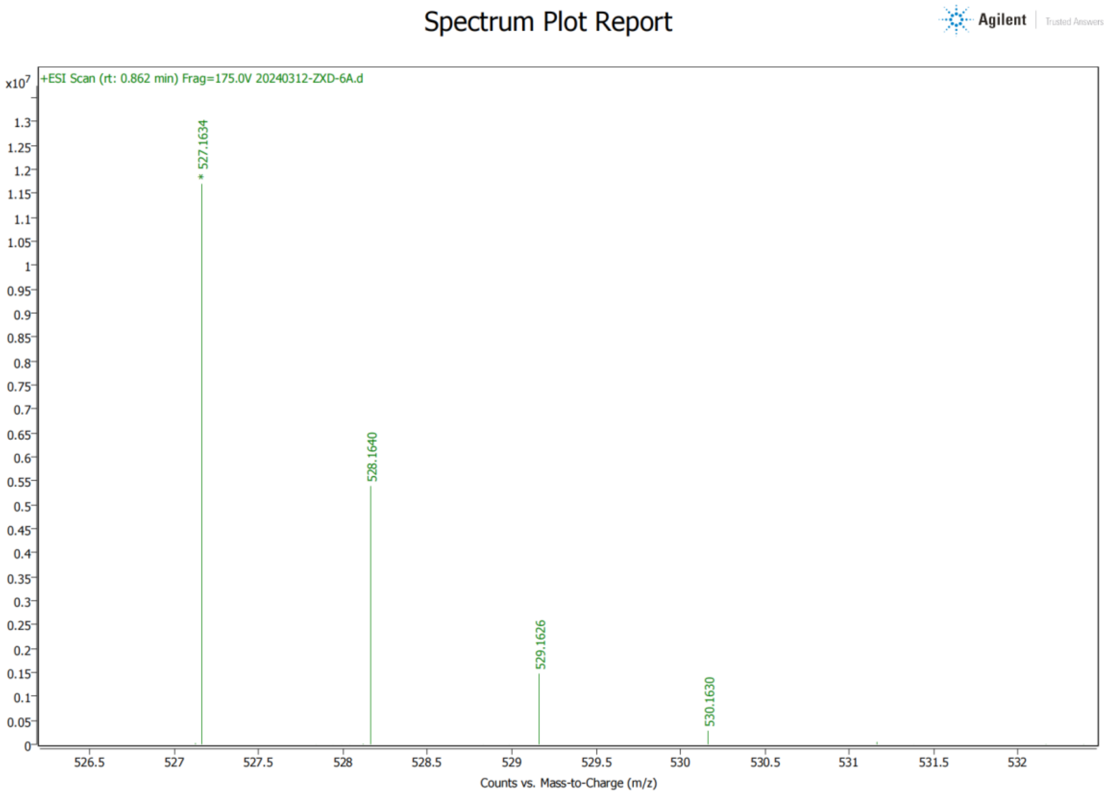

Compound 11b [M + H]$^+$

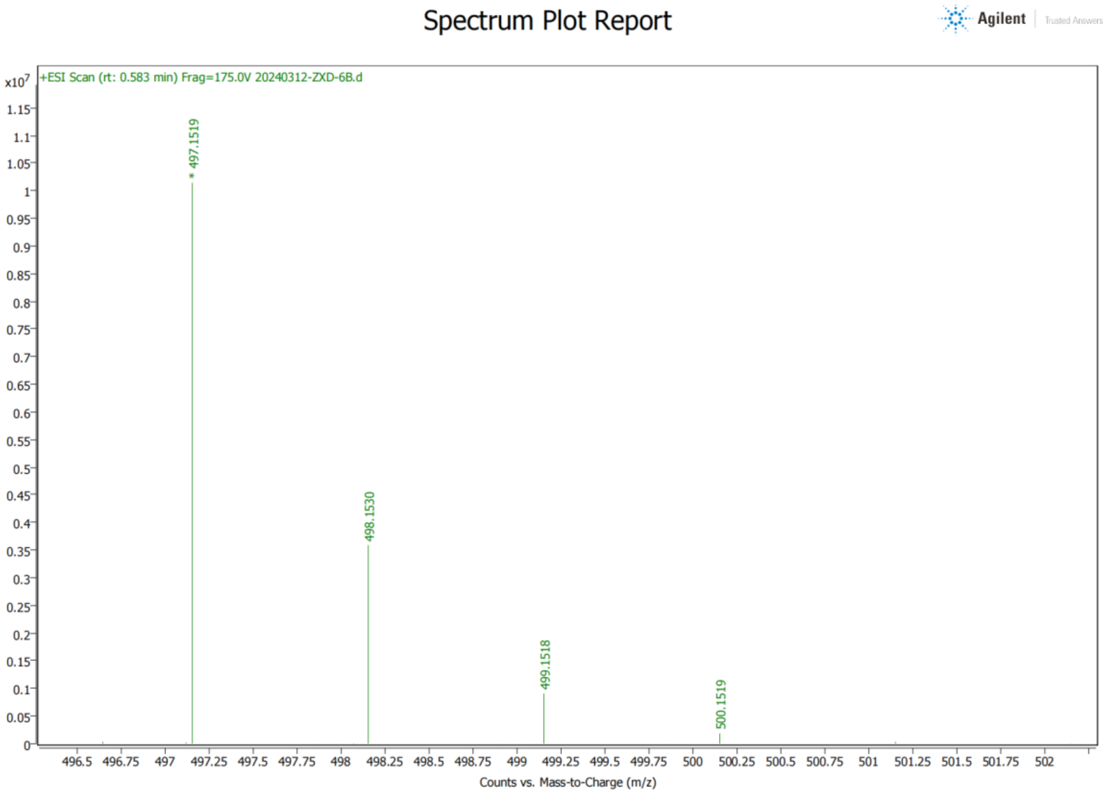

Compound 11c [M + H]$^+$

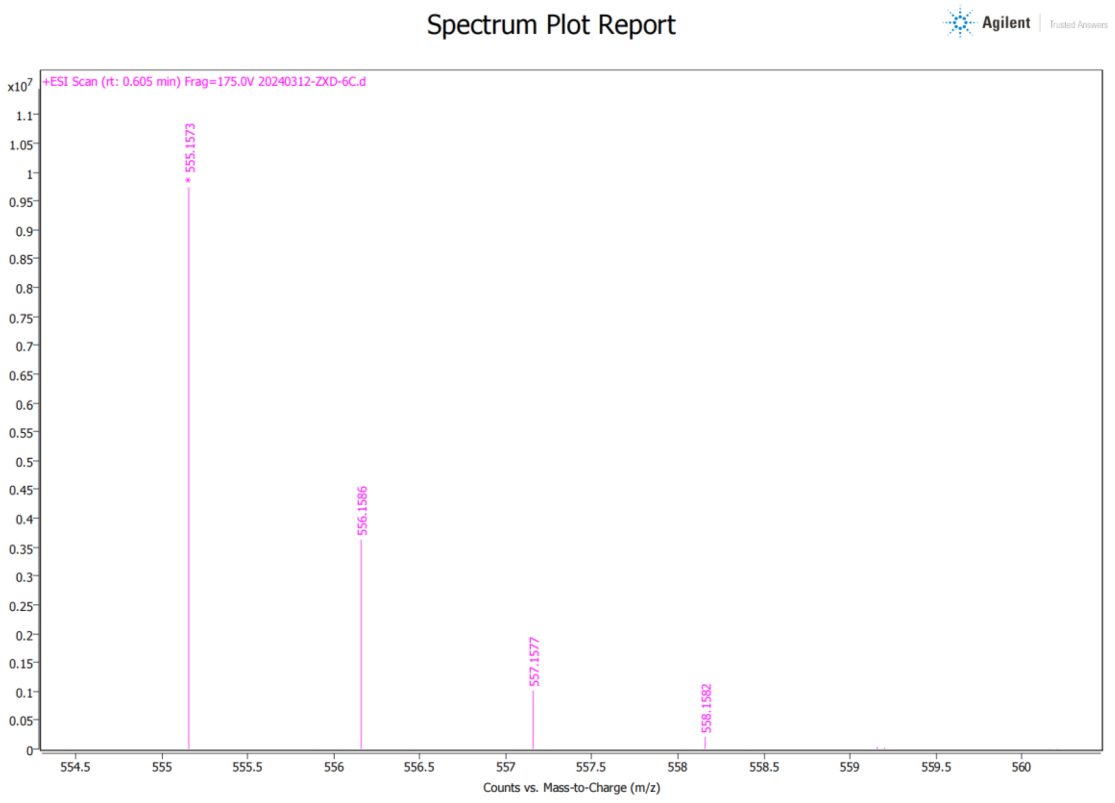

Compound 11d [M + H]$^+$

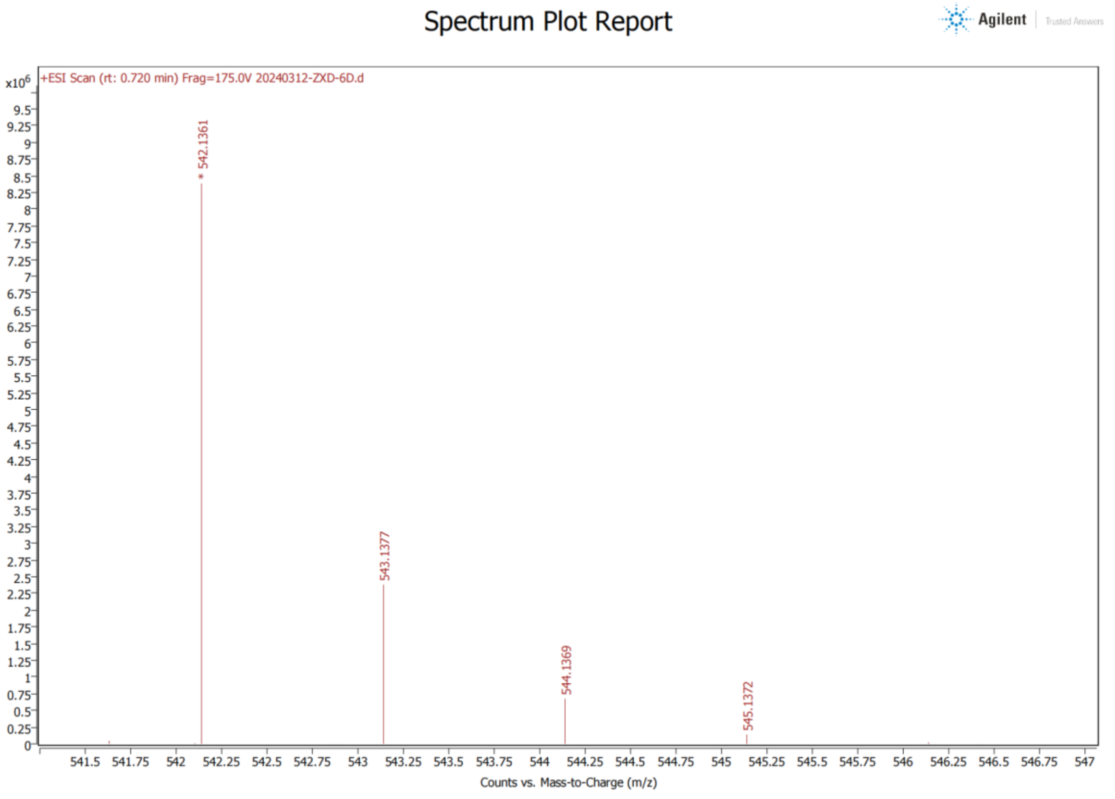

Compound 11e [M + H]$^+$

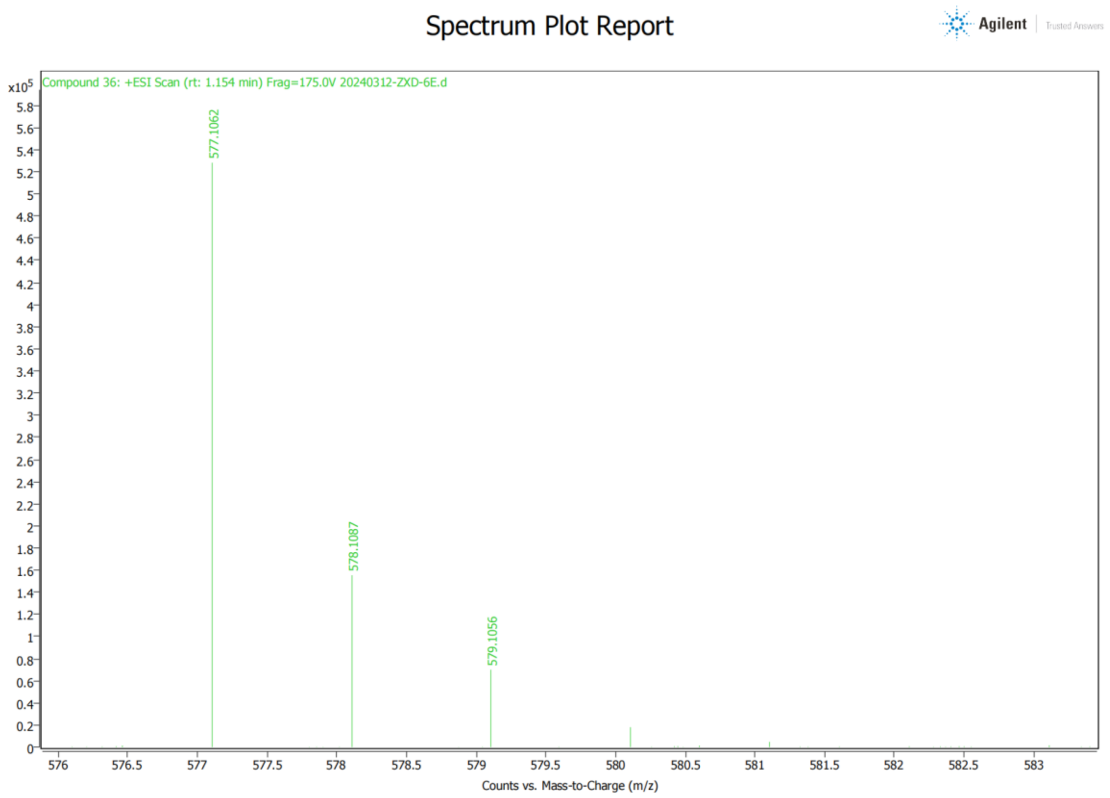

Compound 11h [M + H]$^+$

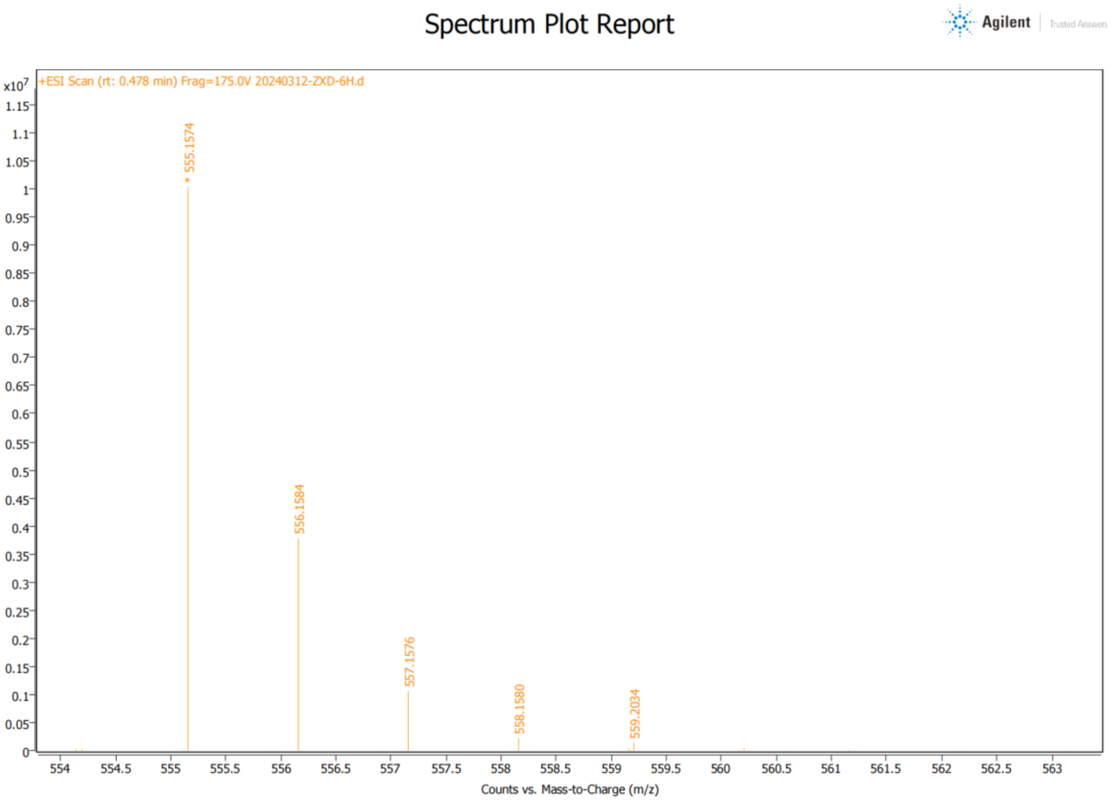

Compound 11i [M + H]$^+$

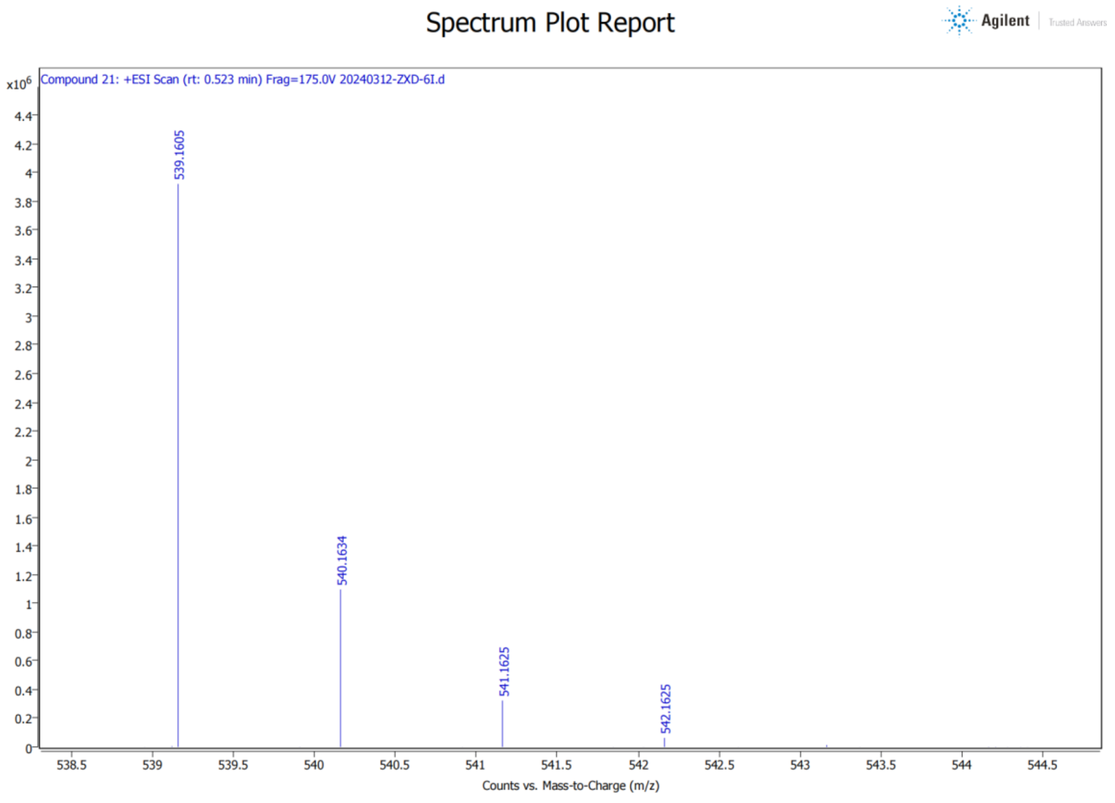

Compound 11j [M + H]$^+$

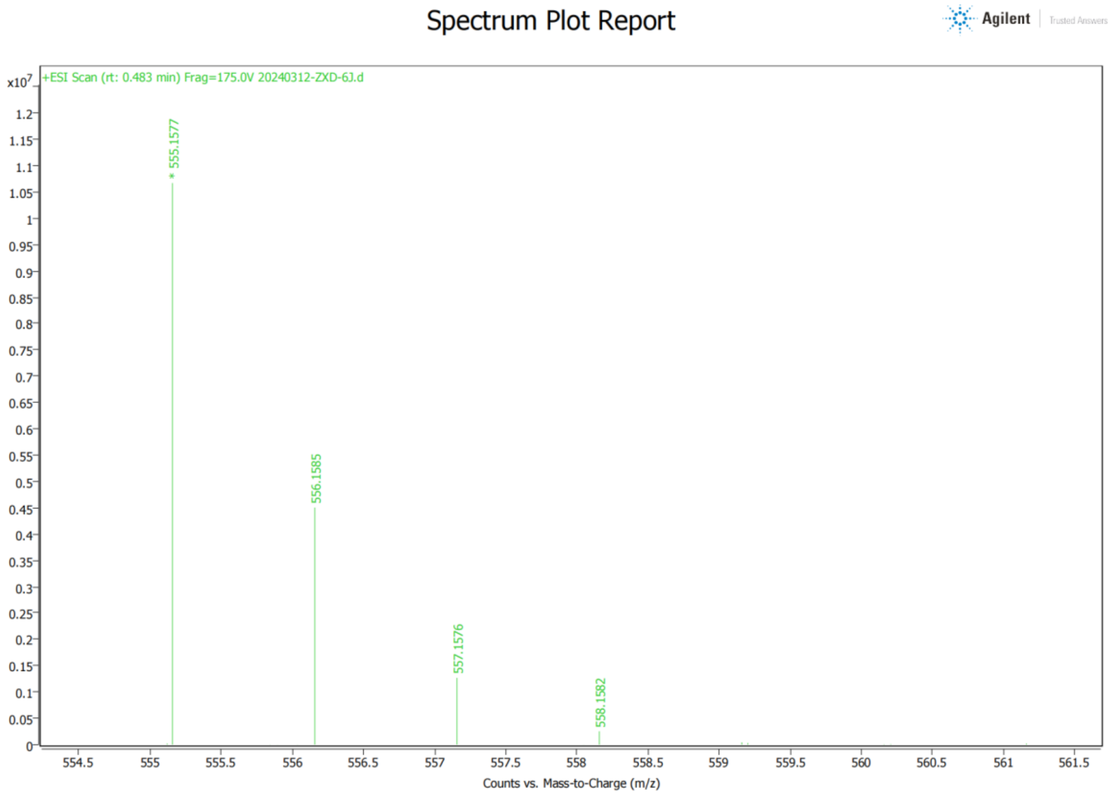

Compound 11k [M + H]$^+$

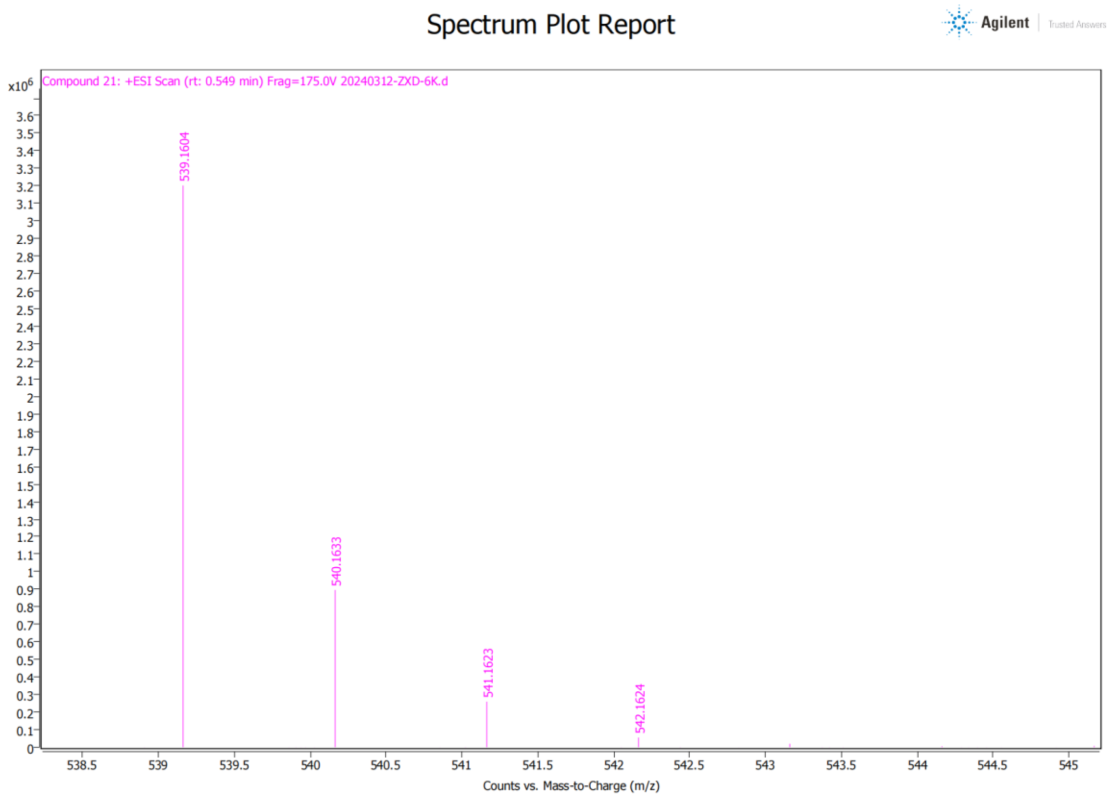

Compound 12b [M + H]$^+$

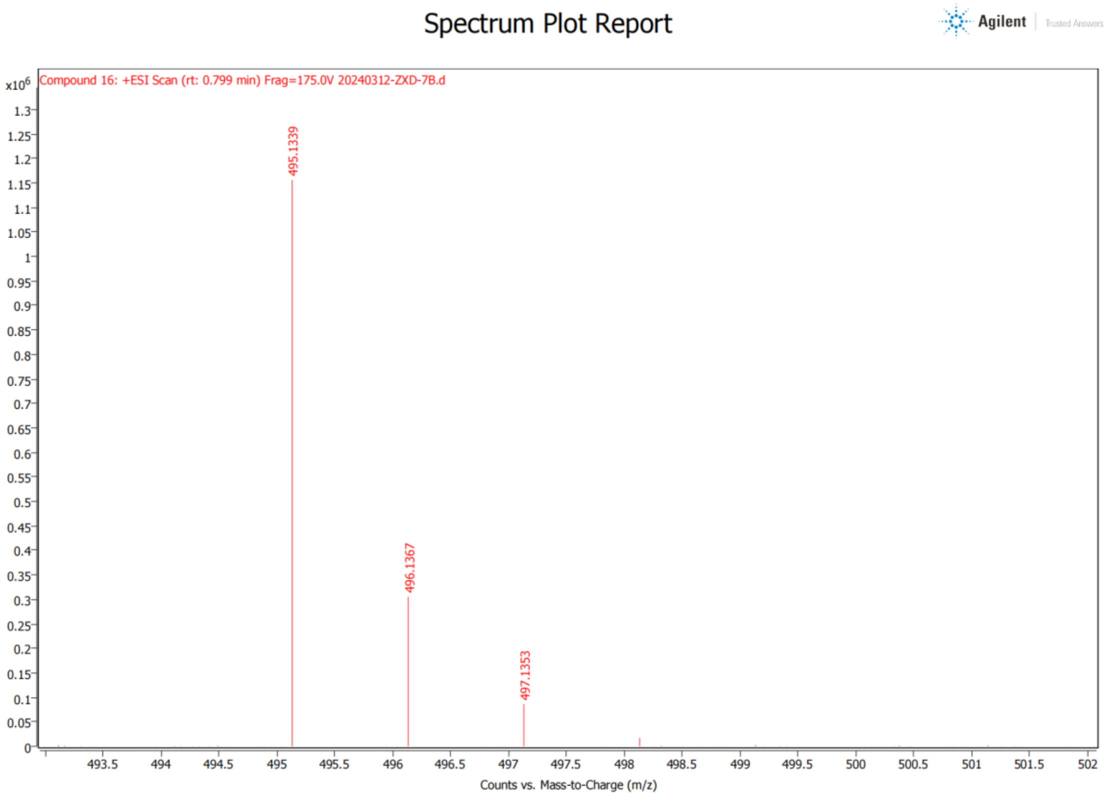

Compound 12c [M + H]$^+$

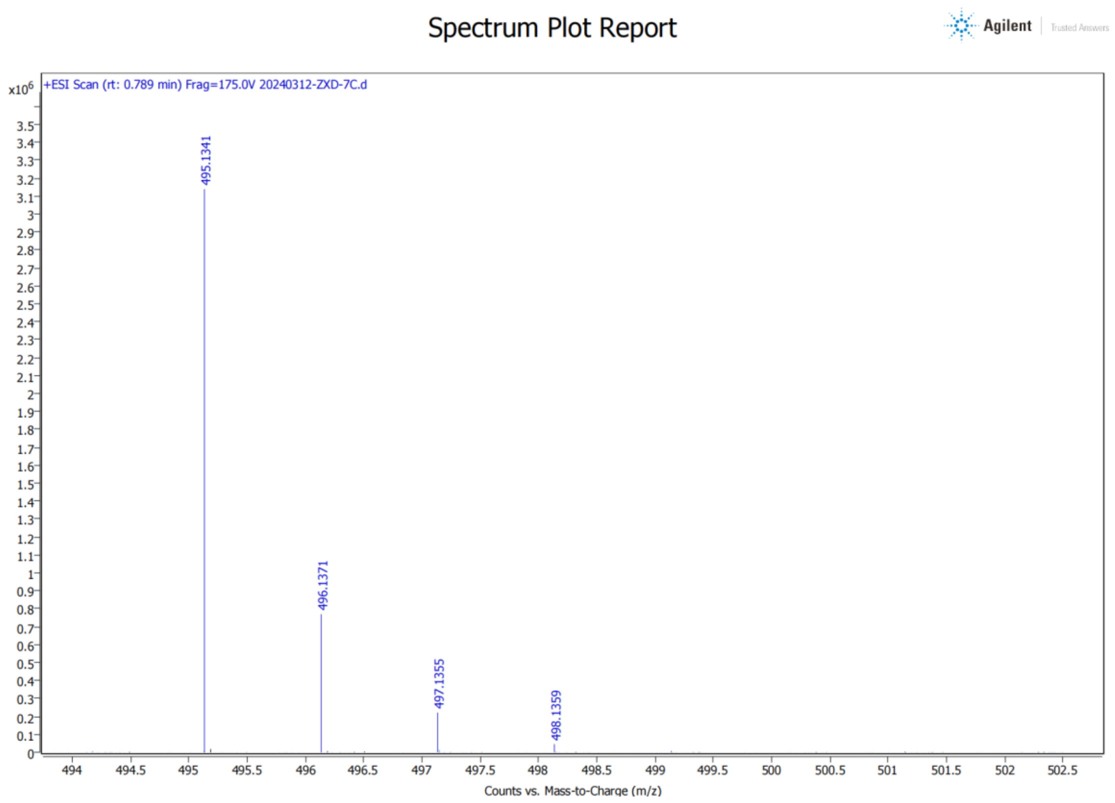

Compound 12d [M + H]$^+$

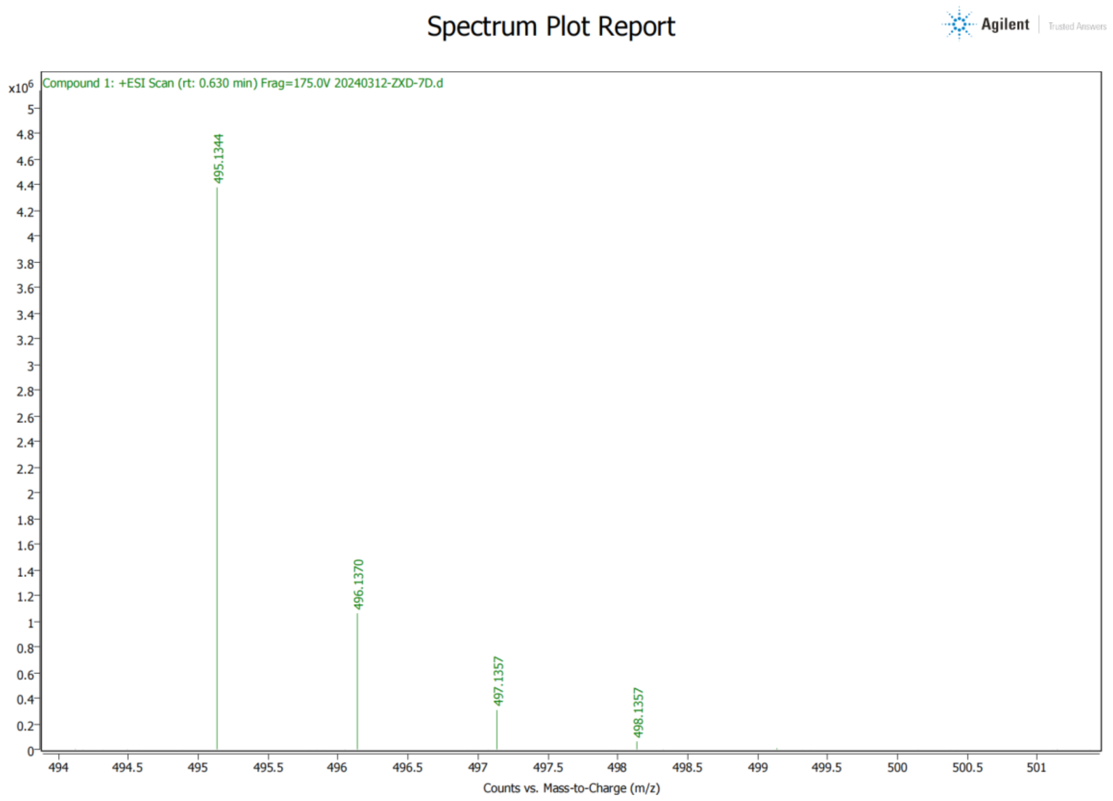

Compound 12e [M + H]$^+$

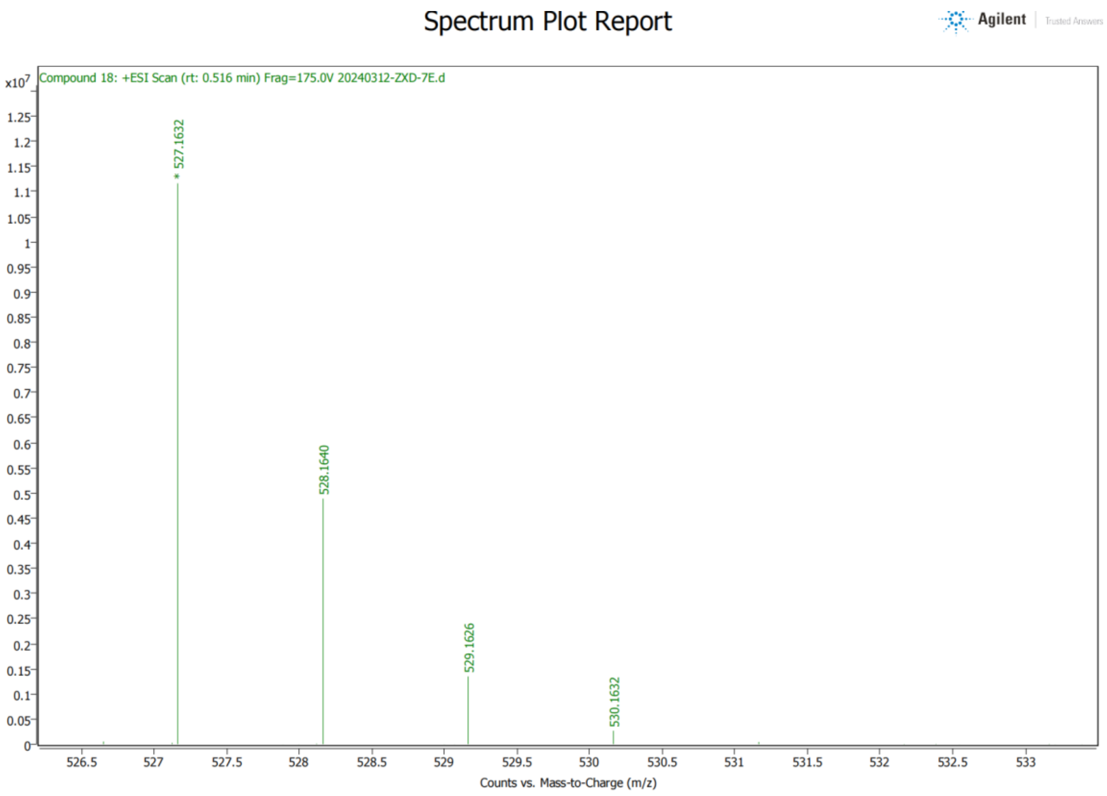

Compound 12f [M + H]$^+$

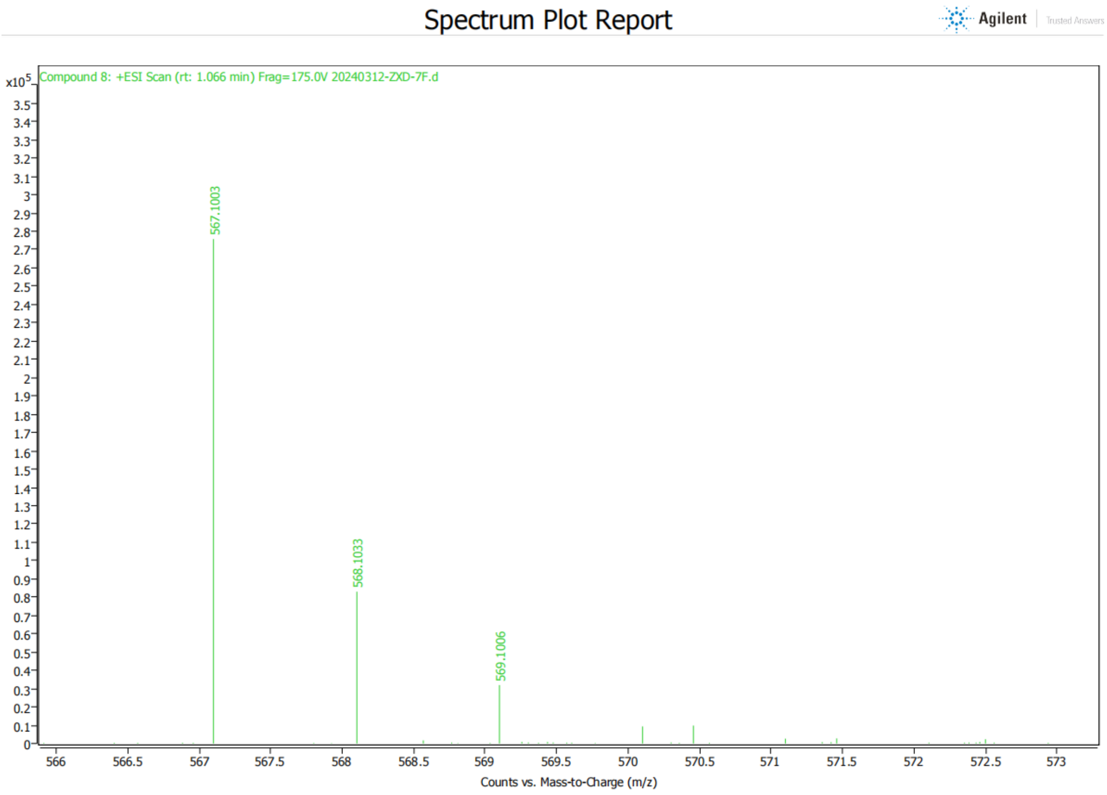

Compound 10a [M - H]$^-$

Display Report

Analysis Info Acquisition Date 3/23/2024 9:49:29 AM

Sample Name 20240322-ZXD-5A
Comment

Acquisition Parameter

Source Type	ESI	Ion Polarity	Negative	Set Nebulizer	2.0 Bar
Focus	Active	Set Capillary	2800 V	Set Dry Heater	200 °C
Scan Begin	50 m/z	Set End Plate Offset	-500 V	Set Dry Gas	8.0 l/min
Scan End	1500 m/z	Set Charging Voltage	2000 V	Set Divert Valve	Waste
		Set Corona	0 nA	Set APCI Heater	0 °C

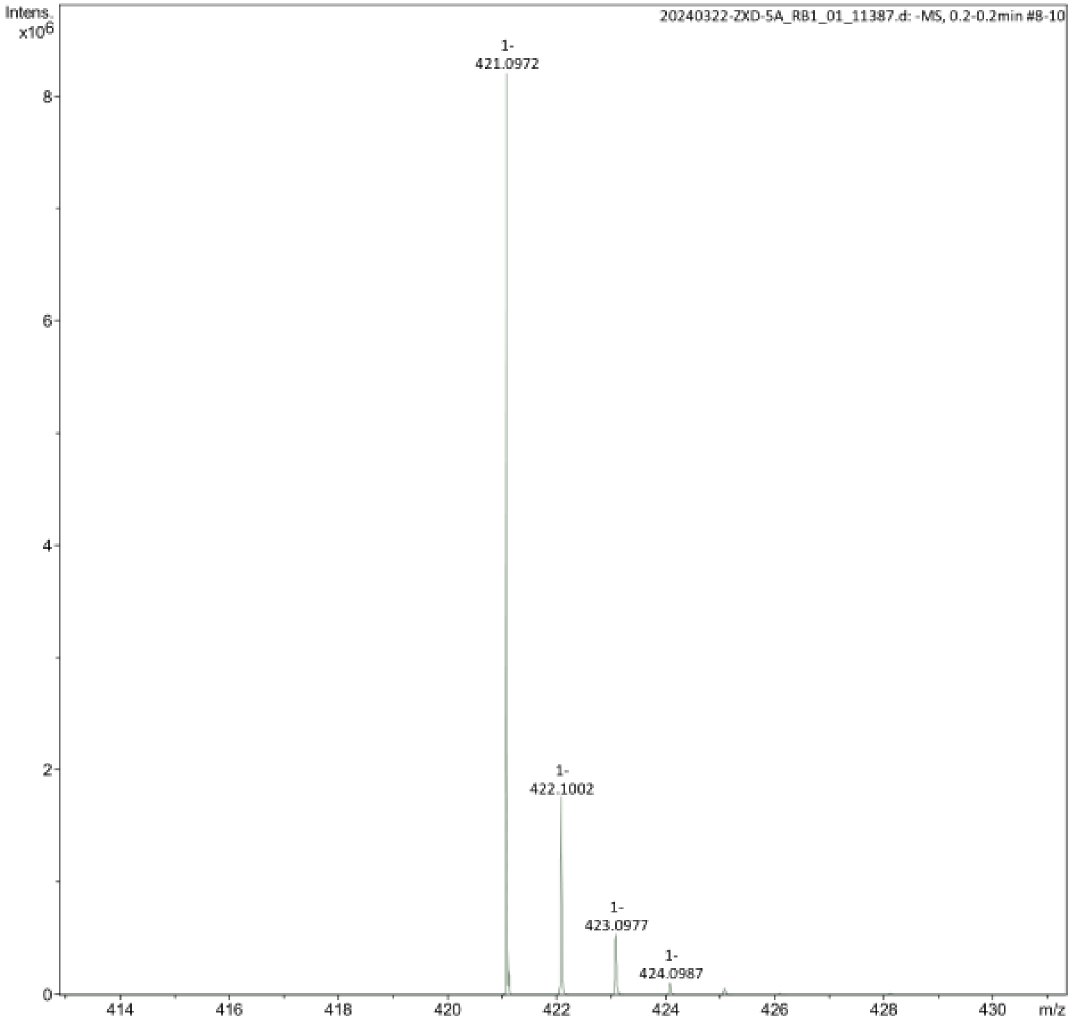

Compound 10f [M - H]$^-$

Display Report

Analysis Info Acquisition Date 3/23/2024 9:55:01 AM

Sample Name 20240322-ZXD-5F
Comment

Acquisition Parameter

Source Type	ESI	Ion Polarity	Negative	Set Nebulizer	2.0 Bar
Focus	Active	Set Capillary	2800 V	Set Dry Heater	200 °C
Scan Begin	50 m/z	Set End Plate Offset	-500 V	Set Dry Gas	8.0 l/min
Scan End	1500 m/z	Set Charging Voltage	2000 V	Set Divert Valve	Waste
		Set Corona	0 nA	Set APCI Heater	0 °C

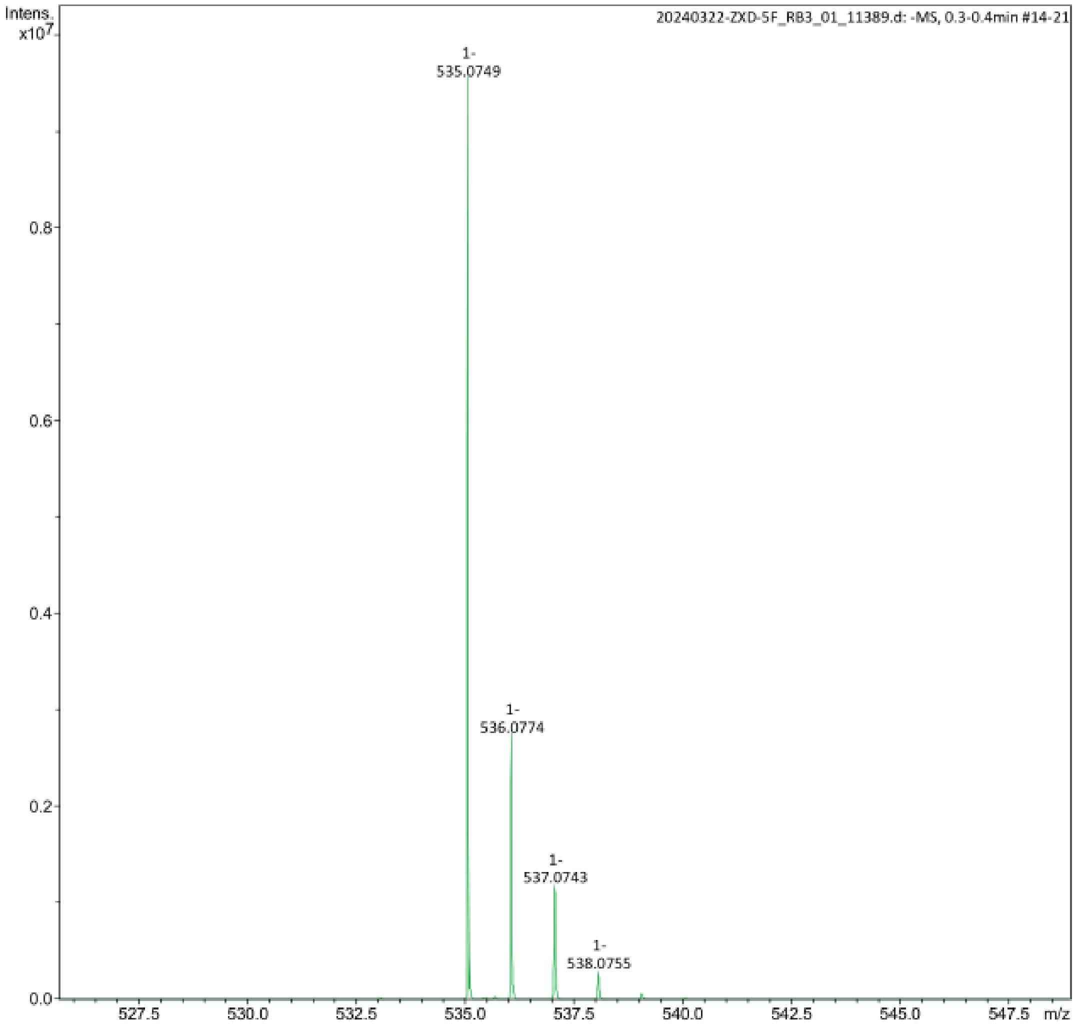

Compound 10g [M - H]$^-$

Display Report

Analysis Info

Acquisition Date 3/23/2024 9:57:45 AM

Sample Name 20240322-ZXD-5G
Comment

Acquisition Parameter

Source Type	ESI	Ion Polarity	Negative	Set Nebulizer	2.0 Bar
Focus	Active	Set Capillary	2800 V	Set Dry Heater	200 °C
Scan Begin	50 m/z	Set End Plate Offset	-500 V	Set Dry Gas	8.0 l/min
Scan End	1500 m/z	Set Charging Voltage	2000 V	Set Divert Valve	Waste
		Set Corona	0 nA	Set APCI Heater	0 °C

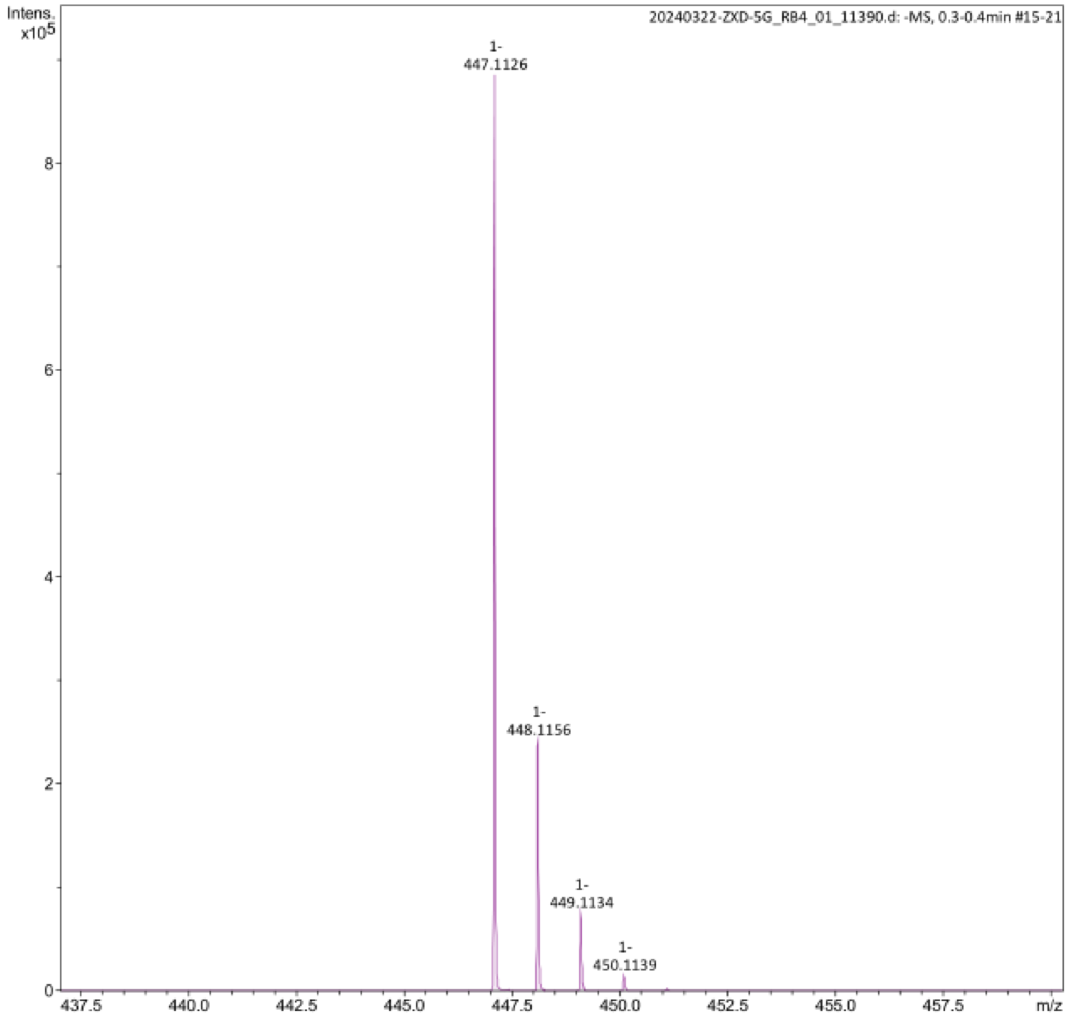

Compound 10j [M - H]$^-$

Display Report

Analysis Info

Acquisition Date 3/23/2024 10:00:31 AM

Sample Name 20240322-ZXD-5J
Comment

Acquisition Parameter

Source Type	ESI	Ion Polarity	Negative	Set Nebulizer	2.0 Bar
Focus	Active	Set Capillary	2800 V	Set Dry Heater	200 °C
Scan Begin	50 m/z	Set End Plate Offset	-500 V	Set Dry Gas	8.0 l/min
Scan End	1500 m/z	Set Charging Voltage	2000 V	Set Divert Valve	Waste
		Set Corona	0 nA	Set APCI Heater	0 °C

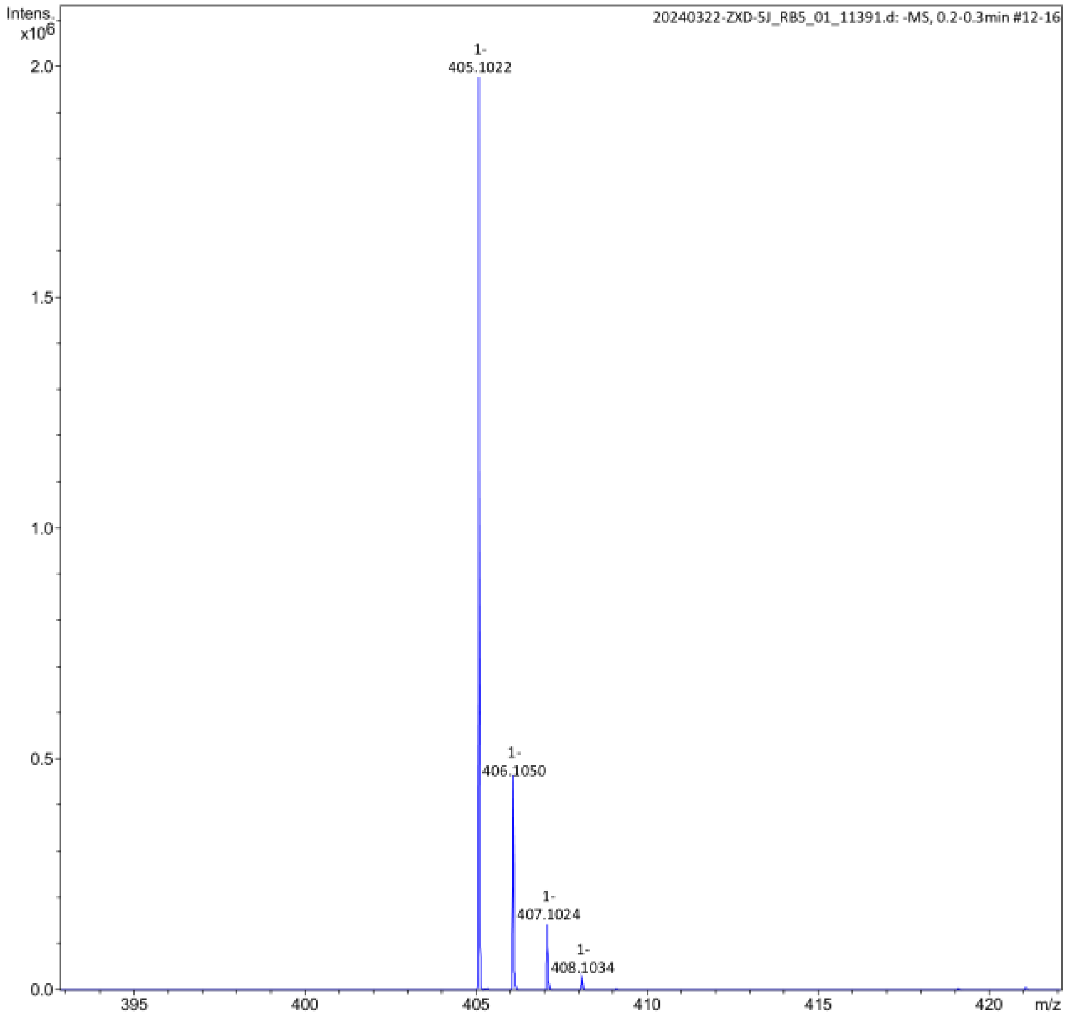

Compound 10k [M - H]$^-$

Display Report

Analysis Info

Acquisition Date 3/23/2024 10:03:18 AM

Sample Name 20240322-ZXD-5K
Comment

Acquisition Parameter

Source Type	ESI	Ion Polarity	Negative	Set Nebulizer	2.0 Bar
Focus	Active	Set Capillary	2800 V	Set Dry Heater	200 °C
Scan Begin	50 m/z	Set End Plate Offset	-500 V	Set Dry Gas	8.0 l/min
Scan End	1500 m/z	Set Charging Voltage	2000 V	Set Divert Valve	Waste
		Set Corona	0 nA	Set APCI Heater	0 °C

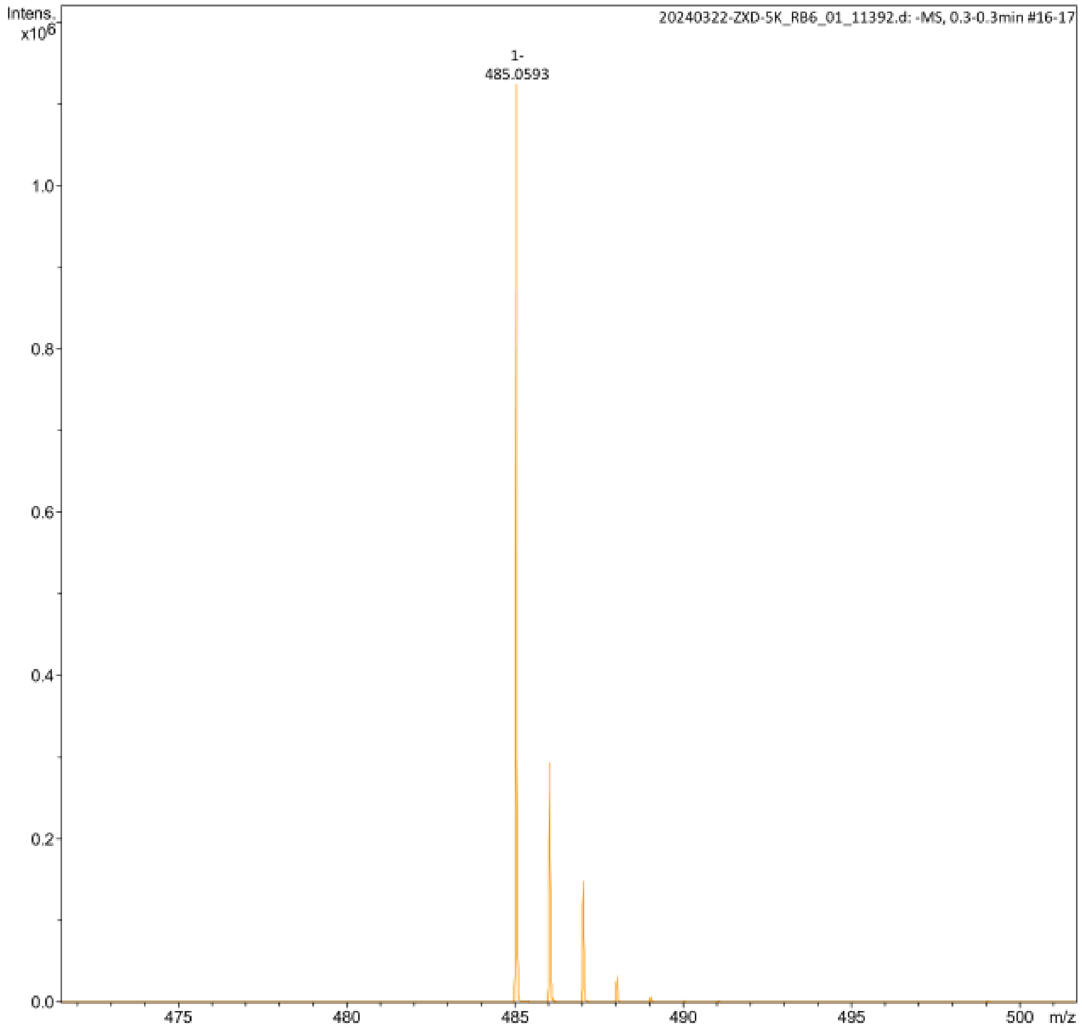

Compound 10l [M - H]$^-$

Display Report

Analysis Info

Acquisition Date 3/23/2024 10:06:03 AM

Sample Name 20240322-ZXD-5L
Comment

Acquisition Parameter

Source Type	ESI	Ion Polarity	Negative	Set Nebulizer	2.0 Bar
Focus	Active	Set Capillary	2800 V	Set Dry Heater	200 °C
Scan Begin	50 m/z	Set End Plate Offset	-500 V	Set Dry Gas	8.0 l/min
Scan End	1500 m/z	Set Charging Voltage	2000 V	Set Divert Valve	Waste
		Set Corona	0 nA	Set APCI Heater	0 °C

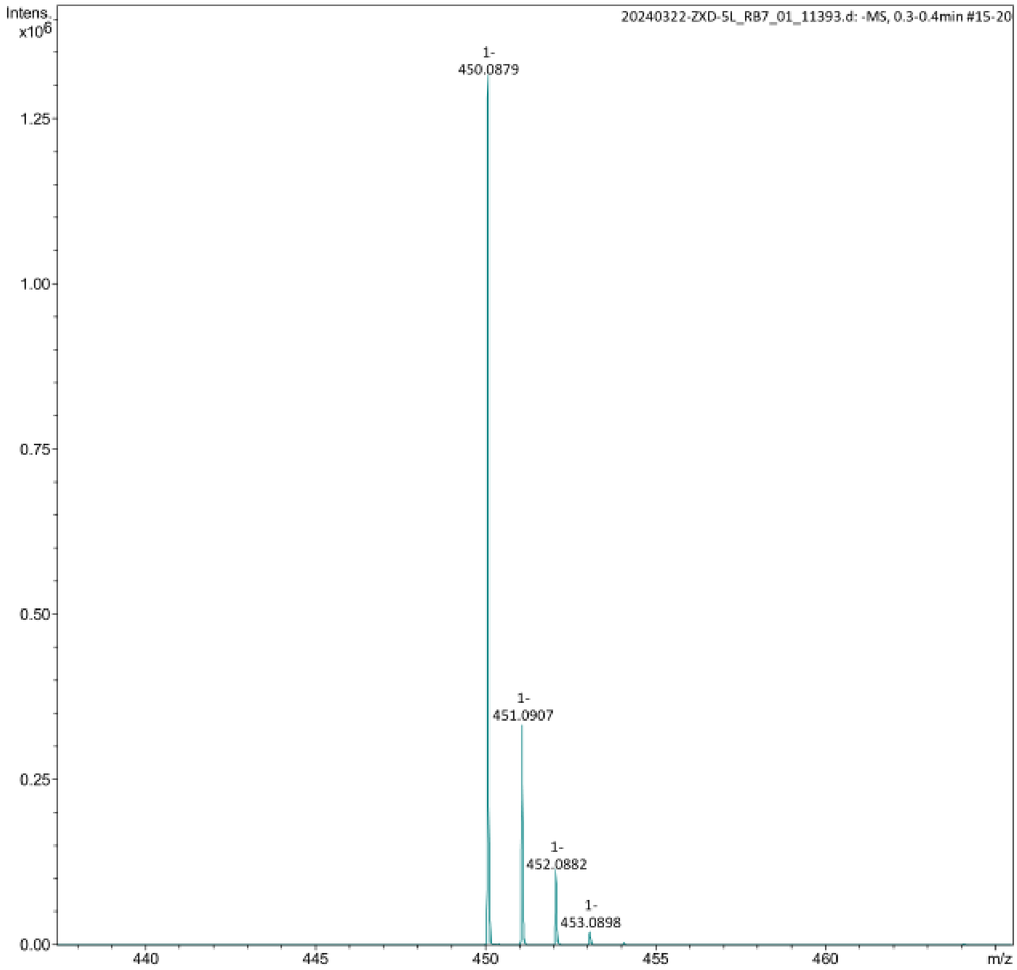

Compound 11f [M - H]⁻

Display Report

Analysis Info

Acquisition Date 3/23/2024 10:08:49 AM

Sample Name 20240322-ZXD-6F
Comment

Acquisition Parameter

Source Type	ESI	Ion Polarity	Negative	Set Nebulizer	2.0 Bar
Focus	Active	Set Capillary	2800 V	Set Dry Heater	200 °C
Scan Begin	50 m/z	Set End Plate Offset	-500 V	Set Dry Gas	8.0 l/min
Scan End	1500 m/z	Set Charging Voltage	2000 V	Set Divert Valve	Waste
		Set Corona	0 nA	Set APCI Heater	0 °C

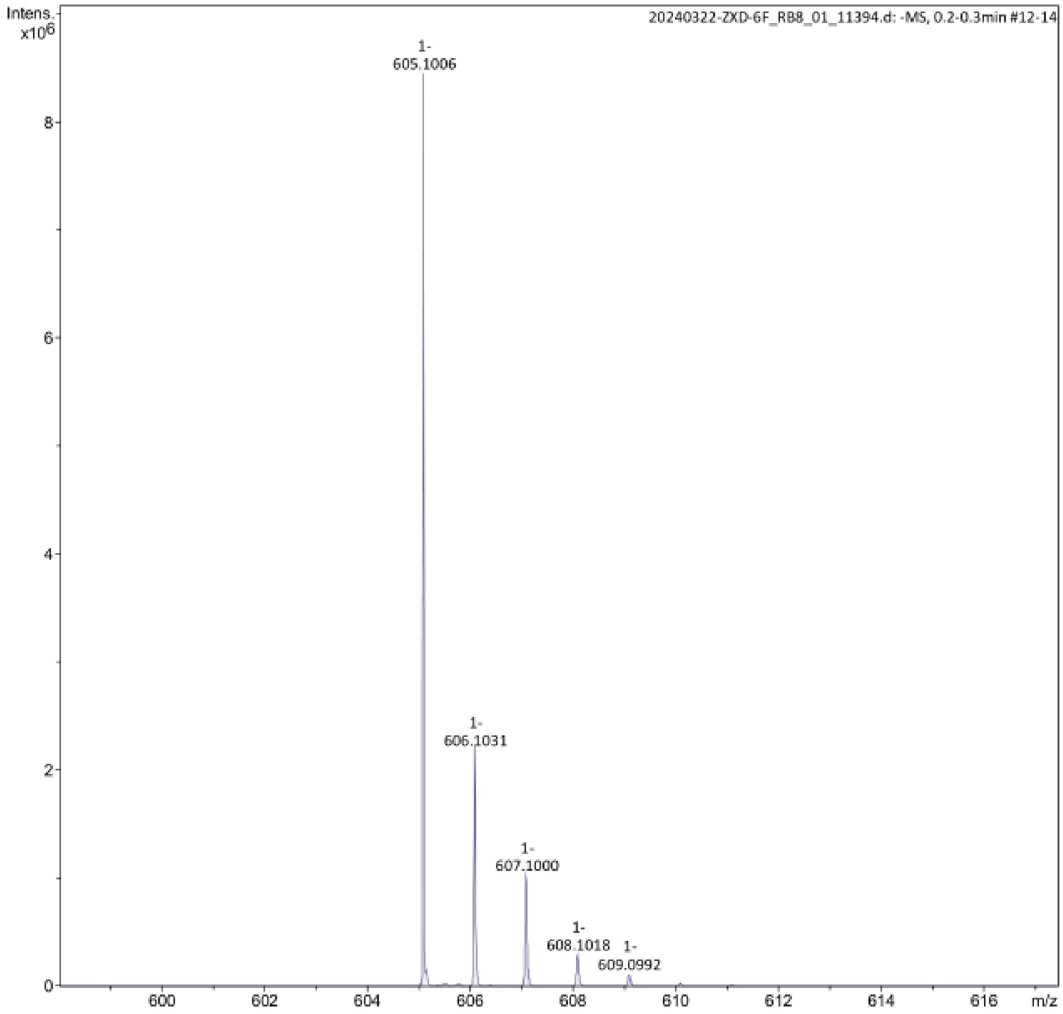

Compound 11g [M - H]$^-$

Display Report

Analysis Info

Acquisition Date 3/23/2024 10:11:35 AM

Sample Name 20240322-ZXD-6G

Comment

Acquisition Parameter

Source Type	ESI	Ion Polarity	Negative	Set Nebulizer	2.0 Bar
Focus	Active	Set Capillary	2800 V	Set Dry Heater	200 °C
Scan Begin	50 m/z	Set End Plate Offset	-500 V	Set Dry Gas	8.0 l/min
Scan End	1500 m/z	Set Charging Voltage	2000 V	Set Divert Valve	Waste
		Set Corona	0 nA	Set APCI Heater	0 °C

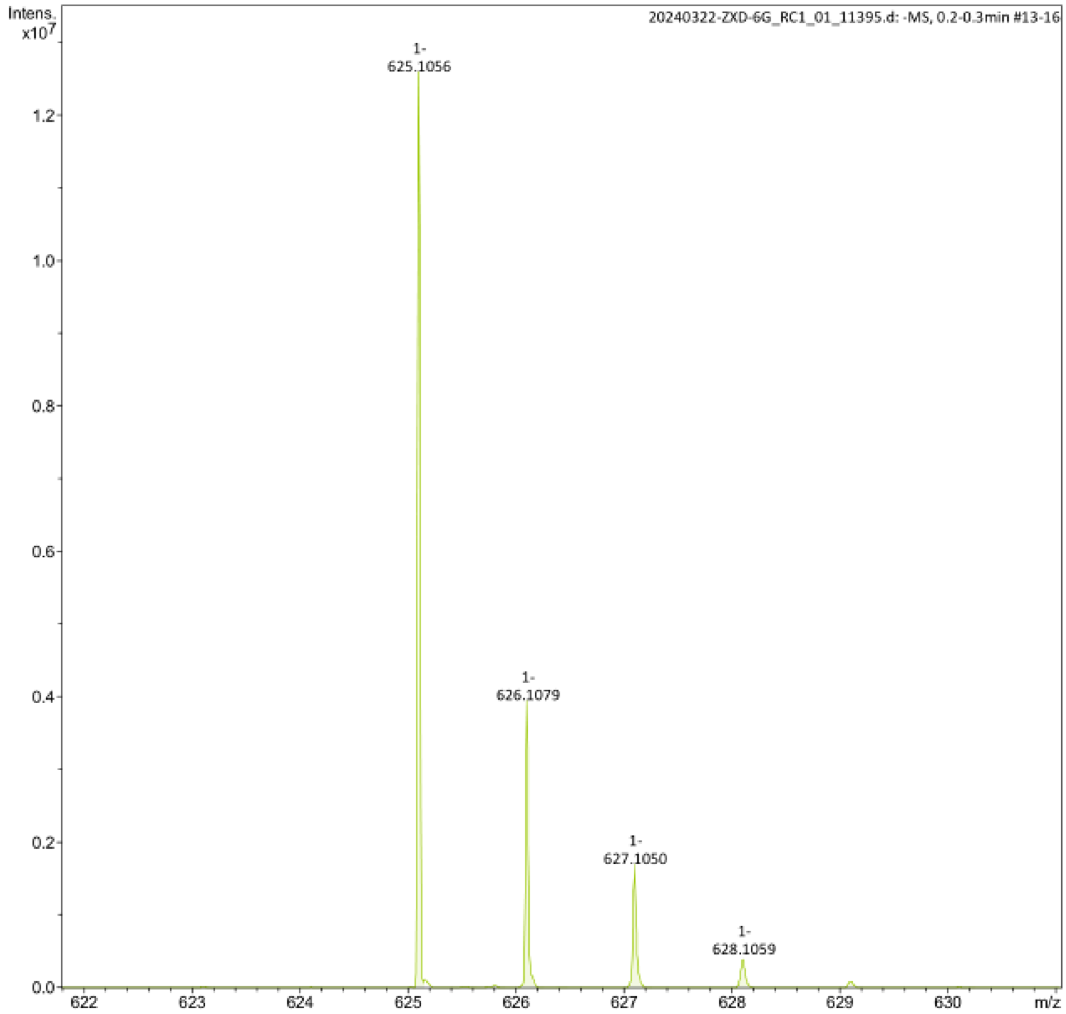

Compound 12a [M - H]$^-$

Display Report

Analysis Info

Acquisition Date 3/23/2024 10:14:20 AM

Sample Name 20240322-ZXD-12A
Comment

Acquisition Parameter

Source Type	ESI	Ion Polarity	Negative	Set Nebulizer	2.0 Bar
Focus	Active	Set Capillary	2800 V	Set Dry Heater	200 °C
Scan Begin	50 m/z	Set End Plate Offset	-500 V	Set Dry Gas	8.0 l/min
Scan End	1500 m/z	Set Charging Voltage	2000 V	Set Divert Valve	Waste
		Set Corona	0 nA	Set APCI Heater	0 °C

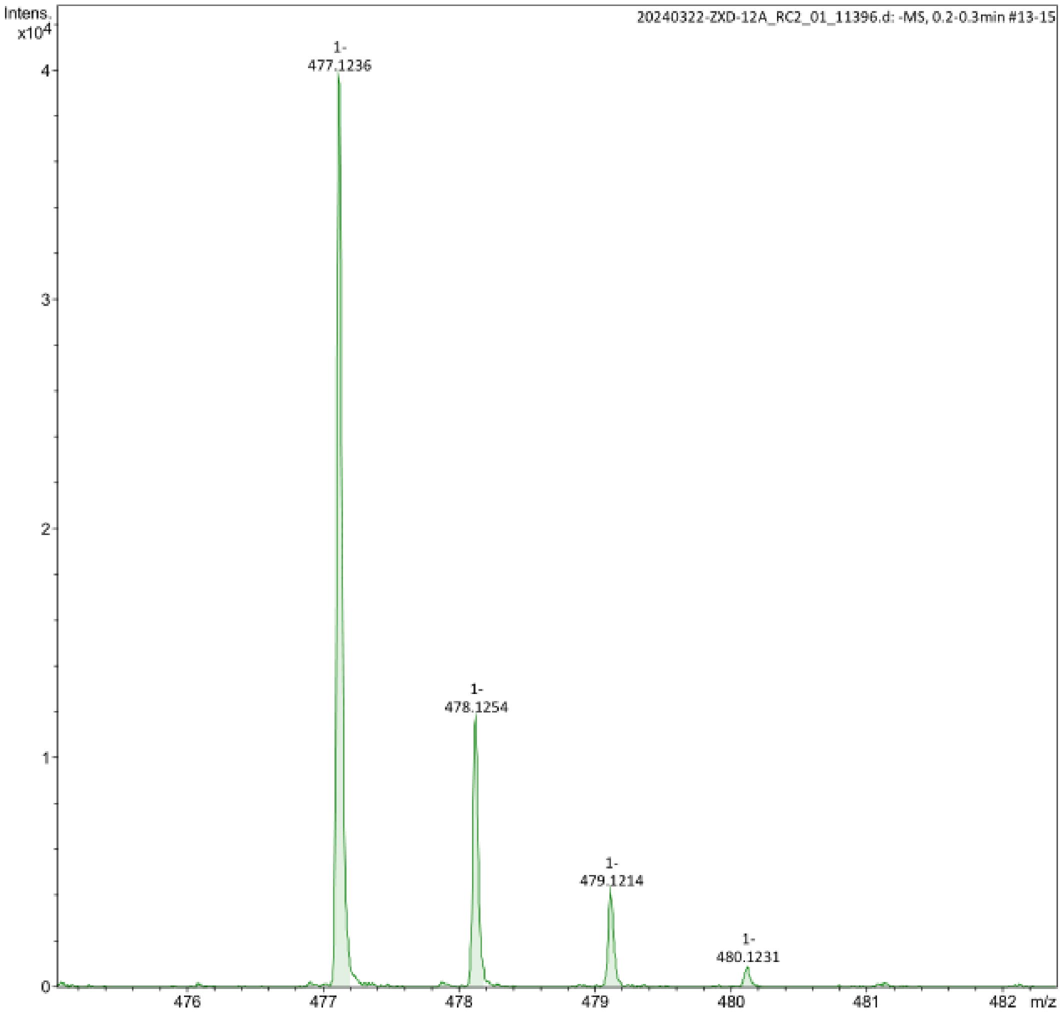

HPLC

Compound 10a

```
Acq. Operator   : BY                              Seq. Line :    1
Acq. Instrument : 1260R                            Location :    2
Injection Date  : 4/11/2024 10:09:00 PM                 Inj :    1
                                                 Inj Volume : 20.000 µl
Acq. Method     : D:\Data\ZXD\DATA\20240411-ZXD-1\zxd-1F_LC 2024-04-11 22-07-34\20240316--ZK-
                  15MIN-35-95ACN.M
Last changed    : 3/16/2024 8:20:27 PM by BY
Analysis Method : D:\DATA\KUN\METHOD\20240204-ZK-15MIN-25-90ACN.M
Last changed    : 4/11/2024 11:02:36 PM by BY
                  (modified after loading)
Method Info     : 90% H2O and 10% ACE

Additional Info : Peak(s) manually integrated
```

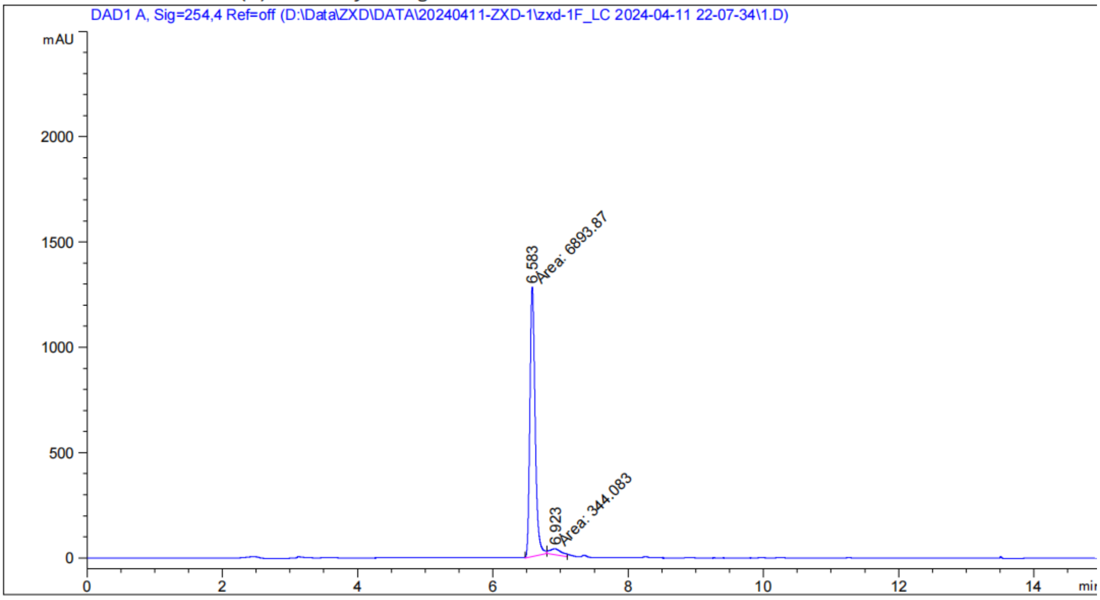


```
=====================================================================
                         Area Percent Report
=====================================================================

Sorted By             :      Signal
Multiplier            :      1.0000
Dilution              :      1.0000
Do not use Multiplier & Dilution Factor with ISTDs


Signal 1: DAD1 A, Sig=254,4 Ref=off

Peak RetTime Type  Width     Area      Height     Area
  #   [min]        [min]   [mAU*s]     [mAU]       %
----|-------|----|-------|----------|----------|--------|
   1   6.583 MM    0.0898 6893.86963 1279.50305  95.2461
   2   6.923 MM    0.2076  344.08289   27.62641   4.7539

Totals :                  7237.95251 1307.12946
```

Compound 10b

```
Acq. Operator   : BY                              Seq. Line :    1
Acq. Instrument : 1260R                            Location :   33
Injection Date  : 4/12/2024 6:36:23 PM                   Inj :    1
                                                 Inj Volume : 20.000 µl
Different Inj Volume from Sample Entry!  Actual Inj Volume : 10.000 µl
Acq. Method     : D:\Data\ZLM\DATA\20240412\20240412 2024-04-12 18-34-58\20240316--ZK-15MIN-
                  35-95ACN.M
Last changed    : 4/12/2024 12:05:51 AM by BY
Analysis Method : D:\DATA\KUN\METHOD\20240204-ZK-15MIN-25-90ACN.M
Last changed    : 4/12/2024 4:57:03 PM by BY
                  (modified after loading)
Method Info     : 90% H2O and 10% ACE

Additional Info : Peak(s) manually integrated
```

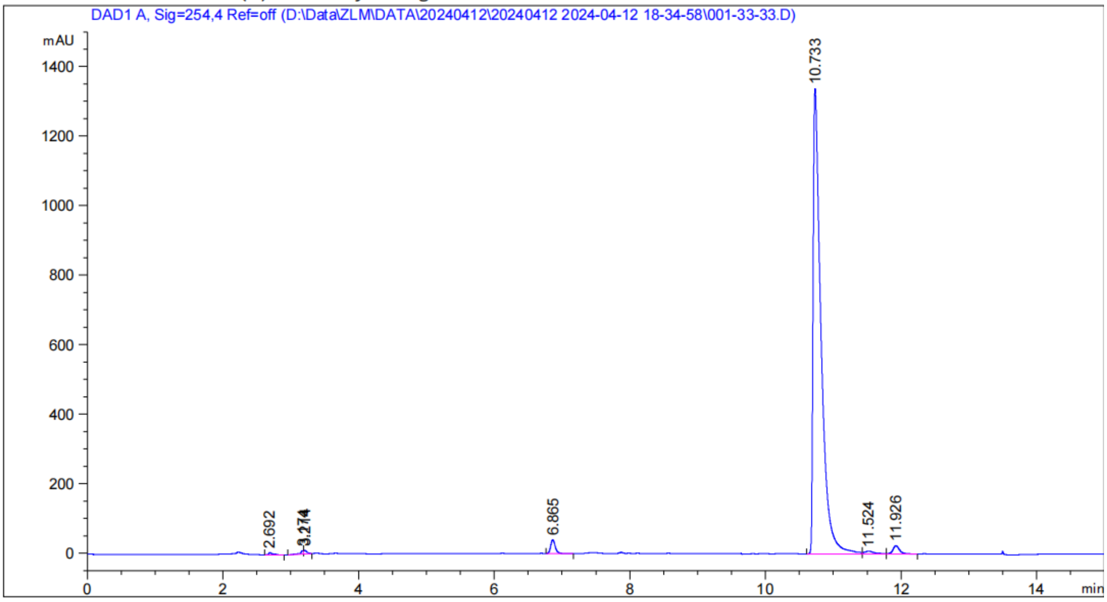


```
=====================================================================
                         Area Percent Report
=====================================================================

Sorted By             :      Signal
Multiplier            :      1.0000
Dilution              :      1.0000
Do not use Multiplier & Dilution Factor with ISTDs


Signal 1: DAD1 A, Sig=254,4 Ref=off
```

Peak #	RetTime [min]	Type	Width [min]	Area [mAU*s]	Height [mAU]	Area %
1	2.692	VB	0.0677	36.18795	7.13532	0.3165
2	3.174	BV	0.0472	31.69120	9.43974	0.2772
3	3.214	VB	0.0472	34.31916	10.20702	0.3001

Peak #	RetTime [min]	Type	Width [min]	Area [mAU*s]	Height [mAU]	Area %
4	6.865	VB	0.0714	191.38441	40.68355	1.6737
5	10.733	BV	0.1159	1.08908e4	1338.75342	95.2437
6	11.524	VV	0.1440	86.61273	8.24843	0.7575
7	11.926	VB	0.1024	163.66512	24.05088	1.4313

Totals : 1.14346e4 1438.51837

Compound 10c

```
Acq. Operator   : BY                              Seq. Line :   1
Acq. Instrument : 1260R                            Location :   3
Injection Date  : 4/12/2024 12:09:00 AM                 Inj :   1
                                                Inj Volume : 20.000 µl
Acq. Method     : D:\Data\ZXD\DATA\20240411-ZXD-1\zxd-1F_LC 2024-04-12 00-07-33\20240316--ZK-
                  15MIN-35-95ACN.M
Last changed    : 4/12/2024 12:05:51 AM by BY
Analysis Method : D:\DATA\KUN\METHOD\20240204-ZK-15MIN-25-90ACN.M
Last changed    : 4/12/2024 3:25:38 PM by BY
                  (modified after loading)
Method Info     : 90% H2O and 10% ACE

Additional Info : Peak(s) manually integrated
```

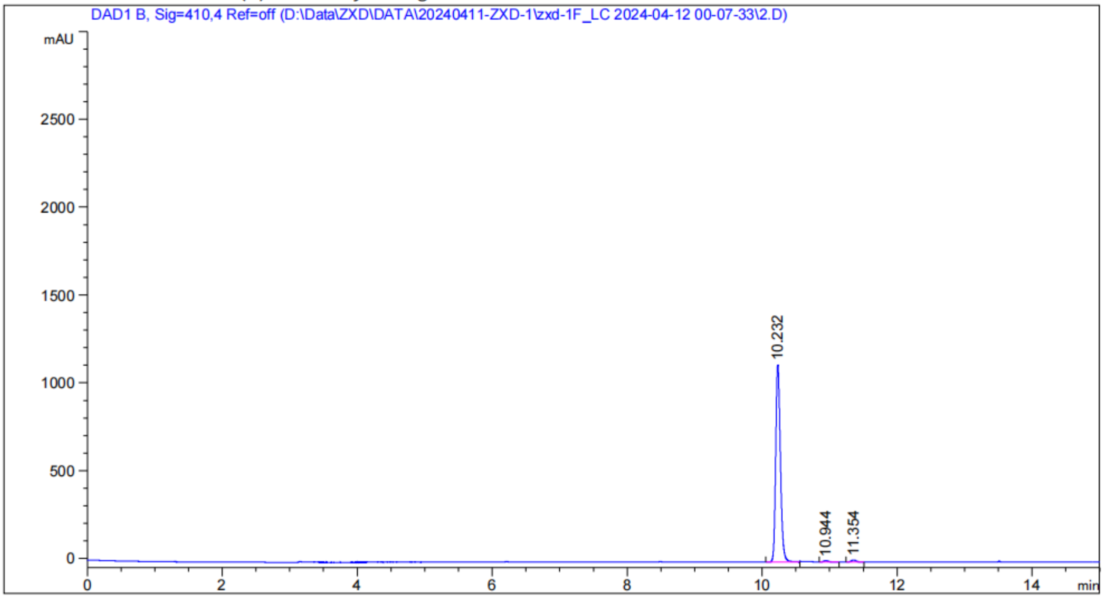


```
=====================================================================
                         Area Percent Report
=====================================================================

Sorted By             :      Signal
Multiplier            :      1.0000
Dilution              :      1.0000
Do not use Multiplier & Dilution Factor with ISTDs


Signal 1: DAD1 B, Sig=410,4 Ref=off
```

Peak #	RetTime [min]	Type	Width [min]	Area [mAU*s]	Height [mAU]	Area %
1	10.232	BV	0.0746	5452.07373	1124.41870	98.2411
2	10.944	VB	0.0650	39.60649	7.63603	0.7137
3	11.354	VB	0.0640	58.00705	11.66171	1.0452

Totals : 5549.68727 1143.71644

Compound 10d

```
Acq. Operator   : BY                             Seq. Line :   1
Acq. Instrument : 1260R                           Location :   61
Injection Date  : 4/18/2024 8:27:22 PM                 Inj :   1
                                                Inj Volume : 20.000 µl
Different Inj Volume from Sample Entry!  Actual Inj Volume : 10.000 µl
Acq. Method     : D:\Data\ZLM\DATA\20240416-2\20240416 2024-04-18 20-25-58\20240316--ZK-15MIN
                  -35-95ACN.M
Last changed    : 4/12/2024 12:05:51 AM by BY
Analysis Method : D:\DATA\ZLM\METHOD\AB-ACN-H2O(5-90)15MIN.M
Last changed    : 4/19/2024 12:04:54 AM by BY
                  (modified after loading)
Method Info     : HYDRO RP-80A

Additional Info : Peak(s) manually integrated
```

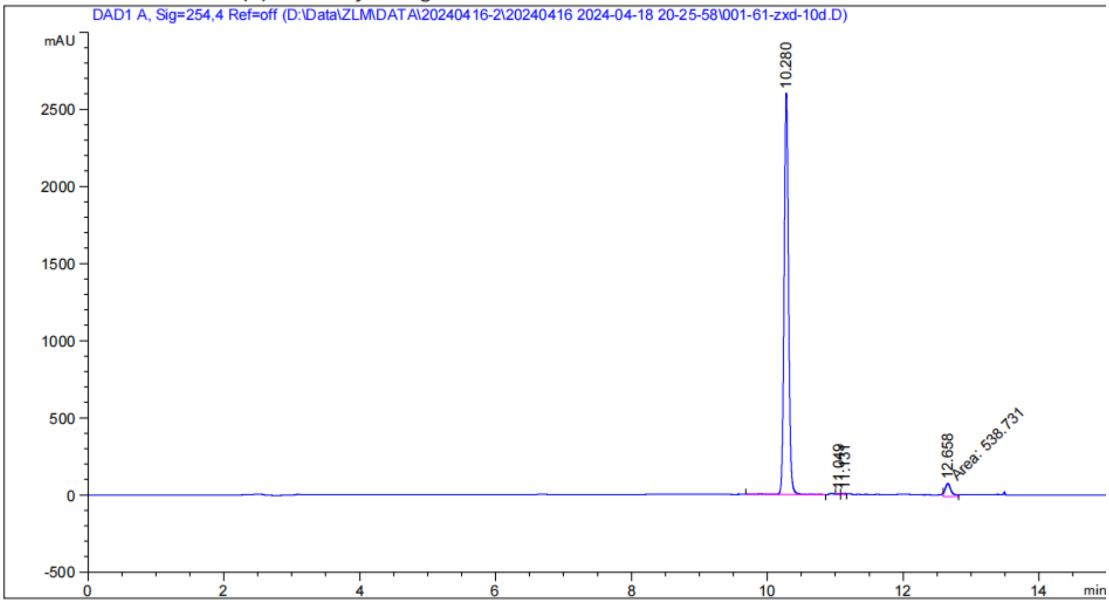


```
=====================================================================
                         Area Percent Report
=====================================================================

Sorted By             :      Signal
Multiplier            :      1.0000
Dilution              :      1.0000
Do not use Multiplier & Dilution Factor with ISTDs


Signal 1: DAD1 A, Sig=254,4 Ref=off

Peak RetTime Type  Width     Area      Height     Area
  #   [min]        [min]   [mAU*s]     [mAU]       %
----|-------|----|-------|----------|----------|--------|
   1  10.280 VV R  0.0686 1.17049e4  2605.04297  95.2729
   2  11.049 BV    0.0424    8.24244    2.79510   0.0671
   3  11.131 VV    0.0580   33.78830    8.39511   0.2750
Peak RetTime Type  Width     Area      Height     Area
  #   [min]        [min]   [mAU*s]     [mAU]       %
----|-------|----|-------|----------|----------|--------|
   4  12.658 MM    0.1045  538.73102   85.89119   4.3850

Totals :                   1.22857e4  2702.12437
```

Compound 10e

```
Acq. Operator   : BY                          Seq. Line :   4
Acq. Instrument : 1260R                        Location :   6
Injection Date  : 4/12/2024 12:58:25 AM             Inj :   1
                                             Inj Volume : 20.000 µl
Acq. Method     : D:\Data\ZXD\DATA\20240411-ZXD-1\zxd-1F_LC 2024-04-12 00-07-33\20240316--ZK-
                  15MIN-35-95ACN.M
Last changed    : 4/12/2024 12:05:51 AM by BY
Analysis Method : D:\DATA\KUN\METHOD\20240204-ZK-15MIN-25-90ACN.M
Last changed    : 4/12/2024 3:37:06 PM by BY
                  (modified after loading)
Method Info     : 90% H2O and 10% ACE

Additional Info : Peak(s) manually integrated
```

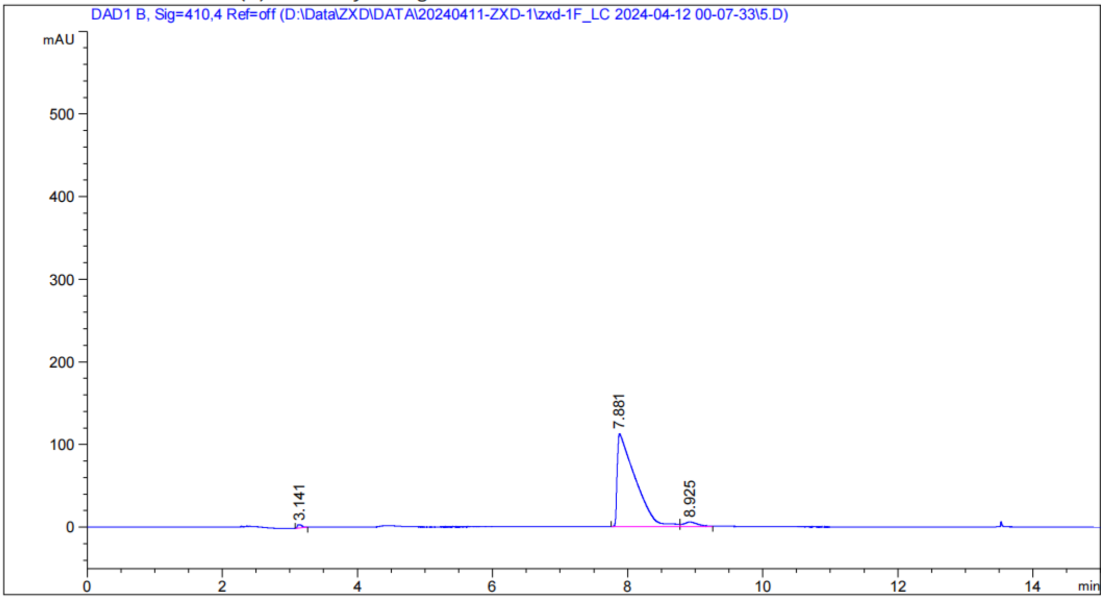


```
=====================================================================
                         Area Percent Report
=====================================================================

Sorted By             :      Signal
Multiplier            :      1.0000
Dilution              :      1.0000
Do not use Multiplier & Dilution Factor with ISTDs


Signal 1: DAD1 B, Sig=410,4 Ref=off
```

Peak #	RetTime [min]	Type	Width [min]	Area [mAU*s]	Height [mAU]	Area %
1	3.141	BB	0.0533	18.00039	4.05547	0.8259
2	7.881	BV	0.2354	2085.56421	112.41651	95.6857
3	8.925	VV	0.1683	76.03349	5.36567	3.4884

Totals : 2179.59809 121.83766

Compound 10f

```
Acq. Operator   : BY                            Seq. Line :   5
Acq. Instrument : 1260R                          Location :   7
Injection Date  : 4/12/2024 1:14:58 AM                Inj :   1
                                               Inj Volume : 20.000 µl
Acq. Method     : D:\Data\ZXD\DATA\20240411-ZXD-1\zxd-1F_LC 2024-04-12 00-07-33\20240316--ZK-
                  15MIN-35-95ACN.M
Last changed    : 4/12/2024 12:05:51 AM by BY
Analysis Method : D:\DATA\KUN\METHOD\20240204-ZK-15MIN-25-90ACN.M
Last changed    : 4/12/2024 3:38:15 PM by BY
                  (modified after loading)
Method Info     : 90% H2O and 10% ACE

Additional Info : Peak(s) manually integrated
```

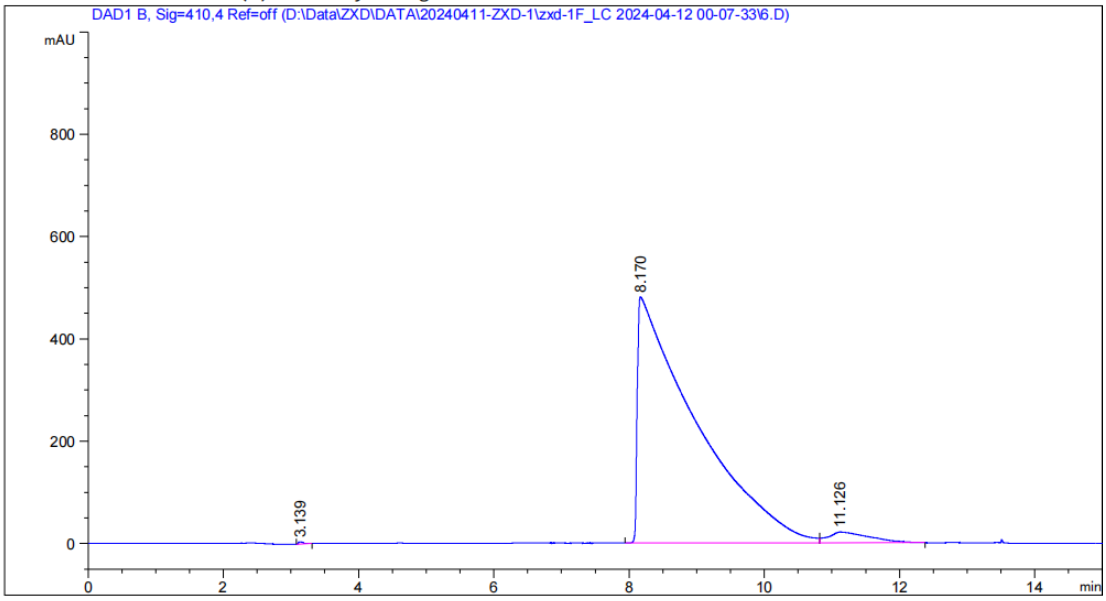


```
=====================================================================
                         Area Percent Report
=====================================================================

Sorted By             :      Signal
Multiplier            :      1.0000
Dilution              :      1.0000
Do not use Multiplier & Dilution Factor with ISTDs


Signal 1: DAD1 B, Sig=410,4 Ref=off
```

Peak #	RetTime [min]	Type	Width [min]	Area [mAU*s]	Height [mAU]	Area %
1	3.139	BB	0.0618	20.29373	4.17090	0.0684
2	8.170	BV	0.7097	2.87768e4	481.19464	96.9540
3	11.126	VB	0.4901	883.77112	21.08182	2.9776

Totals : 2.96808e4 506.44736

Compound 10g

```
Acq. Operator   : BY                            Seq. Line :   3
Acq. Instrument : 1260R                          Location :   35
Injection Date  : 4/12/2024 7:09:12 PM                Inj :   1
                                               Inj Volume : 20.000 µl
Different Inj Volume from Sample Entry!  Actual Inj Volume : 10.000 µl
Acq. Method     : D:\Data\ZLM\DATA\20240412\20240412 2024-04-12 18-34-58\20240316--ZK-15MIN-
                  35-95ACN.M
Last changed    : 4/12/2024 12:05:51 AM by BY
Analysis Method : D:\DATA\KUN\METHOD\20240204-ZK-15MIN-25-90ACN.M
Last changed    : 4/12/2024 4:57:03 PM by BY
                  (modified after loading)
Method Info     : 90% H2O and 10% ACE

Additional Info : Peak(s) manually integrated
```

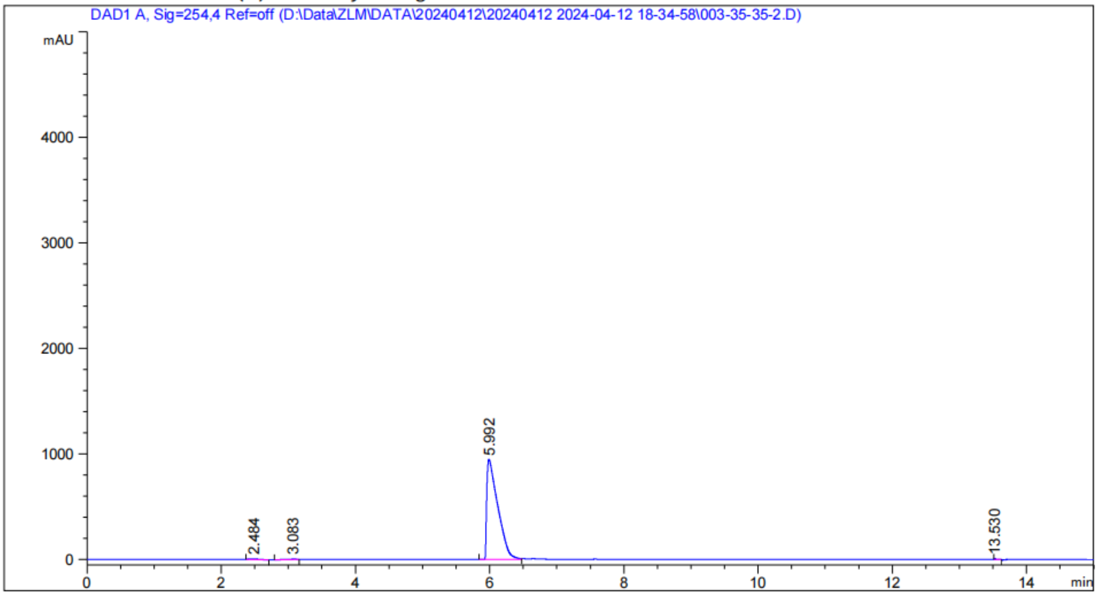

===

Area Percent Report

===

```
Sorted By             :      Signal
Multiplier            :      1.0000
Dilution              :      1.0000
Do not use Multiplier & Dilution Factor with ISTDs
```

Signal 1: DAD1 A, Sig=254,4 Ref=off

Peak #	RetTime [min]	Type	Width [min]	Area [mAU*s]	Height [mAU]	Area %
1	2.484	VB	0.1122	61.26885	6.51511	0.5600
2	3.083	BV	0.0836	37.81834	6.03738	0.3456
3	5.992	BV	0.1579	1.08280e4	947.29718	98.9644

Peak #	RetTime [min]	Type	Width [min]	Area [mAU*s]	Height [mAU]	Area %
4	13.530	VB	0.0191	14.22219	10.66458	0.1300

Totals : 1.09414e4 970.51425

Compound 10h

```
Acq. Operator   : BY                              Seq. Line :   2
Acq. Instrument : 1260R                            Location :   34
Injection Date  : 4/12/2024 6:52:48 PM                  Inj :   1
                                                 Inj Volume : 20.000 µl
Different Inj Volume from Sample Entry!  Actual Inj Volume : 10.000 µl
Acq. Method     : D:\Data\ZLM\DATA\20240412\20240412 2024-04-12 18-34-58\20240316--ZK-15MIN-
                  35-95ACN.M
Last changed    : 4/12/2024 12:05:51 AM by BY
Analysis Method : D:\DATA\KUN\METHOD\20240204-ZK-15MIN-25-90ACN.M
Last changed    : 4/12/2024 4:57:03 PM by BY
                  (modified after loading)
Method Info     : 90% H2O and 10% ACE

Additional Info : Peak(s) manually integrated
```

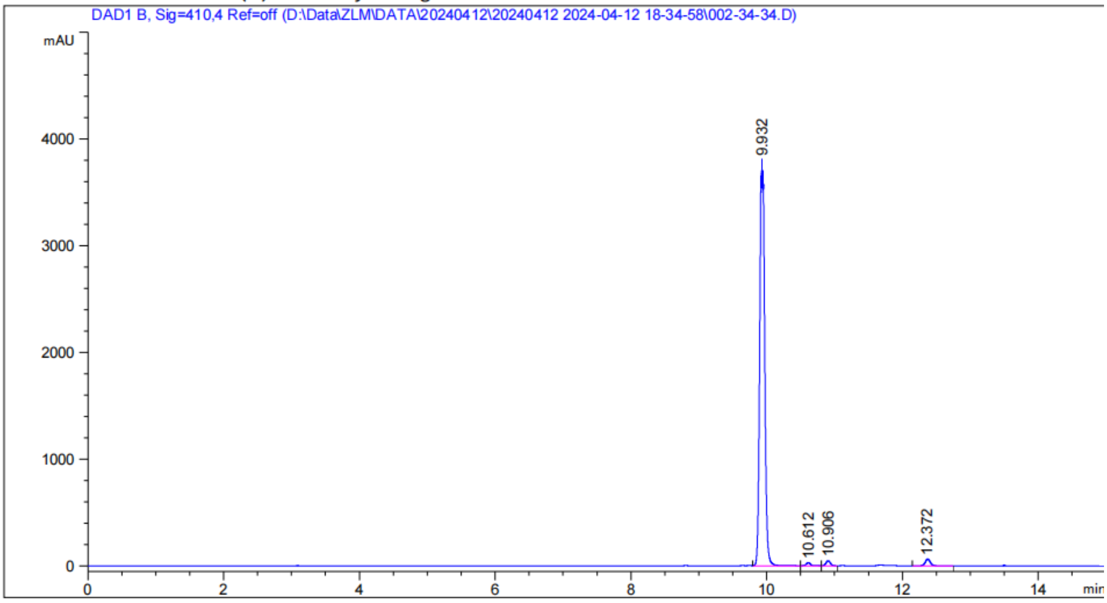

Area Percent Report

```
Sorted By             :      Signal
Multiplier            :      1.0000
Dilution              :      1.0000
Do not use Multiplier & Dilution Factor with ISTDs
```

Signal 1: DAD1 B, Sig=410,4 Ref=off

Peak #	RetTime [min]	Type	Width [min]	Area [mAU*s]	Height [mAU]	Area %
1	9.932	VV	0.0605	1.92137e4	3798.98242	96.4796
2	10.612	VV	0.0760	152.79659	29.94452	0.7673
3	10.906	VV	0.0675	218.24248	48.99380	1.0959

Peak #	RetTime [min]	Type	Width [min]	Area [mAU*s]	Height [mAU]	Area %
4	12.372	BB	0.0793	330.03577	63.31224	1.6572

Totals : 1.99147e4 3941.23297

Compound 10i

```
Acq. Operator   : BY                              Seq. Line :   1
Acq. Instrument : 1260R                            Location :   62
Injection Date  : 4/18/2024 11:27:30 PM                 Inj :   1
                                                 Inj Volume : 20.000 µl
Different Inj Volume from Sample Entry!  Actual Inj Volume : 1.500 µl
Acq. Method     : D:\Data\ZLM\DATA\20240416-2\20240416 2024-04-18 23-25-24\20240316--ZK-15MIN
                  -35-95ACN.M
Last changed    : 4/12/2024 12:05:51 AM by BY
Analysis Method : D:\DATA\ZLM\METHOD\AB-ACN-H2O(5-90)15MIN.M
Last changed    : 4/18/2024 11:55:56 PM by BY
                  (modified after loading)
Method Info     : HYDRO RP-80A

Additional Info : Peak(s) manually integrated
```

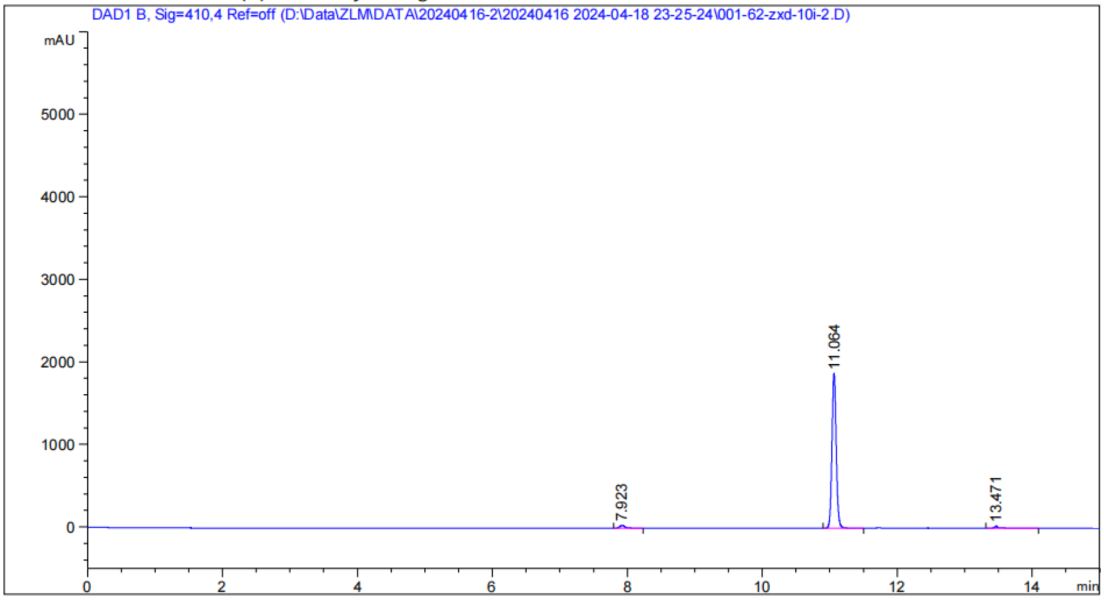


```
=====================================================================
                         Area Percent Report
=====================================================================

Sorted By             :      Signal
Multiplier            :      1.0000
Dilution              :      1.0000
Do not use Multiplier & Dilution Factor with ISTDs


Signal 1: DAD1 B, Sig=410,4 Ref=off
```

Peak #	RetTime [min]	Type	Width [min]	Area [mAU*s]	Height [mAU]	Area %
1	7.923	BB	0.0808	190.41331	35.11571	2.2311
2	11.064	BB	0.0676	8221.08691	1875.74304	96.3270
3	13.471	BB	0.0529	123.06314	29.38262	1.4419
Totals :				8534.56337	1940.24137	

Compound 10j

```
Acq. Operator   : BY                              Seq. Line :   2
Acq. Instrument : 1260R                            Location :   63
Injection Date  : 4/18/2024 11:43:52 PM                 Inj :   1
                                                 Inj Volume : 20.000 µl
Different Inj Volume from Sample Entry!  Actual Inj Volume : 1.500 µl
Acq. Method     : D:\Data\ZLM\DATA\20240416-2\20240416 2024-04-18 23-25-24\20240316--ZK-15MIN
                  -35-95ACN.M
Last changed    : 4/12/2024 12:05:51 AM by BY
Analysis Method : D:\DATA\ZLM\METHOD\AB-ACN-H2O(5-90)15MIN.M
Last changed    : 4/19/2024 12:10:27 AM by BY
                  (modified after loading)
Method Info     : HYDRO RP-80A

Additional Info : Peak(s) manually integrated
```

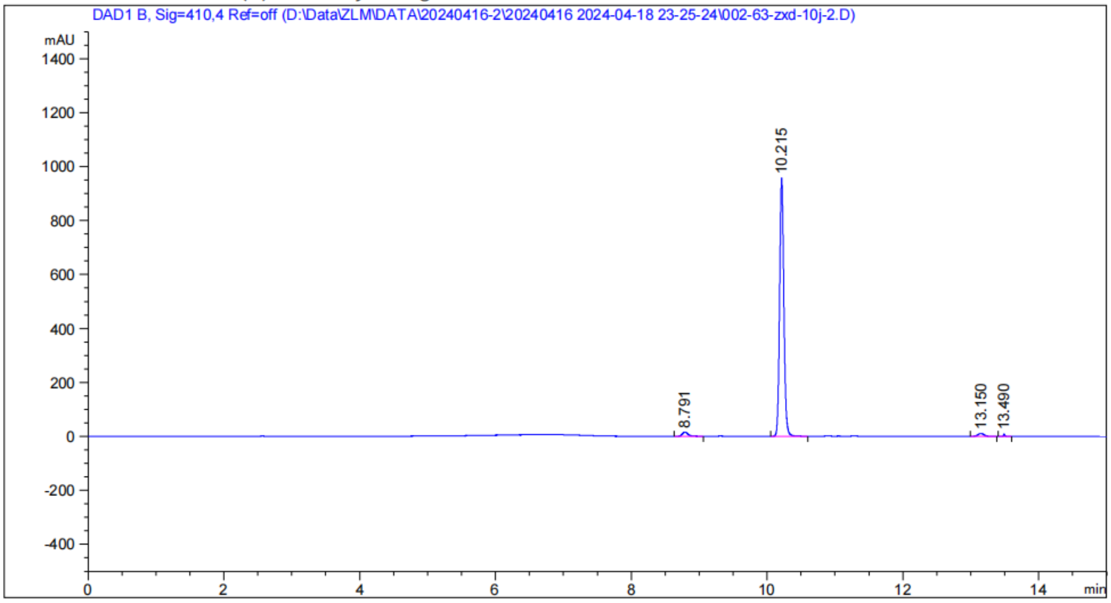


```
=====================================================================
                         Area Percent Report
=====================================================================

Sorted By             :      Signal
Multiplier            :      1.0000
Dilution              :      1.0000
Do not use Multiplier & Dilution Factor with ISTDs


Signal 1: DAD1 B, Sig=410,4 Ref=off
```

Peak #	RetTime [min]	Type	Width [min]	Area [mAU*s]	Height [mAU]	Area %
1	8.791	BB	0.0831	87.31108	15.64773	2.0258
2	10.215	BB	0.0666	4148.13672	956.85345	96.2472
3	13.150	BB	0.0822	64.82144	10.93350	1.5040

Peak #	RetTime [min]	Type	Width [min]	Area [mAU*s]	Height [mAU]	Area %
4	13.490	BB	0.0177	9.60909	7.88929	0.2230

Totals : 4309.87833 991.32397

Compound 10k

```
Acq. Operator   : BY                              Seq. Line :    1
Acq. Instrument : 1260R                            Location :   71
Injection Date  : 4/19/2024 4:11:46 AM                  Inj :    1
                                                Inj Volume : 20.000 µl
Different Inj Volume from Sample Entry!  Actual Inj Volume : 3.000 µl
Acq. Method     : D:\Data\ZLM\DATA\20240416-2\20240416 2024-04-19 04-10-26\20240316--ZK-15MIN
                  -35-95ACN.M
Last changed    : 4/12/2024 12:05:51 AM by BY
Analysis Method : D:\DATA\ZLM\METHOD\AB-ACN-H2O(5-90)15MIN.M
Last changed    : 4/19/2024 11:11:40 AM by BY
                  (modified after loading)
Method Info     : HYDRO RP-80A
```

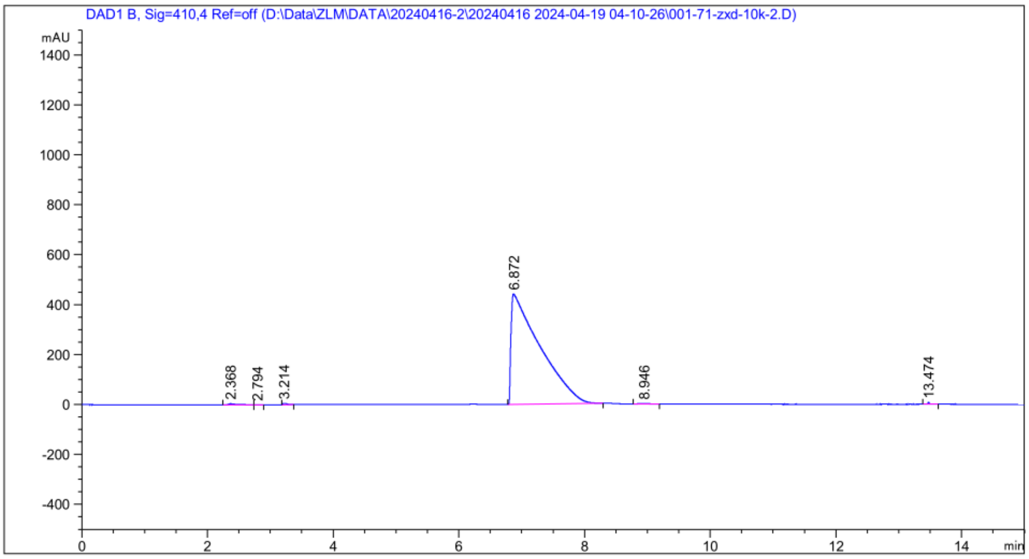

===

Area Percent Report

===

```
Sorted By             :      Signal
Multiplier            :      1.0000
Dilution              :      1.0000
Do not use Multiplier & Dilution Factor with ISTDs
```

Signal 1: DAD1 B, Sig=410,4 Ref=off

Peak #	RetTime [min]	Type	Width [min]	Area [mAU*s]	Height [mAU]	Area %
1	2.368	BV	0.1310	50.60454	4.54075	0.3572
2	2.794	VB	0.0616	6.85166	1.32963	0.0484
3	3.214	BB	0.0673	26.18634	5.52393	0.1849
4	6.872	BB	0.3858	1.40456e4	440.78781	99.1536
5	8.946	BB	0.1243	25.36696	2.41083	0.1791
6	13.474	BB	0.0197	10.89408	8.09205	0.0769

Totals : 1.41655e4 462.68499

Compound 10l

```
Acq. Operator   : BY                                Seq. Line :  11
Acq. Instrument : 1260R                              Location :   13
Injection Date  : 4/12/2024 2:54:03 AM                    Inj :   1
                                                   Inj Volume : 20.000 µl
Acq. Method     : D:\Data\ZXD\DATA\20240411-ZXD-1\zxd-1F_LC 2024-04-12 00-07-33\20240316--ZK-
                  15MIN-35-95ACN.M
Last changed    : 4/12/2024 12:05:51 AM by BY
Analysis Method : D:\DATA\ZLM\METHOD\AB-ACN-H2O(5-90)15MIN.M
Last changed    : 4/19/2024 11:32:58 AM by BY
                  (modified after loading)
Method Info     : HYDRO RP-80A

Additional Info : Peak(s) manually integrated
```

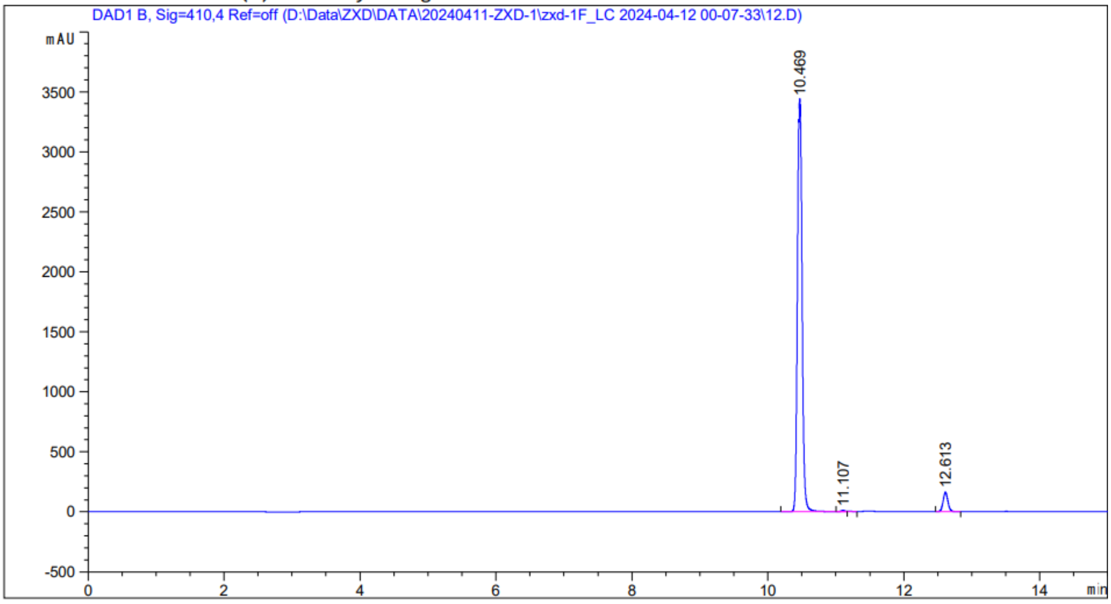

===

Area Percent Report

===

```
Sorted By             :      Signal
Multiplier            :      1.0000
Dilution              :      1.0000
Do not use Multiplier & Dilution Factor with ISTDs
```

Signal 1: DAD1 B, Sig=410,4 Ref=off

Peak #	RetTime [min]	Type	Width [min]	Area [mAU*s]	Height [mAU]	Area %
1	10.469	BV R	0.0574	1.64379e4	3429.56763	95.1396
2	11.107	VV E	0.0617	40.21786	9.53596	0.2328
3	12.613	BB	0.0761	799.54175	161.86668	4.6276

Totals : 1.72777e4 3600.97027

Compound 10m

Acq. Operator : BY Seq. Line : 3
Acq. Instrument : 1260R Location : 64
Injection Date : 4/19/2024 12:00:14 AM Inj : 1
Inj Volume : 20.000 µl
Different Inj Volume from Sample Entry! Actual Inj Volume : 1.500 µl
Acq. Method : D:\Data\ZLM\DATA\20240416-2\20240416 2024-04-18 23-25-24\20240316--ZK-15MIN-35-95ACN.M
Last changed : 4/12/2024 12:05:51 AM by BY
Analysis Method : D:\DATA\ZLM\METHOD\AB-ACN-H2O(5-90)15MIN.M
Last changed : 4/19/2024 12:34:52 AM by BY
(modified after loading)
Method Info : HYDRO RP-80A

Additional Info : Peak(s) manually integrated

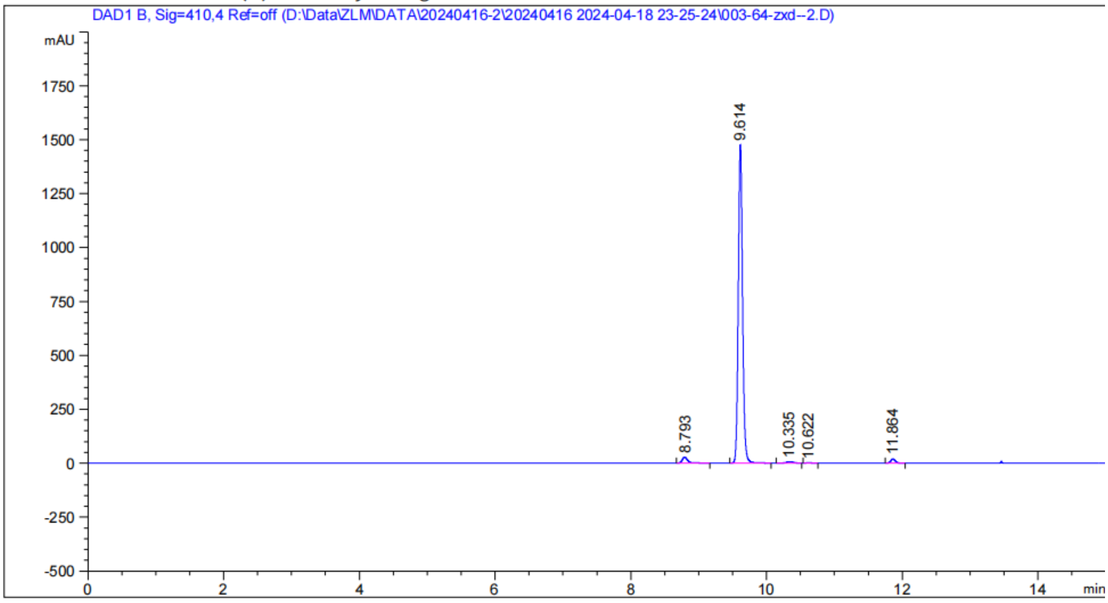

===
Area Percent Report
===

Sorted By : Signal
Multiplier : 1.0000
Dilution : 1.0000
Do not use Multiplier & Dilution Factor with ISTDs

Signal 1: DAD1 B, Sig=410,4 Ref=off

Peak #	RetTime [min]	Type	Width [min]	Area [mAU*s]	Height [mAU]	Area %
1	8.793	BV R	0.0820	151.90813	27.70000	2.2502
2	9.614	BB	0.0671	6456.26367	1475.33154	95.6360
3	10.335	BB	0.0835	44.61168	6.34394	0.6608
4	10.622	BB	0.0545	8.94564	2.04806	0.1325
5	11.864	BB	0.0708	89.14001	19.15693	1.3204

Totals : 6750.86913 1530.58047

Compound 10n

```
Acq. Operator   : BY                              Seq. Line :   4
Acq. Instrument : 1260R                            Location :   65
Injection Date  : 4/19/2024 12:16:38 AM                 Inj :   1
                                                Inj Volume : 20.000 µl
Different Inj Volume from Sample Entry!  Actual Inj Volume : 1.500 µl
Acq. Method     : D:\Data\ZLM\DATA\20240416-2\20240416 2024-04-18 23-25-24\20240316--ZK-15MIN
                  -35-95ACN.M
Last changed    : 4/12/2024 12:05:51 AM by BY
Analysis Method : D:\DATA\ZLM\METHOD\AB-ACN-H2O(5-90)15MIN.M
Last changed    : 4/19/2024 12:36:24 AM by BY
                  (modified after loading)
Method Info     : HYDRO RP-80A

Additional Info : Peak(s) manually integrated
```

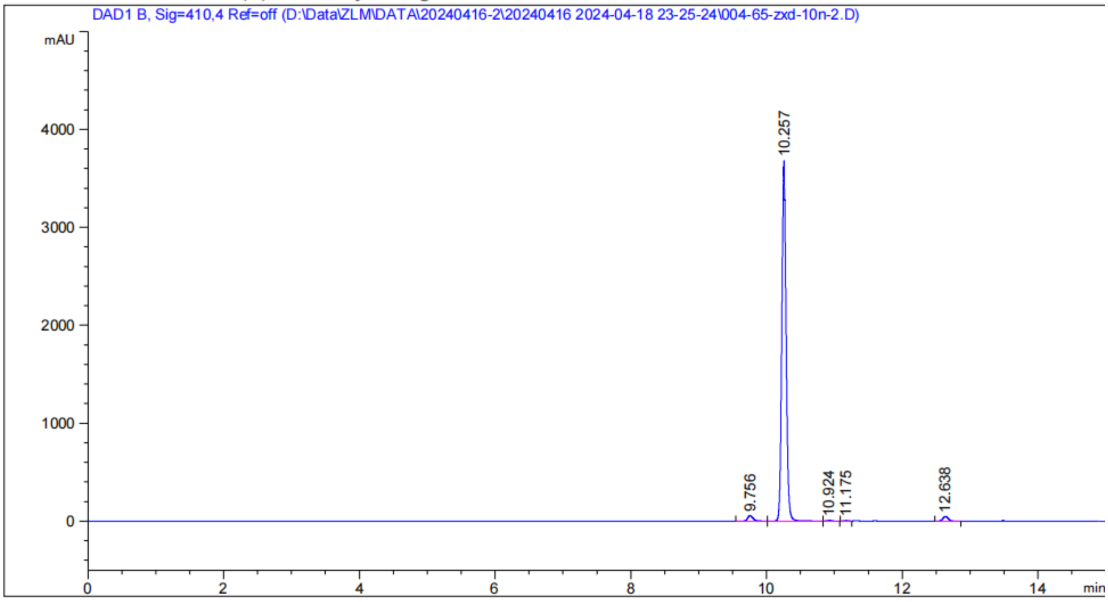

===
Area Percent Report
===

```
Sorted By             :      Signal
Multiplier            :      1.0000
Dilution              :      1.0000
Do not use Multiplier & Dilution Factor with ISTDs
```

Signal 1: DAD1 B, Sig=410,4 Ref=off

Peak #	RetTime [min]	Type	Width [min]	Area [mAU*s]	Height [mAU]	Area %
1	9.756	BB	0.0815	303.81830	56.73564	1.8214
2	10.257	VV R	0.0525	1.60662e4	3675.09253	96.3192
3	10.924	VB	0.0586	31.70495	7.23587	0.1901

Peak #	RetTime [min]	Type	Width [min]	Area [mAU*s]	Height [mAU]	Area %
4	11.175	BV	0.0543	23.94740	5.72691	0.1436
5	12.638	BB	0.0809	254.49269	47.97326	1.5257

Totals : 1.66801e4 3792.76421

Compound 11a

```
Acq. Operator   : BY                              Seq. Line :   2
Acq. Instrument : 1260R                            Location :   72
Injection Date  : 4/19/2024 4:28:04 AM                  Inj :   1
                                                 Inj Volume : 20.000 µl
Different Inj Volume from Sample Entry!  Actual Inj Volume : 1.500 µl
Acq. Method     : D:\Data\ZLM\DATA\20240416-2\20240416 2024-04-19 04-10-26\20240316--ZK-15MIN
                  -35-95ACN.M
Last changed    : 4/12/2024 12:05:51 AM by BY
Analysis Method : D:\DATA\ZLM\METHOD\AB-ACN-H2O(5-90)15MIN.M
Last changed    : 4/19/2024 11:12:29 AM by BY
                  (modified after loading)
Method Info     : HYDRO RP-80A

Additional Info : Peak(s) manually integrated
```

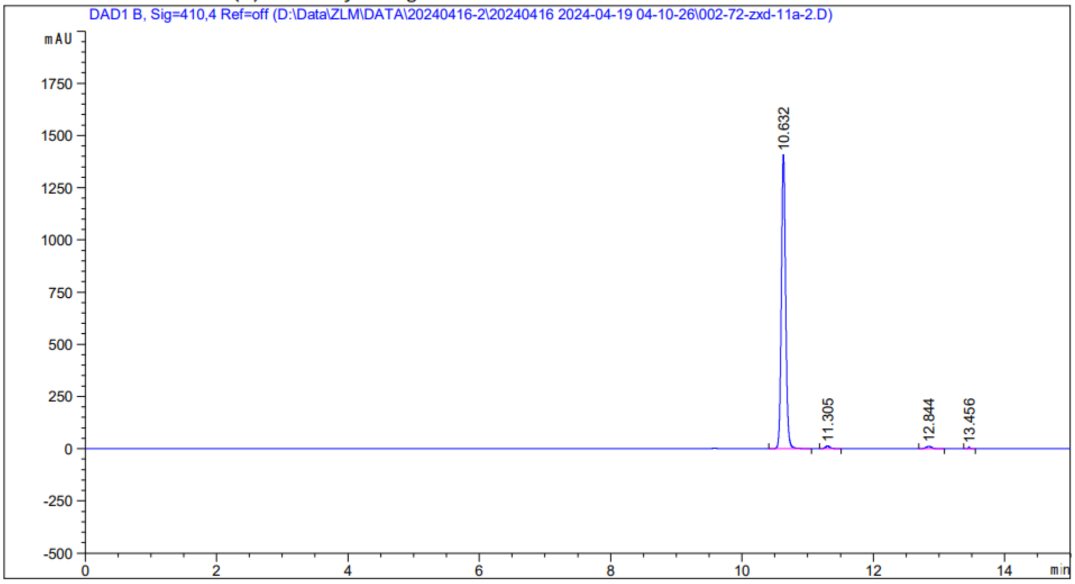

===

Area Percent Report

===

Sorted By : Signal
Multiplier : 1.0000
Dilution : 1.0000
Do not use Multiplier & Dilution Factor with ISTDs

Signal 1: DAD1 B, Sig=410,4 Ref=off

Peak #	RetTime [min]	Type	Width [min]	Area [mAU*s]	Height [mAU]	Area %
1	10.632	BB	0.0697	6353.18408	1407.23389	97.9894
2	11.305	BB	0.0699	59.02020	12.66451	0.9103
3	12.844	BB	0.0813	61.69806	10.77266	0.9516

Peak #	RetTime [min]	Type	Width [min]	Area [mAU*s]	Height [mAU]	Area %
4	13.456	BB	0.0176	9.63782	7.97042	0.1487

Totals : 6483.54016 1438.64147

Compound 11b

```
Acq. Operator   : BY                              Seq. Line :    3
Acq. Instrument : 1260R                            Location :   73
Injection Date  : 4/19/2024 4:44:23 AM                  Inj :    1
                                                 Inj Volume : 20.000 µl
Different Inj Volume from Sample Entry!  Actual Inj Volume : 3.000 µl
Acq. Method     : D:\Data\ZLM\DATA\20240416-2\20240416 2024-04-19 04-10-26\20240316--ZK-15MIN
                  -35-95ACN.M
Last changed    : 4/12/2024 12:05:51 AM by BY
Analysis Method : D:\DATA\ZLM\METHOD\AB-ACN-H2O(5-90)15MIN.M
Last changed    : 4/19/2024 11:13:55 AM by BY
                  (modified after loading)
Method Info     : HYDRO RP-80A

Additional Info : Peak(s) manually integrated
```

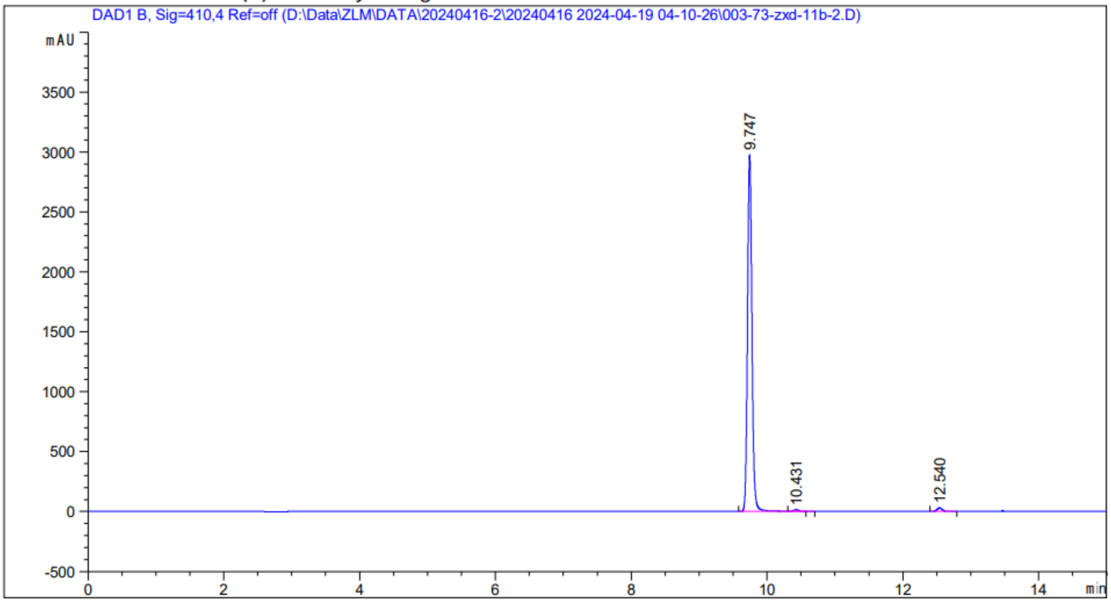


```
=====================================================================
                         Area Percent Report
=====================================================================

Sorted By             :      Signal
Multiplier            :      1.0000
Dilution              :      1.0000
Do not use Multiplier & Dilution Factor with ISTDs


Signal 1: DAD1 B, Sig=410,4 Ref=off
```

Peak #	RetTime [min]	Type	Width [min]	Area [mAU*s]	Height [mAU]	Area %
1	9.747	BV R	0.0566	1.35502e4	2978.04150	98.3951
2	10.431	VV E	0.0648	59.61275	13.29459	0.4329
3	12.540	BB	0.0808	161.39809	30.48005	1.1720
Totals :				1.37712e4	3021.81615	

Compound 11c

Acq. Operator : BY Seq. Line : 6
Acq. Instrument : 1260R Location : 66
Injection Date : 4/18/2024 9:49:31 PM Inj : 1
Inj Volume : 20.000 µl
Different Inj Volume from Sample Entry! Actual Inj Volume : 3.000 µl
Acq. Method : D:\Data\ZLM\DATA\20240416-2\20240416 2024-04-18 20-25-58\20240316--ZK-15MIN-35-95ACN.M
Last changed : 4/12/2024 12:05:51 AM by BY
Analysis Method : D:\DATA\ZLM\METHOD\AB-ACN-H2O(5-90)15MIN.M
Last changed : 4/19/2024 12:06:54 AM by BY
(modified after loading)
Method Info : HYDRO RP-80A

Additional Info : Peak(s) manually integrated

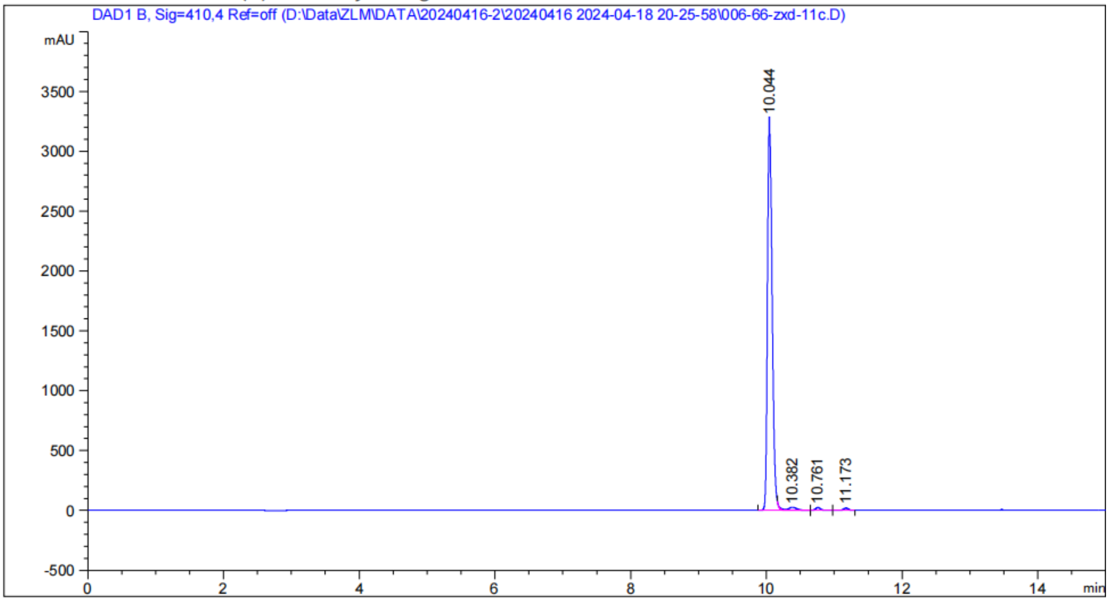

===
Area Percent Report
===

Sorted By : Signal
Multiplier : 1.0000
Dilution : 1.0000
Do not use Multiplier & Dilution Factor with ISTDs

Signal 1: DAD1 B, Sig=410,4 Ref=off

Peak #	RetTime [min]	Type	Width [min]	Area [mAU*s]	Height [mAU]	Area %
1	10.044	BV R	0.0599	1.57088e4	3277.49316	97.1021
2	10.382	VV E	0.1437	262.65686	25.49599	1.6236
3	10.761	VB	0.0753	115.70425	23.14659	0.7152
4	11.173	BV	0.0697	90.44169	18.61432	0.5591

Totals : 1.61776e4 3344.75007

Compound 11d

Acq. Operator : BY Seq. Line : 7
Acq. Instrument : 1260R Location : 67
Injection Date : 4/18/2024 10:05:56 PM Inj : 1
Inj Volume : 20.000 µl
Different Inj Volume from Sample Entry! Actual Inj Volume : 3.000 µl
Acq. Method : D:\Data\ZLM\DATA\20240416-2\20240416 2024-04-18 20-25-58\20240316--ZK-15MIN-35-95ACN.M
Last changed : 4/12/2024 12:05:51 AM by BY
Analysis Method : D:\DATA\ZLM\METHOD\AB-ACN-H2O(5-90)15MIN.M
Last changed : 4/19/2024 12:07:47 AM by BY
(modified after loading)
Method Info : HYDRO RP-80A

Additional Info : Peak(s) manually integrated

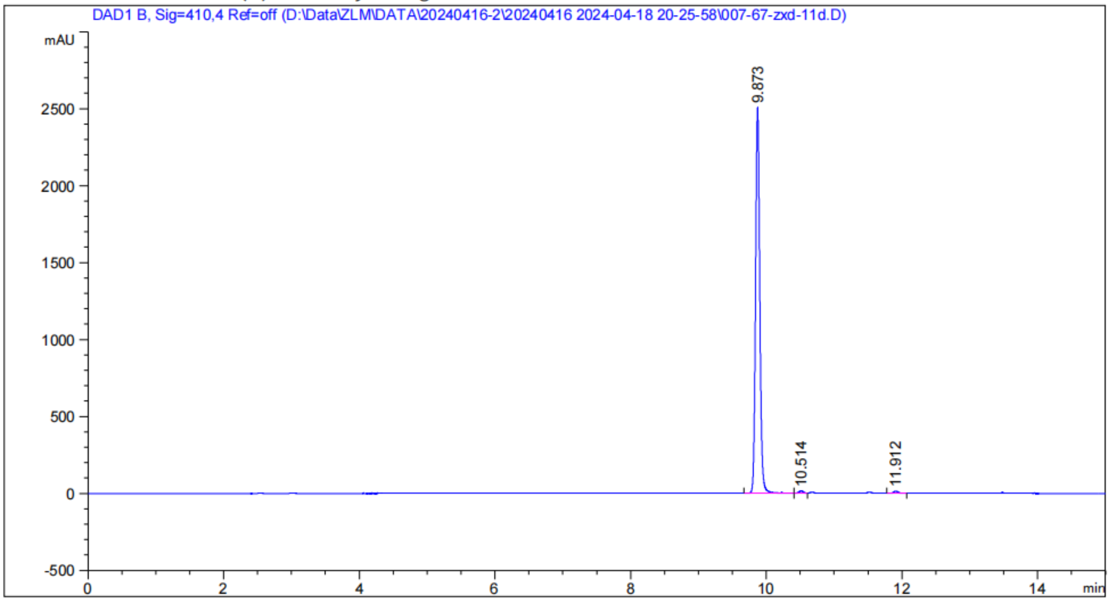

===
Area Percent Report
===

Sorted By : Signal
Multiplier : 1.0000
Dilution : 1.0000
Do not use Multiplier & Dilution Factor with ISTDs

Signal 1: DAD1 B, Sig=410,4 Ref=off

Peak #	RetTime [min]	Type	Width [min]	Area [mAU*s]	Height [mAU]	Area %
1	9.873	BV R	0.0673	1.09259e4	2505.33472	98.8624
2	10.514	BV	0.0652	64.60635	15.17704	0.5846
3	11.912	BV R	0.0671	61.11150	13.54739	0.5530

Peak #	RetTime [min]	Type	Width [min]	Area [mAU*s]	Height [mAU]	Area %
Totals :				1.10516e4	2534.05914	

Compound 11e

```
Acq. Operator   : BY                              Seq. Line :  18
Acq. Instrument : 1260R                            Location :   20
Injection Date  : 4/12/2024 4:49:46 AM                  Inj :   1
                                                Inj Volume : 20.000 µl
Acq. Method     : D:\Data\ZXD\DATA\20240411-ZXD-1\zxd-1F_LC 2024-04-12 00-07-33\20240316--ZK-
                  15MIN-35-95ACN.M
Last changed    : 4/12/2024 12:05:51 AM by BY
Analysis Method : D:\DATA\KUN\METHOD\20240204-ZK-15MIN-25-90ACN.M
Last changed    : 4/12/2024 4:21:22 PM by BY
                  (modified after loading)
Method Info     : 90% H2O and 10% ACE

Additional Info : Peak(s) manually integrated
```

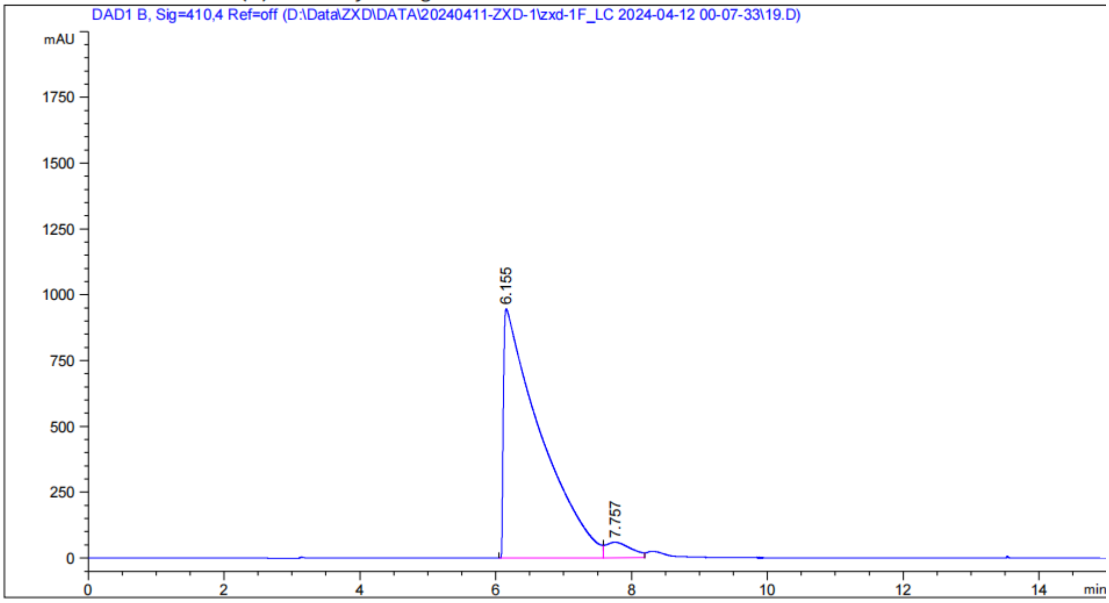


```
=====================================================================
                         Area Percent Report
=====================================================================

Sorted By             :      Signal
Multiplier            :      1.0000
Dilution              :      1.0000
Do not use Multiplier & Dilution Factor with ISTDs


Signal 1: DAD1 B, Sig=410,4 Ref=off

Peak RetTime Type  Width     Area      Height     Area
  #   [min]        [min]   [mAU*s]     [mAU]       %
----|-------|----|-------|----------|----------|--------|
   1   6.155 BV    0.4595 3.56240e4  945.72571  95.8358
   2   7.757 VV    0.3070 1547.92822   59.11162   4.1642

Totals :                  3.71719e4  1004.83733
```

Compound 11f

```
Acq. Operator   : BY                              Seq. Line :   4
Acq. Instrument : 1260R                            Location :   74
Injection Date  : 4/19/2024 5:00:49 AM                  Inj :   1
                                                 Inj Volume : 20.000 µl
Different Inj Volume from Sample Entry!  Actual Inj Volume : 3.000 µl
Acq. Method     : D:\Data\ZLM\DATA\20240416-2\20240416 2024-04-19 04-10-26\20240316--ZK-15MIN
                  -35-95ACN.M
Last changed    : 4/12/2024 12:05:51 AM by BY
Analysis Method : D:\DATA\ZLM\METHOD\AB-ACN-H2O(5-90)15MIN.M
Last changed    : 4/19/2024 11:14:57 AM by BY
                  (modified after loading)
Method Info     : HYDRO RP-80A

Additional Info : Peak(s) manually integrated
```

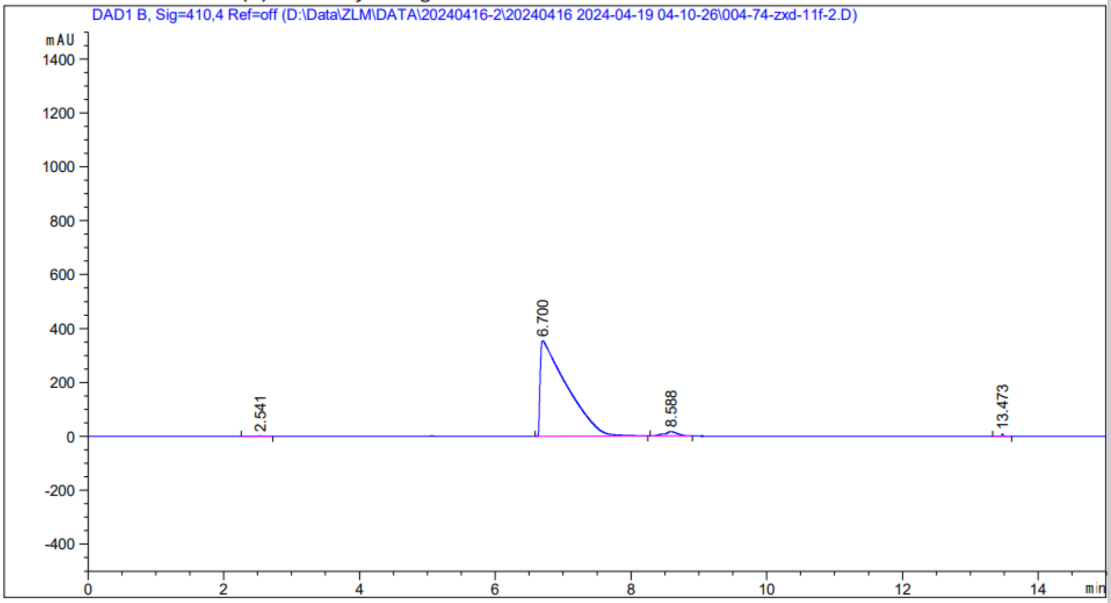

===
Area Percent Report
===

Sorted By : Signal
Multiplier : 1.0000
Dilution : 1.0000
Do not use Multiplier & Dilution Factor with ISTDs

Signal 1: DAD1 B, Sig=410,4 Ref=off

Peak #	RetTime [min]	Type	Width [min]	Area [mAU*s]	Height [mAU]	Area %
1	2.541	VB R	0.0602	13.54403	2.86515	0.1369
2	6.700	BB	0.3367	9647.68262	354.79312	97.5031
3	8.588	BB	0.1639	222.22571	16.79710	2.2459

Peak #	RetTime [min]	Type	Width [min]	Area [mAU*s]	Height [mAU]	Area %
4	13.473	BV R	0.0191	11.29014	8.42157	0.1141

Totals : 9894.74249 382.87693

Compound 11g

```
Acq. Operator   : BY                              Seq. Line :  20
Acq. Instrument : 1260R                            Location :   22
Injection Date  : 4/12/2024 5:22:38 AM                  Inj :   1
                                                 Inj Volume : 20.000 µl
Acq. Method     : D:\Data\ZXD\DATA\20240411-ZXD-1\zxd-1F_LC 2024-04-12 00-07-33\20240316--ZK-
                  15MIN-35-95ACN.M
Last changed    : 4/12/2024 12:05:51 AM by BY
Analysis Method : D:\DATA\KUN\METHOD\20240204-ZK-15MIN-25-90ACN.M
Last changed    : 4/12/2024 4:21:22 PM by BY
                  (modified after loading)
Method Info     : 90% H2O and 10% ACE

Additional Info : Peak(s) manually integrated
```

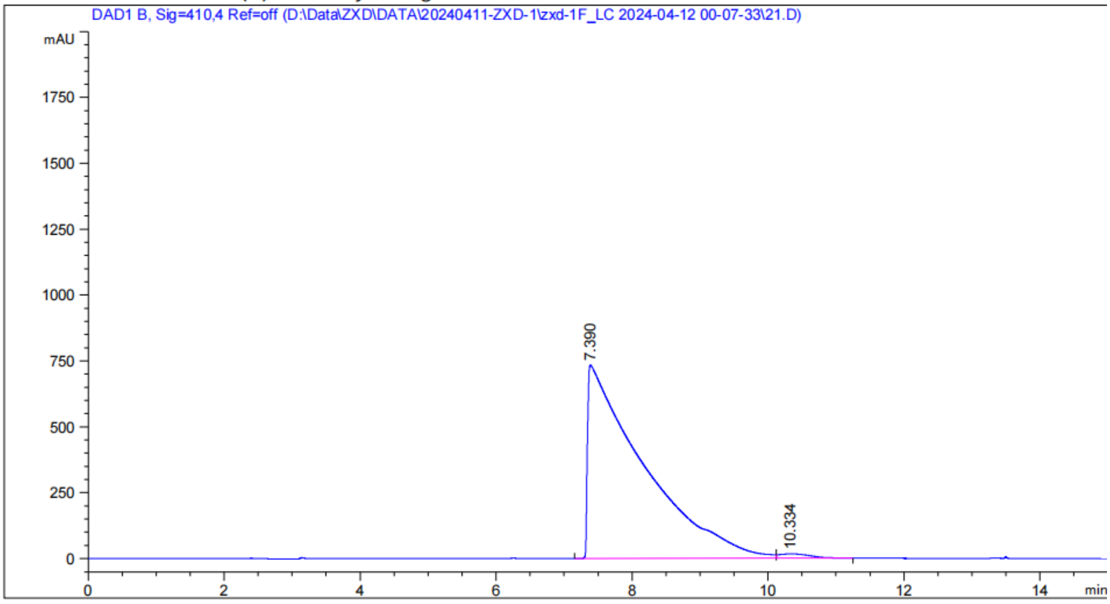


```
=====================================================================
                         Area Percent Report
=====================================================================

Sorted By             :      Signal
Multiplier            :      1.0000
Dilution              :      1.0000
Do not use Multiplier & Dilution Factor with ISTDs


Signal 1: DAD1 B, Sig=410,4 Ref=off
```

Peak #	RetTime [min]	Type	Width [min]	Area [mAU*s]	Height [mAU]	Area %
1	7.390	BV	0.6769	4.17285e4	733.30786	98.7609
2	10.334	VB	0.3660	523.53467	16.75860	1.2391

Totals : 4.22520e4 750.06646

Compound 11h

```
Acq. Operator   : BY                              Seq. Line :  21
Acq. Instrument : 1260R                            Location :   23
Injection Date  : 4/12/2024 5:39:09 AM                  Inj :   1
                                                 Inj Volume : 20.000 µl
Acq. Method     : D:\Data\ZXD\DATA\20240411-ZXD-1\zxd-1F_LC 2024-04-12 00-07-33\20240316--ZK-
                  15MIN-35-95ACN.M
Last changed    : 4/12/2024 12:05:51 AM by BY
Analysis Method : D:\DATA\KUN\METHOD\20240204-ZK-15MIN-25-90ACN.M
Last changed    : 4/12/2024 4:21:22 PM by BY
                  (modified after loading)
Method Info     : 90% H2O and 10% ACE

Additional Info : Peak(s) manually integrated
```

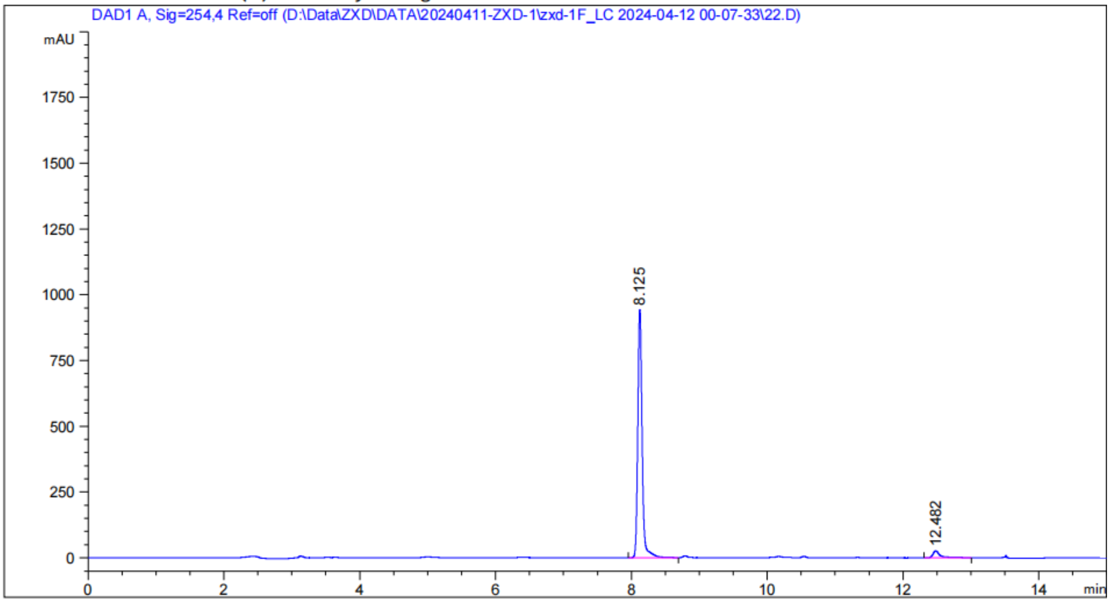


```
=====================================================================
                         Area Percent Report
=====================================================================

Sorted By             :      Signal
Multiplier            :      1.0000
Dilution              :      1.0000
Do not use Multiplier & Dilution Factor with ISTDs


Signal 1: DAD1 A, Sig=254,4 Ref=off
```

Peak #	RetTime [min]	Type	Width [min]	Area [mAU*s]	Height [mAU]	Area %
1	8.125	BV	0.0674	4189.67041	942.65955	95.3635
2	12.482	BB	0.1067	203.69757	26.95395	4.6365

Totals : 4393.36798 969.61349

Compound 11i

```
Acq. Operator   : BY                              Seq. Line :  22
Acq. Instrument : 1260R                            Location :   24
Injection Date  : 4/12/2024 5:55:35 AM                  Inj :   1
                                                Inj Volume : 20.000 µl
Acq. Method     : D:\Data\ZXD\DATA\20240411-ZXD-1\zxd-1F_LC 2024-04-12 00-07-33\20240316--ZK-
                  15MIN-35-95ACN.M
Last changed    : 4/12/2024 12:05:51 AM by BY
Analysis Method : D:\DATA\KUN\METHOD\20240204-ZK-15MIN-25-90ACN.M
Last changed    : 4/12/2024 4:21:22 PM by BY
                  (modified after loading)
Method Info     : 90% H2O and 10% ACE

Additional Info : Peak(s) manually integrated
```

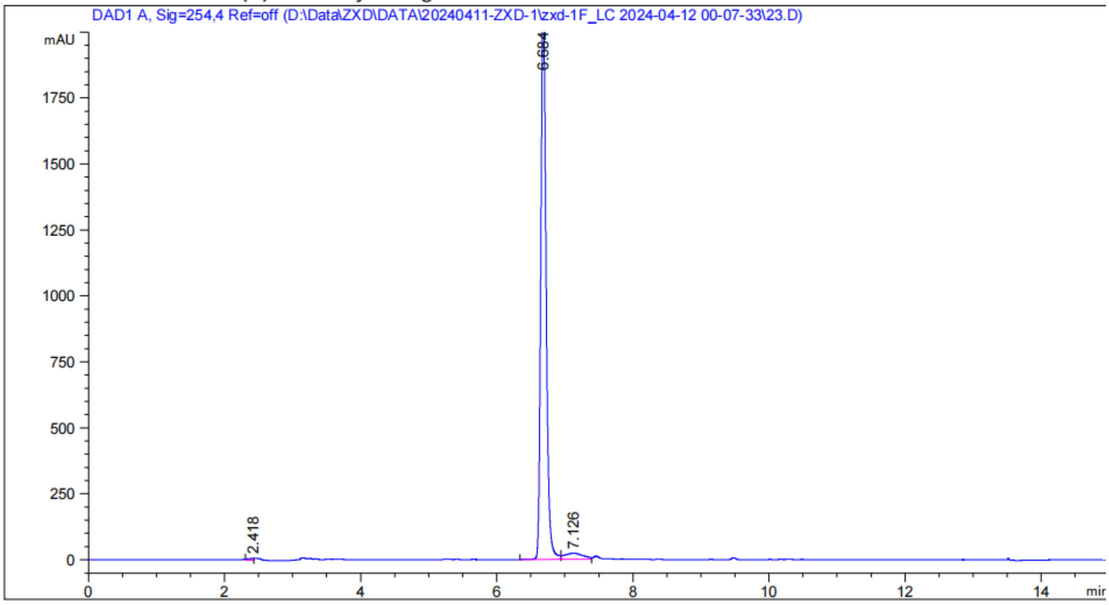


```
=====================================================================
                         Area Percent Report
=====================================================================

Sorted By             :      Signal
Multiplier            :      1.0000
Dilution              :      1.0000
Do not use Multiplier & Dilution Factor with ISTDs


Signal 1: DAD1 A, Sig=254,4 Ref=off
```

Peak #	RetTime [min]	Type	Width [min]	Area [mAU*s]	Height [mAU]	Area %
1	2.418	VV	0.0699	42.48051	7.46477	0.3580
2	6.684	BV	0.0828	1.13872e4	2116.10376	95.9565
3	7.126	VV	0.2310	437.36792	22.78071	3.6856

Totals : 1.18671e4 2146.34924

Compound 11j

```
Acq. Operator   : BY                              Seq. Line :   4
Acq. Instrument : 1260R                            Location :   36
Injection Date  : 4/12/2024 7:25:42 PM                  Inj :   1
                                                 Inj Volume : 20.000 µl
Different Inj Volume from Sample Entry!  Actual Inj Volume : 10.000 µl
Acq. Method     : D:\Data\ZLM\DATA\20240412\20240412 2024-04-12 18-34-58\20240316--ZK-15MIN-
                  35-95ACN.M
Last changed    : 4/12/2024 12:05:51 AM by BY
Analysis Method : D:\DATA\KUN\METHOD\20240204-ZK-15MIN-25-90ACN.M
Last changed    : 4/12/2024 4:57:03 PM by BY
                  (modified after loading)
Method Info     : 90% H2O and 10% ACE

Additional Info : Peak(s) manually integrated
```

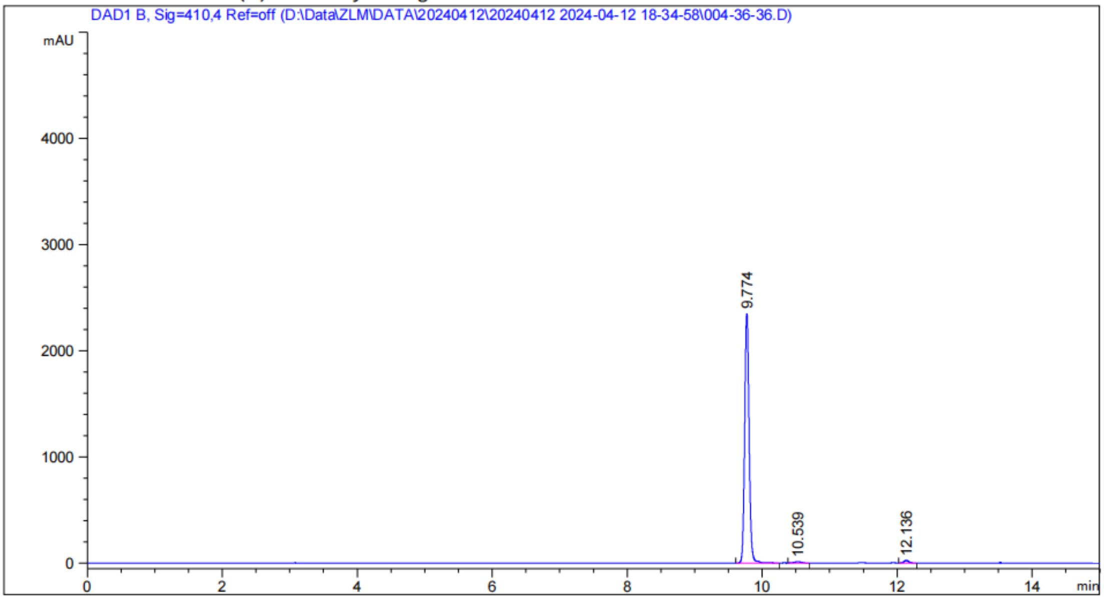


```
=====================================================================
                         Area Percent Report
=====================================================================

Sorted By             :      Signal
Multiplier            :      1.0000
Dilution              :      1.0000
Do not use Multiplier & Dilution Factor with ISTDs


Signal 1: DAD1 B, Sig=410,4 Ref=off
```

Peak #	RetTime [min]	Type	Width [min]	Area [mAU*s]	Height [mAU]	Area %
1	9.774	BV	0.0701	1.06882e4	2349.57568	98.0519
2	10.539	VB	0.0961	88.99992	11.33529	0.8165
3	12.136	VV	0.0782	123.35857	24.29147	1.1317

Peak #	RetTime [min]	Type	Width [min]	Area [mAU*s]	Height [mAU]	Area %
Totals :				1.09005e4	2385.20244	

Compound 11k

```
Acq. Operator   : BY                              Seq. Line :    8
Acq. Instrument : 1260R                            Location :   68
Injection Date  : 4/18/2024 10:34:16 PM                 Inj :    1
                                                 Inj Volume : 20.000 µl
Different Inj Volume from Sample Entry!  Actual Inj Volume : 3.000 µl
Acq. Method     : D:\Data\ZLM\DATA\20240416-2\20240416 2024-04-18 20-25-58\20240316--ZK-15MIN
                  -35-95ACN.M
Last changed    : 4/12/2024 12:05:51 AM by BY
Analysis Method : D:\DATA\ZLM\METHOD\AB-ACN-H2O(5-90)15MIN.M
Last changed    : 4/19/2024 12:08:24 AM by BY
                  (modified after loading)
Method Info     : HYDRO RP-80A

Additional Info : Peak(s) manually integrated
```

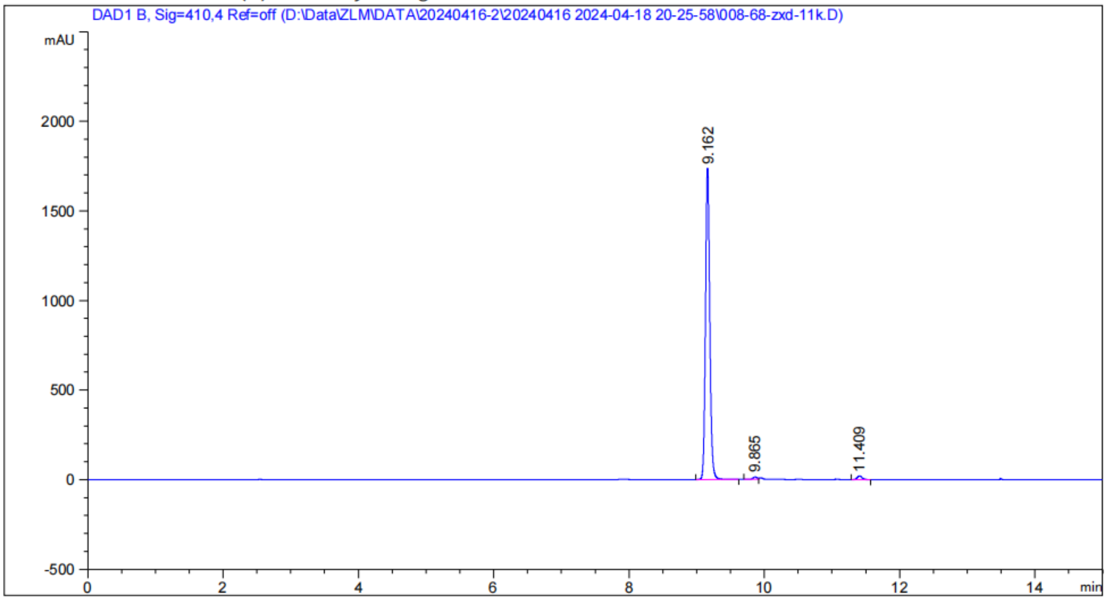


```
=====================================================================
                         Area Percent Report
=====================================================================

Sorted By             :      Signal
Multiplier            :      1.0000
Dilution              :      1.0000
Do not use Multiplier & Dilution Factor with ISTDs


Signal 1: DAD1 B, Sig=410,4 Ref=off
```

Peak #	RetTime [min]	Type	Width [min]	Area [mAU*s]	Height [mAU]	Area %
1	9.162	BB	0.0680	7670.71484	1736.94189	97.9726
2	9.865	BV	0.0663	61.24231	13.92678	0.7822
3	11.409	BB	0.0696	97.49361	21.03873	1.2452

Peak #	RetTime [min]	Type	Width [min]	Area [mAU*s]	Height [mAU]	Area %
Totals :				7829.45077	1771.90741	

Compound 12a

```
Acq. Operator   : BY                             Seq. Line :   25
Acq. Instrument : 1260R                           Location :   27
Injection Date  : 4/12/2024 6:45:04 AM                 Inj :    1
                                               Inj Volume : 20.000 µl
Acq. Method     : D:\Data\ZXD\DATA\20240411-ZXD-1\zxd-1F_LC 2024-04-12 00-07-33\20240316--ZK-
                  15MIN-35-95ACN.M
Last changed    : 4/12/2024 12:05:51 AM by BY
Analysis Method : D:\DATA\KUN\METHOD\20240204-ZK-15MIN-25-90ACN.M
Last changed    : 4/12/2024 4:29:43 PM by BY
                  (modified after loading)
Method Info     : 90% H2O and 10% ACE

Additional Info : Peak(s) manually integrated
```

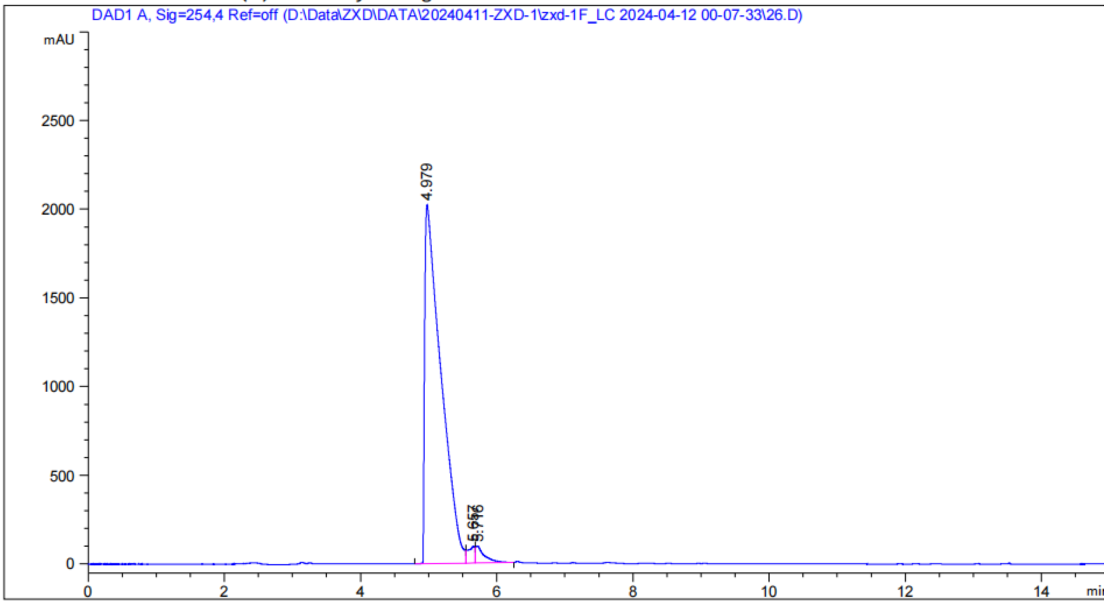


```
=====================================================================
                         Area Percent Report
=====================================================================

Sorted By             :      Signal
Multiplier            :      1.0000
Dilution              :      1.0000
Do not use Multiplier & Dilution Factor with ISTDs


Signal 1: DAD1 A, Sig=254,4 Ref=off

Peak RetTime Type  Width     Area      Height     Area
  #   [min]        [min]   [mAU*s]     [mAU]        %
----|-------|----|-------|----------|----------|--------|
   1   4.979 BV    0.2150 3.35267e4 2021.52502 95.6116
   2   5.657 VV    0.0903  648.21887   94.14844   1.8486
   3   5.716 VB    0.1238  890.58801   95.96742   2.5398

Totals :                  3.50655e4  2211.64088
```

Compound 12b

```
Acq. Operator   : BY                              Seq. Line :  26
Acq. Instrument : 1260R                            Location :   28
Injection Date  : 4/12/2024 7:01:33 AM                  Inj :   1
                                                Inj Volume : 20.000 µl
Acq. Method     : D:\Data\ZXD\DATA\20240411-ZXD-1\zxd-1F_LC 2024-04-12 00-07-33\20240316--ZK-
                  15MIN-35-95ACN.M
Last changed    : 4/12/2024 12:05:51 AM by BY
Analysis Method : D:\DATA\KUN\METHOD\20240204-ZK-15MIN-25-90ACN.M
Last changed    : 4/12/2024 4:33:41 PM by BY
                  (modified after loading)
Method Info     : 90% H2O and 10% ACE

Additional Info : Peak(s) manually integrated
```

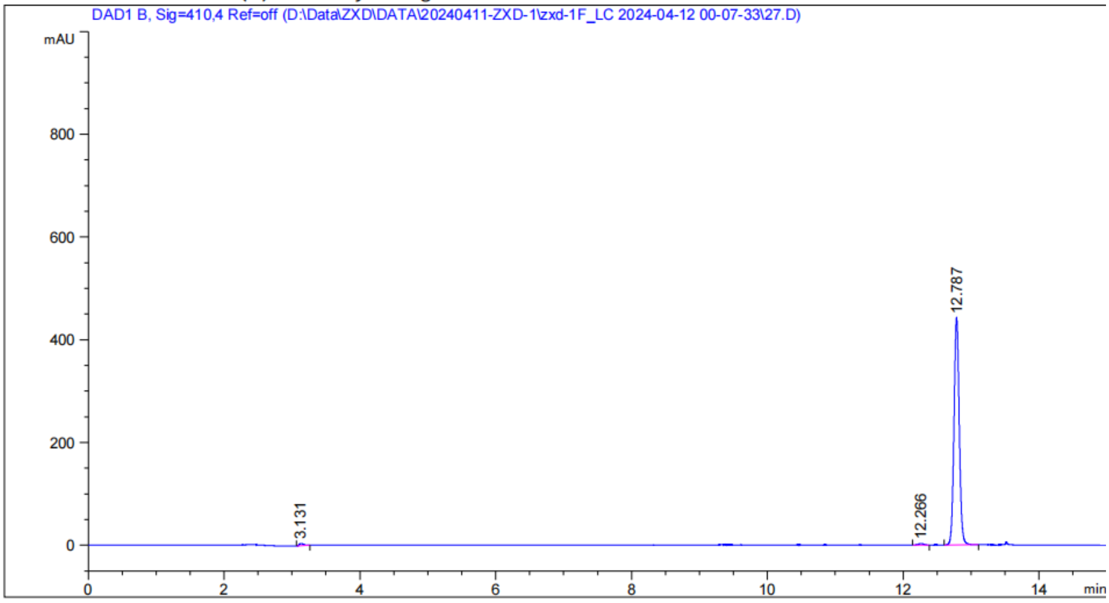


```
=====================================================================
                         Area Percent Report
=====================================================================

Sorted By             :      Signal
Multiplier            :      1.0000
Dilution              :      1.0000
Do not use Multiplier & Dilution Factor with ISTDs


Signal 1: DAD1 B, Sig=410,4 Ref=off

Peak RetTime Type  Width     Area      Height     Area
  #   [min]        [min]   [mAU*s]     [mAU]       %
----|-------|----|-------|----------|----------|--------|
   1   3.131 BB    0.0561   18.49863    4.14327   0.7709
   2  12.266 BB    0.0671   14.86164    2.83681   0.6194
   3  12.787 BV    0.0819 2366.18555  442.62436  98.6097

Totals :                  2399.54582  449.60443
```

Compound 12c

```
Acq. Operator   : BY                              Seq. Line :    2
Acq. Instrument : 1260R                            Location :   38
Injection Date  : 4/12/2024 9:10:26 PM                  Inj :    1
                                                Inj Volume : 20.000 µl
Acq. Method     : D:\Data\ZLM\DATA\20240412\20240412 2024-04-12 20-51-13\20240316--ZK-15MIN-
                  35-95ACN.M
Last changed    : 4/12/2024 12:05:51 AM by BY
Analysis Method : D:\DATA\KUN\METHOD\20240204-ZK-15MIN-25-90ACN.M
Last changed    : 4/12/2024 9:34:52 PM by BY
                  (modified after loading)
Method Info     : 90% H2O and 10% ACE

Additional Info : Peak(s) manually integrated
```

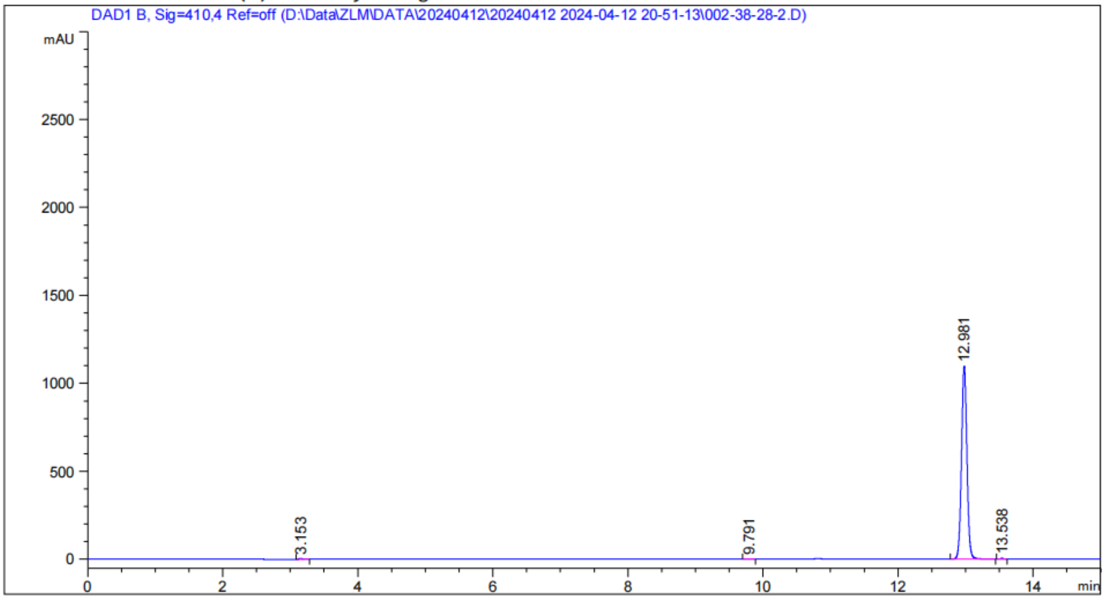


```
=====================================================================
                         Area Percent Report
=====================================================================

Sorted By             :      Signal
Multiplier            :      1.0000
Dilution              :      1.0000
Do not use Multiplier & Dilution Factor with ISTDs


Signal 1: DAD1 B, Sig=410,4 Ref=off
```

Peak #	RetTime [min]	Type	Width [min]	Area [mAU*s]	Height [mAU]	Area %
1	3.153	BB	0.0552	18.80663	4.12496	0.3054
2	9.791	BV	0.0562	6.40597	1.39158	0.1040
3	12.981	BB	0.0861	6123.86914	1097.54834	99.4461
4	13.538	BB	0.0213	8.89822	6.35827	0.1445

Compound 12d

```
Acq. Operator   : BY                              Seq. Line :  28
Acq. Instrument : 1260R                            Location :   30
Injection Date  : 4/12/2024 7:34:39 AM                  Inj :   1
                                                 Inj Volume : 20.000 µl
Acq. Method     : D:\Data\ZXD\DATA\20240411-ZXD-1\zxd-1F_LC 2024-04-12 00-07-33\20240316--ZK-
                  15MIN-35-95ACN.M
Last changed    : 4/12/2024 12:05:51 AM by BY
Analysis Method : D:\DATA\KUN\METHOD\20240204-ZK-15MIN-25-90ACN.M
Last changed    : 4/12/2024 4:50:25 PM by BY
                  (modified after loading)
Method Info     : 90% H2O and 10% ACE

Additional Info : Peak(s) manually integrated
```

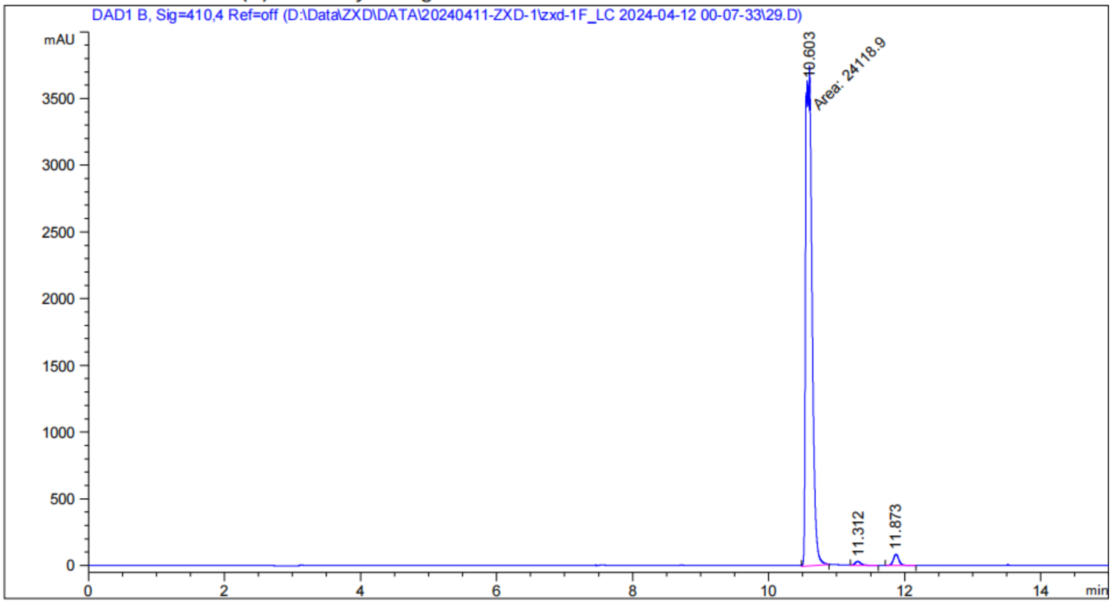


```
=====================================================================
                         Area Percent Report
=====================================================================

Sorted By             :      Signal
Multiplier            :      1.0000
Dilution              :      1.0000
Do not use Multiplier & Dilution Factor with ISTDs


Signal 1: DAD1 B, Sig=410,4 Ref=off
```

Peak #	RetTime [min]	Type	Width [min]	Area [mAU*s]	Height [mAU]	Area %
1	10.603	MM	0.1074	2.41189e4	3743.87427	97.4569
2	11.312	VB	0.0855	170.47946	29.24509	0.6889
3	11.873	BB	0.0845	458.89401	83.68165	1.8542

Compound 12e

```
Acq. Operator   : BY                            Seq. Line :    9
Acq. Instrument : 1260R                          Location :   69
Injection Date  : 4/18/2024 10:50:43 PM               Inj :    1
                                               Inj Volume : 20.000 µl
Different Inj Volume from Sample Entry!  Actual Inj Volume : 3.000 µl
Acq. Method     : D:\Data\ZLM\DATA\20240416-2\20240416 2024-04-18 20-25-58\20240316--ZK-15MIN
                  -35-95ACN.M
Last changed    : 4/12/2024 12:05:51 AM by BY
Analysis Method : D:\DATA\ZLM\METHOD\AB-ACN-H2O(5-90)15MIN.M
Last changed    : 4/19/2024 12:09:41 AM by BY
                  (modified after loading)
Method Info     : HYDRO RP-80A

Additional Info : Peak(s) manually integrated
```

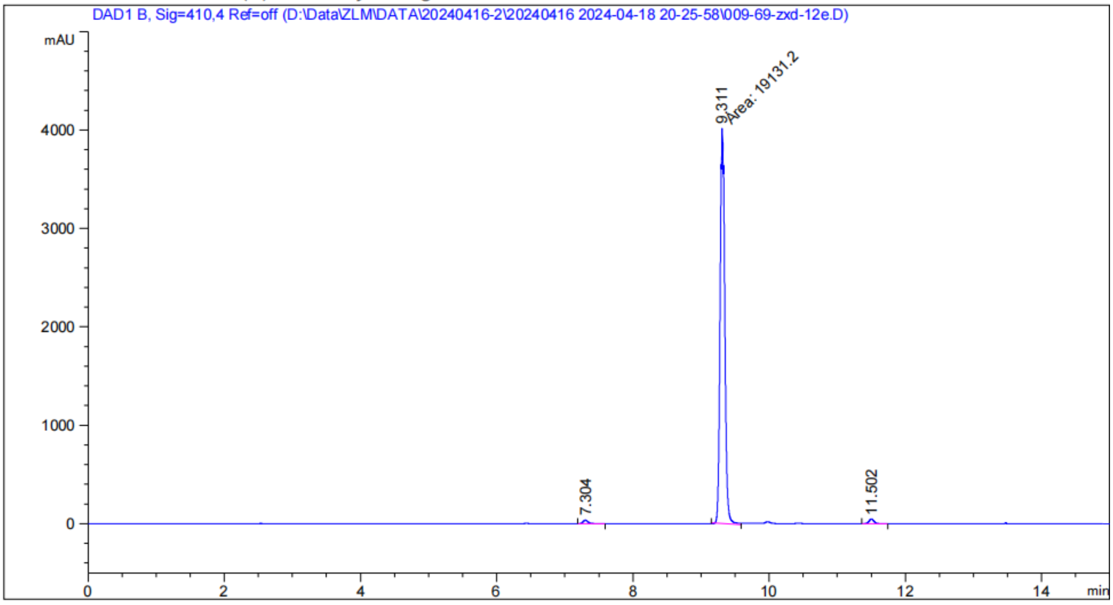


```
=====================================================================
                         Area Percent Report
=====================================================================

Sorted By             :      Signal
Multiplier            :      1.0000
Dilution              :      1.0000
Do not use Multiplier & Dilution Factor with ISTDs


Signal 1: DAD1 B, Sig=410,4 Ref=off

Peak RetTime Type  Width     Area      Height     Area
  #   [min]        [min]   [mAU*s]     [mAU]        %
----|-------|----|-------|----------|----------|--------|
   1   7.304 BB    0.0712  166.36632   34.84110   0.8523
   2   9.311 MM    0.0793 1.91312e4  4023.17847  98.0124
   3  11.502 BB    0.0716  221.59727   47.35620   1.1353

Peak RetTime Type  Width     Area      Height     Area
  #   [min]        [min]   [mAU*s]     [mAU]        %
----|-------|----|-------|----------|----------|--------|
Totals :                   1.95192e4  4105.37577
```

Compound 12f

```
Acq. Operator   : BY                          Seq. Line :  30
Acq. Instrument : 1260R                        Location :   32
Injection Date  : 4/12/2024 8:07:36 AM              Inj :   1
                                            Inj Volume : 20.000 µl
Acq. Method     : D:\Data\ZXD\DATA\20240411-ZXD-1\zxd-1F_LC 2024-04-12 00-07-33\20240316--ZK-
                  15MIN-35-95ACN.M
Last changed    : 4/12/2024 12:05:51 AM by BY
Analysis Method : D:\DATA\KUN\METHOD\20240204-ZK-15MIN-25-90ACN.M
Last changed    : 4/12/2024 4:57:03 PM by BY
                  (modified after loading)
Method Info     : 90% H2O and 10% ACE

Additional Info : Peak(s) manually integrated
```

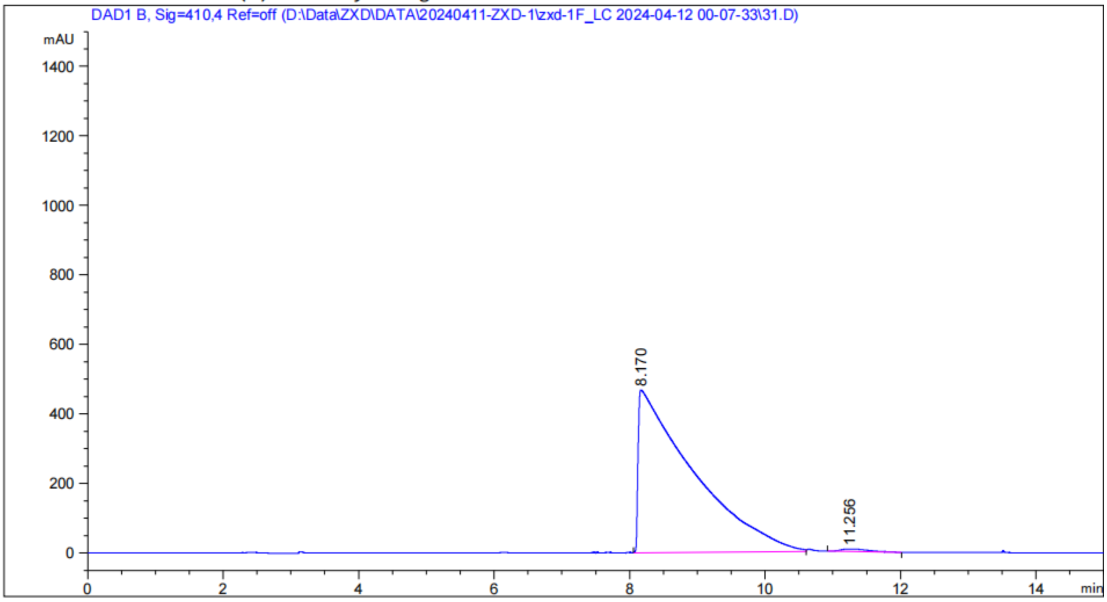


```
=====================================================================
                         Area Percent Report
=====================================================================

Sorted By             :      Signal
Multiplier            :      1.0000
Dilution              :      1.0000
Do not use Multiplier & Dilution Factor with ISTDs


Signal 1: DAD1 B, Sig=410,4 Ref=off
```

Peak #	RetTime [min]	Type	Width [min]	Area [mAU*s]	Height [mAU]	Area %
1	8.170	BV	0.6742	2.61960e4	467.89276	99.2207
2	11.256	BB	0.3338	205.75861	7.22474	0.7793

Totals : 2.64017e4 475.11750

References

1. Costa G, Rocca R, Corona A, Grandi N, Moraca F, Romeo I, Talarico C, Gagliardi MG, Ambrosio FA, Ortuso F, Alcaro S, Distinto S, Maccioni E, Tramontano E, Artese A. Novel natural non-nucleoside inhibitors of HIV-1 reverse transcriptase identified by shape- and structure-based virtual screening techniques. *Eur J Med Chem*. **2019**, 1(161), 1-10.
2. Corona A, Meleddu R, Esposito F, Distinto S, Bianco G, Masaoka T, Maccioni E, Menéndez-Arias L, Alcaro S, Le Grice SF, Tramontano E. Ribonuclease H/DNA Polymerase HIV-1 Reverse Transcriptase Dual Inhibitor: Mechanistic Studies on the Allosteric Mode of Action of Isatin-Based Compound RMNC6. PLoS One. **2016**, *11*, (1), e0147225.
3. Corona A, Schneider A, Schweimer K, Rösch P, Wöhrl BM, Tramontano E. Inhibition of foamy virus reverse transcriptase by human immunodeficiency virus type 1 RNase H inhibitors. Antimicrob Agents Chemother. **2014**, *58*, (7), 4086-4093.
4. Messore A, Corona A, Madia VN, Saccoliti F, Tudino V, De Leo A, Scipione L, De Vita D, Amendola G, Di Maro S, Novellino E, Cosconati S, Métifiot M, Andreola ML, Valenti P, Esposito F, Grandi N, Tramontano E, Costi R, Di Santo R. Pyrrolyl Pyrazoles as Non-Diketo Acid Inhibitors of the HIV-1 Ribonuclease H Function of Reverse Transcriptase. *ACS Med Chem Lett*. **2020**, *11*, (5), 798-805.
